# Ultrasensitive deletion detection links mitochondrial DNA replication, disease, and aging

**DOI:** 10.1186/s13059-020-02138-5

**Published:** 2020-09-17

**Authors:** Scott A. Lujan, Matthew J. Longley, Margaret H. Humble, Christopher A. Lavender, Adam Burkholder, Emma L. Blakely, Charlotte L. Alston, Grainne S. Gorman, Doug M. Turnbull, Robert McFarland, Robert W. Taylor, Thomas A. Kunkel, William C. Copeland

**Affiliations:** 1 Genome Integrity and Structural Biology Laboratory, DNA Replication Fidelity Group, National Institute of Environmental Health Sciences, National Institutes of Health, Research Triangle Park, NC 27709 USA; 2 Genome Integrity and Structural Biology Laboratory, Mitochondrial DNA Replication Group, National Institute of Environmental Health Sciences, National Institutes of Health, Research Triangle Park, NC 27709 USA; 3 Integrative Bioinformatics, National Institute of Environmental Health Sciences, National Institutes of Health, Research Triangle Park, NC 27709 USA; 4 Wellcome Centre for Mitochondrial Research, Translational and Clinical Research Institute, Faculty of Medical Sciences, Newcastle University, Newcastle upon Tyne, NE2 4HH UK; 5 NHS Highly Specialised Mitochondrial Diagnostic Laboratory, Newcastle upon Tyne Hospitals NHS Foundation Trust, Newcastle upon Tyne, NE1 4LP UK

## Abstract

**Background:**

Acquired human mitochondrial genome (mtDNA) deletions are symptoms and drivers of focal mitochondrial respiratory deficiency, a pathological hallmark of aging and late-onset mitochondrial disease.

**Results:**

To decipher connections between these processes, we create LostArc, an ultrasensitive method for quantifying deletions in circular mtDNA molecules. LostArc reveals 35 million deletions (~ 470,000 unique spans) in skeletal muscle from 22 individuals with and 19 individuals without pathogenic variants in POLG. This nuclear gene encodes the catalytic subunit of replicative mitochondrial DNA polymerase γ. Ablation, the deleted mtDNA fraction, suffices to explain skeletal muscle phenotypes of aging and POLG-derived disease. Unsupervised bioinformatic analyses reveal distinct age- and disease-correlated deletion patterns.

**Conclusions:**

These patterns implicate replication by DNA polymerase γ as the deletion driver and suggest little purifying selection against mtDNA deletions by mitophagy in postmitotic muscle fibers. Observed deletion patterns are best modeled as mtDNA deletions initiated by replication fork stalling during strand displacement mtDNA synthesis.

## Introduction

**Fig. 1:**
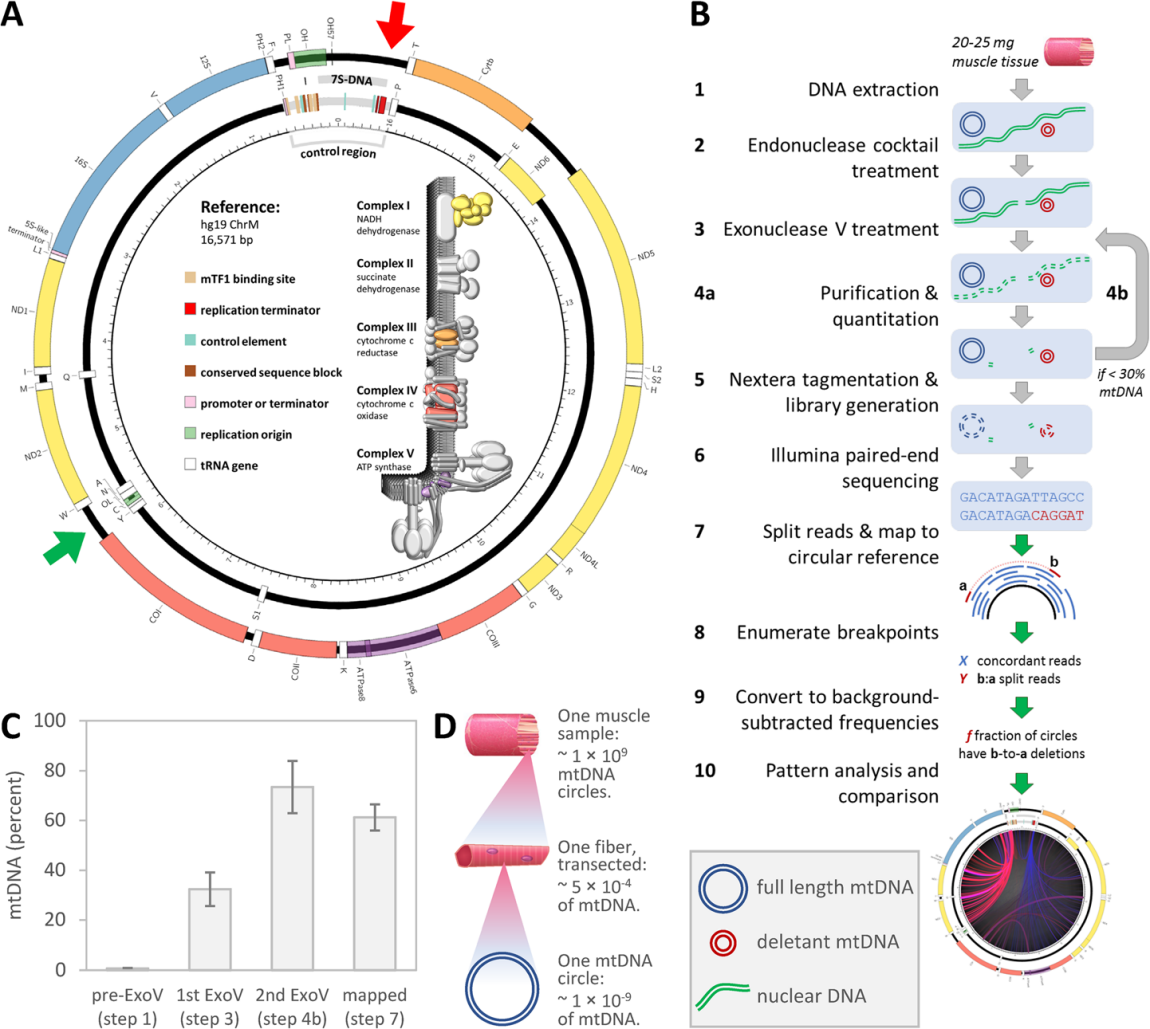
The human mtDNA reference map and the mtDNA deletion mapping pipeline. **a** Human mtDNA reference map, color-coded by feature. Inset: respiratory chain complexes encoded by mtDNA (color) and nuclear genes (gray). MtDNA-encoded genes and feature abbreviations: tRNA genes, IUPAC single-letter amino acid codes; rRNA genes, sedimentation coefficients (e.g., 16S); previously proposed heavy and light strand features, _H and _L; promoters, P_; replication origins, O_ (e.g., PH1, OL); and *MT-* prefixes are omitted. The 7S-3′-terminus and *oriL* are indicated (red and green arrows). **b** LostArc method outline. **c** The mtDNA fractions (mean ± 95% CL) indicate enrichment during library preparation and a subsequent lack of selection in sequencing (qPCR for steps 1, 3, and 4b, fraction of reads mapped to mtDNA reference for step 7). See also Additional file 1: Fig. S1. **d** Estimated mtDNA frequencies. Species with frequencies over 5 × 10^−4^ must reside in multiple truncated fibers (i.e., they predate adulthood or mark mutational hotspots). Deletions observed ≥ 2x must have ≥ 20 copies in the muscle sample. Deletions observed once will have frequencies inflated to roughly 1/depth. These collectively represent all less frequent species

Along with many other vital functions, mitochondria generate most of the cell’s energy through aerobic respiration. In humans, as in most eukaryotes, mitochondria possess their own indispensable genome (mtDNA) which replicates separately from the nuclear genome. Human mtDNA is a 16.6-kb closed circular molecule encoding 37 genes required to make 13 essential subunits of the oxidative phosphorylation (OXPHOS) complexes, as well as critical elements of the mitochondrial translational machinery (Fig. 1a). OXPHOS drives adenosine triphosphate (ATP) production, generating energy for cellular consumption. Each human cell contains hundreds to thousands of mtDNA copies existing in a mixture of wild type and mutated molecular species that is collectively called heteroplasmy [1–4]. Low levels of heteroplasmy have been detected in most healthy individuals [1], whereas high levels of heteroplasmy have been implicated in a broad range of neurometabolic disorders under the umbrella term of “mitochondrial diseases” [4–6].

Pathogenic variants of at least 24 nuclear genes whose protein products are responsible for mtDNA maintenance co-segregate with mitochondrial disease [7–10]. Two such genes (*POLG* and *POLG2*, respectively) encode the catalytic and accessory subunits of DNA polymerase γ (Pol γ). Pol γ is a family A DNA polymerase with 3′-5′ exonuclease and 5′-dRP lyase activities and the only known mitochondrial replicase [11, 12]. Pol γ works in concert with the Twinkle helicase, a mitochondrial ssDNA binding protein and other accessory factors to carry out efficient mtDNA replication [13, 14]. Pathogenic mutations in *POLG* are the most common cause of mitochondrial disease linked to improper maintenance of the mitochondrial genome [15]. Over three hundred missense mutations in *POLG* have been reported to cause a wide spectrum of mitochondrial diseases with ages of onset ranging from early (childhood myocerebrohepatopathy or Alpers-Huttenlocher syndrome) to late (ataxia neuropathies or progressive external ophthalmoplegia) [16, 17]. Pathogenic *POLG* variants encode proteins with assorted biochemical deficiencies, including catalytic deficits, structural instability, and defects in binding DNA or accessory proteins [14]. Early-onset *POLG* disorders are associated with a quantitative loss of mtDNA copies (mtDNA depletion), whereas later-onset *POLG* disorders are associated with accumulation of mtDNA deletions [17].

Point mutations and deletions in mtDNA accumulate with age and have been implicated in normal aging and age-related pathologies [18–23]. However, as assayed in human brain tissue, the accumulation of point mutations is orders of magnitude too low to explain the phenotypes of aging [24]. Prior work by this group had shown that point mutations in mtDNA do not limit the natural lifespan of mice [25]. These observations lead to the hypothesis that the greater genetic disruption caused by mtDNA deletions may be a significant contributor to mechanisms of aging. Disruption of Pol γ’s exonuclease function prevents proofreading of DNA replication errors [26], resulting in a 160-fold increase in mtDNA deletions in yeast [27]. In homozygous *POLG* exonuclease deficient mice [28, 29], random mutation capture assays identified mtDNA deletions as a causative factor in the premature aging phenotype [30]. The biochemical mechanisms underlying the formation of mtDNA deletions are not well defined. Several models have been proposed, including primer relocation between direct repeats during mtDNA replication [31, 32] and ectopic re-annealing of broken strands within short homologous DNA sequences [33].

To gain insight into the molecular mechanisms underlying formation of mtDNA deletions and to define the roles of deletions in aging and human mitochondrial disease, we sought to delineate the position, length, sequence context, and abundance of individual mtDNA deletions in human tissue samples. Because mtDNA represents only 0.93% of total DNA in human skeletal muscle, existing deletion detection techniques rely on PCR to selectively enrich for mtDNA sequences [34, 35]. In order to avoid PCR artifacts and target biasing due to primer selection, we developed LostArc, an ultra-sensitive high throughput deletion detection pipeline. Here, we used LostArc to analyze mtDNA sequences from skeletal muscle samples of mitochondrial disease patients bearing inherited pathogenic *POLG* variants and from age-matched individuals with wild type *POLG*. Hundreds of thousands of unique deletions were identified with frequencies sufficient to explain the musculoskeletal phenotypes of aging and disease. Patterns in the locations and sequence contexts suggest that DNA replication is the primary driver of deletion formation and strongly support asynchronous strand displacement models of mtDNA replication.

## Results

The LostArc method was applied to cultured human embryonic kidney cells (HEK-293) and 41 skeletal muscle biopsies (Additional file 1: Table S1): 19 biopsies with wild type *POLG* sequences (Gwt samples; biopsied at 17 to 93 years of age; GenBank NM_002693.2) and 22 samples with variant *POLG* sequences (Gvar; 17 to 80 years). The latter include 18 variant *POLG* alleles in 17 combinations with a continuum of clinical symptoms (Additional file 1: Table S2). Eleven of these variant samples have not been previously reported. Three variants (G952R, M797I, and T914P) and one genotypic combination (A467T *in trans* with S933R) were not found in the Leiden Open Variation Database [36], NCBI ClinVar [37], or the Human DNA Polymerase Gamma Mutation Database (https://tools.niehs.nih.gov/polg/) [38]. In total, 35 million mtDNA deletions were identified, relative to the reference sequence (Fig. 1a; GenBank AF347015.1), encompassing roughly 470,000 unique spans (dbGaP phs002052.v1.p1).

### The LostArc pipeline, its outputs, and the human mtDNA reference map

Genomic DNA was isolated from approximately 25 mg sample of each frozen skeletal muscle tissue (Fig. 1b step 1). Linear DNA, predominantly nuclear, from 0.2 to 2.0 μg of the resulting genomic DNA, was digested with Exonuclease V and a cocktail of seven restriction endonucleases that did not cut mtDNA (steps 2–3). Remaining DNA was column purified and concentrated, and the nuclear and mtDNA components were quantified via real-time PCR (step 4a). If the mtDNA was found to be under 30% of the total, then nuclease treatment and purification steps were repeated (step 4b). Sequencing libraries were prepared via transposon-mediated tagmentation using the minimum sufficient number of PCR amplification cycles, followed by purification and quantitation (step 5). Libraries were sequenced (step 6) and the resulting reads were filtered, trimmed, and then mapped to the circular human mtDNA reference via the ROTLA package (steps 7–8, see the “Materials and methods” section). Among all samples, the mtDNA fraction rose from an average of 0.85 ± 0.52% (*n* = 17; ± 95% CL) in whole DNA extracts to an average of 73 ± 11% at the beginning of library preparation. The final fraction of sequencing reads that mapped to the mtDNA genome was 61 ± 5.3%. Examples from individual samples are presented in Additional file 1: Figure S1a. Several samples, including one Gwt (M05), had lower mtDNA fractions than indicated by qPCR (Additional file 1: Fig. S1b). These samples were not unified by age or genotype, so the cause of this effect is unknown. Deletion counts were converted into deletion frequencies and their patterns were analyzed (steps 9–10). Individual deletion frequencies above 10^−6^ were highly reproducible (Additional file 1: Fig. S2).

LostArc was designed to measure accurately the individual frequencies of very rare deletant species and the total frequency of ultra-rare deletant species (boundary set at approximately 3 × 10^−5^ by the sensitivity achieved; see Methods). Library preparation and sequencing added no systematic bias for or against mtDNA (see Fig. 1c for averages, Additional file 1: Fig. S1 for individual samples; see Methods for details). High sequencing depth (Additional file 1: Table S1) allowed the LostArc method to call roughly one deletion per million mtDNA circles (10^–5.9 ± 0.09^; median ± 95% CL in the exponent). LostArc sensitivity exceeds that for sequencing after long-range PCR [39] but not for digital drop PCR (~ 10^−8^ [35, 40, 41];). Unlike digital drop PCR, which targets deletions at specific loci, LostArc assays the full deletion spectrum across the whole mitochondrial genome. LostArc captures a representative subset of uniquely deleted molecules (10^−9^ each) while easily mapping deletions that dominate one homoplasmic muscle fiber within a sample (~ 5 × 10^−4^, Fig. 1d; see Methods for details).

### Levels of mtDNA do not vary significantly with age

Estimates of mtDNA as a fraction of total DNA ranged from 0.01 to 4.5%. Among tested Gwt samples, the average mtDNA content is 0.78% (*n* = 6; examples in Additional file 1: Fig. S1a, blue bars), in line with previous estimates [34]. The Gvar samples we tested were not significantly different in this regard (mean 0.89%; *n* = 10; 1-tailed Welch’s *t* test *p* = 0.41). There is no significant linear relationship between age and mtDNA copy number (via qPCR; representative subset in Additional file 1: Fig. S1a; Gwt *R*^2^ = 0.44; Gvar *R*^2^ = 0.21).

### Levels of mtDNA ablation vary with age

**Fig. 2:**
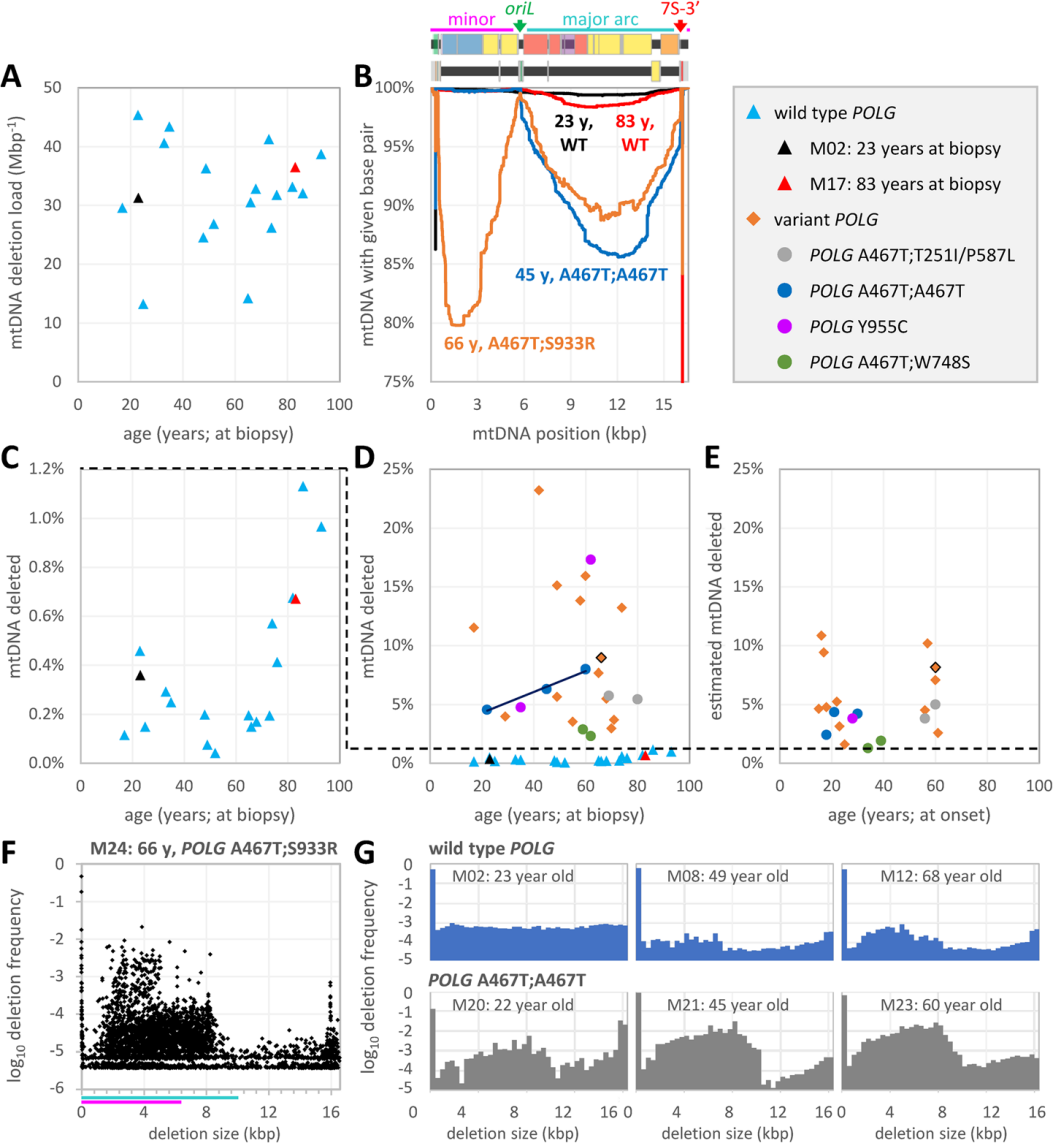
Deletion frequencies and ablation levels of mtDNA vary with age, disease, and mapped location. **a**–**e** Gwt muscle samples are represented by triangles, and Gvar samples are represented by circles. Among wild type samples, one young and one aged sample are highlighted (M03, biopsied at 23 years, in black and M17, biopsied at 83 years, in red, respectively). **a** Among Gwt muscle samples, deletion loads are independent of age (per Mbp mapped to mtDNA reference). **b** The fraction of mtDNA remaining for two Gwt and two Gvar samples. A linearized mtDNA map (see Fig. 1a) is shown above the graph with key features indicated: *oriL* (green); 7S-DNA 3′-end (red); minor arc (magenta); and major arc (teal). **c** Ablation (area above the curve in **b**) depends upon age, as seen in Gwt samples. **d** Ablation at biopsy is higher and more varied among Gvar samples. Ablation increases with age between well-spaced Gvar samples: A467T;A467T (dark blue); and heterozygous Y955C (purple). **e** The minimum estimated ablation at onset in Gvar samples exceeds the maximum observed ablation at biopsy in Gwt samples, implying a threshold for clinical symptoms (dashed line). **f** A deletion length spectrum example (1 bp bins; Gvar sample M24). Very large deletions (> 15 kbp) may indicate insertions, primarily within the control region. For all samples, see LostArc Reports in Supplemental Information (also see Additional file 1: Figs. S4 and S5). Major and minor arc sizes are shown below the histogram (teal and magenta). **g** Deletion length spectra (400 bp bins) for Gwt and variant (A467T;A467T) samples of similar ages

In human skeletal muscle cells, raw deletion loads do not correlate with age (Fig. 2a; *R*^2^ < 0.002). Raw deletion loads in Gwt samples ranged from 13.3 to 45.4 deletions per Mbp mapped to the mtDNA reference (Additional file 1: Table S1). On average, Gwt muscle (*n* = 19) has a higher mtDNA deletion load than cultured HEK cells (*n* = 3), but not significantly so (29 ± 7.9 Mbp^−1^ versus 32 ± 8.7 Mbp^−1^; ± SD; two-tailed Welch’s *t* test *p* = 0.30). Gvar samples showed higher average deletion loads (59 ± 23 Mbp^−1^, versus wild type muscle *p* < 10^−5^), but the variance is so high that 40% of Gvar samples had deletion loads lower than the highest Gwt sample. After correcting deletion load for deletant molecule size and renormalizing for differential mapping efficiencies across the mtDNA reference, the resulting deletion frequencies and missing arc lengths can be combined to estimate the degree of mtDNA lost to deletions. We define this degree as the mtDNA ablation level. Low mtDNA ablation in HEK cells may be due to mitophagy and purifying selection in an actively dividing culture. Ab initio, it is unclear what fraction of HEK deletions are artifactual, but the high rates of small deletions are in accord with known Illumina error rates. Therefore, taking the most conservative stance from this point forward, all deletion frequencies have the mean HEK frequencies subtracted (see Methods for calculations; a frequency floor is set at 0). A renormalized depth plot shows the remaining mtDNA (Fig. 2b). The area above each curve represents the mtDNA ablation level.

Ablation is nonuniform with respect to biopsy age in Gwt muscle samples (Fig. 2c; Additional file 1: Table S1; *p* < 0.05, Kolmogorov–Smirnov test). On average, samples biopsied before age 40 have higher and more varied ablation (0.27 ± 0.14%, ± 95% CL, *n* = 6) than those from age 40 to 73 (0.15 ± 0.059%, *n* = 7; two-tailed Welch’s *t* test *p* = 0.036). Ablation increases linearly after age 60 (Fig. 2c; *n* = 10, *R*^2^ = 0.84; *p* < 0.0002, based on one million simulations with observed population variance).

### Higher ablation is associated with pathogenic POLG variants

Ablation levels in Gvar muscles are higher and more varied than in Gwt muscles (Fig. 2d). Gvar ablation ranges from 2.3% ablation (M31, biopsy at 62 years) to 23% (M28, biopsy at 42 years). Samples from similarly aged patients (< 10 years difference at biopsy) with identical *POLG* genotypes have similar ablation levels (Fig. 2d; A467T;W748S, 2.3 to 2.9% ablation; A467T;T251I/P587L, 5.7 to 5.4%). Samples from patients with identical *POLG* genotypes but disparate ages (> 20 years) show ablation increasing with age (Fig. 2d; heterozygous Y955C, *n* = 2; A467T;A467T, *n* = 3, linear trend, *R*^2^ = 0.988). This suggests three nonexclusive *POLG*-dependent possibilities: (1) deletions accumulate over time, (2) the deletion rate increases with age, and/or (3) the deletion removal rate decreases with age.

Regardless of the mechanism, it follows that the ablation level must have been lower at the age of onset of symptoms than at the later age of biopsy for each muscle sample (Additional file 1: Table S1). We have attempted to estimate the levels of ablation at the age of onset of symptoms. The ablation versus time trends for the A467T;A467T and heterozygous Y955C data sets have different slopes, which imply either different ablation levels at birth or nonlinear accumulation in youth (Fig. 2d). Therefore, as a simple first approximation, ablation at onset was calculated by linear interpolation between 0% at birth and the measured ablation level at the age of biopsy (Fig. 2e). The minimum calculated ablation at disease onset (1.3% for M31) barely exceeds the maximum among Gwt samples (1.1% for 86-year-old M18). This threshold separating Gwt from Gvar is delineated in Fig. 2c-e (dashed line).

### Ablation patterns implicate replication and lack of mitophagy in deletion accumulation

Inspection of ablation patterns reveals the conspicuous retention of certain genomic features. Young Gwt samples show roughly uniform ablation. However, *oriL* and the 7S-DNA sequence are strongly preserved in Gvar and older Gwt muscles (Fig. 2b), suggesting these features may be either inherently refractory to deletion or benefit from positive selection. Absent a rational explanation for these DNA sequences to preclude deletion, we prefer a model in which these features are necessary for a deleted species to become detectable within the sample population. Because both *oriL* and the 3′end of the 7S-DNA (7S-3′) are considered replication origins in some mtDNA replication models, the ability to propagate deletant genomes may be essential. In contrast, deletions are frequently observed in genes that support electron transport and oxidative phosphorylation. Although mitophagy culls mitochondria that cannot maintain membrane potential [42], the failure to more fully retain these OXPHOS sequences implies incomplete or absent mitophagy in postmitotic muscle fibers.

### Ablation correlates with cytochrome c oxidase deficiency

The degree to which deletions affect individual mitochondrial systems may be estimated by mapping ablation across the mtDNA reference map (Fig. 1a) and then may be compared with observed phenotypes (Additional file 1: Fig. S3). First, we assume mitochondria within a muscle fiber are able to share gene products and metabolites through fusion dynamics and inter-mitochondrial complementation [43–45]. Second, whereas very frequent deletions must occupy multiple fibers (see the “Materials and methods”), infrequent deletions may also do so. For example, identical rare mtDNA deletions in neighboring fibers could originate early in muscle development, or through fusions with a shared myosatellite lineage, or through independent events at a deletion hotspot. The most conservative estimate of deletion-driven deficiency comes from treating all mitochondria in all fibers as a single pool with evenly distributed deletions and perfect metabolic complementation (see Additional file 2: LostArc Reports for individual samples; examples in Additional file 1: Figs. S4 and S5). Ablation of mitochondrial systems, individually and in multiplicative combinations, was compared with histological estimates of the fraction of fibers lacking cytochrome *c* oxidase (Complex IV) activity (COX-deficient fibers (COX-ve) %; Additional file 1: Table S1). The best correlation, explaining 74.5% of COX-ve variation (Additional file 1: Fig. S3a; *p* < 10^−11^, *F*-test of least squares linear regression), was produced by considering deletion of COX genes and transcriptional/translational machinery (tRNA genes, rRNA genes, promoters and terminators; Additional file 1: Figs. S3a and S3b).

### Deletion length spectra vary with age and disease

Deletion lengths are not distributed uniformly across all possible sizes. An example deletion length spectrum for sample M24 is shown in Fig. 2f (66 years at biopsy; *POLG* A467T;S933R). Small deletions (< 100 bp) are very frequent in all samples (see Additional file 2: LostArc Reports for all samples). Since small deletions are markedly more frequent in muscle samples than in HEK cultures, they are included in overall deletion load and ablation calculations after HEK background subtraction (as in Fig. 2a). However, since small insertions and deletions are also the most frequent artifact of short-read sequencing technologies (e.g., [46]), they were excluded from position- or context-specific calculations unless otherwise noted. Most other deletions are confined by the minor and major arcs, respectively (magenta and teal in Fig. 2b and f). Very large deletions are also observed (> 15 kbp). These are discussed below.

In Gwt samples, since mtDNA ablation increases linearly after age 60 (Fig. 2c; *n* = 10, *R*^2^ = 0.84), but deletion loads are independent of age (Fig. 2a), then deletion sizes (e.g., M24 in Fig. 2f) must change instead. Age-related trends in deletion length spectra are illustrated in Fig. 2g by the three homozygous *POLG* A467T samples and three age-matched Gwt samples (22 to 68 years at biopsy). The youngest sample in each set exhibits the most uniform deletion length spectrum. Large deletions are less frequent in middle-aged versus younger wild type samples, but such deletions become more frequent with further age and/or *POLG* disease, especially within the major arc size range (e.g., Fig. 2g). Many deletions are so long that they must eliminate either *oriL* or the 7S-3′. These may represent large populations of non-replicating mtDNA circles in the oldest wild type muscle samples. These deletion length spectra are broadly representative (see Additional file 2: LostArc Reports for individual spectra).

### Deletion sequence contexts change with age and disease

Deletions were cataloged by length, frequency of occurrence, position within the genome, and for the presence and length of terminal microhomology (TMH). TMH is defined here as small patches of identical DNA sequence that could support relocation of nascent DNA primers between deletion termini. This classification relies entirely on sequence complementarity and is agnostic to the directionality of potential deletion mechanisms.

**Fig. 3:**
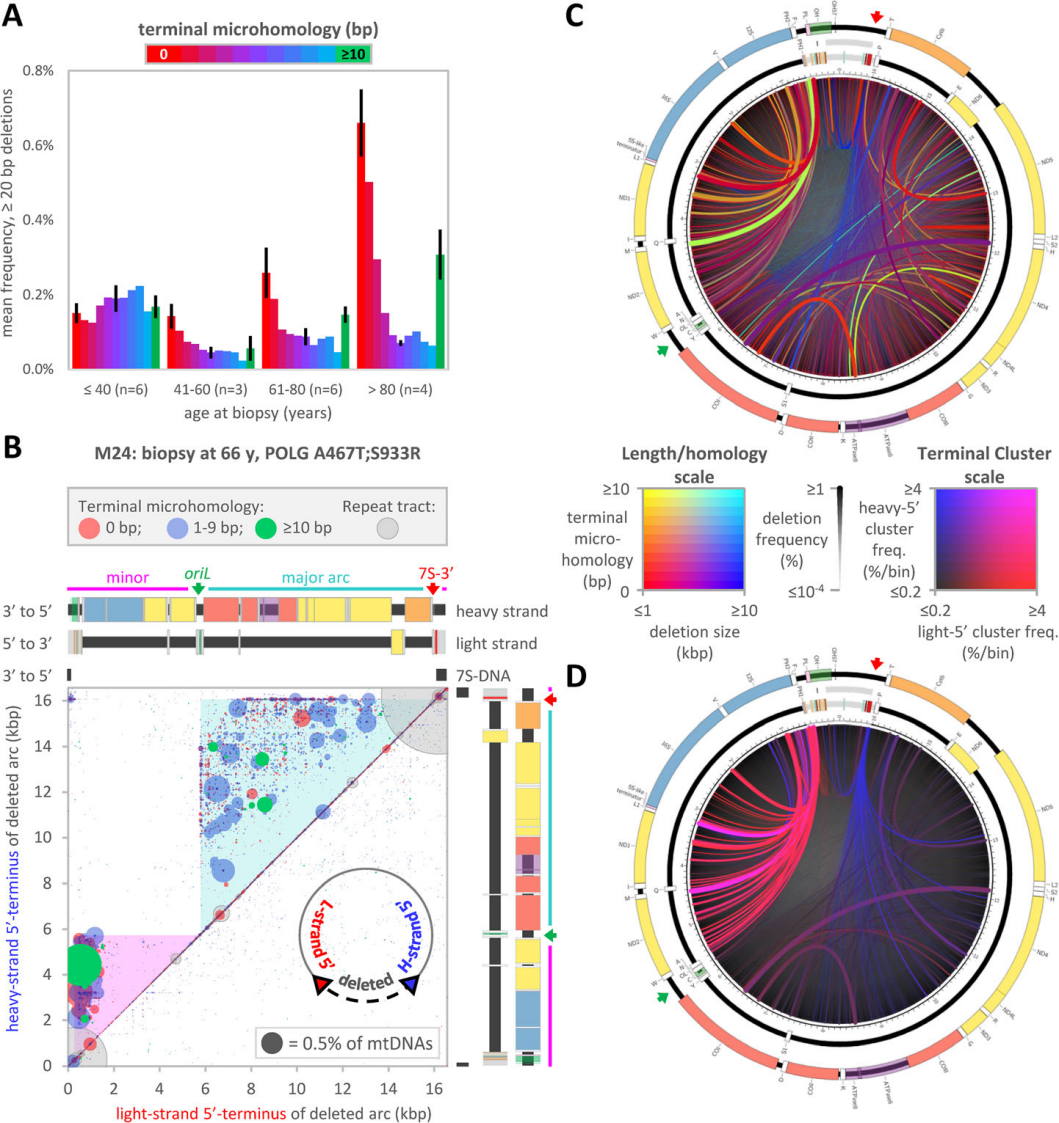
Size, position, frequency, and context of mtDNA deletions. **a** Frequencies of ≥ 20 bp deletions by Gwt age cohort and colored by degree of terminal microhomology (TMH; red to blue for 0 to 9 bp; green for ≥ 10 bp). TMH refers to identical sequences found in one deletion terminus and in the region flanking the other terminus. For simplicity, error bars (standard error) are only shown for 0, 5, and ≥ 10 bp of TMH. **b**–**d** Example visualizations of deletion frequencies by subsets of size, position, frequency, and sequence context (Gvar sample M24) with versions of the mtDNA map (**b** linear; **c**, **d** circular). The 7S-3′ terminus and *oriL* are indicated by red and green arrows. **b** A Bubble Map of deletions by terminal positions. Termini are classified starting from the center of a deletion (see inset diagram): one terminus lies in the light strand 5′-direction (red) and the other lies in the heavy strand 5′-direction (blue). Bubble area scaled by deletion frequency and colored by surrounding repeat tract (gray) or by the length of TMH: 0 bp (red); 1–9 bp (blue); ≥ 10 bp (green). The diagonal contains deletions of under 100 bp (peak in Fig. 2f). Minor and major arc deletions fall in the magenta and cyan areas (broad overlaid ranges of peaks in Fig. 2f). Plots equivalent to **b** may be found for all samples in Additional file 2: LostArc Reports (also see Additional file 1: Figs. S4 and S5). **c** An Arc Map with data as in **b**. Line width and transparency code for frequency (see deletion frequency key). Colors code for deletion size and terminal homology (see Length/homology key). Note the paucity of extensive TMH. The thin green arc from positions 8471 to 13,447 has been termed the common deletion. **d** As per **c**, but colors code for the degree of deletion terminus clustering (see Terminal Cluster key). Plots equivalent to **c**, **d** may be found for Gvar samples in Supplemental LostArc Arc Maps

The degree of TMH differs with age and *POLG* genotype. The 19 Gwt samples were divided by age into four cohorts in 20-year increments, counting down from 100 years, and then frequencies of deletions of at least 20 bp were averaged (Fig. 3a; individual samples in Additional file 2: LostArc Reports). The following differences are significant (at *α* ≤ 0.05; Welch’s *t* test with Šidák correction for multiple hypothesis testing): deletions with 0–3 bp of TMH are more frequent after 80 years, deletions with 4–9 bp of TMH are more frequent before 40 years, and deletions with ≥ 10 bp of TMH are more frequent after 80 years than between 41 and 80 years. In the youngest cohort, deletion frequencies are relatively uniform regardless of TMH length. In each of eleven Gwt samples, including the seven youngest, over 80% of deletions have at least one base pair of TMH. Only one Gvar sample has such a high proportion of deletions with TMH (M33; 71 years at biopsy, heterozygous *POLG* G952R). In contrast, in the 41–60-year Gwt cohort, deletions with TMH of either ≤ 2 bp or ≥ 10 bp begin to dominate the deletion pool. These species continue to increase in frequency in older cohorts, and deletions with ≤ 2 bp TMH outpace deletions with ≥ 10 bp TMH. Less than half of the deletions have any TMH in each of the two oldest Gwt samples and in 15 of 22 Gvar samples. As in Gwt samples, the fraction of deletions with ≥ 10 bp of TMH increases with age among the three homozygous *POLG* A467T samples. Therefore, the proportion of deletions with TMH decreases with both age and *POLG* defects.

### Visualizing the size, position, frequency, and context of mtDNA deletions

MtDNA deletions are uniquely identified by two points in the circular genome. To remain unbiased about the directionality of deletion formation, we chose to define the boundaries of each deletion as the positions of the last missing bases in the light-strand 5′-direction (LS5′) and the heavy-strand 5′-direction (HS5′; Fig. 3b inset; red and blue, respectively). Individual deletion locations are displayed here in two ways: Bubble Maps (example in Fig. 3b; all samples in Additional file 2: LostArc Reports) and Arc Maps (examples in Fig. 3c, d; all samples in Additional file 3: Arc Maps). In practice, we find circular Arc Maps more intuitive for visualizing some features (e.g., deletions that cross the control region, terminus clustering) and linear Bubble Plots more useful for other purposes (e.g., combining deletion length, position, TMH, and frequency in one plot).

Because few deletions traverse position 1 within the control region of mtDNA, the vast majority of deletions localize to the upper left half on Bubble Maps (e.g., M24; Fig. 3b). Across all samples, most deletions are further confined either to a narrow zone near the diagonal or to one of two triangular zones that are bounded by *oriL* (green arrow) and the 7S-DNA termini (7S-3′ red arrow). Deletions near the diagonal are small and often located within repeat tracts. This included the most frequent deletions, those within long poly-C-runs in the control region (large gray circles at positions 303 and 16,184), the lengths of which are highly polymorphic within the human population (MitoMap [47]). These poly-C-run deletions cause the deep, narrow ablation valleys at the extreme left and right edges of the ablation map (Fig. 2b), the narrow red spikes near the top of the Length/homology Arc Map (Fig. 3c), and the small deletion peak in the deletion length spectrum (≤ 100 bp, Fig. 2f). Deletions in the two triangular Bubble Map zones are confined to the minor and major arcs (Fig. 3b, magenta and cyan, respectively) and are responsible for the wide peaks in deletion length spectra (Fig. 2f) and the wide troughs in the ablation map (Fig. 2b). A small cluster of deletions in the upper left of the Bubble Map includes most of the very large deletions (> 15 kbp; Fig. 2f). However, when mapped to a circular reference sequence, split reads from the junctions of very large deletions are indistinguishable from those of insertions that exceed the read length. If these are truly large deletions, rather than insertions, then the remaining DNA would consist of micro-circles that include most of the control region and, usually, all of the 7S-DNA sequence. These large deletions appear as small blue arcs that straddle the gray 7S-DNA near the top of the Length/homology Arc Map (Fig. 3c). Other potential large deletions are visible either directly below the diagonal or in the white rectangular zone in the upper left portion of high-resolution versions of the Bubble Map (Fig. 3b). These are very rare mtDNA species that lack one or more potential replication origins.

Bubble Maps for other muscle samples differ in detail but are similar overall (see Additional file 2: LostArc Reports). Five of the six Gwt samples under age 40 have few off-diagonal (i.e., large) deletions that are frequent enough to be easily visible at reasonable resolution (exception: M05). Bubble Maps for Gwt samples between the ages of 40 and 80 are dominated by deletions in the major arc. A deletion spanning positions 8471 to 13,447 is often visible in these samples (Fig. 3b, green circle; Fig. 3c, green arc). This 4977 bp deletion is flanked by 13 nucleotide direct repeat sequences and is often referred to as the “common deletion” [48]. This deletion appears in most samples, generally at frequencies under 10^−3^. This is never the most frequent deletion (see control region poly-C-runs) and is rarely the most frequent deletion of over 20 bp (2 of 22 Gvars, 8 of 19 Gwt samples).

All Gvar Bubble Maps show a dense horizontal line of deletions with HS5′-termini at around position 16,070 near the 7S-3′ on the reference map. We call such a Bubble Map feature a deletion ridge. These deletions extend unidirectionally from the 7S-3′ into the major arc. Bubble Map deletion ridges appear as highly colored arc fountains in Terminal Cluster Arc Maps, such as the blue fountain issuing from near the 7S-3′ in the M24 example (Fig. 3d). Twenty of 22 Gvars (exceptions: M34 and M33) have a second, vertical deletion ridge with LS5′-termini near *oriL* at around position 5780. These deletions extend unidirectionally from *oriL* into the major arc. The corresponding red Terminal Cluster fountain is very faint for M24 (Fig. 3d). A clearer example, M38, is discussed later. Other deletions occur with decreasing frequencies as their termini venture farther from these two ridges/fountains. This apparent frequency gradient is punctuated by individual high frequency deletions at sites with extensive TMH, of which the common deletion is one example.

Particular samples have idiosyncratic features. For instance, M24 has, by far, the most frequent minor arc deletions, most of which emanate between positions 400 and 600 bp (Fig. 3b; Fig. 3d, pink fountains). Many of these deletions eliminate common PCR priming sites, such as the *MT-ND1* qPCR site used in the current work. Studies attempting to quantify mtDNA deletions through PCR from such sites would miss the most drastic effect on M24, the loss of components from at least 20% of its ribosomes (Additional file 2: LostArc Reports). Note the deletion from positions 537 to 4430, which by itself removes *MT-ND1* and both mt-rRNA genes from over 2% of mtDNA circles (Fig. 3b largest green circle; Fig. 3c, thickest green-yellow arc). At least 10 of 17 Gvar samples (depending on thresholds) have a third ridge/fountain with HS5′-termini in the major arc around position 13.9 kbp (horizontal, within the *MT-ND5* gene). One member of this group has at least three additional unique ridges/fountains, including one in the minor arc (M40, 35 years at biopsy, heterozygous *POLG* Y955C). Another has an unusually high frequency of very large deletions below the Bubble Map diagonal (M28, 42 years at biopsy, *POLG* A467T;R1096C).

### Unsupervised exploratory analysis of mtDNA deletion patterns separates samples by age and POLG genotype

The Bubble and Arc Plots show that deletion size, position, and frequency vary with age and disease state in diverse and complex ways. Therefore, in an attempt to uncover basic mechanisms of mtDNA deletion, we undertook unsupervised exploratory analyses to search for previously hidden patterns in the mtDNA deletion datasets (see Methods).

**Fig. 4:**
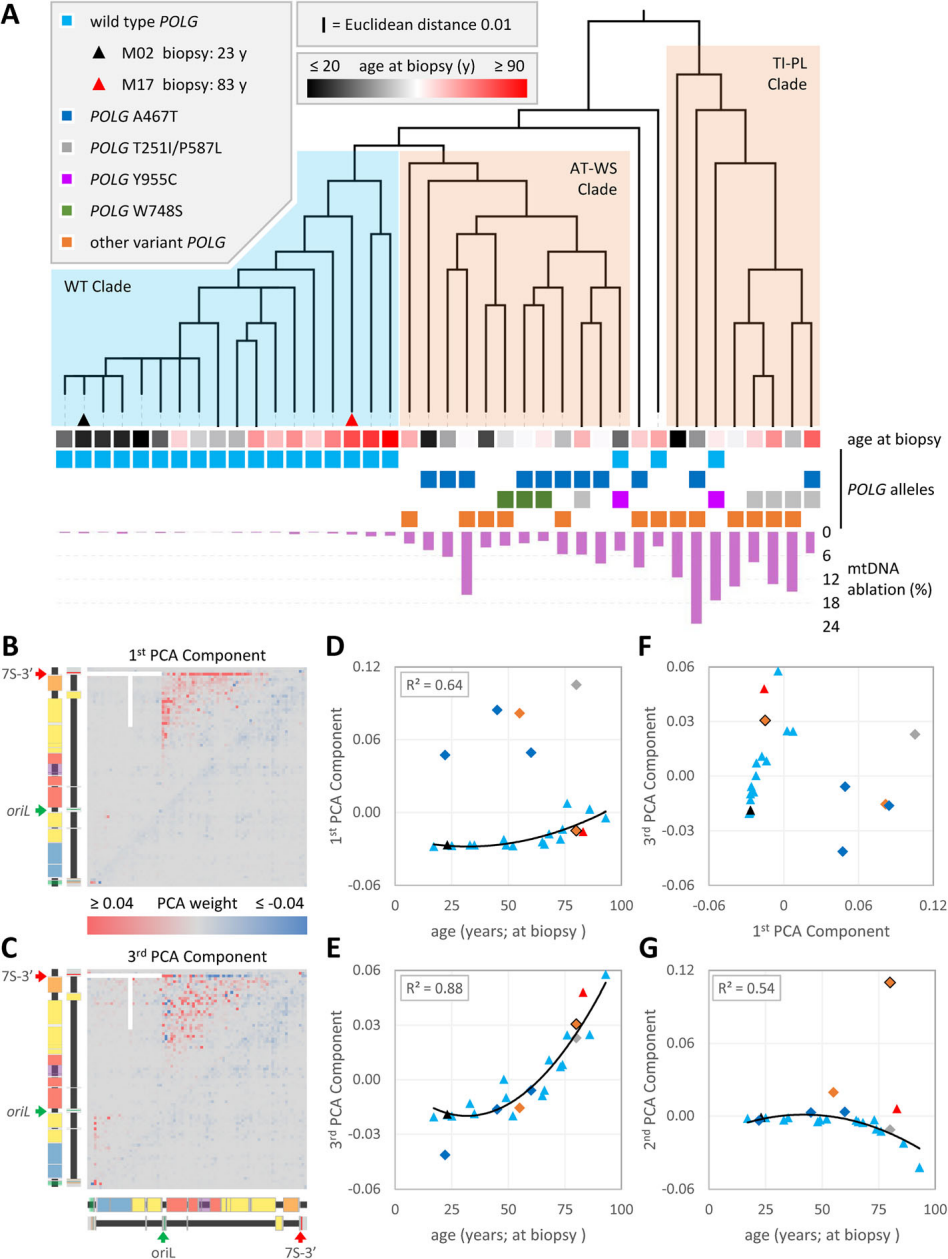
Unsupervised exploratory analysis of mtDNA deletion patterns. Hierarchical clustering revealed similarities between samples and principal component analysis (PCA) revealed the underlying patterns that explain the most variation between samples. Both analyses used the fraction of deletions, binned by terminus positions (80 × 80 bins), rather than deletion frequencies or mtDNA ablation levels. Deletions of < 40 bp and deletions mapped to the T-shaped white region (**b**, **c**) were excluded as possible false positives (see Methods). **a** Hierarchical clustering by Euclidean distance separates samples into three clades. Gwt samples form a WT clade and sort largely by age at biopsy (black to red; see Additional file 1: Table S1). Gvar samples are “paraphyletic,” i.e., some have patterns that are more like Gwts (AT-WS clade) than like other Gvars (TI-PL clade). Colored squares below the dendrogram indicate *POLG* genotypes, with ablation levels shown below. Similar Gvar genotypes tend to cluster. Purple bars below the dendrogram illustrate ablation levels. **b**, **c** A representative subset of Gvar samples was used for PCA (see the “Materials and methods” section): all Gwts; AT-WS and TI-PL clade representatives; and cladeless sample M24. **b** The 1st PCA component vector (deletion terminus positions as per Fig. 3b) colored by PCA weight (red to blue for ≥ 0.04 to ≤ − 0.04). **c** The 3rd PCA component weight vector. **d**–**g** Samples were scored using PCA weights. Black curves are parabolas fit to Gwt data (*R*^2^ inset). **d** The 1st component distinguishes Gwt (triangles) from Gvar deletion patterns, except M24 (orange diamond with black border). **e** 3rd component scores correlate with age at biopsy, regardless of *POLG* disease state. **f** These two components separate Gwt and Gvar clusters by deletion pattern (naïve to disease, age, and overall deletion frequency). **g** The 2nd PCA component was spent describing unique features of the disease state M24. In order to display minor arc features, the vectors in **b**, **c** were recalculated without M24

Hierarchical clustering of muscle samples by the Euclidean distance between their deletion patterns separates diseased samples from unafflicted. One Gwt (blue) and two Gvar (orange) clades were recovered (Fig. 4a). Wild type samples were monophyletic (WT clade) and arranged roughly by age. The youngest samples clustered tightly, indicating a high degree of similarity. For older samples, branch lengths and the branching order roughly followed increasing age, suggesting a gradual shift away from some underlying youthful pattern. Gvar samples are paraphyletic. Some have deletion patterns that are more like the WT clade than they are like other Gvars. One Gvar clade contains all three samples with a *POLG* W748S allele and eight of eleven samples with at least one A467T allele, including all three homozygous *POLG* A467T samples. This sister to the WT clade is dubbed the AT-WS clade. The other variant clade contains eight out of ten samples that have the 13.9 kbp terminal ridge/fountain major arc feature and four of five samples with a *POLG* T251I/P587L allele, and so is dubbed the TI-PL clade. Two Gvar samples have underlying deletion patterns so divergent that they lay outside these three clades. M24 is the only Gvar with extensive minor arc deletions, and M33 is the only Gvar with extensive TMH and neither *oriL* nor 7S-3′ deletion “fountains.” The only other Gvar sample lacking the *oriL* fountain is both the oldest and the most diverged member of the AT-WS clade (M34, 70 years at biopsy; L411P;R574Q).

Though the hierarchical clustering relied only upon normalized deletion fractions, the clades that it generated also differ by mtDNA ablation level and histology. The TI-PL clade has a significantly higher average ablation level (13.4 ± 4.5%; ± CL 95%; *n* = 8) than the AT-WS clade (5.6 ± 2.3%; *n* = 12; *p* = 0.0021, Student’s *t* test), which in turn has significantly higher ablation than the WT clade (0.37 ± 0.145%; *n* = 19; *p* = 0.00024). This trend is parallel with the ranking of COX-ve fractions (TI-PL at 18.6 ± 6.8% > AT-WS at 10.1 ± 4.1% > WT at 1.2 ± 1.5%; *p* = 0.017 and 0.00026, respectively). The deepest branching member of the TI-PL clade, sample M36, is exceptional by many measures, making its inclusion debatable. Within the TI-PL clade, M36 is the youngest sample (17 years versus 42 to 80), has the lowest fraction of deletions with TMH (51%), and has the lowest COX-ve fraction (3% versus 13–30%). Without M36, the COX-ve difference between the TI-PL and AT-WS clades is more significant (*p* = 0.0014).

### Deletion patterns have separable age- and disease-associated components

Principal component analysis (PCA) reduces the dimensionality of a data set and isolates independent trends (components) that explain the most variation within the data. Each component may be expressed as a weight vector (Fig. 4b, c) which results in a score when applied to an individual sample (Fig. 4d, e, and g). A higher component score suggests greater contribution of an underlying process to the final deletion pattern.

The primary (1st; Fig. 4b) and tertiary (3rd; Fig. 4c) principal components focus on deletions within the major arc. The 1st component accounts for the greatest variability among deletion patterns and neatly separates Gwt from Gvar patterns (Fig. 4d). Because 1st component scores are higher for Gvar samples (0.047 to 0.105, excluding M24) than for Gwt samples (− 0.028 to 0.007), we consider the 1st component vector to be the disease-like axis of the deletion pattern dataset. First component weights are maximal near *oriL* and the 7S-3′ (i.e., the most common deletion fountains) and decrease more or less smoothly with distance from those features (Fig. 4b). Among Gwt samples, 1st component scores increase slightly with age at biopsy (cohorts as in Fig. 4b). It is unclear what function should model this relationship, but an arbitrary parabolic fit explains the majority of score variation with age (*R*^2^ = 0.64). This sub-trend suggests the disease-like mechanism within the 1st component could be contributing to the accumulation of deletions in aging Gwt muscles. Also, these deletions have little TMH, implying much of the observed increase in low TMH deletions with age (Fig. 3a) may be influenced by the 1st component.

The 3rd component has positive weights for deletion termini scattered about the major arc with bins near origins mostly penalized (Fig. 4c). Third component scores generally increase with age at biopsy, regardless of *POLG* genotype (Fig. 4e). Because the oldest sample (Gwt M19, 93 years at biopsy) has the highest 3rd component score, we consider the 3rd component vector to be the aging-like axis of the deletion pattern dataset. An arbitrary parabolic fit explains most of the Gwt score variation with biopsy age (*R*^2^ = 0.88). For reasons unknown, parabolas fit to 1st and 3rd component scores have similar age minima (32.1 and 31.8 years at biopsy, respectively). More Gwt samples would help to determine the true functions underlying these curves, assuming that PCA scores remain stable in a larger dataset. Also, the deletions that dominate 3rd component scores have extensive TMH, so the observed increase in TMH-rich deletions with age (Fig. 3a) may be highly influenced by mechanisms underlying this 3rd component aging axis of the deletion dataset.

While 1st and 3rd component scores separate samples by Gvar state (Fig. 4f), Gvar sample M24 behaves like a Gwt sample along these axes. The 2nd component (Fig. 4g) is dedicated to the frequent minor arc deletions that distinguish M24 from all other samples (see Fig. 3b–d; note M24 exclusion from clades in Fig. 4a), which limits its explanatory power to that sample. Idiosyncratic components like this were the impetus for confining PCA to a subset of representative samples (see Methods).

### Deletion maps support a strand displacement mode of mtDNA replication

**Fig. 5:**
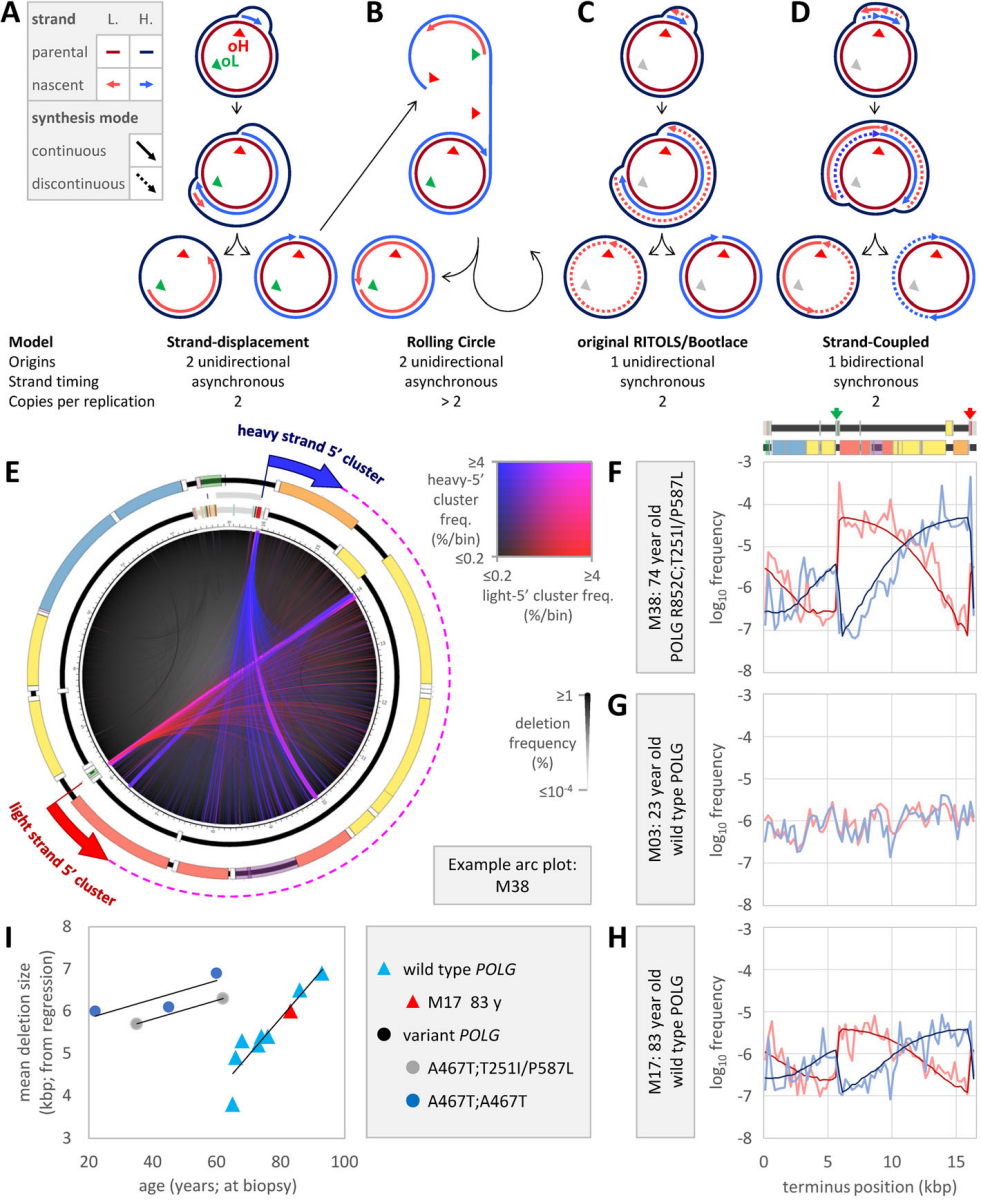
Deletion maps support a strand displacement mode of mtDNA replication. Deletion patterns were compared to four replication modes. **a**–**d** Heavy and light strands are shown in red and blue, with template strands darker. Arrows indicate 5′-to-3′ synthesis directions. Continuously and discontinuously replicated strands are shown as solid and dashed, respectively. Potential heavy and light strand replication origins (labeled oH and oL) are indicated by triangles (gray when unused). See the “Results” section for detailed descriptions of the **a** strand displacement mode, **b** the rolling circle mode proposed for *Caenorhabditis elegans* mtDNA, **c** a unidirectional, strand-synchronous mode as proposed for *Drosophila* mtDNA and older RITOLS and Bootlace modes, and **d** strand-coupled DNA replication. **e** Deletion arcs, scaled by individual deletion frequency and colored by terminus frequency in 100 bp bins, cluster near *oriL* and the 3′-terminus of 7S-DNA. See Supplemental Arc Plots for all Gvar samples. **f**–**h** Heavy- and light-strand-5′-terminus frequencies (lighter blue and red; 250 bp bins; excluding deletions with TMH ≥ 10 bp) reveal double-bowtie patterns in Gvar and aged Gwt samples (see 1st PCA component in Fig. 4b). Except in Gwt samples under 65 years of age, these patterns fit replication/deletion Monte Carlo models (darker curves; simulation 6 in Additional file 1: Fig. S6a). **f** Gvar example M38. **g** Younger Gwt example M03. **h** Older Gwt example M17. **i** Mean deletion lengths, from Monte Carlo regression, increased with age in Gwt (triangles) and Gvar (circles) samples

Several alternative modes of mtDNA replication have been proposed (reviewed in [49–51]). We focus here on four of them (Fig. 5a–d). Mode 1: in the strand displacement mode (SDM; Fig. 5a) [52, 53], nascent heavy-strand replication proceeds unidirectionally and continuously from an initiation site within the control region (oH in Fig. 5a–d). The displaced parental heavy strand is partially coated by mitochondrial ssDNA binding protein (mtSSB). After passage of the replication fork, priming occurs at the light-strand replication origin (oL in Fig. 5a–d; presumed to be *oriL* when assigned to a single site) and replication of the nascent light strand proceeds unidirectionally and continuously. Replication of each strand terminates at their respective origins. A subset of RITOLS (RNA incorporated throughout the lagging strand) and Bootlace modes ([54] and reviewed in [51]) resemble the SDM except that RNA transcripts assist/replace mtSSB and that the light strand is occasionally primed by a transcript distant from *oriL*. Mode 2: analogous to rolling circle replication proposed for *Caenorhabditis elegans* mtDNA [55], nascent heavy-strand replication may initiate as in the SDM but continues beyond the control region (oH in Fig. 5a–d). Light-strand synthesis initiates from *oriL* (shown here Fig. 5b) or other possible sites. Later steps resolve the expanding duplex into unit length circles (Fig. 5b). Mode 3: in unidirectional, strand synchronous replication, analogous to that proposed for *Drosophila* mtDNA [56], both strands initiate in the control region (oH in Fig. 5a–d). The nascent heavy strand is continuously replicated as a classical leading strand and the nascent light strand is discontinuously replicated as a classical lagging strand (Fig. 5c). A subset of RITOLS [57] resemble this mode more than the SDM mode. Mode 4: in bidirectional, strand-coupled DNA replication (SCD [58] and reviewed in [51]), mtDNA replication is similar to nuclear replication with classical leading and lagging strands and two replication forks per origin. Here, initiation is depicted as occurring in the control region, but some modes allow initiation anywhere in the major arc (i.e., *oriZ* [59]), oH is a replication termination site, and there is little or no role for a light-strand origin (i.e., *oriL*; Fig. 5d).

Deletion termini are not uniformly distributed in Gvar samples, as exemplified by sample M38 (Fig. 5e; 74 years at biopsy; *POLG* R852C;T251I/P587L). Excluding deletions with TMH over nine bp (associated with the 2nd PCA component), log-scale terminus frequencies resemble a double bowtie (Fig. 5f; 250 bp bins). One pair of peaks is bounded by the major arc. These peaks represent the recurrent deletion fountains and recapitulate the smooth falloff from origins in the 1st PCA component. The second pair of peaks is bounded by the minor arc. When viewed in this way, samples with significant quantities of deletion termini in the minor arc, such as M24, lie at one extreme in the spectrum of major and minor arc deletion frequencies. The double-bowtie pattern is absent from younger Gwt samples (e.g., M03, 23 years at biopsy, Fig. 5g) but present in older Gwt samples (e.g., M17, 83 years at biopsy, Fig. 5h). In fact, the nine oldest wild type samples (65 to 93 years) and one 48-year-old wild type sample (M07) all show the pattern, and these ten samples also have the highest wild type 1st PCA component scores.

The distribution of deletion termini for a variety of potential deletion mechanisms were modeled in silico. Monte Carlo models simulated the iterative generation of 240,000 deletions in arbitrarily clockwise or counterclockwise directions relative to the reference genome, and the impact of each deletion on selected genomic features was monitored. The 7S-3′ terminus and *oriL* were selected as critical genomic features based on the lack of ablation near replication origins (Fig. 2b). For replication modes that invoke specific, fixed origins, the 7S-3′ terminus and *oriL* were used in simulations because of their proximity to the sharp transitions of the double-bowtie pattern (Fig. 5f,h and Additional file 1: Fig. S6) and the interplay between replication and deletions implied by the marked amplification of the bowtie pattern in Gvar samples (Fig. 5f). Small pilot simulations were run for a wide variety of deletion generation schemata which were then winnowed down to six (Fig. 5a–d and Additional file 1: Fig. S6a). Each satisfied the following requirements: they must have some basis in proposed mechanisms of deletion generation, they must be able to produce something resembling a bowtie pattern, and they must use no more than five adjustable parameters in order to avoid overfitting. Two selected schemata were independent of mtDNA replication origins, as if deletions were triggered by attempted repair of random strand breaks or non-coding lesions without (sim. 1) or with (sim. 2) a dependence on deletion length. Four simulations (sims. 3–6) were based on recovery of replication intermediates generated during previously proposed replication modes. Two (sims. 3 and 4) assume formation of deletions during unidirectional H-strand replication from 7S-3′. One (sim. 3) models deletions initiated during continuous H-strand synthesis as in the original RITOLS/Bootlace synchronous synthesis mode or the first stage of asynchronous replication in either the rolling circle or strand displacement synthesis modes. Another (sim. 4) models deletions formed during original RITOLS/Bootlace DNA mode of synthesis without selection for *oriL*. The last two (sims. 5 and 6) assume deletions arise during synchronous bidirectional replication from 7S-3′ (i.e., strand-coupled synthesis) or during asynchronous unidirectional synthesis from 7S-3′ and *oriL* (i.e., the strand displacement model), respectively. In each iteration, deletion start points were randomly selected from a defined distribution, and endpoints were chosen from a second distribution. Critical genomic features were optionally selected for by removing deletions that impinged upon them from the population with an adjustable probability. The limit on adjustable parameters required that the same parameters be used to simulate deletions in both clockwise and counterclockwise directions.

Among the six Monte Carlo models of potential deletion mechanisms, sim. 6 fit the observed terminus frequencies better than all other models in both aged and diseased samples (Fig. 5h and f, respectively). Simulation 6, based on deletions made during asynchronous strand displacement replication (Fig. 5a), produced uniformly higher log-scale *R*^2^ values versus observed terminus frequencies for all test samples (mean 0.84, 0.70 and 0.88 for M38, M17 and M25, respectively; M38 and M17 for all simulations in Additional file 1: Fig. S6). The next best correlations were with simulation 3, which assumed no deletion initiation from *oriL* (mean *R*^2^ = 0.81, 0.58 and 0.82 for M38, M17 and M25, respectively). Simulation 6 correlation coefficients were lower for older wild type samples (over 65 years, mean *R*^2^ = 0.58; *n* = 8) than for Gvars (mean *R*^2^ = 0.78; *n* = 21; e.g., Additional file 1: Fig. S6). Samples exhibiting the worst fits for simulation 6 were the two Gvar samples that fell outside the AT-WS and TI-PL disease clades (M24 and M33; Fig. 4a) and the longest branch within the AT-WS clade (M34; Fig. 4a; excluding these three, Gvar mean *R*^2^ = 0.81). The mean deletion length, one of the parameters optimized in simulation 6, increased with age in both wild type and Gvar samples (Fig. 5i). Mean simulated deletion lengths are longer than the mean observed lengths, presumably because the simulations account for deletions that were selectively removed from the observed population. In younger Gwt samples, deletion terminus frequencies are explainable as independent termini selected from uniform distributions over the whole mtDNA. Such deletions may freely impinge upon *oriL* and the 7S-3′ terminus.

## Discussion

Mutations in nuclear genes encoding components of the mitochondrial replisome co-segregate with mitochondrial diseases [7], indicating that faulty mtDNA replication can cause a variety of heritable mitochondrial diseases [14]. Certain mitochondrial disease symptoms mimic those of natural aging, in particular those associated with skeletal muscle, and improper maintenance of the mitochondrial genome has been implicated as a major driver in the aging process [60]. Depletion of mtDNA or accumulation of point mutations and deletions in mtDNA directly leads to mitochondrial dysfunction. Depletion of mtDNA is linked to early-onset, often fatal mitochondrial diseases [61, 62]. However, skeletal muscle mtDNA levels vary little with age [34, 63, 64], and mtDNA copy number did not vary significantly, on average, between Gwt and Gvar samples in this study (see Additional file 1: Fig. S1a for representative values). Although mutations involving substitution of individual base pairs in mtDNA increase with age [65], they are insufficiently abundant to explain the phenotypes of aging [24, 25]. Accordingly, mtDNA deletions have been proposed to explain both mitochondrial disease and some aging phenotypes [30, 66–69].

### Ultrasensitive mtDNA deletion detection by LostArc

If mtDNA deletions are driven by replication, then mapping of mtDNA deletions should help to reveal the underlying causes of mitochondrial dysfunction and help distinguish between alternative modes of mtDNA replication (Fig. 5a–d). However, for purposes of sequencing library preparation, human skeletal muscle biopsies (Fig. 1b, step 1) yield mtDNA in very low quantities and with very low mtDNA to gDNA ratios (Fig. 1c). In pilot experiments, we, like others before us, found that every round of long-range PCR enrichment of mtDNA inflated the frequencies of random subsets of deletant mtDNA species. To avoid such artifacts, past high-throughput mapping attempts either focused on mtDNA deletions with population frequencies over 10^−3^ [70] or purified targeted deletions via digital drop PCR before library preparation [35]. The latter found 451 unique deletions in human brain tissue. Recently, one high-throughput study found a remarkable 4489 unique deletions, in the brain and blood, by accepting the flaws of long-range PCR enrichment and eschewing absolute frequency measurements [39]. LostArc achieves mtDNA enrichment without long-range PCR amplification by selectively degrading linear nuclear DNA with nucleases (Fig. 1b, steps 2–4b). Nextera tagmentation [71] allowed library preparation from the resulting nanogram level of DNA yields (step 5). Minimal post-tagmentation amplification is still necessary prior to Illumina paired-end sequencing (step 6). Methods for further reducing or eliminating amplification require further study.

The LostArc pipeline detects deletions by mapping split-reads to a circular mtDNA reference (step 7). Roughly 470,000 unique deletion spans were recovered from skeletal muscle biopsies collected from 19 Gwt individuals and 22 Gvar patients with mitochondrial diseases (Additional file 1: Table S1). Roughly 99% of these deletions have not been reported previously [35, 39, 47, 70, 72]. Deletion counts are converted to frequencies, the background subtracted, and then deletion patterns discovered (steps 8–10). The complex deletion patterns within individual samples depend upon both the sample age at biopsy and the *POLG* genotype.

### MtDNA deletions during aging

Deletion frequencies among Gwt samples do not vary significantly with age at biopsy (Fig. 2a), whereas mtDNA ablation levels increase non-linearly with age (Fig. 2c). Ablation levels refer to the loss of mtDNA segments through deletion, rather than the loss of entire mtDNA circles through depletion. Although deletions are not evenly distributed around the mtDNA circle in either wild type or Gvar cells, they rarely impinge upon *oriL* and the 3′-end of the 7S-DNA (7S-3′; Fig. 2b). These regions are putative light- and heavy-strand origins of replication, respectively, in two commonly proposed modes of mtDNA replication (Fig. 5a. b). If faulty mitochondria were selectively removed within these skeletal muscle samples—the prime function of mitophagy—then genomic features encoding fully functional OXPHOS machinery and its transcription and translation should also be preserved. OXPHOS is clearly impacted in both Gvar and older Gwt samples, as measured by the fraction of fibers lacking COX activity (Additional file 1: Table S1). Taken together, these observations have two implications: (1) retention of both *oriL* and the 7S-3′ is important for deletant species to be replicated frequently enough to reach the threshold for detection, which supports the hypothesis that they are initiation sites for strand displacement mtDNA replication, and (2) purifying selection against ablated circles though the process of mitophagy is insufficient to cleanse mtDNA pools, at least in the skeletal muscles of Gvar patients and older wild type individuals.

Gvar samples have higher ablation levels than wild type samples (Fig. 2d). Ablation increases with age in both homozygous *POLG* A467T and heterozygous *POLG* Y955C samples. If Gvar ablation levels are projected back to the age of disease onset, assuming a linear progression starting at zero ablation, then symptoms appear to become clinically relevant when ablation is as low as 1.3%, which exceeds the maximum ablation seen in older Gwt samples (Fig. 2d–e). Ablation of transcription, translation, and Complex IV machinery can explain roughly 75% of the variation in the fraction of fibers lacking cytochrome c oxidase activity (Additional file 1: Fig. S3), which is sufficient to explain some symptoms of age and disease in skeletal muscle. Additional work is needed to determine whether this holds true in brain and other organ systems that are affected by improper mtDNA maintenance.

### MtDNA deletion size, patterns, and spectra implicate the strand displacement mode of mtDNA replication

Size spectra and sequence context of deletions change with age and disease. Excluding deletions under 100 bp, the spectra of deletion sizes in young wild type muscle samples are essentially uniform (Fig. 2g). However, in Gvar and older Gwt samples, the apparent prohibition on deletions that impinge upon *oriL* and the 7S-3′ constrains deletion sizes (Fig. 2f, g) and positions (Fig. 3b–d) to zones within the minor and major arcs. Similarly, lengths of terminal microhomology for deletions in Gwt samples collected by 40 years of age are also uniformly distributed around the genome (Fig. 3a; Additional file 2: LostArc Reports). In contrast, Gvar samples (excluding M33) and older wild type samples have proportionally more deletions with little or no TMH. The frequency of deletions with over 10 bp of TMH increases with age in Gwt samples and is dependent upon *POLG* genotype among variant samples.

These complex dependencies divide deletions into three general classes with mechanistic implications. Class 1 deletions have low TMH, and formation of this type of deletion would be expected for ligation of DNA termini independent of base-base hydrogen bonding. Class 1 deletions increase in frequency of occurrence with advancing age or disease state (excluding M33). Class 2 deletions have high TMH, and frequent formation of this type of deletion would be expected for primer slippage during replication [32, 73, 74]. Class 2 deletions become more frequent with age (Fig. 3a), depending on *POLG* genotype (Additional file 2: LostArc Reports). Classes 1 and 2 largely preserve *oriL* and the 7S-3′ terminus (Fig. 2b, f, and g and Fig. 3b–d), which implicates asynchronous strand displacement DNA replication initiated from these potential origins (Fig. 5a, b) in the expansion and perhaps the creation of these deletions. Class 3 deletions do not respect these potential origins of replication, and they are uniformly distributed throughout the genome in young Gwt samples without regard for deletion size or TMH length. Class 3 deletions are expected to form by mechanisms independent of DNA replication [33, 75, 76]. Mitophagy and other phenotypic selectors for mitochondrial survival are not needed to explain these patterns.

Unsupervised analyses of deletion patterns confirm these classes quantitatively. A principal component analysis recovered two generally applicable components. The first PCA component (Fig. 4b) separates diseased from wild type samples but increases with age in the latter (Fig. 4d). This component is dominated by class 1 deletions. Scores for the third PCA component (Fig. 4c) increase with age, regardless of disease state (Fig. 4e). This component is dominated by class 2 deletions. Hierarchical clustering confirms that young wild type samples have very similar deletion patterns, but that patterns become more like those seen in diseased samples as age increases (Fig. 4a). This represents the age-dependent transition from class 3 to classes 1 and 2. Clustering of deletion patterns for similar *POLG* genotypes implies similar mechanisms of deletion formation. Gvar samples separated into two clades. The first clade contains all *POLG* W748S and most A467T alleles. The second, which contains most *POLG* T251I/P587L alleles, differs more from aged wild type patterns. PCA did not recover the difference underlying these divergent clades, likely due to the limited sample counts. This suggests a line of future inquiry as more samples become available for each genotype and for new allelic combinations.

Deletions that underlie the first PCA component, mostly of low-TMH class 1, have termini that cluster near potential origins (Fig. 4b; e.g., M38, Fig. 5e). Log-scale deletion terminus frequency maps (Fig. 5f–h; excluding TMH over 9 bp) vary by age and disease. Younger wild type samples again have a relatively uniform distribution of frequencies around the mtDNA circle (Fig. 5g). In Gvar and older wild type samples (Fig. 5f, h), light-strand-5′ termini peak to the right of potential origins (especially *oriL*) and heavy-strand-5′ termini peak to the left of potential origins (especially the 7S-3′). Because this double-bowtie pattern resembles diagrams of strand-biased mutagenesis and ribonucleotide incorporation that were used to define origin locations and replication strand identity in nuclear genomes (reviewed [77]), our analysis again strongly implicates mtDNA replication utilizing two origins in the creation and maintenance of mtDNA deletions.

Monte Carlo simulations were performed to test models for deletion formation (Additional file 1: Fig. S6a) that were either independent of DNA replication or dependent on competing modes of mtDNA replication (Fig. 5a–d). The model that best explained the observed frequencies of deletion termini required unidirectional formation of deletions independently proceeding from each potential origin, followed by selection against deletant species lacking either or both potential origins. The ability to accurately model observed deletion frequencies strongly implicates the strand displacement mode of mtDNA synthesis. A model invoking rolling circle replication (Fig. 5b) cannot be excluded; however, we discount this mode of replication in human muscle tissue as it has really only been suggested for nematodes and certain yeasts [55, 78]. Our data also support aspects of the RITOLS/Bootlace replication mechanism (Fig. 5c), although initiation of replication must be constrained to both origins and we cannot resolve whether the displaced DNA strand is coated with RNA [51].

Ultrasensitive deletion detection via the LostArc method yielded mtDNA deletion patterns that reveal the links between replication, aging, and mitochondrial disease. Deletion frequency does not correlate with age, but mtDNA ablation is sufficient to explain some symptoms of age and disease, and to approximate a clinical threshold. The patterns suggest little to no mitophagy in post-mitotic skeletal muscle fibers. Deletion sizes, locations, and sequence contexts change with age and disease, and the dependencies between them suggest three simultaneous mechanisms for deletion creation. One mechanism becomes less frequent with age and has features expected for replication-independent deletions, such as ligation of random strand breaks or interruption of DNA repair events. Incomplete degradation of damaged mtDNA [76] also presents opportunities to form mtDNA deletions with random lengths and positions. The other two mechanisms are synergistic, become more frequent with age, and have features that imply replication dependence. One of these mechanisms appears to be independent of base-base hydrogen bonding, like ligation of termini across large single-stranded gaps during replication. This suggests replication restart, whether by Pol γ or by another mitochondrially localized primase/polymerase, such as PrimPol [79, 80]. The other mechanism that increases with age resembles classical primer slippage during replication. Both primer slippage and replication restart could emerge from replication stalling, though why stalling would become more frequent with age is unclear. Myosatellite-mediated repair of muscular damage can introduce new mtDNA through formation of new fibers or fusion with damaged fibers [81–83]. Perhaps muscular trauma, such as that induced by exercise, is more frequent in younger muscles, leading to repeated dilution of the deletion-bearing population with fresh, myosatellite-derived populations. Differences in physical activity could then explain why some wild type samples look older (M07) or younger (M09) than their biopsy age. Regardless of the initiating event, the patterns underlying deletion frequencies strongly implicate the strand displacement mode of mtDNA synthesis.

The sensitivity of LostArc creates potential applications beyond basic research, such as genetic screening, diagnostic applications, and medical forensics. For instance, deletion patterns sort different *POLG* genotypes into divergent phylogenetic clades but separating the contributions of different *POLG* alleles will require deeper sampling of each genotype and new allelic combinations. Different components of the mitochondrial replisome may generate different patterns for mtDNA deletions. Availability of variant cell lines or tissue samples expressing disease alleles for Twinkle helicase (*TWNK*), the Pol γ accessory subunit (*POLG2*), or the mitochondrial single-stranded binding protein (*SSBP1*) would allow orthogonal testing of these ideas. While the current study strongly implicates faulty strand displacement replication as the causative agent of the muscular symptoms of aging and *POLG-*derived mitochondrial disease, a complete reckoning must await samples from other tissue types, from longitudinal collections from the same individual, from individuals with different environmental or pharmaceutical exposures or exercise regimes, and from patients with disparate disease phenotypes.

## Materials and methods

### Tissue samples and patient cohort

We studied diagnostic *vastus lateralis* (quadriceps) muscle biopsies from 22 patients, biopsied at 17 to 80 years of age, with genetically confirmed Gvars and evidence of multiple mtDNA deletions in muscle that are associated with focal cytochrome *c* oxidase (COX) deficiency (see Additional file 1: Tables S1 and S2 for information on clinical presentation and histopathological mitochondrial changes observed in the muscle). Quadriceps muscle biopsy samples from patients, aged 17 to 93 years with no evidence of clinical mitochondrial disease, were obtained either during anterior cruciate ligament (ACL) operations (*n* = 9; aged 17 to 52 years) or post-mortem (*n* = 9; aged 65 to 93 years), with one exception (below). This study was approved and performed under the ethical guidelines issued by the Newcastle and North Tyneside Local Research Ethics Committees (reference 09/H0906/75) and complied with the Declaration of Helsinki. One additional non-malignant muscle sample (M11; aged between 64 and 68 years) was obtained from the University of North Carolina Pathology during a left medial thigh mass biopsy (only normal tissue was used).

### Histopathological and molecular genetic studies

Cryostat sections (10 μm) were cut from transversely orientated muscle blocks and subjected to diagnostic histopathological protocols including COX, SDH, and sequential COX-SDH histochemistry [84]. The presence of mitochondrial DNA rearrangements was investigated using validated long-range PCR [85] and quantitative real-time assays [86]. The presence of pathogenic Gvars was confirmed either by candidate screening of all coding exons and intronic regions of the *POLG* gene (NM_002693), or the application of a massively parallel sequencing strategy using a custom, targeted Ampliseq gene panel covering 18 genes implicated in human disorders of mtDNA maintenance. Where necessary, all Gvars identified by next generation sequencing were confirmed by direct Sanger sequencing.

### Long-range PCR

Long-range PCR was carried out using human mitochondrial DNA primers targeting the 5′ end starting at bp position 10–40 and in the 3′ direction position 16,496–16,465 [87]. Phusion High-Fidelity DNA Polymerase (New England BioLabs, Inc., catalog # E0553S) was used to amplify the 16.5 kb amplicon per the manufacturer’s recommendations. Seventy-five nanograms of gDNA was used as the template in a 50-μl PCR reaction. Cycling conditions were as follows: hold cycle at 98 °C for 30 s, followed by 34 cycles consisting of 98 °C for 20 s and 70 °C for 6.5 min, and extended at 70 °C for 10 min, and upon completion held at 4 °C. Amplicons were run on a 0.8% agarose gel stained with ethidium bromide.

### Control plasmid generation

Human mitochondrial DNA was amplified using 75 ng DNA from HEK cell gDNA with New England Biolab’s Phusion polymerase (catalog # M0530S). The primers used were, in NC_012920 coordinates, mtDNA 9318F (cac tcc ata acg ctc ctc ata c) and mtDNA 13370R (cga ccc gga gca cat aaa tag). The amplification protocol was as follows: 98 °C hold for 30 s, followed by 30 cycles consisting of 98 °C for 20 s, 60 °C for 30 s, and 72 °C for 2.5 min, followed by extension at 72 °C for 10 min and holding at 4 °C. To confirm the appropriate size of the mtDNA fragments, the amplicons were run on a 0.8% agarose gel stained with ethidium bromide. The 4052 bp mtDNA amplicon was purified using Qiagen’s PCR clean up kit (catalog # 28104). The purified mtDNA fragment was A-tailed using Taq DNA polymerase and sub-cloned into TOPO pCR 2.1 vector (ThermoFisher Scientific TOPO-TA Cloning kit, catalog # 451641) per the manufacturer’s recommendations. Miniprep plasmid DNA was isolated from single colonies using Qiagen’s Miniprep kit (catalog # 27106). The plasmids were Sanger sequenced to verify the human mtDNA sequence.

A series of endonucleases were used to generate control plasmids with deletions. A sample of control plasmid DNA (4 μg) was cut with HpaI (New England Biolabs Inc., Catalog # R0105S) to generate a 2392 bp deletion between positions 10,014–12,405. A second deletion plasmid was generated using BseRI (New England Biolabs Inc., Catalog # R0581S), which cut at positions 9323 and 12,974 to generate a 3652 bp deletion. A third control plasmid generated by using PmeI and NdeI (New England Biolabs Inc., Catalog # R0560S and R0111S), which cut at positions 10,419 and 10,722, respectively (304 bp deletion). A Klenow (New England Biolabs Inc., Catalog # M0210S) reaction was used to fill the overhang generated from NdeI, and a blunt end ligation reaction was performed to join the ends. All of the remaining plasmid DNA was agarose gel purified and eluted with 100 μL of 1X T4 ligation buffer with dNTPs. Two microliters of T4 ligase (New England Biolabs Inc., Catalog # M0201S) was added and the reaction incubated at 16 °C overnight. The ligated plasmids were then transformed, miniprep DNA was generated and the sequence was verified by Sanger sequencing.

### Genomic DNA isolation

Genomic DNA was isolated (Fig. 1b) from either approximately 25 mg of frozen skeletal muscle tissue or 1 × 10^6^ cultured HEK cells using the Qiagen’s QiAmp kit (catalog # 51304). Frozen tissue samples were minced with a razor blade by hand on a disposable sterile surface. After adding 20 μl of proteinase K (600 mAU/mL solution or 40 mAU/mg protein), the tissue and cell lysates were incubated at 55 °C overnight. The remainder of the protocol followed Qiagen’s recommendations. The concentration of gDNA was determined using Invitrogen’s Qubit Fluorimeter.

In pilot experiments, immediately following genomic DNA isolation, technical replicate samples spiked with plasmid standards containing mtDNA sequence with artificial deletion junctions did not change the apparent frequencies of other deletant species (e.g., sample M22; Additional file 1: Fig. S2a, b, d, and e). Calculated control plasmid frequencies recapitulated input frequencies (*R*^2^ = 0.943, or 0.940 if the intercept is set to the origin) regardless of plasmid sizes (~ 4.2–7.5 kbp), deletion sizes (304–3652 bp), input concentrations (10^−5^–10^−1^), or the sample milieu (Additional file 1: Fig. S2c). Frequencies of individual deletions correlate well between DNA and tissue subsamples (Additional file 1: Fig. S2d and e; slopes and *R*^2^ values very near 1). Together, these demonstrate LostArc’s sensitivity, accuracy, and lack of bias.

### mtDNA enrichment

The following endonucleases were pooled in equal volumes, to final concentrations recommended by the manufacturer: AgeI, DraIII, FspI, PshAI, PspXI, SexAI, XmaI, and ExoV (New England Biolabs Inc.). The pooled endonucleases offered 1.77 × 10^6^ additional cuts to gDNA that provided more ends for the ExoV reaction that followed. Exonuclease digests were set up with 0.2 to 2.0 μg of human skeletal muscle gDNA, 10X NEB Buffer 4, 10 mM ATP, 4 μL of pooled endonucleases, and water to a final volume of 60 μL. Reactions were incubated at 37 °C for 1 h following the manufacturer’s protocol for ExoV reactions. Following heat inactivation for 30 min at 70 °C, a 60-μL ExoV only reaction without gDNA was added to the same tube and incubated for 24 h. ExoV treatment was for 72 h, with the daily addition of a complete ExoV reaction (60 μL) to avoid the accumulation of glycerol and salt that may inhibit in the reaction. Following the mtDNA enrichment, the reaction was again heat inactivated for 30 min at 70 °C. The reactions were column purified using Zymo DNA Clean and Concentrator kit (Zymo Research, catalog # D4013), per the manufacturer’s recommendation. Enriched mtDNA was eluted with ddH_2_0, and the concentration of the DNA was determined using Invitrogen’s Qubit Fluorimeter.

### Real-time PCR

Real-Time PCR was used to quantify the relative amount of mtDNA in each sample following mtDNA enrichment. Fluorescently labeled probes and primers targeting the nuclear gene *RPPH1* for normalization purposes and the mitochondrial target *MT-ND1* were purchased from ThermoFisher Scientific (catalog #‘s 4426961 and 4331182, respectively). Target amplicons were TA-cloned using Invitrogen’s TOPO TA-cloning kit (catalog # 451641), and then verified with Sanger sequencing. The cloned targets were used as positive controls, to generate standard curves to enable relative quantitation of mtDNA copy number of enriched samples. Real-Time PCR amplifications were carried out on an ABI PRISM 7900HT Sequence Detector (Applied Biosystems) with a cycling protocol consisting of 95 °C for 10 min, and 40 cycles at 95 °C for 15 s and 60 °C for 1 min. All reactions were done in triplicate, and 100 pg of enriched mitochondrial DNA was used in each reaction. Each 20 μL reaction used TaqMan 2X Universal Mix (Applied Biosystems, catalog # 4324018).

### Tagmentation

DNA libraries for next-generation sequencing were prepared with the Nextera XT Sample Preparation Kit (Illumina) using genomic DNA enriched for mitochondrial DNA. Each DNA sample was diluted to a final concentration of 1.0 ng/μL, and a total of 1 ng was used to prepare each library. Ten microliters of Tagment DNA Buffer were added to 1 ng of each DNA sample, and then 9 μL of Amplicon Tagment Mix was added and mixed thoroughly. The samples were incubated at 55 °C for 5 min and held at 10 °C. Neutralize Tagment Buffer (5 μL) was added and the samples were incubated at room temperature for 5 min to stop the tagmentation reaction. Fragmented DNA was amplified with limited-cycle PCR using a Nextera XT Index Kit. Fifteen microliters of Nextera PCR Master Mix were added to the neutralized samples, and then 5 μL of an index primer 1 and an index primer 2 was added and mixed thoroughly. The PCR was completed using the following cycling parameters: 72 °C for 3 min, 95 °C for 30 s, 12 cycles of 95 °C for 10 s, 55 °C for 30 s, 72 °C for 30 s, then a final extension of 72 °C for 5 min, and a hold at 10 °C (Illumina, Nextera XT DNA Library Preparation Guide, Rev. E ed., Illumina 2015).

### Library purification and sequencing

Small fragments were removed from the PCR reaction by incubating the sample for 2 min with 90 μL of Agencourt AMPure XP beads (Beckman Coulter) at room temperature. The bead mixture was incubated for 2 min on a magnetic stand to collect the beads. After supernatants were discarded, the beads were washed twice with 200 μL of fresh 80% ethanol. The beads were dried for 15 min at room temperature, resuspended in 52.5 μL of Resuspension Buffer (RSB) and allowed to incubate for 2 min to elute the DNA. Samples were placed on the magnetic stand until the supernatants were cleared. Cleared supernatants were transferred to a new microcentrifuge tube and the libraries were quantitated using the Qubit dsDNA High Sensitivity Kit (Invitrogen). Fragment sizes were determined by Agilent’s 2100 Bioanalyzer. Libraries were sequenced on an Illumina NextSeq with 2 × 150 bp paired-end reads.

### Sequence preparation

Reads were filtered by quality score prior to alignment and reads with average Phred scores less than 20 were excluded from further analysis. Cutadapt [88] was used to trim adapter sequences from reads.

### Deletion detection

The ROTLA package (for Reader of the Lost Arcs) uses a split-read approach for detecting deletions (reviewed in [89]). To detect a deletion, it searches for reads whose ends map to two reference locations separated by more than one read length. The mtDNA reference was ChrM from Genome Reference Consortium Human Build 37 (i.e., hg19, African (Yoruba) Sequence; GenBank AF347015.1). LostArc aligns sequencing reads using BLAT, an algorithm designed to quickly align expressed sequence tags [90]. The small size of the mitochondrial genome allows a BLAT alignment to be performed efficiently, even when aligning many reads. BLAT was not designed for alignment to a circular sequence. To simulate a circular genome, reads were aligned to two copies of the mitochondrial genome arranged in tandem. When parsing alignments, ROTLA will adjust sequence indices consistent with circular alignment. To increase confidence in detected deletions, ROTLA will compare across reads in mate pairs and will only report deletions consistent across paired reads. To facilitate comparison, ROTLA will first left-align all detected breaks. Breaks are then evaluated considering each read alignment. If there is a conflict between two reads, meaning a break is reported in only one read though both reads span the junction of the break, then the break is not reported. ROTLA reports raw counts for each detected deletion. ROTLA makes no attempt to filter or curate the deletion list. We recommend comparison against control samples (such as the cultured HEK cells in the current work) for deletion validation.

The deletion load for each sample is calculable as the total number of reads across deletion junctions divided by the total read count (Additional file 1: Table S1).

### Calculating deletion frequencies from read counts

The ROTLA count of reads supporting each deletion must be converted into deletion frequencies, accounting for differing molecular sizes and the mappability of each locus. This study focuses on mtDNA deletions that are not contained within repeat tracts, and most of our analyses are confined to deletions longer than 10 base pairs. We make a simplifying assumption that each starting DNA molecule has no more than one deletion larger than a few base pairs, and this assumption appears to hold true for most samples. However, the following calculations are approximations for samples with very high deletion frequencies, such as samples with obvious germline or early development deletions (M15, M35, and M36) or samples with very high cumulative deletion rates (e.g., M24).

We further assume that sequencing reads originate from each position within each DNA molecule in the starting sample with equal probability, after correcting for context effects. In other words, sequencing depth is proportional to population fraction. Let ***f***_***i***_ represent the frequency of a particular mtDNA molecular species or set of species, expressed as a fraction of the total mtDNA population. The species may bear a particular deletion (*i*), have no deletions (*i* = *u*), or represent all species present in the sample (*i* = *t*). If ***d***_***i***_ is the average depth of reads due to species *i*, then.

1 $$ {\boldsymbol{f}}_{\boldsymbol{i}}=\frac{{\boldsymbol{d}}_{\boldsymbol{i}}}{\sum_{\boldsymbol{i}}{\boldsymbol{d}}_{\boldsymbol{i}}}. $$

The average depth may be expressed in a generalized form,

2 $$ {\boldsymbol{d}}_{\boldsymbol{i}}=\frac{{\boldsymbol{L}}_{\boldsymbol{r}}{\boldsymbol{r}}_{\boldsymbol{i}}}{{\boldsymbol{L}}_{\boldsymbol{i}}}, $$

and in a form that is specific to molecules containing deletions,

3 $$ {\boldsymbol{d}}_{\boldsymbol{i}}={\boldsymbol{\delta}}_{\boldsymbol{i}}{\boldsymbol{x}}_{\boldsymbol{i}}. $$

Here *r*_*i*_ is the effective number of reads mapped to species *i*, *L*_*i*_ is the length of species *i*, *L*_*r*_ is the read length, here taken to be the average read length for the sample of interest, *x*_*i*_ is the number of reads spanning the breakpoint junction of deletion *i*, and *δ*_*i*_ is the mappability factor for reads with breakpoint *i*. The mappability factor is calculated from the HEK control sample and is simply the median depth divided by the mean depth at the two positions that define the breakpoint junction for deletion *i*.

For molecules without deletions, the generalized form of the average depth is applicable from Eq. 2,

4 $$ {\boldsymbol{d}}_{\boldsymbol{u}}=\frac{{\boldsymbol{L}}_{\boldsymbol{r}}{\boldsymbol{r}}_{\boldsymbol{u}}}{{\boldsymbol{L}}_{\boldsymbol{u}}}. $$

Setting Eqs. 2 and 3 equal to one another and rearranging yields

5 $$ {\boldsymbol{r}}_{\boldsymbol{i}}=\frac{{\boldsymbol{L}}_{\boldsymbol{i}}{\boldsymbol{\delta}}_{\boldsymbol{i}}{\boldsymbol{x}}_{\boldsymbol{i}}}{{\boldsymbol{L}}_{\boldsymbol{r}}}. $$

The total number of mapped reads must equal the sum of the reads mapped to all species, thus

6 $$ {\boldsymbol{r}}_{\boldsymbol{t}}={\boldsymbol{r}}_{\boldsymbol{u}}+{\sum}_{\boldsymbol{i}}{\boldsymbol{r}}_{\boldsymbol{i}} $$

and therefore, after isolating the component due to species *u* and rearranging,

7 $$ {\boldsymbol{r}}_{\boldsymbol{u}}={\boldsymbol{r}}_{\boldsymbol{t}}-{\sum}_{\boldsymbol{i}}\frac{{\boldsymbol{L}}_{\boldsymbol{i}}{\boldsymbol{\delta}}_{\boldsymbol{i}}{\boldsymbol{x}}_{\boldsymbol{i}}}{{\boldsymbol{L}}_{\boldsymbol{r}}}. $$

Taking Eq. 1 and isolating the denominator component due to species *u* yields

8 $$ {\boldsymbol{f}}_{\boldsymbol{i}}=\frac{{\boldsymbol{d}}_{\boldsymbol{i}}}{{\boldsymbol{d}}_{\boldsymbol{u}}+{\sum}_{\boldsymbol{i}}{\boldsymbol{d}}_{\boldsymbol{i}}}. $$

Finally, sequential substitutions of Eqs. 3, 4, and then 7 into Eq. 8 yield

9 $$ {\boldsymbol{f}}_{\boldsymbol{i}}=\frac{{\boldsymbol{\delta}}_{\boldsymbol{i}}{\boldsymbol{x}}_{\boldsymbol{i}}}{{\boldsymbol{d}}_{\boldsymbol{u}}+{\sum}_{\boldsymbol{i}}{\boldsymbol{\delta}}_{\boldsymbol{i}}{\boldsymbol{x}}_{\boldsymbol{i}}}=\frac{{\boldsymbol{\delta}}_{\boldsymbol{i}}{\boldsymbol{x}}_{\boldsymbol{i}}}{\frac{{\boldsymbol{L}}_{\boldsymbol{r}}{\boldsymbol{r}}_{\boldsymbol{u}}}{{\boldsymbol{L}}_{\boldsymbol{u}}}+{\sum}_{\boldsymbol{i}}{\boldsymbol{\delta}}_{\boldsymbol{i}}{\boldsymbol{x}}_{\boldsymbol{i}}}=\frac{{\boldsymbol{\delta}}_{\boldsymbol{i}}{\boldsymbol{x}}_{\boldsymbol{i}}}{\frac{{\boldsymbol{L}}_{\boldsymbol{r}}}{{\boldsymbol{L}}_{\boldsymbol{u}}}\left({\boldsymbol{r}}_{\boldsymbol{t}}-{\sum}_{\boldsymbol{i}}\frac{{\boldsymbol{L}}_{\boldsymbol{i}}{\boldsymbol{\delta}}_{\boldsymbol{i}}{\boldsymbol{x}}_{\boldsymbol{i}}}{{\boldsymbol{L}}_{\boldsymbol{r}}}\right)+{\sum}_{\boldsymbol{i}}{\boldsymbol{\delta}}_{\boldsymbol{i}}{\boldsymbol{x}}_{\boldsymbol{i}}}=\frac{{\boldsymbol{L}}_{\boldsymbol{u}}{\boldsymbol{\delta}}_{\boldsymbol{i}}{\boldsymbol{x}}_{\boldsymbol{i}}}{{\boldsymbol{L}}_{\boldsymbol{r}}{\boldsymbol{r}}_{\boldsymbol{t}}-{\sum}_{\boldsymbol{i}}{\boldsymbol{L}}_{\boldsymbol{i}}{\boldsymbol{\delta}}_{\boldsymbol{i}}{\boldsymbol{x}}_{\boldsymbol{i}}+{\sum}_{\boldsymbol{i}}{\boldsymbol{L}}_{\boldsymbol{u}}{\boldsymbol{\delta}}_{\boldsymbol{i}}{\boldsymbol{x}}_{\boldsymbol{i}}}. $$

This is the formula used to calculate deletion frequency in this study. However, an approximation may be useful when complete information on all species is lacking or when full computation is difficult. The last two denominator terms in Eq. 9 are related by

10 $$ {\sum}_i{L}_i{\delta}_i{x}_i\lesssim {\sum}_i{L}_u{\delta}_i{x}_i $$

because deletant species are shorter than the full-length reference. Thus,

11 $$ {f}_i\lesssim \frac{L_u{\delta}_i{x}_i}{L_r{r}_t} $$

is the upper bound approximation of the fraction of the population for any given deletant species.

Deletion frequencies above 10^−6^ correlate well between subsamples of M22 (Additional file 1: Fig. S2d and e; linear correlation between bins, *R*^2^ > 0.99). Above this boundary lie very rare deletions, for which individual rates are confidently determined. Below this boundary lie ultra-rare deletions, for which collective rates are accurate but individual rates are subject to stochastic variation.

### Estimating the boundary frequencies for multi-fiber deletion species

Given skeletal muscle density of about 1.06 mg/μL [91, 92] and muscle samples of 20–25 mg, sample volumes should average 18.9–23.6 μL. Initial muscle biopsies were approximately cylindrical, but sub-sampling resulted in less regular shapes. As a simplification, these volumes imply cubes of about 2.66–2.87 mm in width with minimum cross-sectional areas of 7.09–8.22 mm^2^. Dividing by the mean quadriceps fiber cross-sectional area of 3630 μm^2^ [93] indicates there are around 1950–2270 truncated muscle fibers per sample. Taking the reciprocal suggests any deletion that exceeds about 5.12 × 10^−4^ of the population must come from multiple fibers. Knowing the total mass of DNA purified from each sample and the relative ratios of nuclear and mtDNA determined by qPCR (Fig. 1c and Additional file 1: Fig. S1) indicates the average number of nuclei per initial Gwt muscle sample is around 380,100. This means there would be an average of 168–195 nuclei per truncated fiber, or about 66 nuclei per mm of fiber, which is similar to the density of nuclei determined with microscopy measurements [94] in which only 1–4% are likely myosatellite nuclei [95, 96]. Similarly, about 1.08 × 10^9^ mtDNA circles are present in each initial Gwt muscle sample, suggesting that one unique deletion in one mtDNA circle would occur at a frequency of about 9.22 × 10^−10^ in a given sample. Also, there would be about 479,000–556,000 mtDNA per truncated fiber. Combining values yields about 2850 mtDNA per nucleus, which essentially matches past measurements of mtDNA copy number in skeletal muscle determined by shotgun sequencing (2788) or digital drop PCR (2744) [63].

### Calculating ablation levels

The ablation level at biopsy (***a***_***biopsy***_) is defined as the amount of mtDNA lost from existing circles due to deletion. It is calculated from deletion frequencies (***f***_***i***_) and fractional deletion lengths (***L***_***u***_ ***− L***_***i***_)/***L***_***u***_:

12 $$ {\boldsymbol{a}}_{\boldsymbol{biopsy}}={\sum}_{\boldsymbol{i}}{\boldsymbol{f}}_{\boldsymbol{i}}\left({\boldsymbol{L}}_{\boldsymbol{u}}-{\boldsymbol{L}}_{\boldsymbol{i}}\right)/{\boldsymbol{L}}_{\boldsymbol{u}}. $$

This is usually expressed as a percentage and is equivalent to the area above curves such as those in Fig. 2b.

The ablation level at onset (***a***_***onset***_, Fig. 2e) is approximated by projecting a line from the ablation level at biopsy age (***y***_***biopsy***_) toward the origin through the age of disease onset (***y***_***onset***_):

13 $$ {\boldsymbol{a}}_{\boldsymbol{onset}}=\frac{{\boldsymbol{a}}_{\boldsymbol{biopsy}}{\boldsymbol{y}}_{\boldsymbol{onset}}}{{\boldsymbol{y}}_{\boldsymbol{biopsy}}}. $$

Note that this is a first approximation. Better approximations will require samples of each *POLG* genotype at many more ages, some of which will be very difficult to acquire due to patient youth and genotypic rarity.

### Visualizing lost arcs

Arc plots (examples in Fig. 3c, d; all samples in Additional file 3: Arc Maps) were created in Circos [97].

### Hierarchical clustering of deletion patterns

For exploratory analysis, breakpoint rates for each sample were first binned by breakpoint position by dividing the mitochondrial genome into 80 equally sized bins. Breakpoints were then binned into a two-dimensional array (80 × 80 bins) based on binned start and end positions. Due to higher sequencing error rates, the veracity of individual small deletions was suspect, so breakpoints were excluded if they were between two positions within 40 bps on the genome. Pilot experiments with commercial DNA had age-independent breakpoint peaks in a T-shaped region of these plots (deletions from between positions 1 and 5700 to between 15,949 and 16,156; deletions from between 3107 and 3314 to between 12,000 and 16,156) that were not seen in Gwt muscle samples collected before 80 years of age. Breakpoints within this region were excluded because we suspected such breaks were caused by commercial DNA extraction protocols. After binning, breakpoint counts were normalized by the total number of binned breakpoints for each sample.

### Principal component analysis of deletion patterns

After samples were clustered into clades by similarity, a representative subset of samples was subjected to principal component analysis (PCA) to find the underlying patterns that explained the most variation between samples. Binned arrays for each sample were flattened into one-dimensional vectors, and these vectors were applied to principal component analysis (PCA) and hierarchical clustering. Both were performed using the Python package scikit-learn [98]. Hierarchical clustering was performed using the complete linkage method considering the Euclidean distance between vectors. Three-dimensional scatterplots of PCA components and dendrograms capturing cluster results were generated using the Python package plotly (Plotly Technologies Inc. Collaborative data science. Montréal, QC, 2015; https://plot.ly).

Initial attempts to identify the principal components affecting variation of deletion patterns between samples included all samples and proved fraught with noise from excessive variance. When balancing breadth of discovery against the potential for overparameterization, we realized that the most explanatory PCA components are consumed describing the idiosyncrasies of unique Gvar samples with unique deletion features. Accordingly, we limited our analysis to the first three principal components and focused on a select subset of samples to identify shared underlying deletion patterns. We included all Gwt samples, the three samples comprising the best Gvar time series (homozygous *POLG* A467T), one Gvar from outside the major clades (M24 *POLG* A467T;S933R), and one representative from each major Gvar clade (Fig. 4a). Samples M22 (*POLG* R1096C;W748S) from the AT-WS clade and M25 (*POLG* A467T;T251I/P587L) from the TI-PL clade were selected because their deletion patterns broadly represent their clades and because a small number of deletions (780 deletions with 623 junctions) previously reported for each sample [32] presented an opportunity for discovery due to the sensitivity and depth of LostArc (3.3 million deletions with 87,071 junctions).

### Monte Carlo simulations of replication/deletion processes

Deletions in Monte Carlo models are simulated by randomly selecting a deletion start point from a defined distribution and then randomly selecting an end site from another defined distribution. A large number of distribution combinations were tested in smaller pilot simulations, from which six, representing potential deletion mechanisms and recapitulating at least some of the features of the observations, were selected for full simulation (Additional file 1: Fig. S6). Simulations were regressed by minimizing the log domain least squares difference between simulated and observed deletion terminus density (e.g., Fig. 5f, h and Additional file 1: Fig. S6). In order to avoid overfitting, no model was allowed more than five adjustable parameters. Thus, deletions modeled in both clockwise and counterclockwise directions were forced to share parameters rather than varying independently. All models included a linear scaling parameter and a selection coefficient. The latter is a value between zero and one. Simulated deletions that impinge upon an origin position are assigned a uniformly distributed random value in that range. If the random value exceeds the coefficient, the deletion is removed from the simulated population. A selection process like the one that this coefficient simulates is implied by the positional ablation levels in Fig. 2b. All six models here select against 7S-DNA 3′-end (7S-3′) deletant species and four also select against *oriL* deletants. Simulated deletions shorter than 1 bp (possible in models 2–6), or that exceed the mtDNA reference size (16,571 bp; models 2–6), or that impinge upon the origin from whence they originated (models 3–6), are also removed from the population. It is possible that the latter should be selected against rather than removed, but the requisite selection coefficient would cause most models to exceed the limit of five tunable parameters. In addition to the scaling and selection parameters, a normal distribution adds mean and standard deviation parameters and an exponential distribution adds a negative shape parameter. Two hundred and forty thousand deletions were simulated in each direction for each model, meaning 240,000 total simulations each for models 3 and 4 and 480,000 simulations for each other model.

## Supplementary information

**Additional file 1:** Results. **Figure S1.** Mitochondrial DNA content during the LostArc procedure. **Figure S2.** Internal controls for validation of the LostArc method. **Figure S3.** The COX-ve count is poorly explained by deletions in Complex IV genes alone. **Figure S4.** LostArc Report example #1: weighted mean of three HEK samples. **Figure S5.** LostArc Report example #2: young Gwt sample M01. **Figure S6.** Example fits to alternative replication/deletion models. **Table S1.** Sample list and deletion mapping statistics. **Table S2.** Patient symptoms and references associated with *POLG* variant samples [99–104].

**Additional file 2.** LostArc Reports.

**Additional file 3.** Arc Maps.

**Additional file 4.** Review history.

## Data Availability

ROTLA was written in Python and is licensed under the MIT License. ROTLA is open source and is freely available on GitHub (https://github.com/NIEHS/ROTLA) [105] and through Zenodo, DOI: 10.5281/zenodo.3966359 [106]. Sequence files and deletion calls may be found at dbGaP (accession number phs002052.v1.p1) [107].

## References

[1] Payne BA (2013). Universal heteroplasmy of human mitochondrial DNA. Hum Mol Genet.

[2] Wallace DC, Chalkia D (2013). Mitochondrial DNA genetics and the heteroplasmy conundrum in evolution and disease. Cold Spring Harb Perspect Biol.

[3] Lightowlers RN, Taylor RW, Turnbull DM (2015). Mutations causing mitochondrial disease: what is new and what challenges remain?. Science.

[4] Gorman GS (2016). Mitochondrial diseases. Nat Rev Dis Primers.

[5] Chinnery PF (2002). Modulating heteroplasmy. Trends Genet.

[6] Craven L, Alston CL, Taylor RW, Turnbull DM (2017). Recent advances in mitochondrial disease. Annu Rev Genomics Hum Genet.

[7] Viscomi C, Zeviani M (2017). MtDNA-maintenance defects: syndromes and genes. J Inherit Metab Dis.

[8] Nicholls TJ (2018). Topoisomerase 3alpha is required for decatenation and segregation of human mtDNA. Mol Cell.

[9] Gustafson MA (2019). Mitochondrial single-stranded DNA binding protein novel de novo SSBP1 mutation in a child with single large-scale mtDNA deletion (SLSMD) clinically manifesting as Pearson, Kearns-Sayre, and Leigh syndromes. PLoS One.

[10] Del Dotto V (2020). SSBP1 mutations cause mtDNA depletion underlying a complex optic atrophy disorder. J Clin Invest.

[11] Kaguni LS (2004). DNA polymerase gamma, the mitochondrial replicase. Annu Rev Biochem.

[12] Graziewicz MA, Longley MJ, Copeland WC (2006). DNA polymerase gamma in mitochondrial DNA replication and repair. Chem Rev.

[13] Korhonen JA, Pham XH, Pellegrini M, Falkenberg M (2004). Reconstitution of a minimal mtDNA replisome in vitro. EMBO J.

[14] Young MJ, Copeland WC (2016). Human mitochondrial DNA replication machinery and disease. Curr Opin Genet Dev.

[15] Chinnery PF, Zeviani M (2008). 155th ENMC workshop: polymerase gamma and disorders of mitochondrial DNA synthesis, 21-23 September 2007, Naarden, The Netherlands. Neuromuscul Disord.

[16] Copeland WC (2008). Inherited mitochondrial diseases of DNA replication. Annu Rev Med.

[17] Rahman S, Copeland WC (2019). POLG-related disorders and their neurological manifestations. Nat Rev Neurol.

[18] Linnane AW, Marzuki S, Ozawa T, Tanaka M (1989). Mitochondrial DNA mutations as an important contributor to ageing and degenerative diseases. Lancet.

[19] Cortopassi GA, Arnheim N (1990). Detection of a specific mitochondrial DNA deletion in tissues of older humans. Nucleic Acids Res.

[20] Cortopassi GA, Shibata D, Soong NW, Arnheim N (1992). A pattern of accumulation of a somatic deletion of mitochondrial DNA in aging human tissues. Proc Natl Acad Sci U S A.

[21] Bua E (2006). Mitochondrial DNA-deletion mutations accumulate intracellularly to detrimental levels in aged human skeletal muscle fibers. Am J Hum Genet.

[22] Bender A (2006). High levels of mitochondrial DNA deletions in substantia nigra neurons in aging and Parkinson disease. Nat Genet.

[23] Vincent AE (2018). Subcellular origin of mitochondrial DNA deletions in human skeletal muscle. Ann Neurol.

[24] Kennedy SR, Salk JJ, Schmitt MW, Loeb LA (2013). Ultra-sensitive sequencing reveals an age-related increase in somatic mitochondrial mutations that are inconsistent with oxidative damage. PLoS Genet.

[25] Vermulst M (2007). Mitochondrial point mutations do not limit the natural lifespan of mice. Nat Genet.

[26] Longley MJ, Nguyen D, Kunkel TA, Copeland WC (2001). The fidelity of human DNA polymerase gamma with and without exonucleolytic proofreading and the p55 accessory subunit. J Biol Chem.

[27] Stumpf JD, Copeland WC (2013). The exonuclease activity of the yeast mitochondrial DNA polymerase gamma suppresses mitochondrial DNA deletions between short direct repeats in Saccharomyces cerevisiae. Genetics.

[28] Trifunovic A (2004). Premature ageing in mice expressing defective mitochondrial DNA polymerase. Nature.

[29] Kujoth GC (2005). Mitochondrial DNA mutations, oxidative stress, and apoptosis in mammalian aging. Science.

[30] Vermulst M (2008). DNA deletions and clonal mutations drive premature aging in mitochondrial mutator mice. Nat Genet.

[31] Shoffner JM (1989). Spontaneous Kearns-Sayre/chronic external ophthalmoplegia plus syndrome associated with a mitochondrial DNA deletion: a slip-replication model and metabolic therapy. Proc Natl Acad Sci U S A.

[32] Persson O (2019). Copy-choice recombination during mitochondrial L-strand synthesis causes DNA deletions. Nat Commun.

[33] Krishnan KJ (2008). What causes mitochondrial DNA deletions in human cells?. Nat Genet.

[34] Miller FJ, Rosenfeldt FL, Zhang C, Linnane AW, Nagley P (2003). Precise determination of mitochondrial DNA copy number in human skeletal and cardiac muscle by a PCR-based assay: lack of change of copy number with age. Nucleic Acids Res.

[35] Taylor SD (2014). Targeted enrichment and high-resolution digital profiling of mitochondrial DNA deletions in human brain. Aging Cell.

[36] Fokkema IF (2011). LOVD v.2.0: the next generation in gene variant databases. Hum Mutat.

[37] Landrum MJ (2018). ClinVar: improving access to variant interpretations and supporting evidence. Nucleic Acids Res.

[38] Longley MJ, Graziewicz MA, Bienstock RJ, Copeland WC (2005). Consequences of mutations in human DNA polymerase gamma. Gene.

[39] Hjelm BE (2019). Splice-Break: exploiting an RNA-seq splice junction algorithm to discover mitochondrial DNA deletion breakpoints and analyses of psychiatric disorders. Nucleic Acids Res.

[40] Phillips NR, Sprouse ML, Roby RK (2014). Simultaneous quantification of mitochondrial DNA copy number and deletion ratio: a multiplex real-time PCR assay. Sci Rep.

[41] Wanagat J, Ahmadieh N, Bielas JH, Ericson NG, Van Remmen H (2015). Skeletal muscle mitochondrial DNA deletions are not increased in CuZn-superoxide dismutase deficient mice. Exp Gerontol.

[42] Hood DA, Memme JM, Oliveira AN, Triolo M (2019). Maintenance of skeletal muscle mitochondria in health, exercise, and aging. Annu Rev Physiol.

[43] Schon EA, Gilkerson RW (2010). Functional complementation of mitochondrial DNAs: mobilizing mitochondrial genetics against dysfunction. Biochim Biophys Acta.

[44] Sato A, Nakada K, Hayashi J (2009). Mitochondrial complementation preventing respiratory dysfunction caused by mutant mtDNA. Biofactors.

[45] Yoneda M, Miyatake T, Attardi G (1994). Complementation of mutant and wild-type human mitochondrial DNAs coexisting since the mutation event and lack of complementation of DNAs introduced separately into a cell within distinct organelles. Mol Cell Biol.

[46] Burkholder AB (2018). Muver, a computational framework for accurately calling accumulated mutations. BMC Genomics.

[47] Lott MT (2013). mtDNA variation and analysis using Mitomap and Mitomaster. Curr Protoc Bioinformatics.

[48] Schon EA (1989). A direct repeat is a hotspot for large-scale deletion of human mitochondrial-DNA. Science.

[49] McKinney EA, Oliveira MT (2013). Replicating animal mitochondrial DNA. Genet Mol Biol.

[50] Ciesielski GL, Oliveira MT, Kaguni LS (2016). Animal mitochondrial DNA replication. Enzymes.

[51] Yasukawa T, Kang D (2018). An overview of mammalian mitochondrial DNA replication mechanisms. J Biochem.

[52] Robberson DL, Clayton DA (1972). Replication of mitochondrial DNA in mouse L cells and their thymidine kinase - derivatives: displacement replication on a covalently-closed circular template. Proc Natl Acad Sci U S A.

[53] Brown TA, Cecconi C, Tkachuk AN, Bustamante C, Clayton DA (2005). Replication of mitochondrial DNA occurs by strand displacement with alternative light-strand origins, not via a strand-coupled mechanism. Genes Dev.

[54] Reyes A (2013). Mitochondrial DNA replication proceeds via a ‘bootlace’ mechanism involving the incorporation of processed transcripts. Nucleic Acids Res.

[55] Lewis SC (2015). A rolling circle replication mechanism produces multimeric lariats of mitochondrial DNA in Caenorhabditis elegans. PLoS Genet.

[56] Joers P, Jacobs HT (2013). Analysis of replication intermediates indicates that Drosophila melanogaster mitochondrial DNA replicates by a strand-coupled theta mechanism. PLoS One.

[57] Pohjoismaki JL (2010). Mammalian mitochondrial DNA replication intermediates are essentially duplex but contain extensive tracts of RNA/DNA hybrid. J Mol Biol.

[58] Holt IJ, Lorimer HE, Jacobs HT (2000). Coupled leading- and lagging-strand synthesis of mammalian mitochondrial DNA. Cell.

[59] Yasukawa T, Yang MY, Jacobs HT, Holt IJ (2005). A bidirectional origin of replication maps to the major noncoding region of human mitochondrial DNA. Mol Cell.

[60] DeBalsi KL, Hoff KE, Copeland WC (2017). Role of the mitochondrial DNA replication machinery in mitochondrial DNA mutagenesis, aging and age-related diseases. Ageing Res Rev.

[61] Naviaux RK, Nguyen KV (2004). POLG mutations associated with Alpers’ syndrome and mitochondrial DNA depletion. Ann Neurol.

[62] Alberio S, Mineri R, Tiranti V, Zeviani M (2007). Depletion of mtDNA: syndromes and genes. Mitochondrion.

[63] Wachsmuth M, Hubner A, Li M, Madea B, Stoneking M (2016). Age-related and heteroplasmy-related variation in human mtDNA copy number. PLoS Genet.

[64] Bai RK, Perng CL, Hsu CH, Wong LJ (2004). Quantitative PCR analysis of mitochondrial DNA content in patients with mitochondrial disease. Ann N Y Acad Sci.

[65] Michikawa Y, Mazzucchelli F, Bresolin N, Scarlato G, Attardi G (1999). Aging-dependent large accumulation of point mutations in the human mtDNA control region for replication. Science.

[66] Holt IJ, Harding AE, Morgan-Hughes JA (1988). Deletions of muscle mitochondrial DNA in patients with mitochondrial myopathies. Nature.

[67] Moraes CT (1989). Mitochondrial DNA deletions in progressive external ophthalmoplegia and Kearns-Sayre syndrome. N Engl J Med.

[68] Corral-Debrinski M (1992). Mitochondrial DNA deletions in human brain: regional variability and increase with advanced age. Nat Genet.

[69] Suomalainen A (1995). An autosomal locus predisposing to deletions of mitochondrial DNA. Nat Genet.

[70] Bosworth CM, Grandhi S, Gould MP, LaFramboise T (2017). Detection and quantification of mitochondrial DNA deletions from next-generation sequence data. BMC Bioinformatics.

[71] Adey A (2010). Rapid, low-input, low-bias construction of shotgun fragment libraries by high-density in vitro transposition. Genome Biol.

[72] Damas J, Carneiro J, Amorim A, Pereira F (2014). MitoBreak: the mitochondrial DNA breakpoints database. Nucleic Acids Res.

[73] Kunkel TA (1986). Frameshift mutagenesis by eucaryotic DNA polymerases in vitro. J Biol Chem.

[74] Streisinger G (1966). Frameshift mutations and the genetic code. This paper is dedicated to Professor Theodosius Dobzhansky on the occasion of his 66th birthday. Cold Spring Harb Symp Quant Biol.

[75] Phillips AF (2017). Single-molecule analysis of mtDNA replication uncovers the basis of the common deletion. Mol Cell.

[76] Zhao L (2019). Mitochondrial DNA degradation: a quality control measure for mitochondrial genome maintenance and stress response. Enzymes.

[77] Lujan SA, Williams JS, Kunkel TA (2016). DNA polymerases divide the labor of genome replication. Trends Cell Biol.

[78] Chen XJ, Clark-Walker GD (2018). Unveiling the mystery of mitochondrial DNA replication in yeasts. Mitochondrion.

[79] Torregrosa-Munumer R (2017). PrimPol is required for replication reinitiation after mtDNA damage. Proc Natl Acad Sci U S A.

[80] Bailey LJ, Doherty AJ (2017). Mitochondrial DNA replication: a PrimPol perspective. Biochem Soc Trans.

[81] Sambasivan R (2011). Pax7-expressing satellite cells are indispensable for adult skeletal muscle regeneration. Development.

[82] Lepper C, Partridge TA, Fan CM (2011). An absolute requirement for Pax7-positive satellite cells in acute injury-induced skeletal muscle regeneration. Development.

[83] Murphy MM, Lawson JA, Mathew SJ, Hutcheson DA, Kardon G (2011). Satellite cells, connective tissue fibroblasts and their interactions are crucial for muscle regeneration. Development.

[84] Old SL, Johnson MA (1989). Methods of microphotometric assay of succinate dehydrogenase and cytochrome c oxidase activities for use on human skeletal muscle. Histochem J.

[85] Pfeffer G (2014). Mutations in the SPG7 gene cause chronic progressive external ophthalmoplegia through disordered mitochondrial DNA maintenance. Brain.

[86] He L (2002). Detection and quantification of mitochondrial DNA deletions in individual cells by real-time PCR. Nucleic Acids Res.

[87] Tengan CH, Moraes CT (1996). Detection and analysis of mitochondrial DNA deletions by whole genome PCR. Biochem Mol Med.

[88] Martin M. Cutadapt removes adapter sequences from high-throughput sequencing reads. EMBnet.journal 2011;17.1:10-12.

[89] Teo SM, Pawitan Y, Ku CS, Chia KS, Salim A (2012). Statistical challenges associated with detecting copy number variations with next-generation sequencing. Bioinformatics.

[90] Kent WJ (2002). BLAT--the BLAST-like alignment tool. Genome Res.

[91] Chowdhury B (1994). A multicompartment body composition technique based on computerized tomography. Int J Obes Relat Metab Disord.

[92] Lonn L, Kvist H, Ernest I, Sjostrom L (1994). Changes in body composition and adipose tissue distribution after treatment of women with Cushing’s syndrome. Metabolism.

[93] Gouzi F (2013). Reference values for vastus lateralis fiber size and type in healthy subjects over 40 years old: a systematic review and metaanalysis. J Appl Physiol.

[94] Cristea A (2010). Effects of aging and gender on the spatial organization of nuclei in single human skeletal muscle cells. Aging Cell.

[95] Verdijk LB (2014). Satellite cells in human skeletal muscle; from birth to old age. Age.

[96] Sajko S (2004). Frequency of M-cadherin-stained satellite cells declines in human muscles during aging. J Histochem Cytochem.

[97] Krzywinski M (2009). Circos: an information aesthetic for comparative genomics. Genome Res.

[98] Pedregosa F (2011). Scikit-learn: machine learning in Python. J Mach Learn Res.

[99] Stewart JD (2009). Novel POLG1 mutations associated with neuromuscular and liver phenotypes in adults and children. J Med Genet.

[100] Lax NZ (2012). Sensory neuronopathy in patients harbouring recessive polymerase gamma mutations. Brain.

[101] Chrysostomou A (2016). Investigating complex I deficiency in Purkinje cells and synapses in patients with mitochondrial disease. Neuropathol Appl Neurobiol.

[102] Lax NZ (2012). Cerebellar ataxia in patients with mitochondrial DNA disease: a molecular clinicopathological study. J Neuropathol Exp Neurol.

[103] Ng YS (2017). Novel POLG variants associated with late-onset de novo status epilepticus and progressive ataxia. Neurol Genet.

[104] Deschauer M (2007). MELAS associated with mutations in the POLG1 gene. Neurology.

[105] Lavender, C.A., Lujan, S.A., Burkholder, A.B. ROTLA (reader of the lost arcs) GitHub. May 13, 2020, Available from: https://github.com/NIEHS/ROTLA. Accessed 13 May 2020.

[106] Lavender, C.A., Lujan, S.A., Burkholder, A.B. ROTLA (Reader of the Lost Arcs). May 13, 2020 at Zenodo, https://zenodo.org/record/3966359#.Xyg79EkpDUI. Accessed 13 May 2020.

[107] Lujan, S.A., Longley, M.J., Humble, M.H., Lavender, C.A., Burkholder, A.B., Blakely, E.L., Alston, C.L., Gorman, G.S., Turnbull, D. M., McFarland R., Taylor, R.W., Kunkel, T.A., and Copeland, W.C. Mitochondrial DNA deletion detection from human tissues. DNA datasets. dbGaP. August 11, 2020. Available from http://www.ncbi.nlm.nih.gov/projects/gap/cgi-bin/study.cgi?study_id=phs002052.v1.p1. Accessed 11 Aug 2020.

