# Supplementary material for: Ultrasensitive deletion detection links mitochondrial DNA replication, disease, and aging
Source: Genome Biol. 2020 Sep 17;21:248. doi: 10.1186/s13059-020-02138-5 (PMC7500033; doi:10.1186/s13059-020-02138-5)
Supplement: Supplementary file 1 — Additional file 1: Results. Figure S1. Mitochondrial DNA content during the LostArc procedure. Figure S2. Internal controls for validation of the LostArc method. Figure S3. The COX-ve count is poorly explained by deletions in Complex IV genes alone. Figure S4. LostArc Report example #1: weighted mean of three HEK samples. Figure S5. LostArc Report example #2: young Gwt sample M01. Figure S6. Example fits to alternative replication/deletion models. Table S1. Sample list and deletion mapping statistics. Table S2. Patient symptoms and references associated with POLG variant samples [99–104]. [file 13059_2020_2138_MOESM1_ESM.docx]

Supplementary Materials

**Table S1. Sample list and deletion mapping statistics.** Pol γ variants in *trans* and in *cis* are separated by semicolons (;) and slashes (/), respectively. Pol γ variant combinations reported for the first time are underlined. Novel Pol γ variants are underlined and written in bold and italics. Histology: COX-ve = percent of muscle fibers deficient in cytochrome *c* oxidase activity; RRF = percent of ragged red muscle fibers assessed by Gomori Trichrome staining. Deletion loads are given per Mbp mapped to the mtDNA reference. unk. = unknown. WT = wild type. na = not applicable. nd = not determined.

**Table S2. Patient symptoms and references associated with *POLG* variant samples.** Pol γ variants in *trans* and in *cis* are separated by semicolons (;) and slashes (/), respectively. Pol γ variant combinations reported for the first time here are indicated with bold font. Wholly novel Pol γ variants are written in bold and italics. PEO = progressive external ophthalmoplegia. unk. = unknown. n/a = not applicable. nd = not determined.


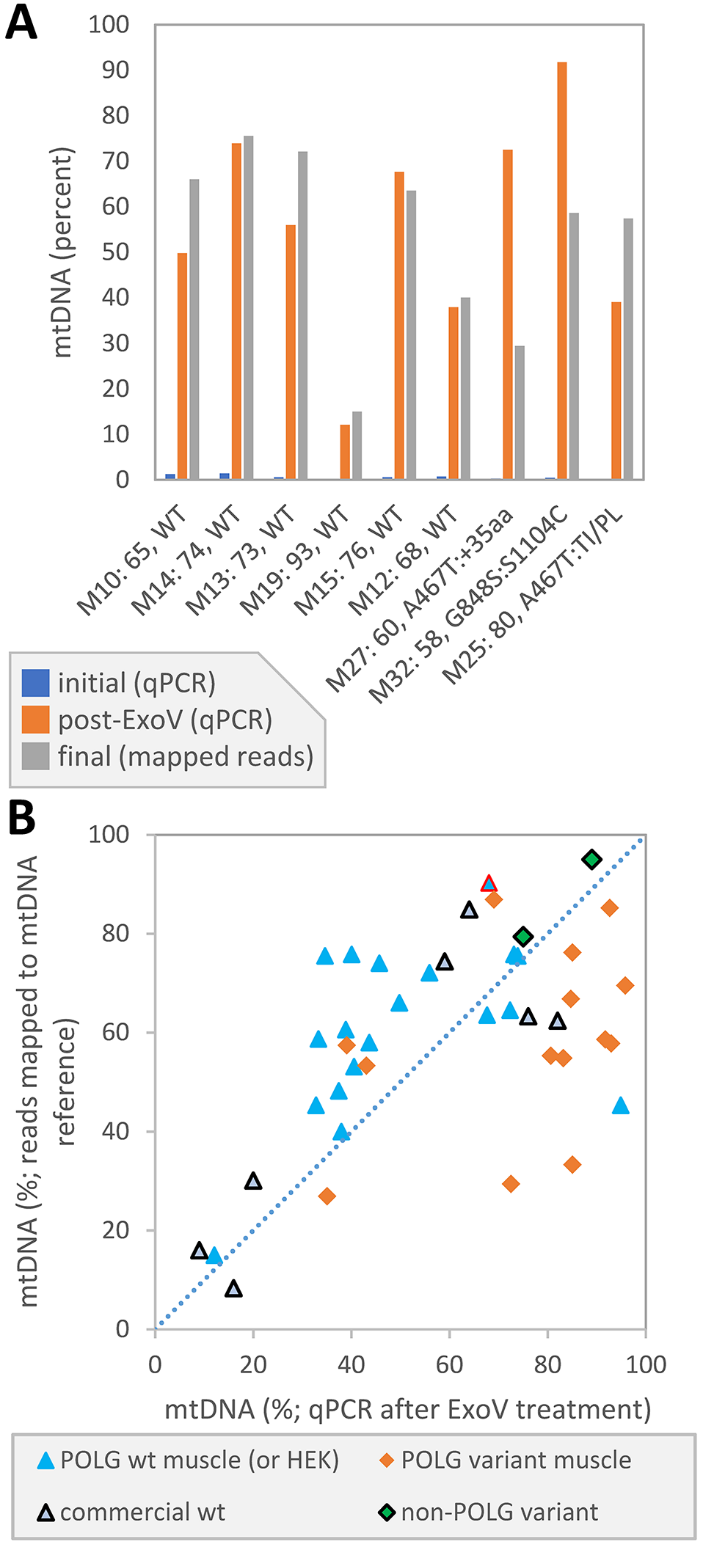


**Figure S1. Mitochondrial DNA content during the LostArc procedure.** A) Comparison of mtDNA content across samples and method steps (**Fig. 1b**). All samples were used where each measure is known. B) MtDNA fractions after read mapping (**Fig. 1b**, Step 7) correlate with fractions measured by qPCR (**Fig. 1b**, Step 4b; *p* = 0.00035, F-test of least squares linear regression). All samples were used where each measure is known. Clustering above or below the diagonal (dotted line) would indicate systematic bias. Such clustering is not significant (*p* > 0. 05; binomial test). Further, ratios of the two measures are centered near 1 (mean = 1.10; skew = 0.37, not significant, *p* > 0.05). The weakness of the correlation (Pearson *r* = 0.53; Spearman *ρ* = 0.41) cannot be fully explained by loss of PCR primer sites. Data from non-Gvar samples and commercially obtained Gwt DNA, used in pilot experiments, are included here for comparison but are not used in any other analyses.

**
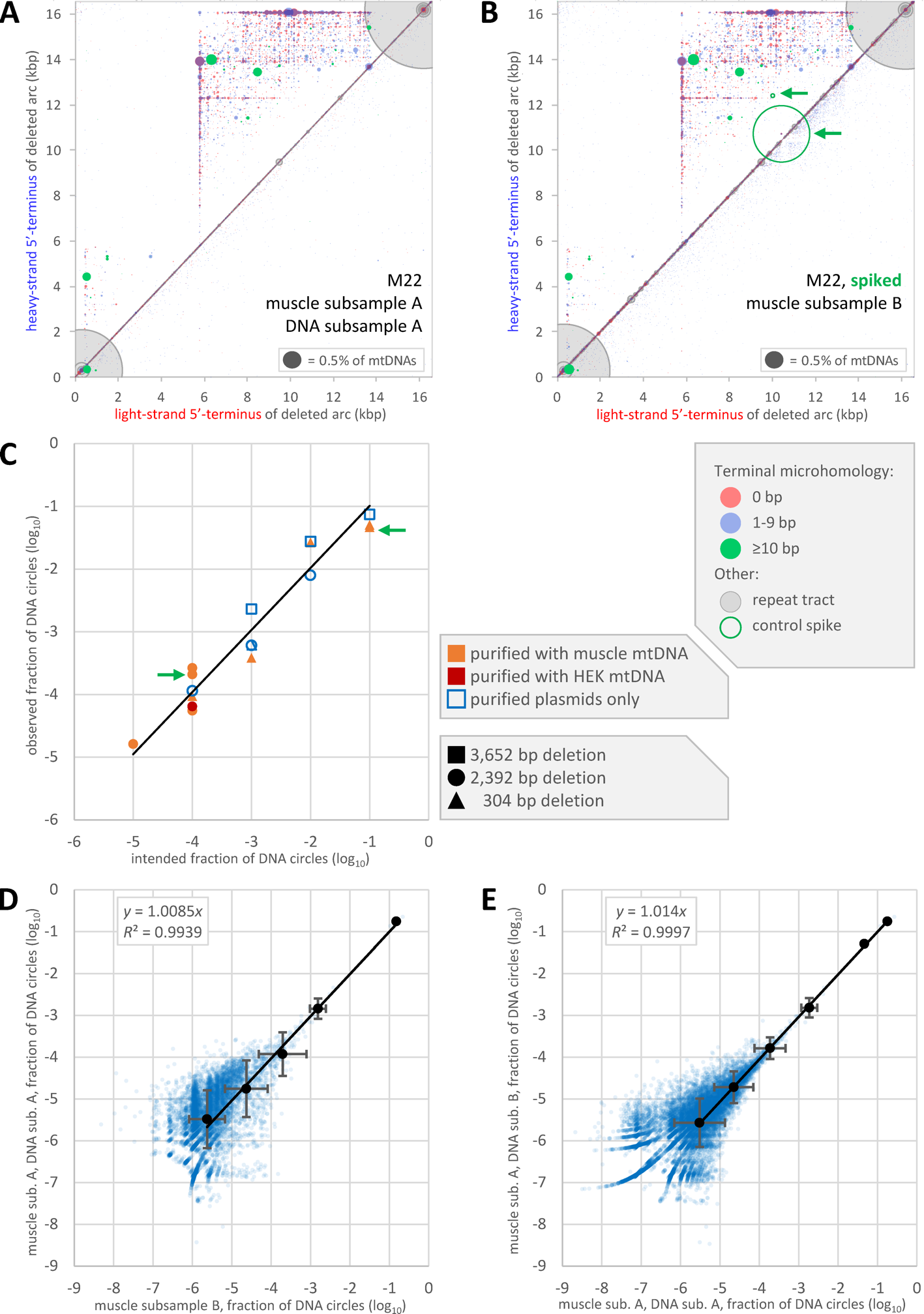
**

**Figure S2. Internal controls for validation of the LostArc method.** A) A Bubble Map of deletions in muscle sample M22. B) A Bubble Map for M22 spiked with control plasmids (4.2 to 7.5 kbp; full length minus deletion length) containing mtDNA segments with varying deletion sizes. Note that the frequencies of other mtDNA deletions are essentially unchanged by plasmid addition. C) A graph of input spiked construct fractions versus observed fractions. There is strong correlation (black trendline; *R*^2^ = 0.940; intercept set to origin) regardless of plasmid length, deletion size (304 to 3,652 bp; data point shapes), input frequency (10^-5^ to 10^-1^), or sample milieu (data point colors). This validates the accuracy of frequency measurements, indicates that concentrations of individual deletions are largely independent of one another, and confirms the absence of size selection within the LostArc pipeline. Note: the relative deviation was 65% for repeated measures (n = 5) of the 2,392 bp deletion at an input frequency of 10^-4^. D-E) Semi-transparent blue circles represent frequencies of deletions detected in two subsamples of muscle sample M22. Black circles represent the same data (above 10^-6^), binned by order of magnitude and averaged (log scale). Error bars represent standard deviations. Linear regressions (black lines; equation and *R*^2^ inset) are for the averaged data. D) Deletions detected in both M22 muscle subsamples A (DNA subsample A) and B (*n* = 7,863). These samples were forked before Step 1 in Fig. 1b. E) Deletions detected in both M22 muscle subsample A DNA subsamples A and B (*n* = 13,491). These samples were forked before Step 2 in Fig. 1b. Errors are distributed normally for deletion frequencies over 10^-6^, with standard deviations (per bin) varying linearly with frequency (*R*^2^ > 0.99). Assuming an intercept at zero, this means that the relative deviation is essentially constant. Regression puts the relative deviation at 59%, but this is dominated by high frequency bins. The average relative deviation across bins is 78%.


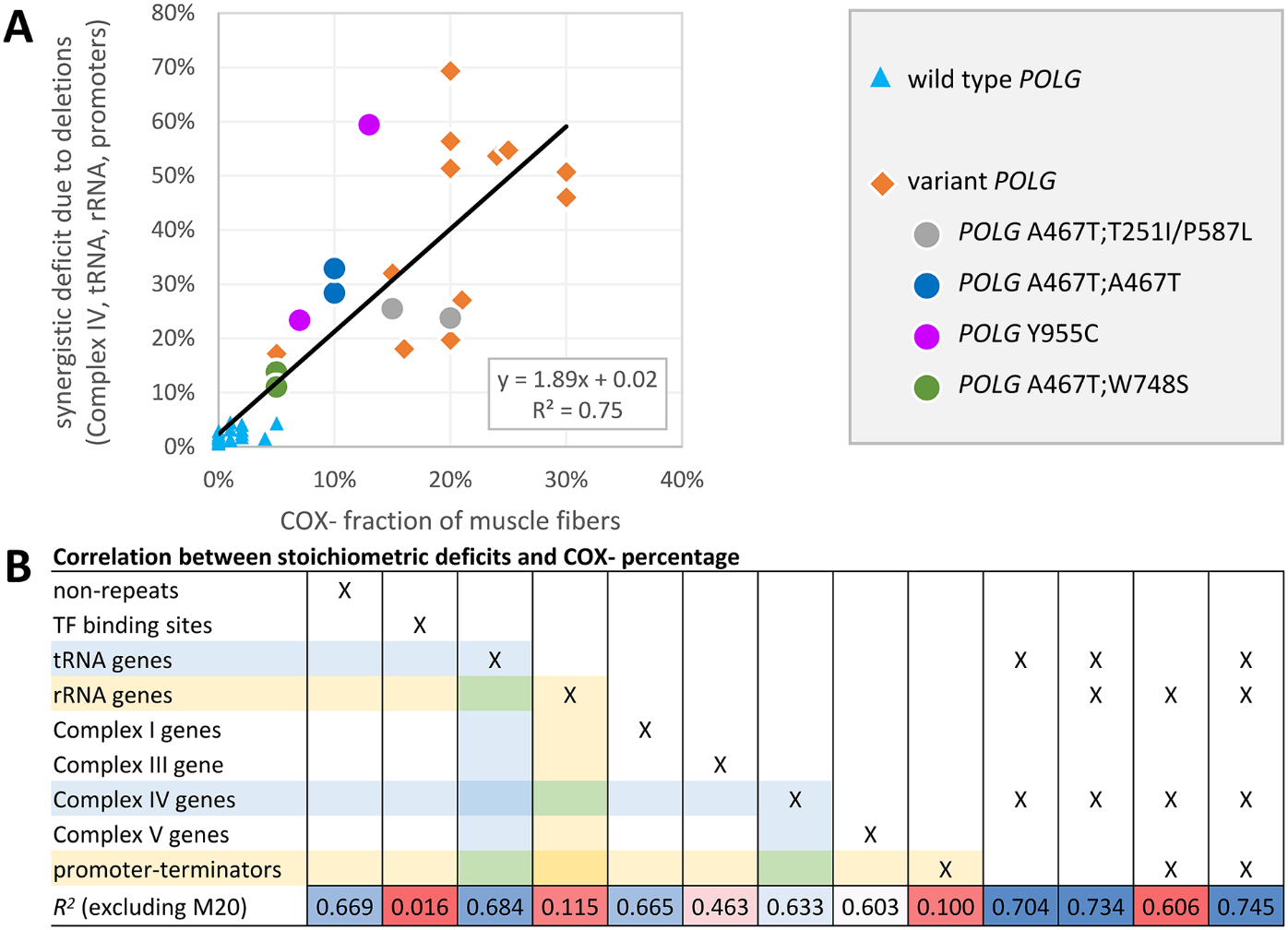


**Figure S3. The COX-ve count is poorly explained by deletions in Complex IV genes alone.** Genes and genetic features named in this figure are mtDNA encoded, except for *POLG*. A) Among 39 samples, 22 with ≥ 5% COX-ve, the best explanation accounts for deletions in mitochondrially-encoded Complex IV genes and transcription/translation machinery (tRNA, rRNA, promoters). The synergistic combination of these, allowing full stoichiometric complementation as if fibers were not separate partitions, explains 75% of the COX-ve variation. B) Other models explain the COX-ve fraction more poorly. Surprisingly, the best single-system explanation is from mitochondrial translation (tRNA genes), not Complex IV cytochrome oxidase genes. However, this is only marginally better than for all non-repeat deletions, implying that much of the tRNA explanatory power may come from the distribution of mt-tRNA genes around the whole mtDNA, thus serving as a marker for total ablation. Note: Sample M20 was excluded due to low depth (log-depth 3.9-standards of deviation below log-mean).


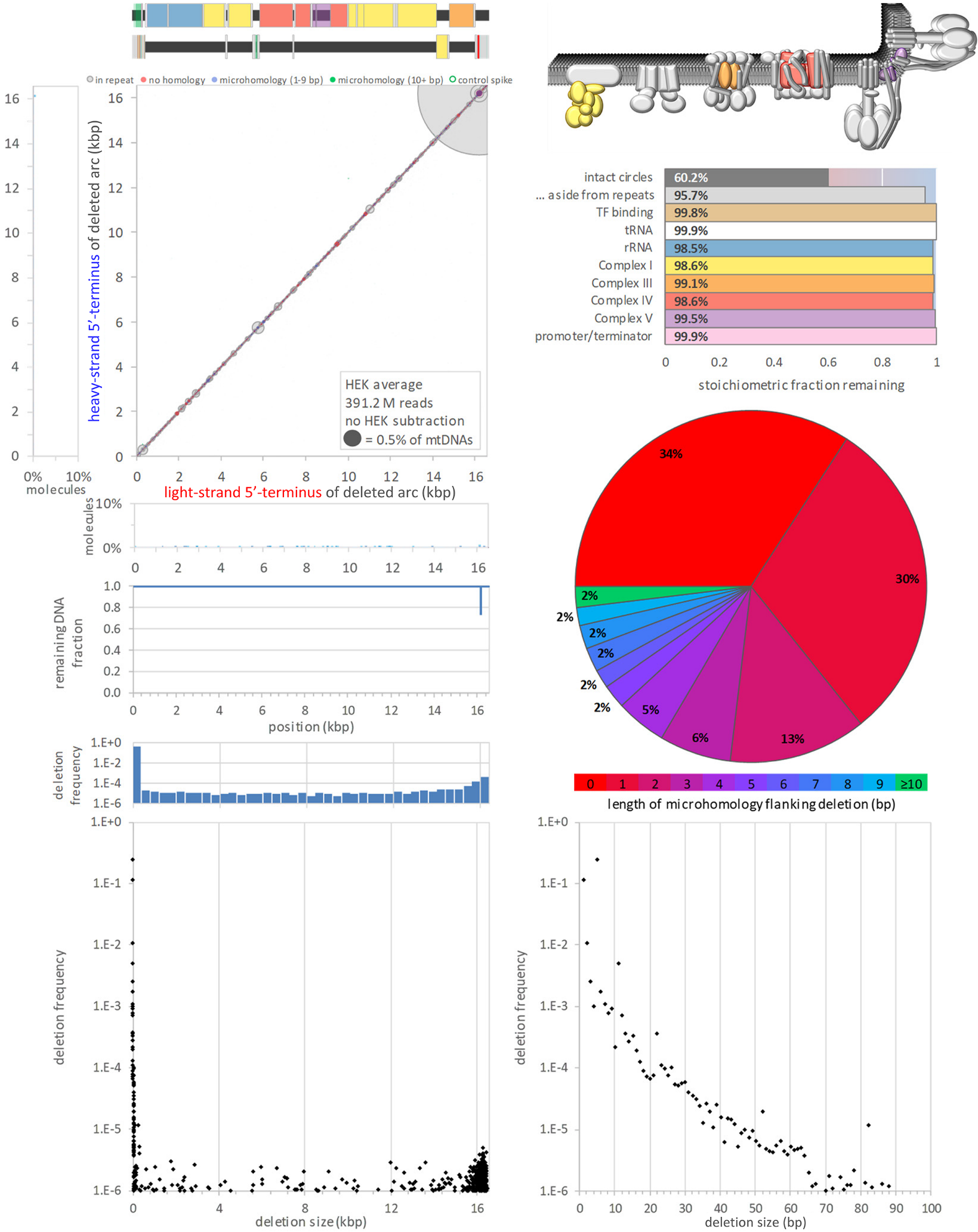


**Figure S4.** **LostArc Report example #1: weighted mean of three HEK samples.** This example LostArc Report shows deletions averaged from three samples of cultured human embryonic kidney cells (HEK-293), weighted linearly by depth. Each LostArc report contains the following information in the left column (top to bottom): a Bubble Map of deletion frequency versus terminal positions (see **Fig. 3b**), with the total ablation (see **Fig. 2c** and **Table S1**) and the mtDNA read count (see **Table S1**) inset and total rates per terminal position shown as bar graphs along each axis; a plot of ablation per position (see **Fig. 2b**); a deletion size spectrum with 400 bp bins (see **Fig. 2g**); and a deletion size spectrum with 1 bp bins (see **Fig. 2f**). Each LostArc report contains the following information in the right column (top to bottom): a diagram of the protein complexes of the electron transport chain (I to V, left to right), as in **Fig. 1a**; the fractions of mtDNA circles (grey) or mitochondrial systems (colored by system or complex; compared to COX-ve fractions in **Fig. S3**) disrupted by deletions, the latter given as lower bound estimates calculated assuming perfect stoichiometric complementation; a pie chart showing the fraction of deletions by the number of bases of terminal microhomology (see **Fig. 3a**); and spectrum of deletions <100 bp with 1 bp bins.


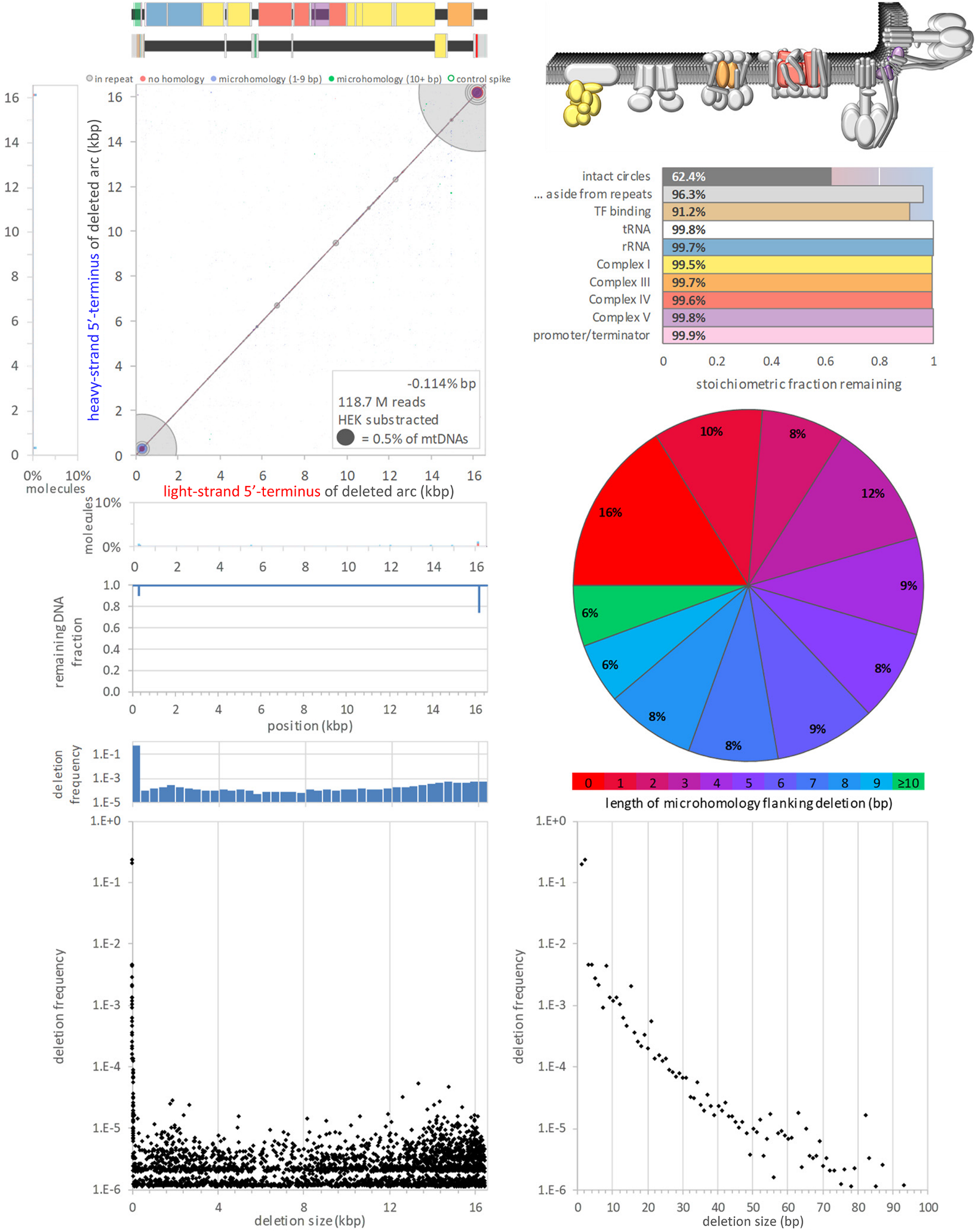


**Figure S5. LostArc Report example #2: young Gwt sample M01.** This example LostArc Report shows that even after background subtraction, young Gwt muscle samples have much higher deletion levels than HEK cells (**Fig. S4**).


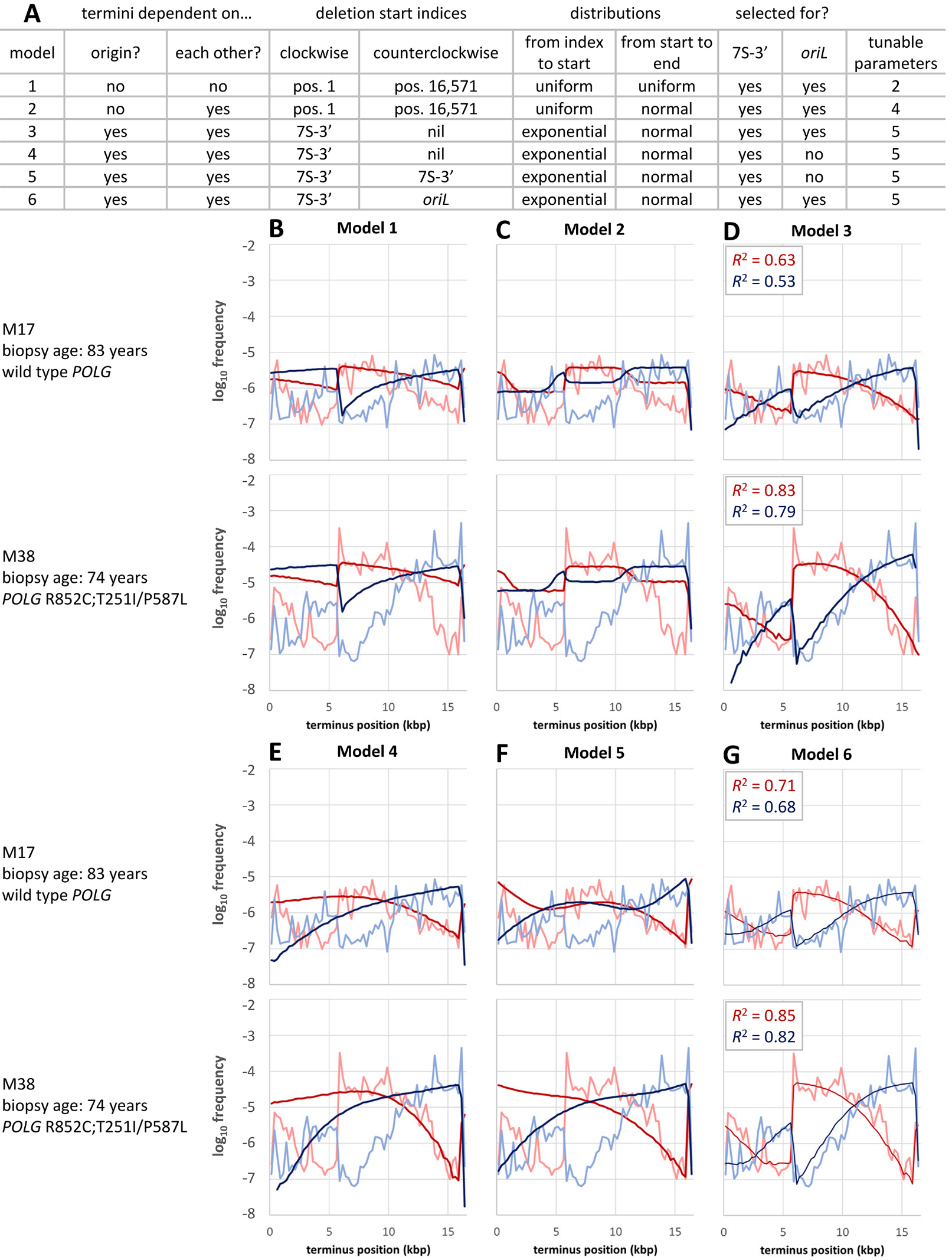


**Figure S6. Example fits to alternative replication/deletion models.** A) Characteristics of Monte Carlo models of mtDNA replication/deletion: 1) deletions begin and end anywhere without regard to origin positions or proximity of termini; 2) deletions begin anywhere without regard to origin positions and end after some normally distributed distance; 3 and 4) the probability of a deletion beginning decays exponentially with distance from the 7S-3’, deletions extend in the direction of heavy strand synthesis, and deletions end after some normally distributed distance; 5) deletions begin and end as in 3-4, but in both directions from 7S-3’; and 6) deletions begin with the same probability distributions as in 3-5 but extend in the direction of heavy strand synthesis from 7S-3’ and in the direction of light strand synthesis from *oriL*. Deletions that impinge upon 7S-3’ are removed from the population with some probability determined by the fit. The same is true for deletions that impinge upon *oriL*, except in models 4 and 5. Models 1 and 2 are most relevant for replication-independent deletion mechanisms. Models 3 and 4 are most relevant for deletion mechanisms that depend on heavy strand replication (any replication mode in **Fig. 5a-d**). Model 5 is for deletions that depend on bidirectional replication from 7S-3’ (as in **Fig. 5d**). Model 6 is for deletions that depend on unidirectional replication from either *oriL* or 7S-3’ in roughly equal measures (best for **Fig. 5a**). B-G) Deletion terminus frequencies for muscle samples M17 (*POLG* wild type) and M38 (*POLG* R382C;T251I/P587L) were plotted in the log domain versus mtDNA position. Heavy-strand-5’-terminus frequencies are represented in blue, whereas light-strand-5’-terminus frequencies are represented in red (250 bp bins). Lighter, jagged lines represent observed frequencies, which exclude deletions with extreme terminal microhomology (≥ 10 bp). Darker, smoother curves are best-fit replication/deletion Monte Carlo models. Correlation coefficients (*R*^2^) are calculated for log transformed frequencies. B) Fits to Simulation Model 1. C) Fits to Simulation Model 2. D) Fits to Simulation Model 3. E) Fits to Simulation Model 4. F) Fits to Simulation Model 5. G) Fits to Simulation Model 6.

**Additional File 2: LostArc Reports.** Each LostArc report contains the following information: mtDNA ablation levels, as summarized in **Fig. 2c** and used for COX-ve regression in **Fig. S3**; a Bubble Map of deletion frequency versus terminal positions, as in **Fig. 3b**; a deletion size spectrum, as in **Fig. 2f**, and a zoomed version showing only deletions of <100 bp; a binned version of that spectrum, as in **Fig. 2g**; and a pie chart showing the fraction of deletions by the number of bases of terminal microhomology, as was used to calculate **Fig. 3a**.

**Additional File 3: Arc Maps.** For each Gvar sample, deletion arcs are plotted relative to circular mtDNA reference. Upper plot: line width and transparency code for frequency (see deletion frequency key); colors code for deletion size and terminal homology (see Length/homology key). Lower plot: colors code for the degree of deletion terminus clustering (see Terminal Cluster key).
