# Supplementary material for: Ultrasensitive deletion detection links mitochondrial DNA replication, disease, and aging
Source: Genome Biol. 2020 Sep 17;21:248. doi: 10.1186/s13059-020-02138-5 (PMC7500033; doi:10.1186/s13059-020-02138-5)

HEK weighted average

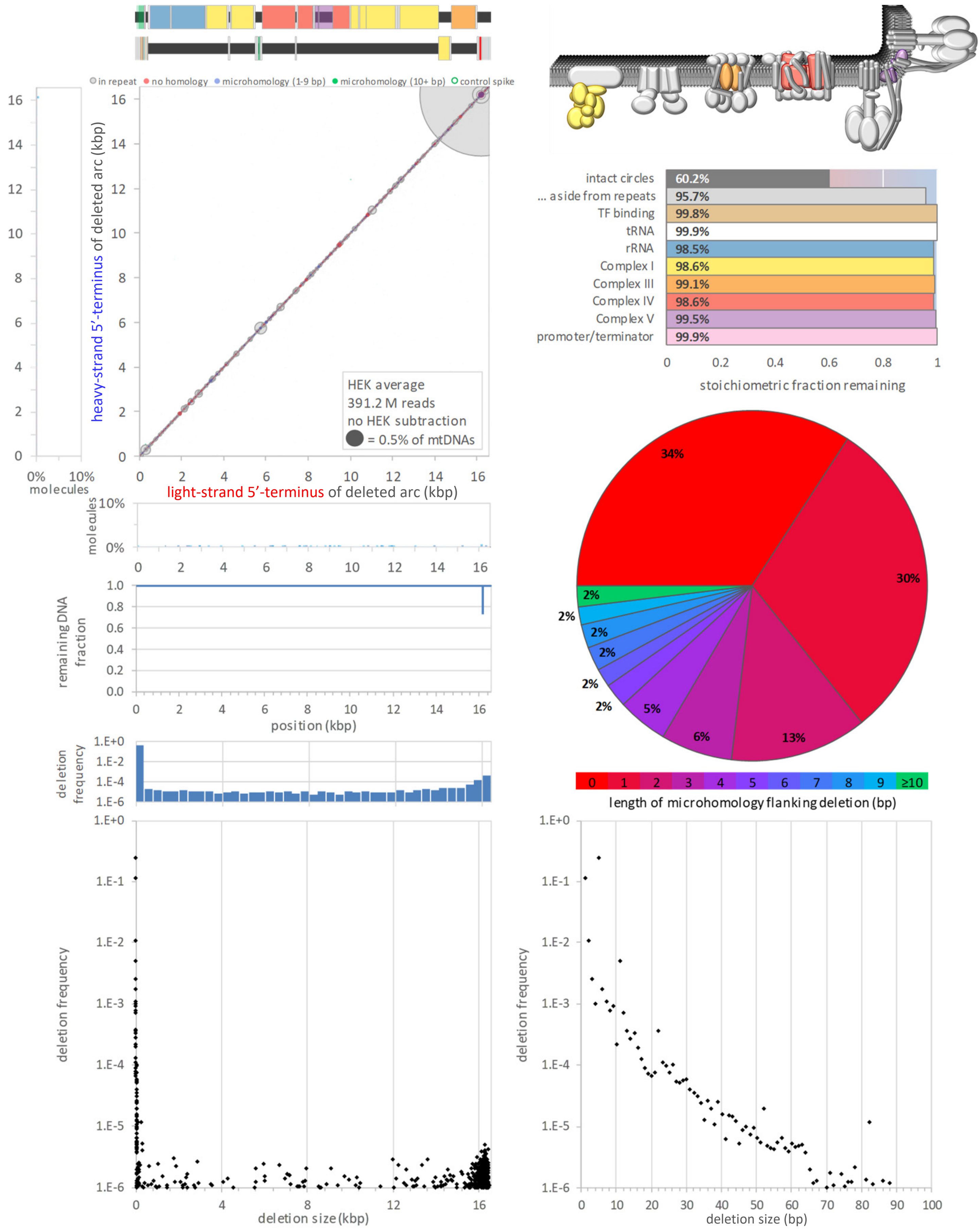

M01: *POLG* wild type, 17 years at biopsy

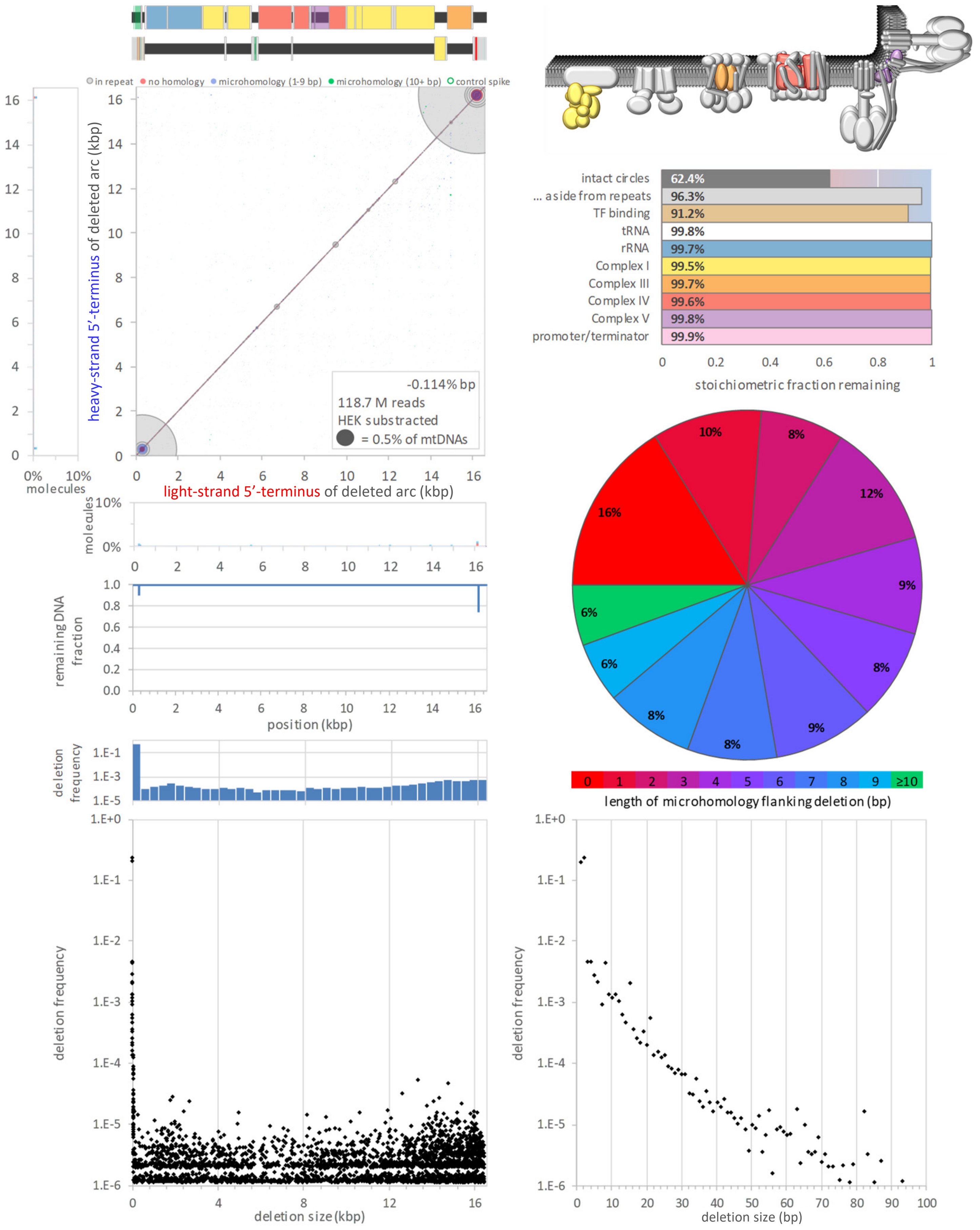

M02: *POLG* wild type, 23 years at biopsy

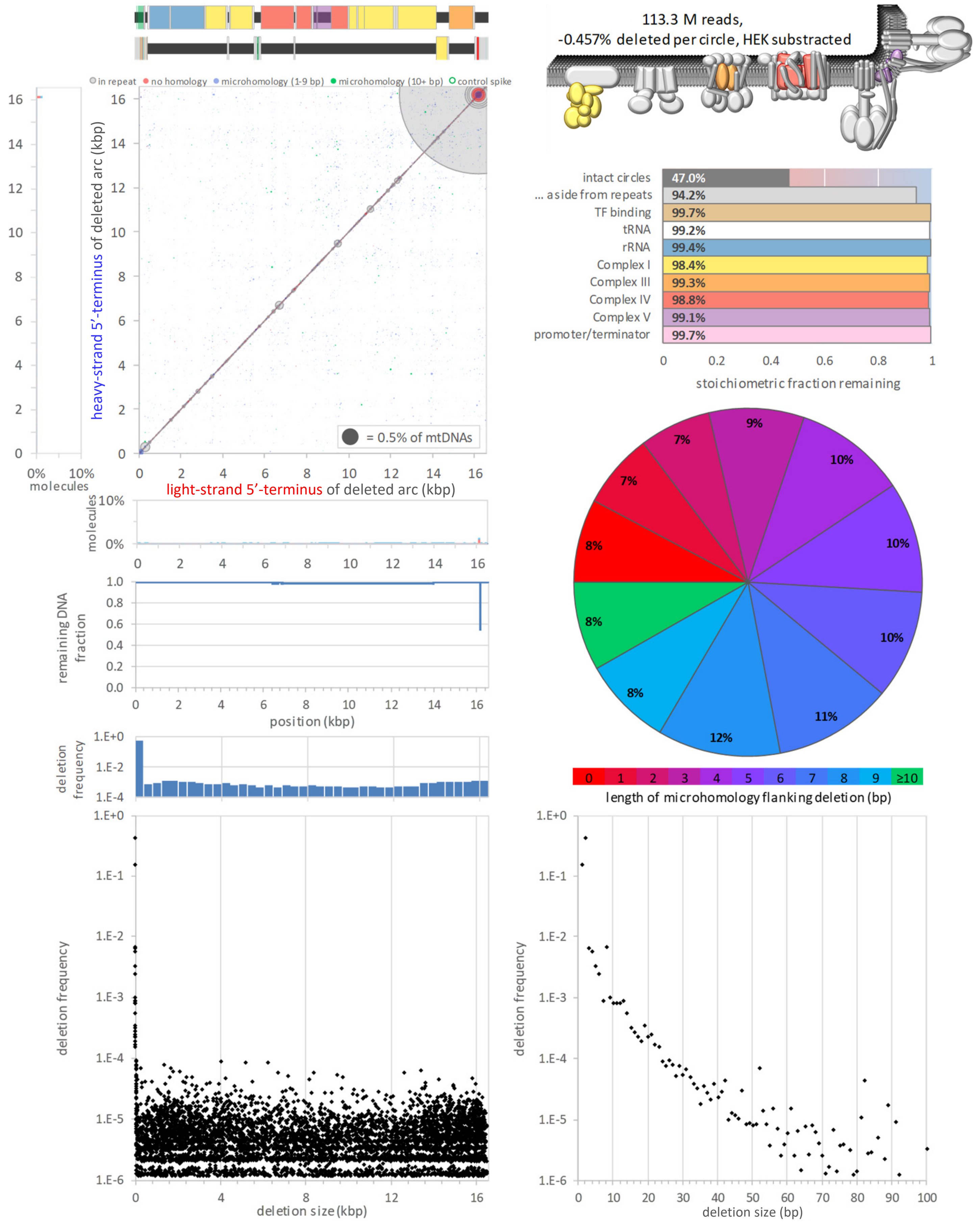

M03: *POLG* wild type, 23 years at biopsy

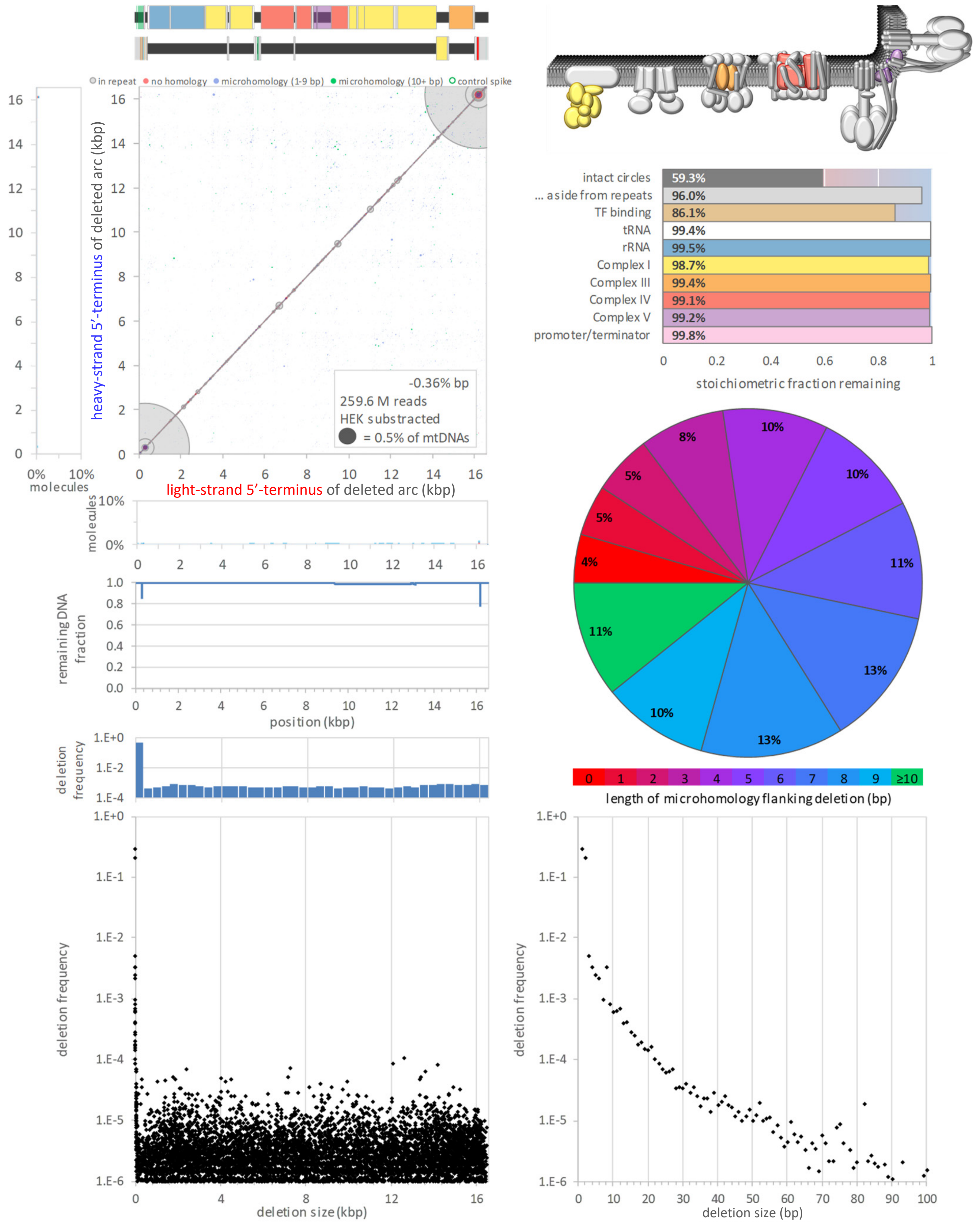

M04: *POLG* wild type, 25 years at biopsy

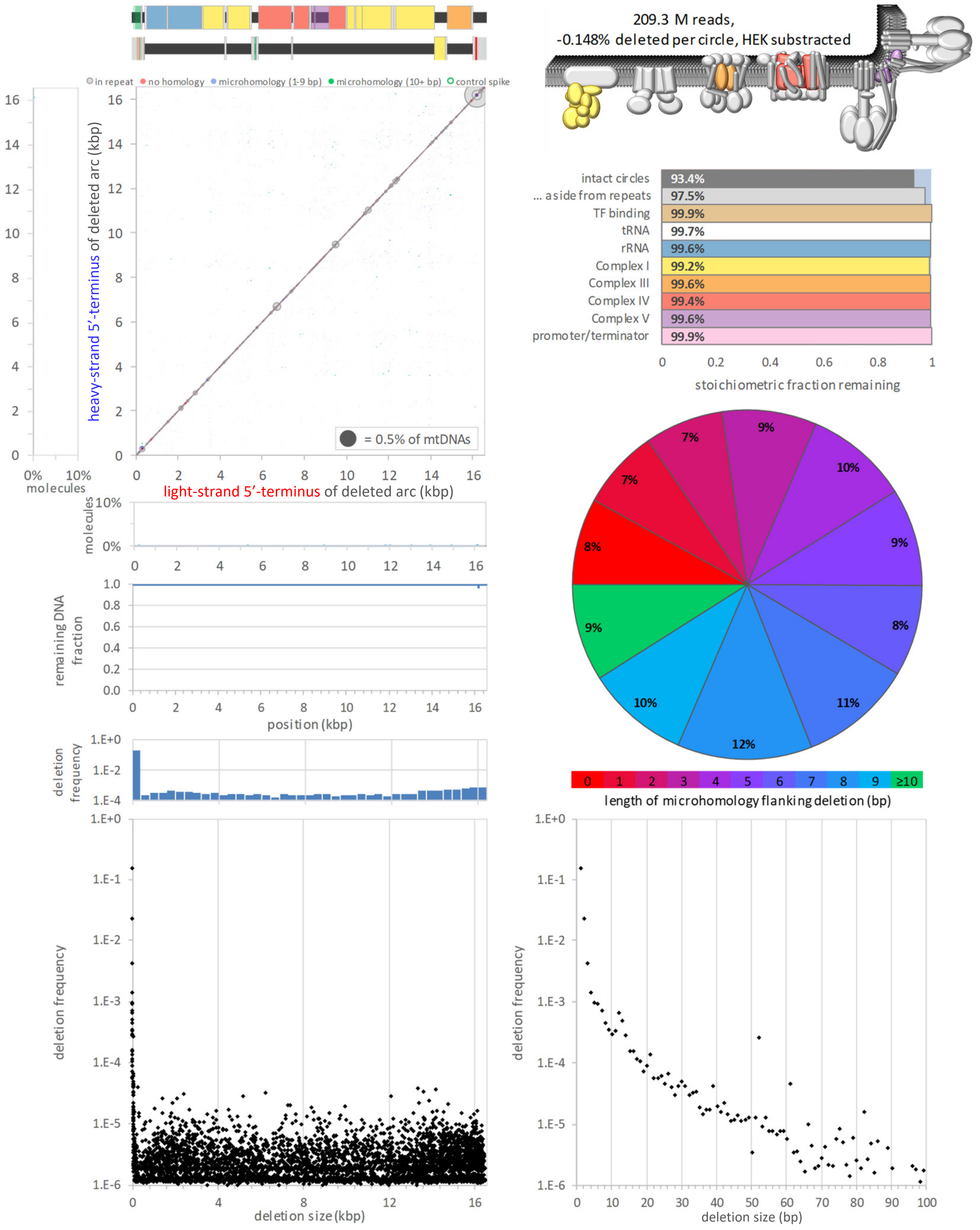

M05: *POLG* wild type, 33 years at biopsy

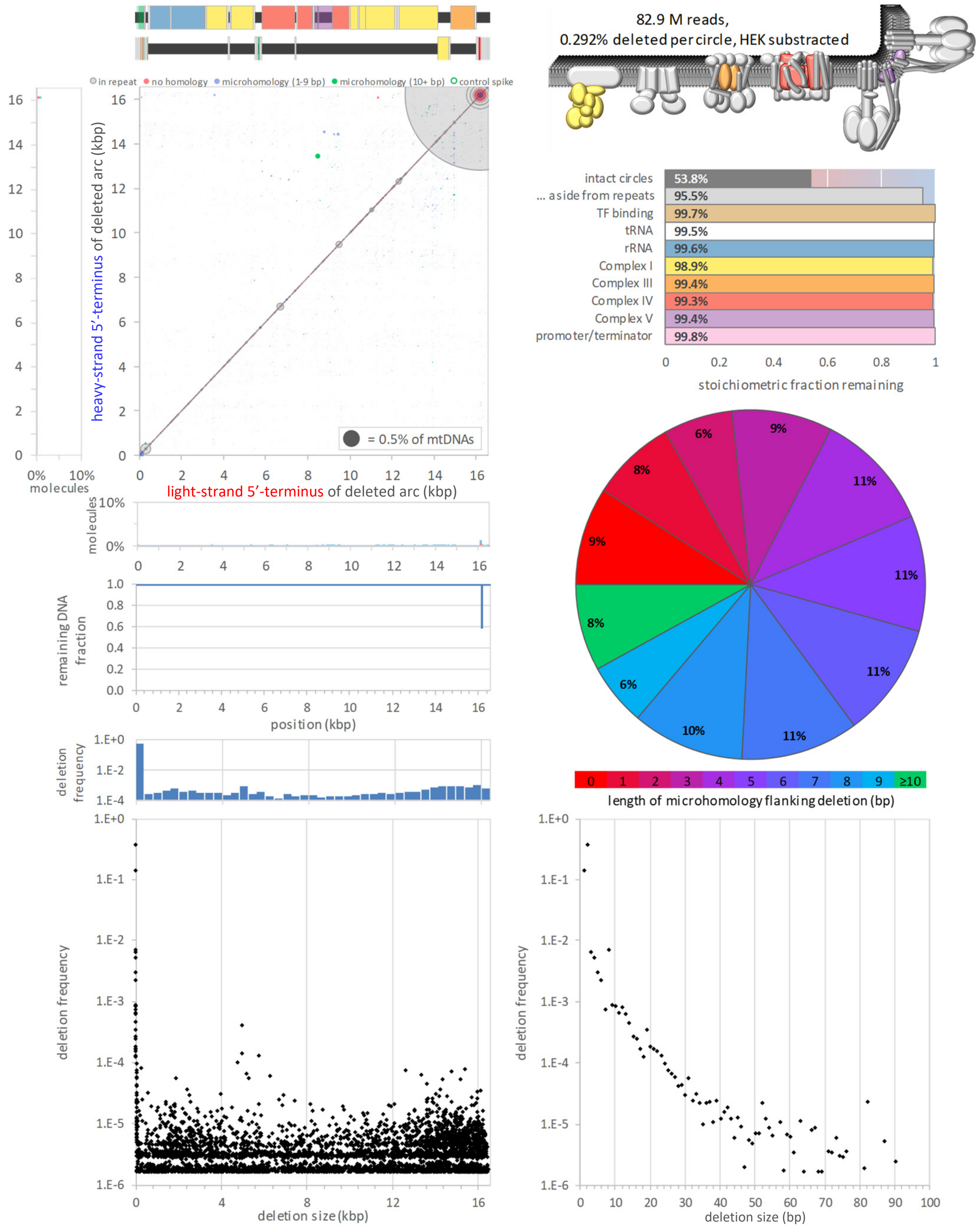

M06: *POLG* wild type, 35 years at biopsy

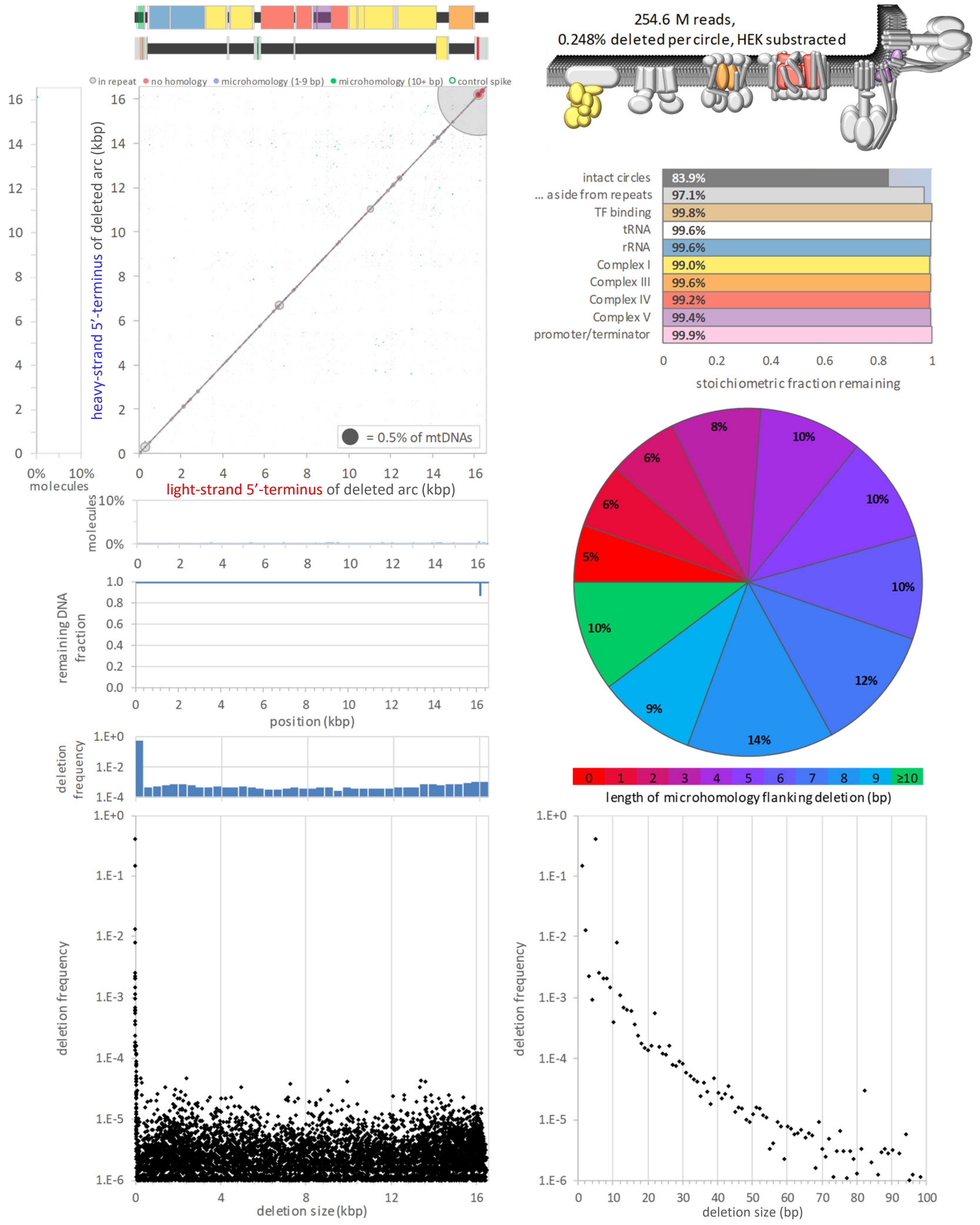

M07: *POLG* wild type, 48 years at biopsy

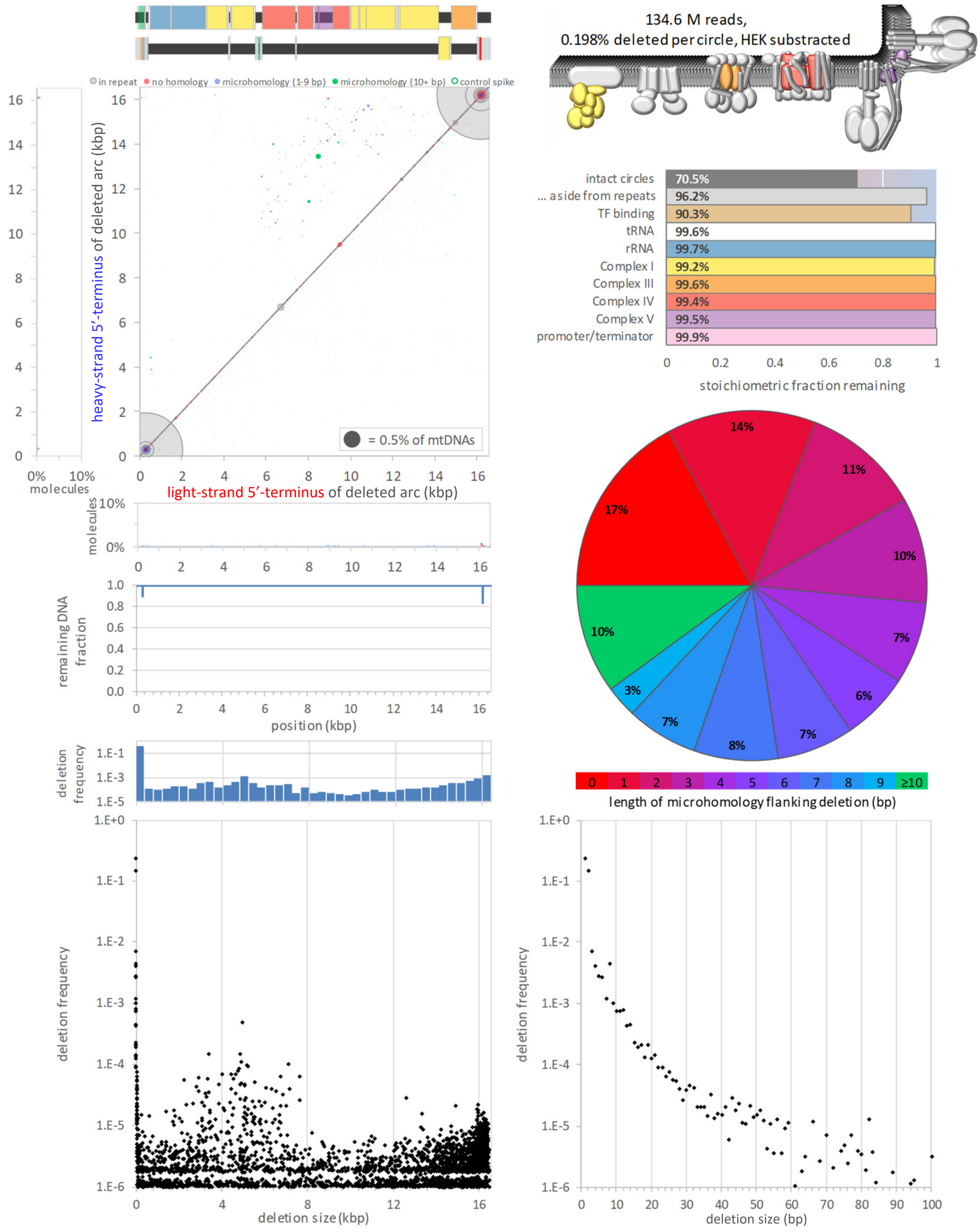

M08: *POLG* wild type, 49 years at biopsy

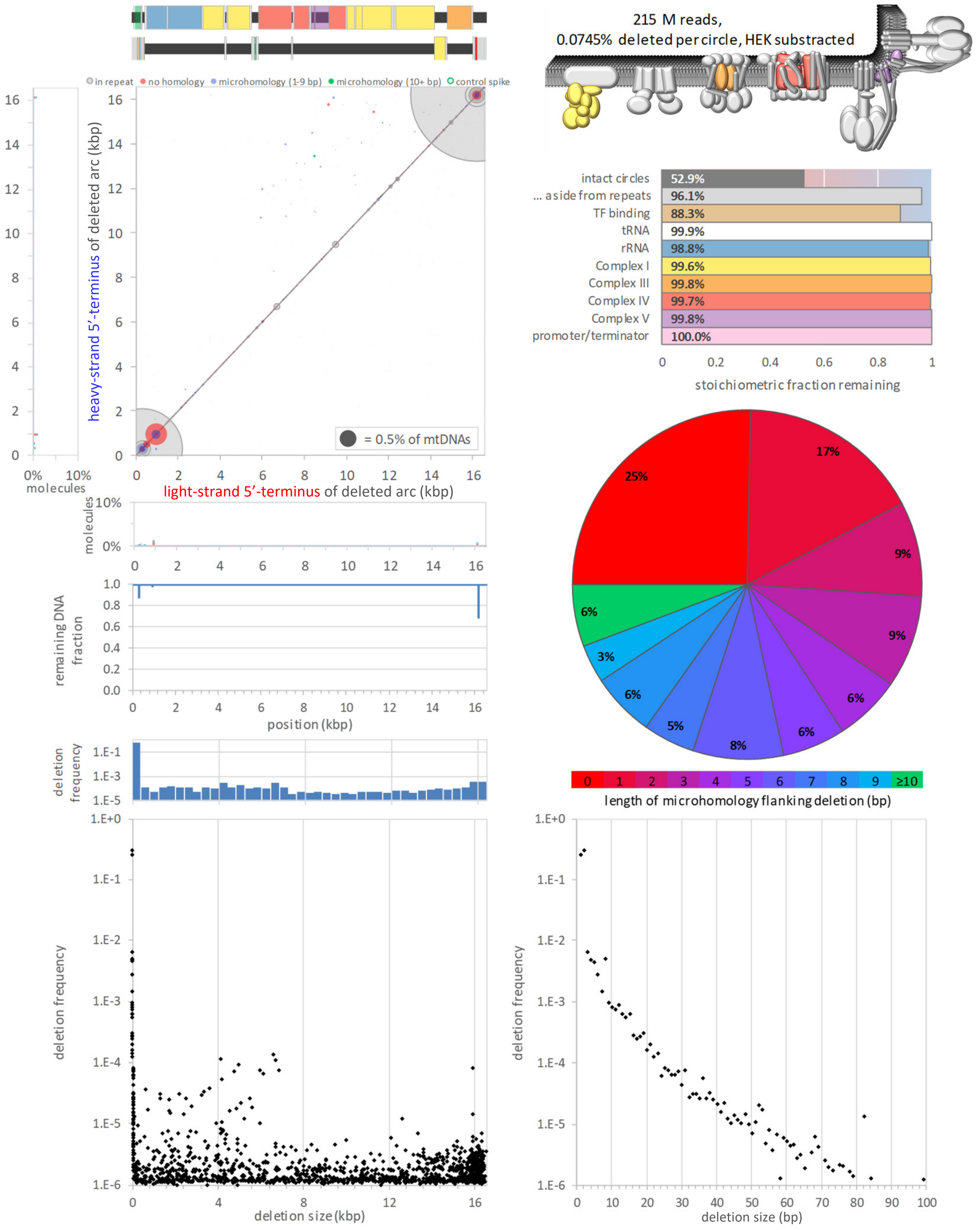

M09: *POLG* wild type, 52 years at biopsy

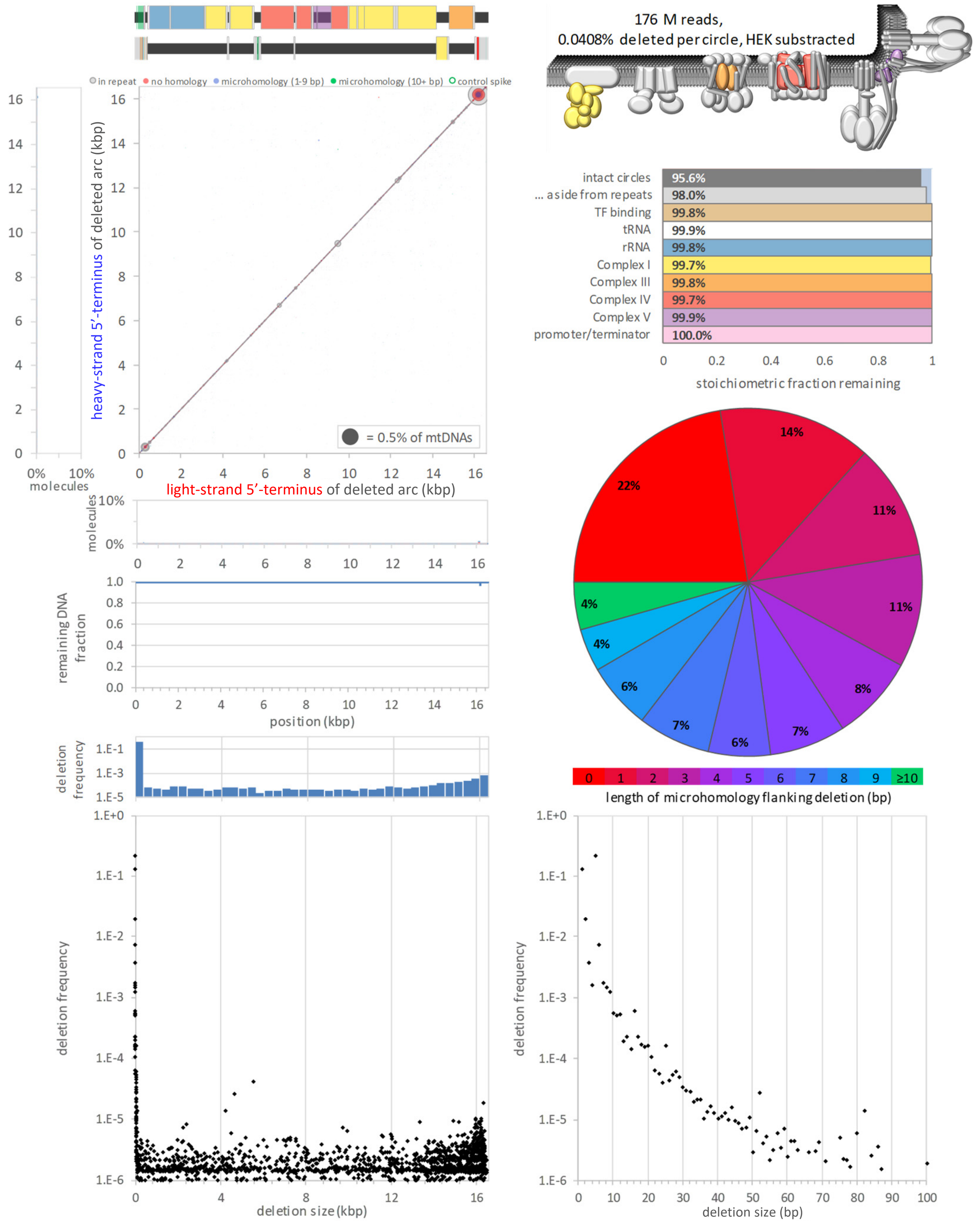

M10: *POLG* wild type, 65 years at biopsy

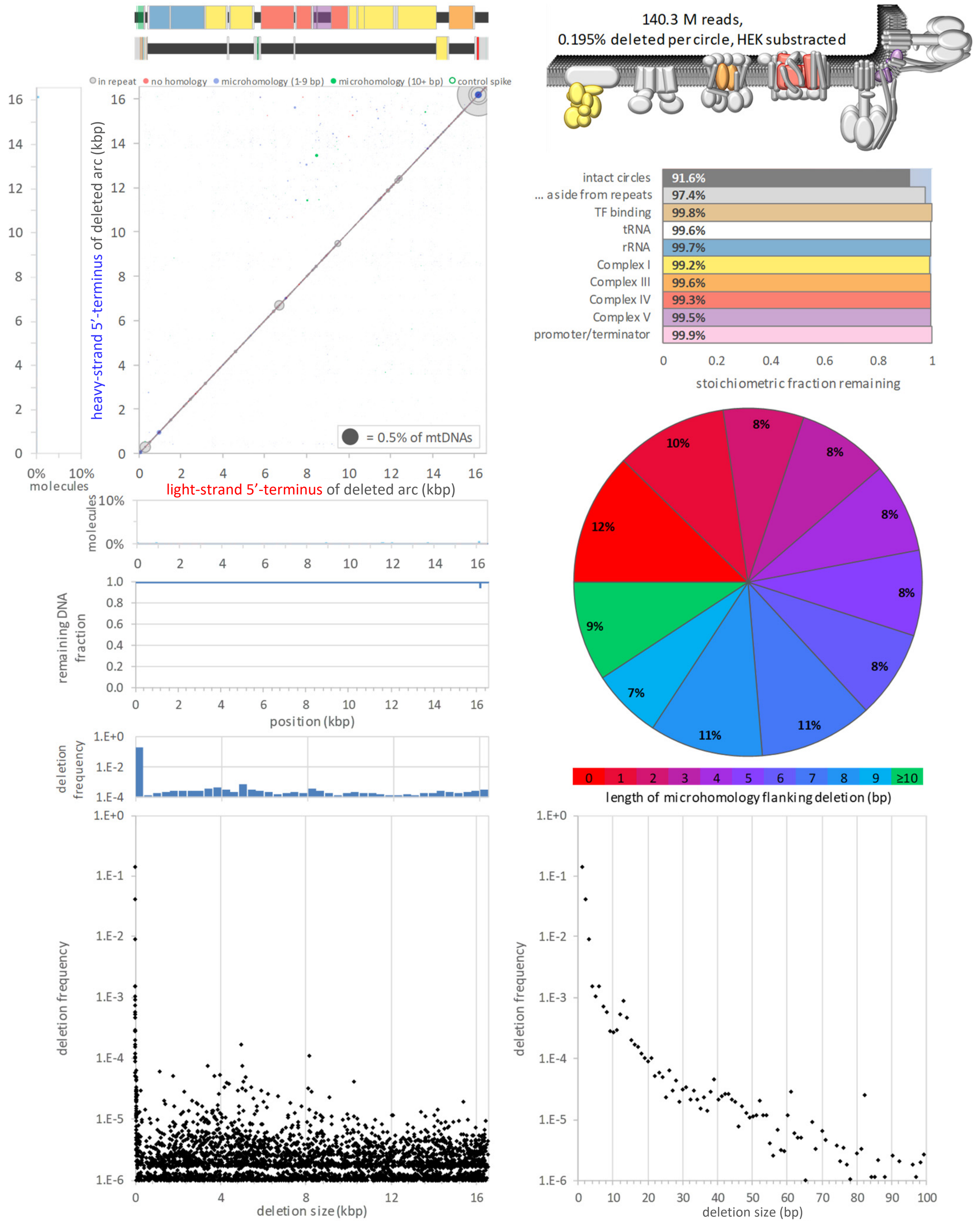

M11: 64-68 years at biopsy, cancer patient, *POLG* wt

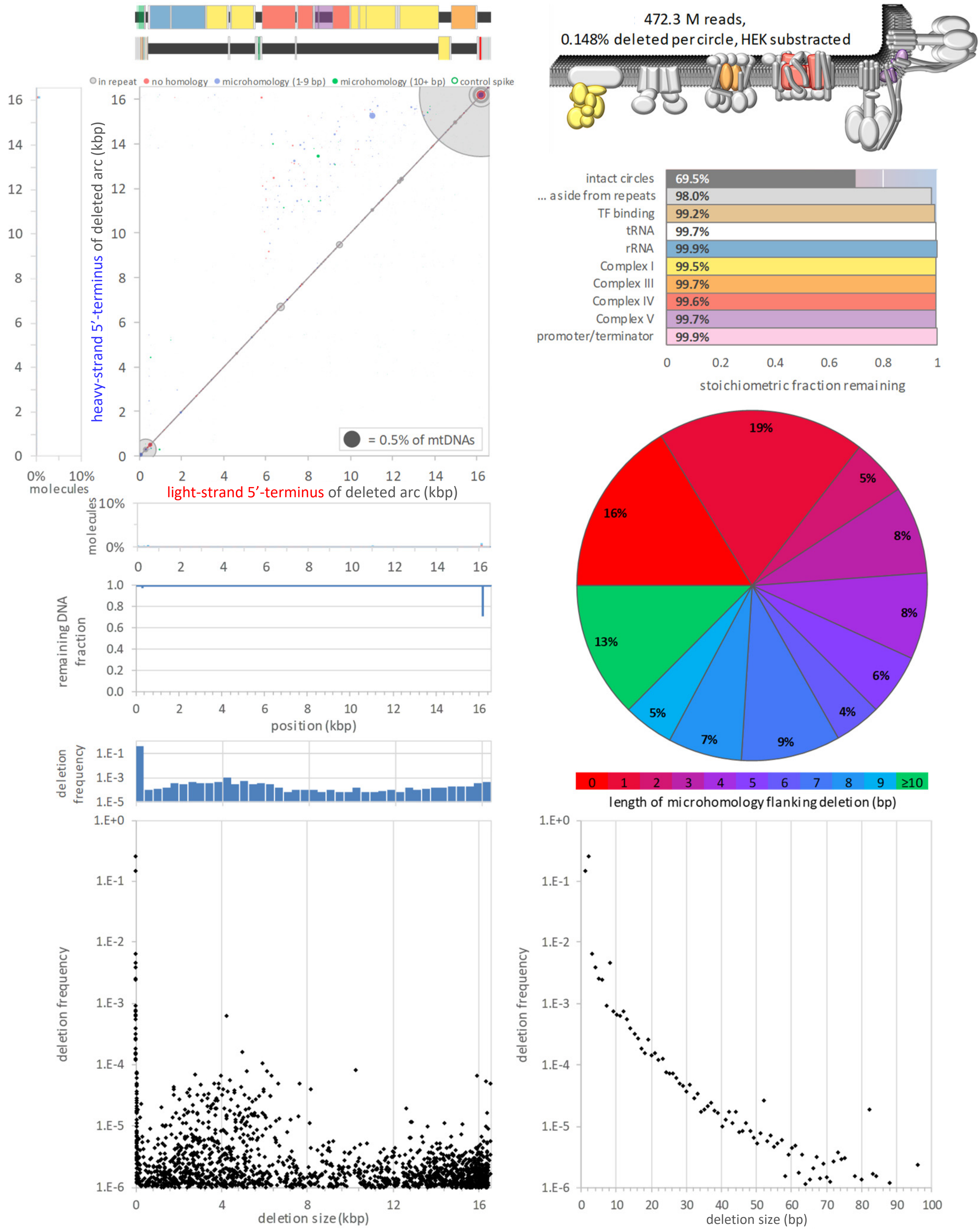

M12: *POLG* wild type, 68 years at biopsy

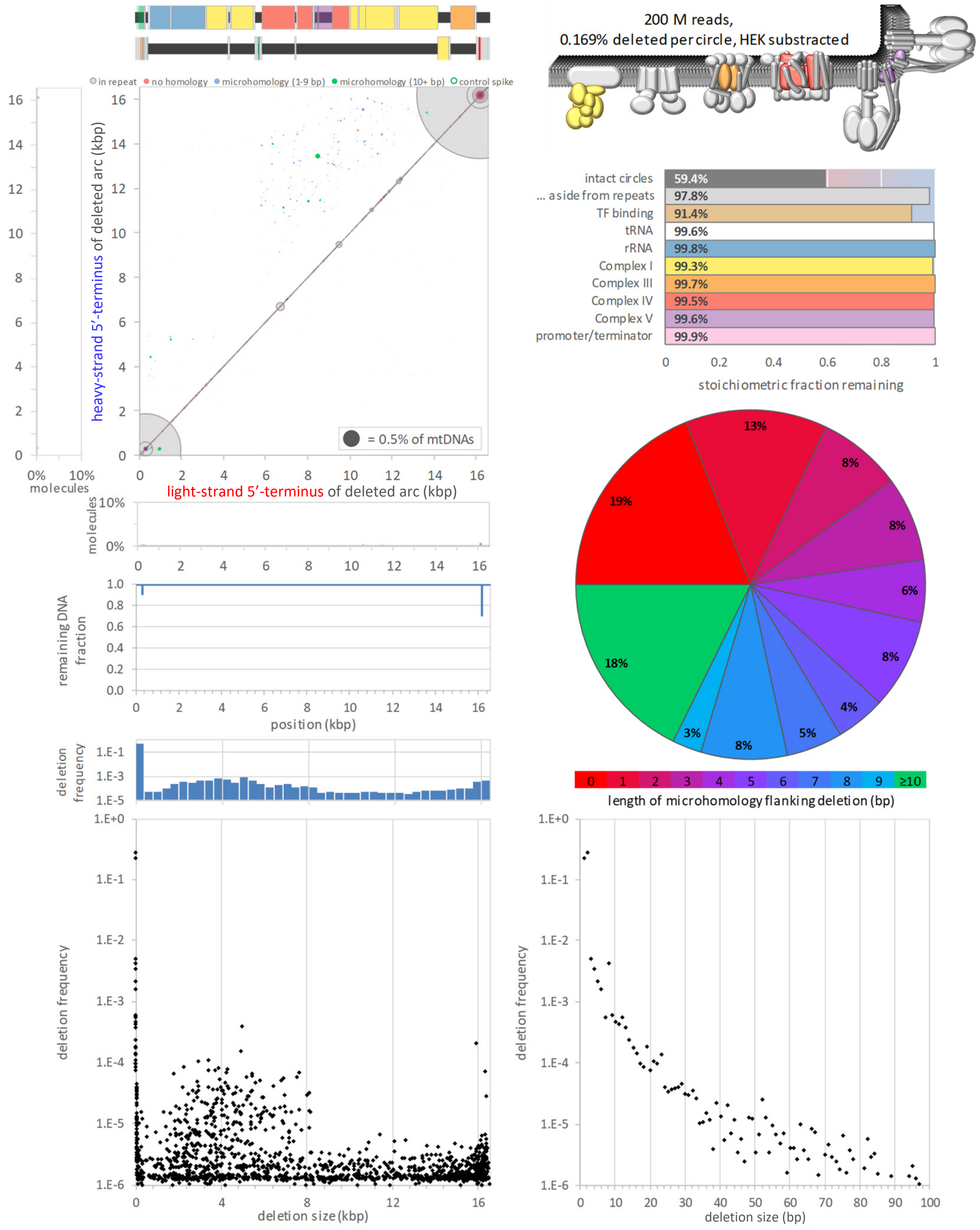

M13: *POLG* wild type, 73 years at biopsy

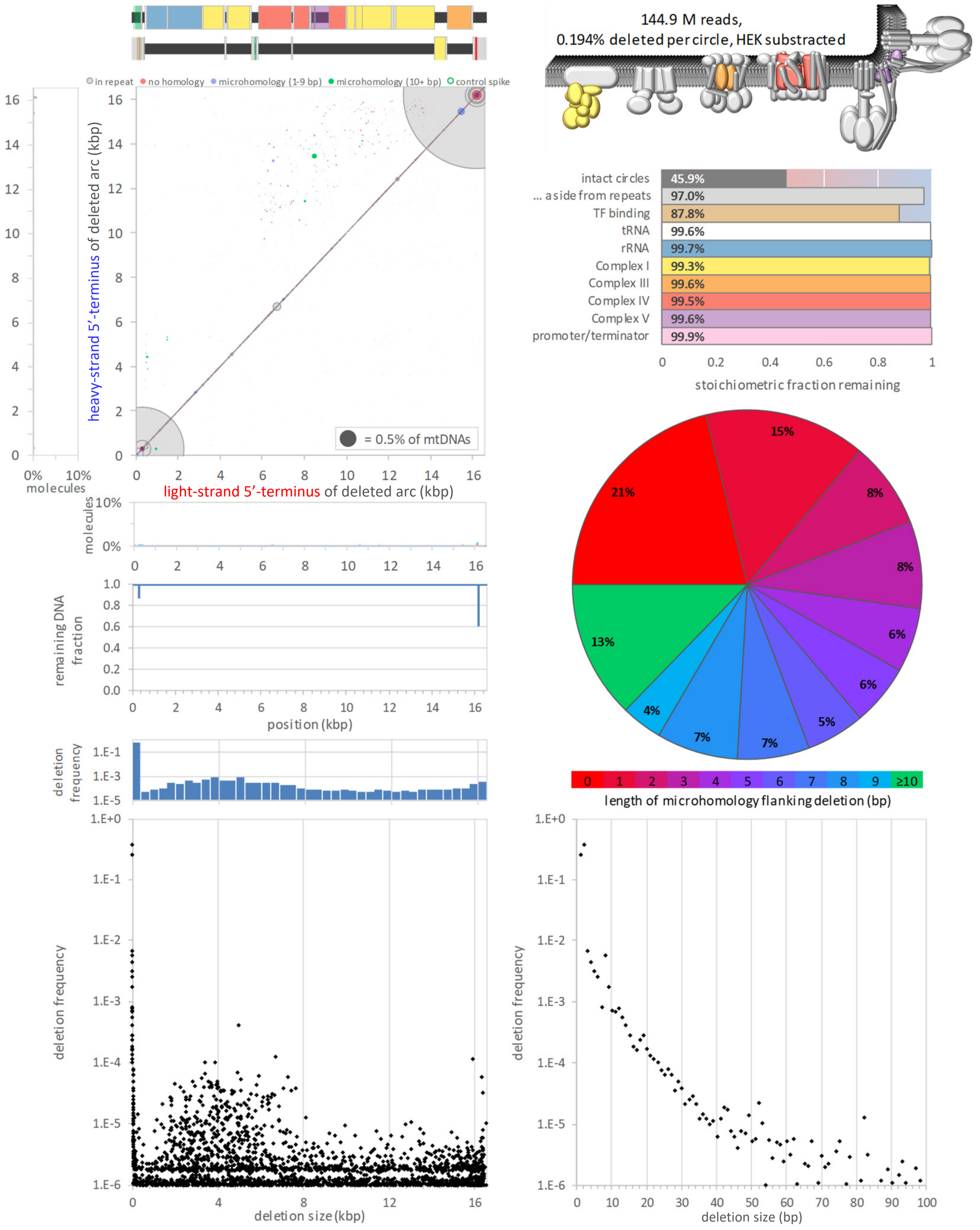

M14: *POLG* wild type, 74 years at biopsy

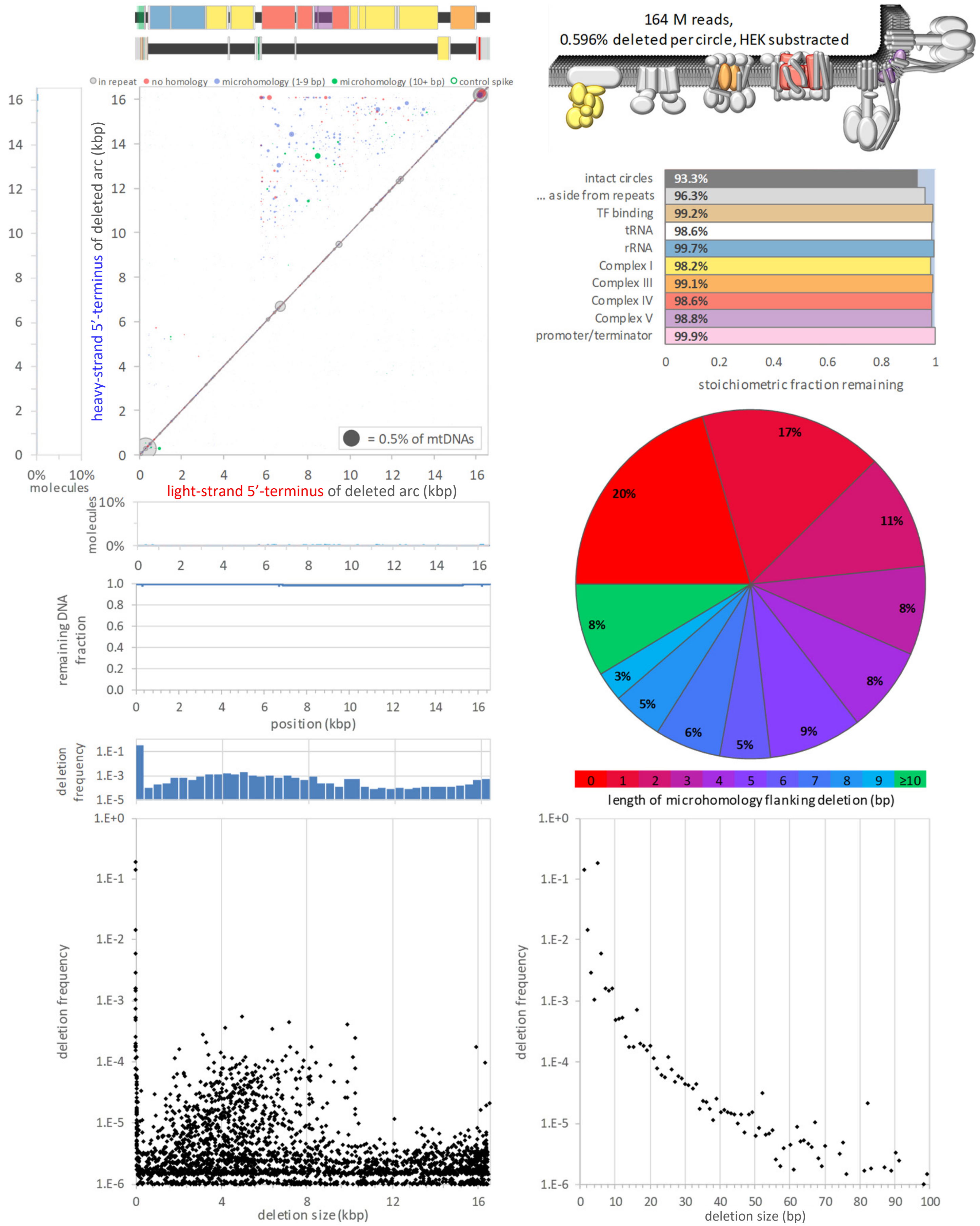

M15: *POLG* wild type, 76 years at biopsy

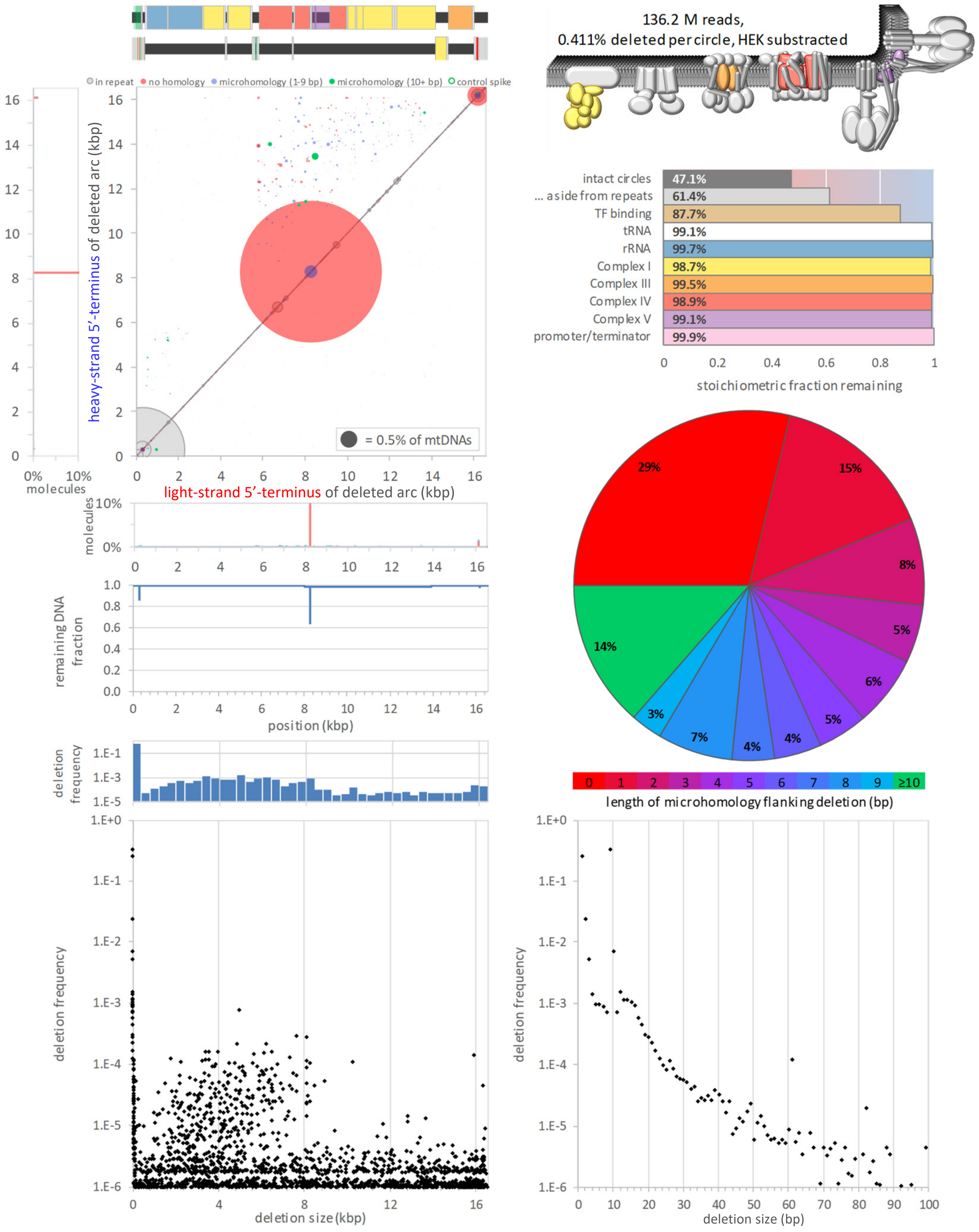

M16: *POLG* wild type, 82 years at biopsy

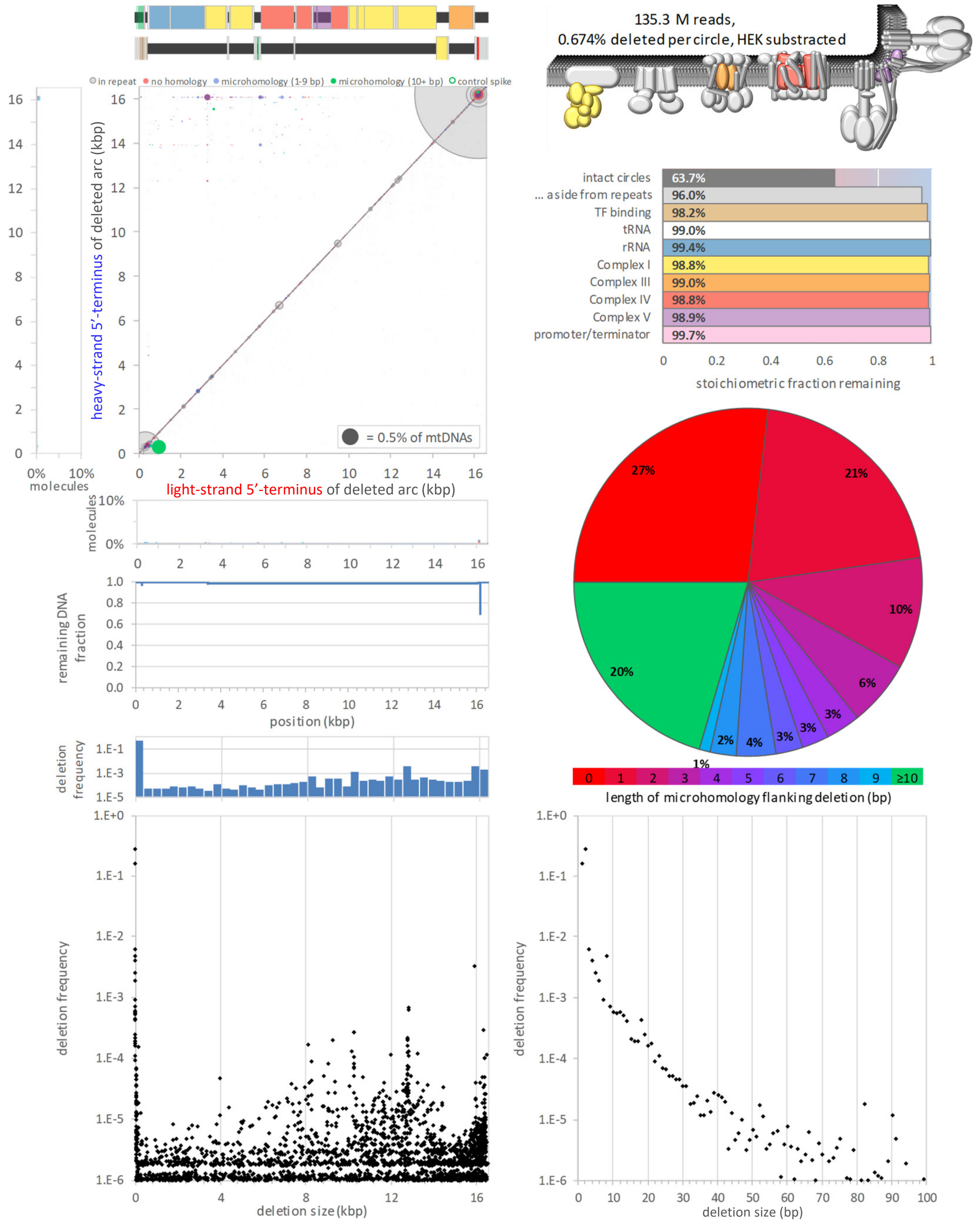

M17: *POLG* wild type, 83 years at biopsy

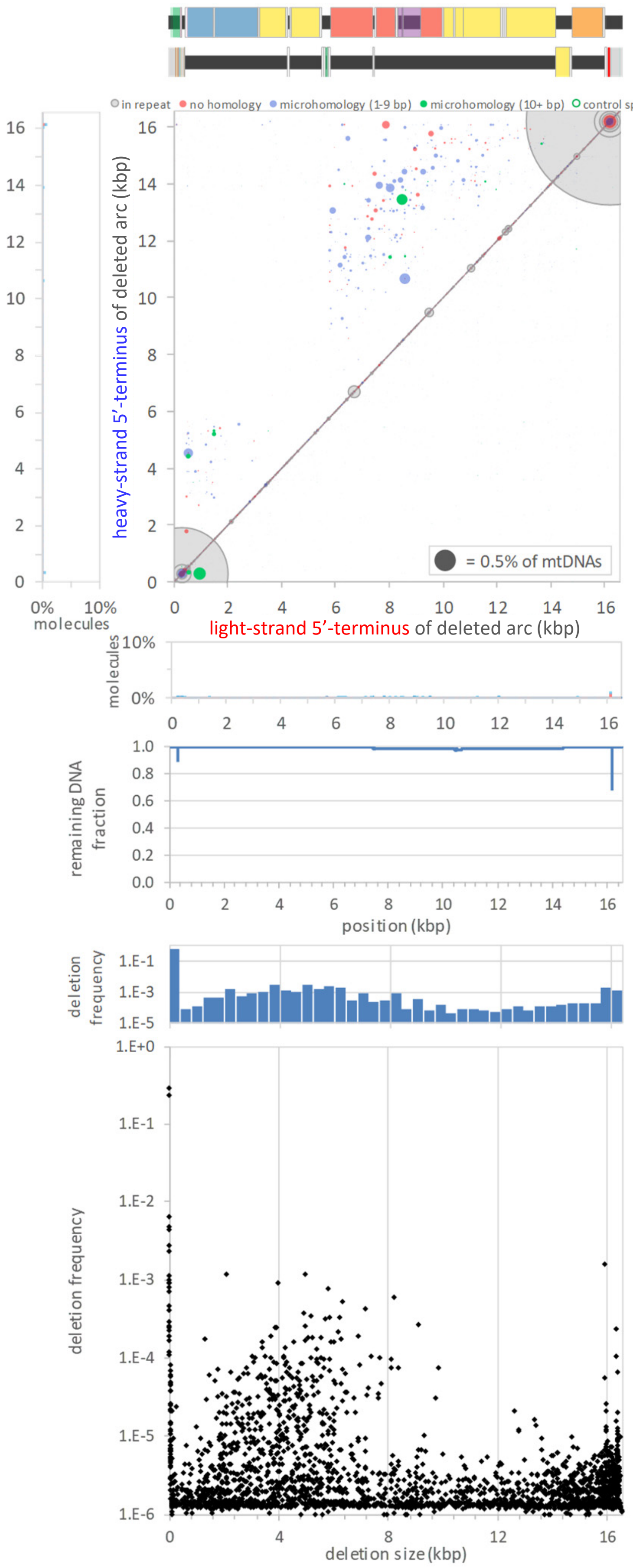

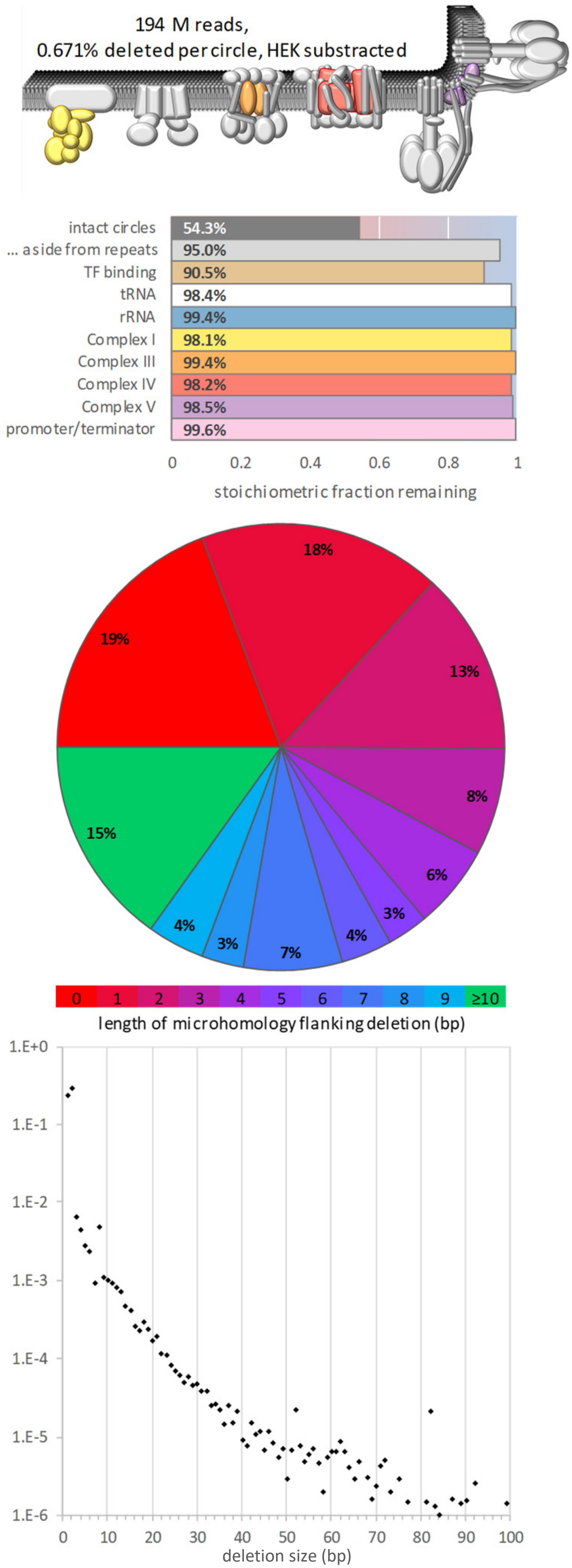

M18: *POLG* wild type, 86 years at biopsy

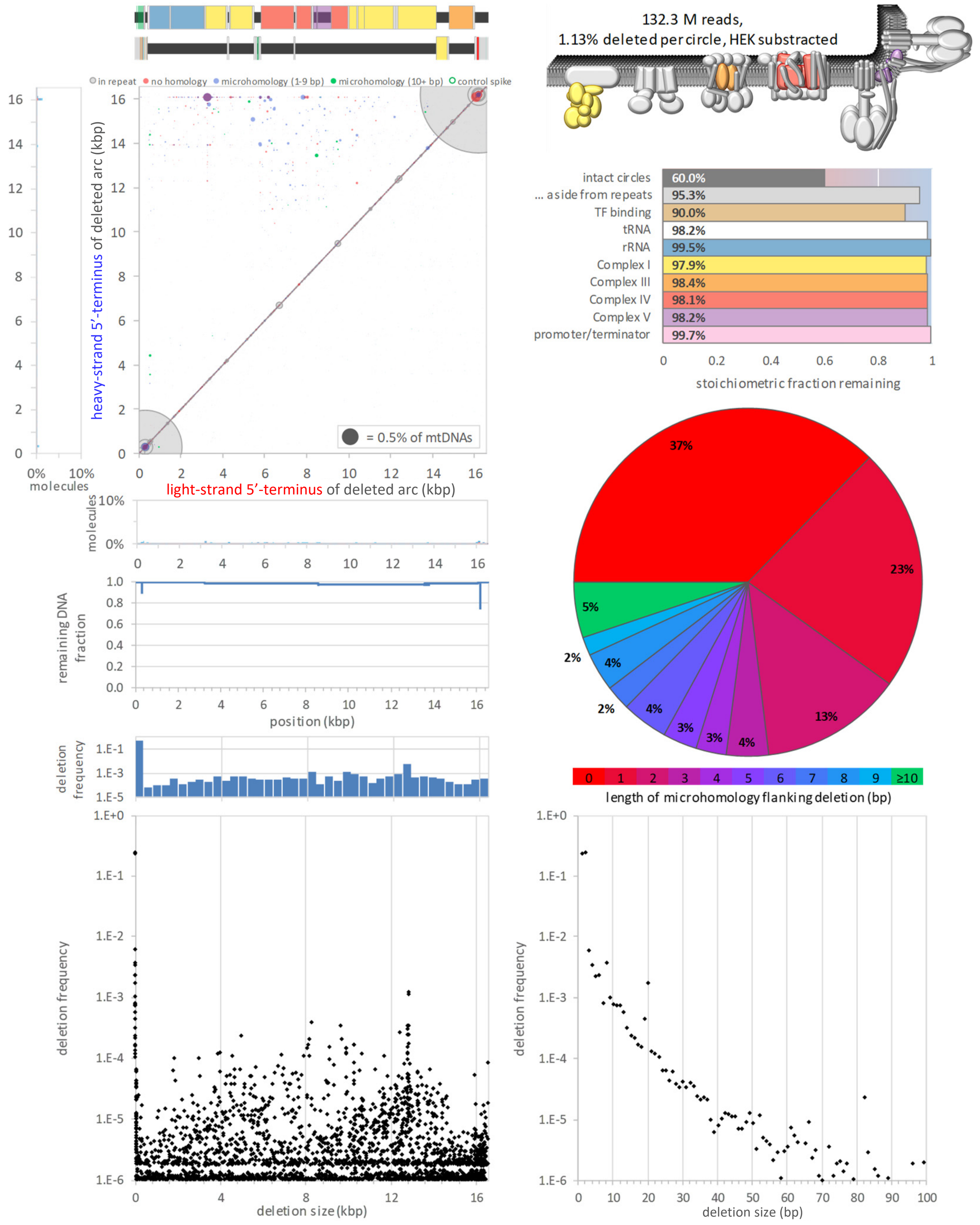

M19: *POLG* wild type, 93 years at biopsy

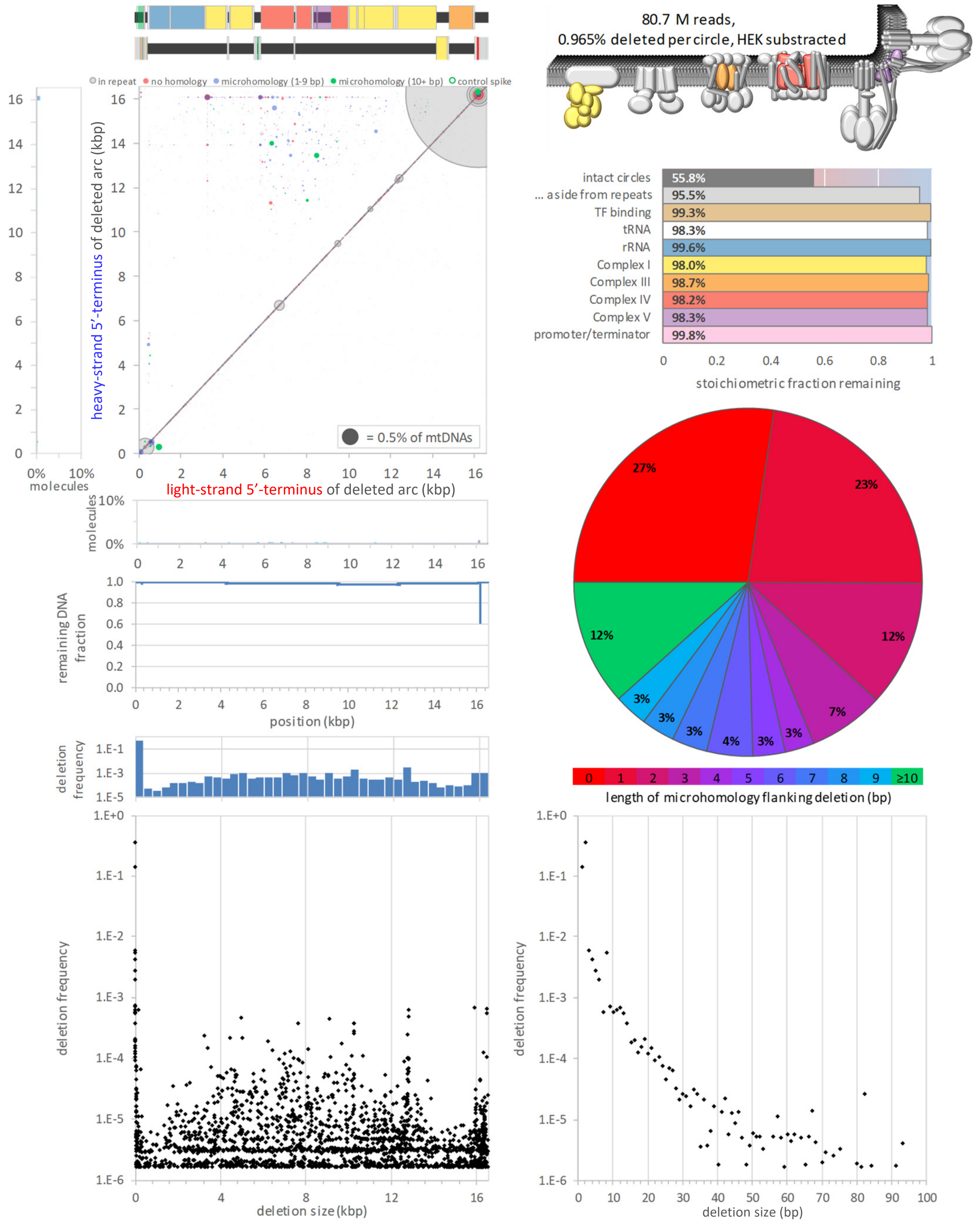

M20: 22 years at biopsy, *POLG* A467T;A467T

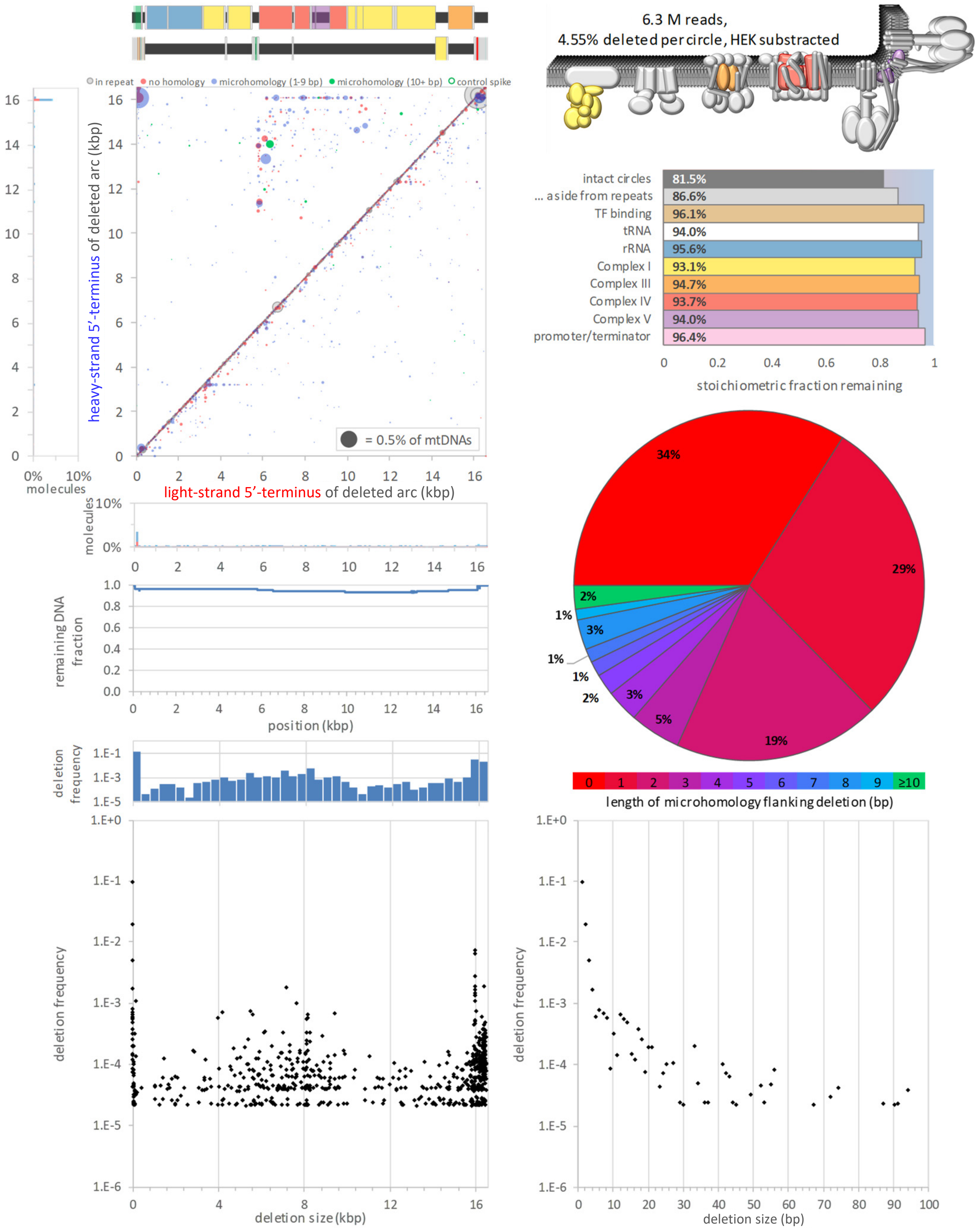

M21: 45 years at biopsy, *POLG* A467T;A467T

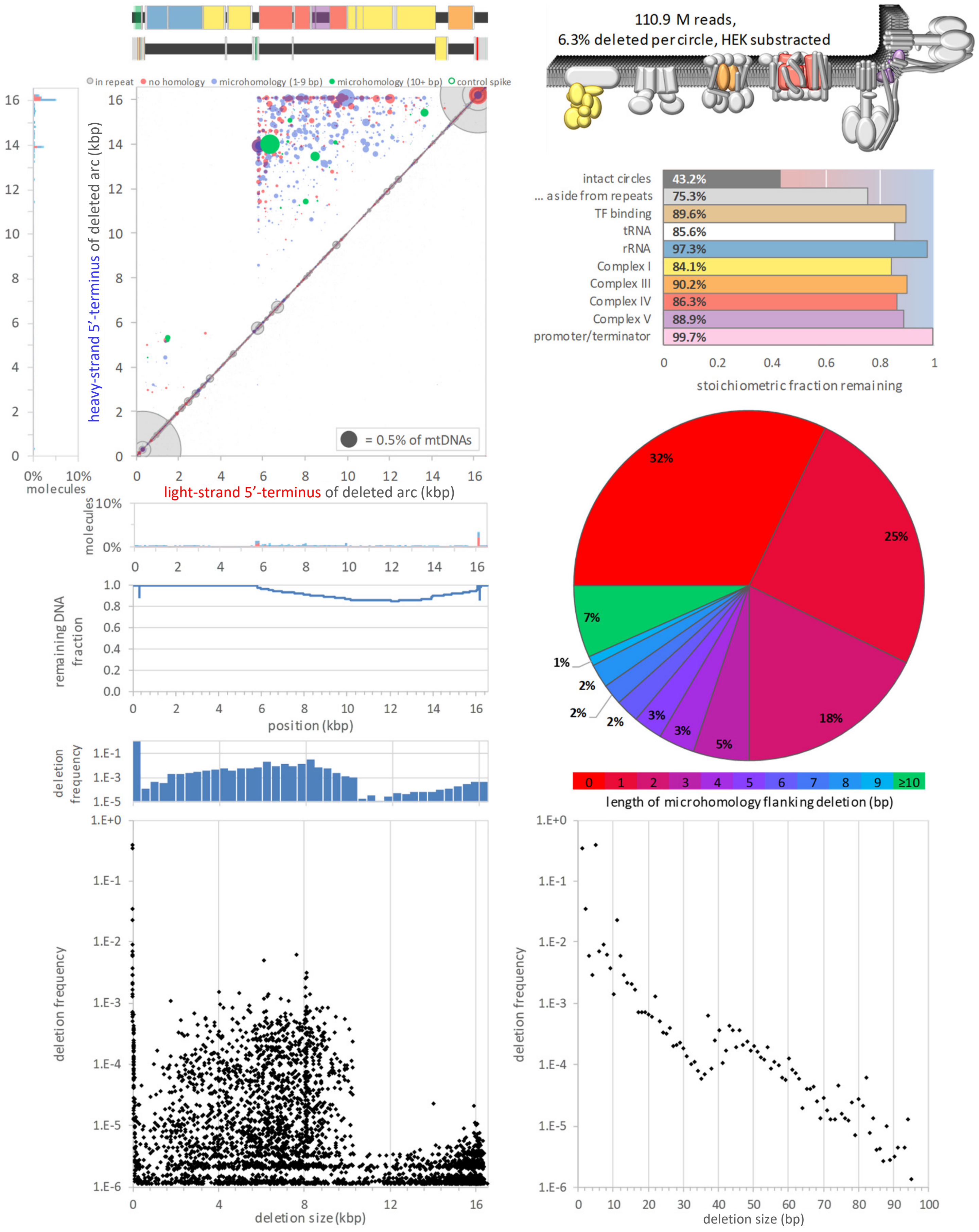

M22: 55 years at biopsy, *POLG* W748S;R1096C

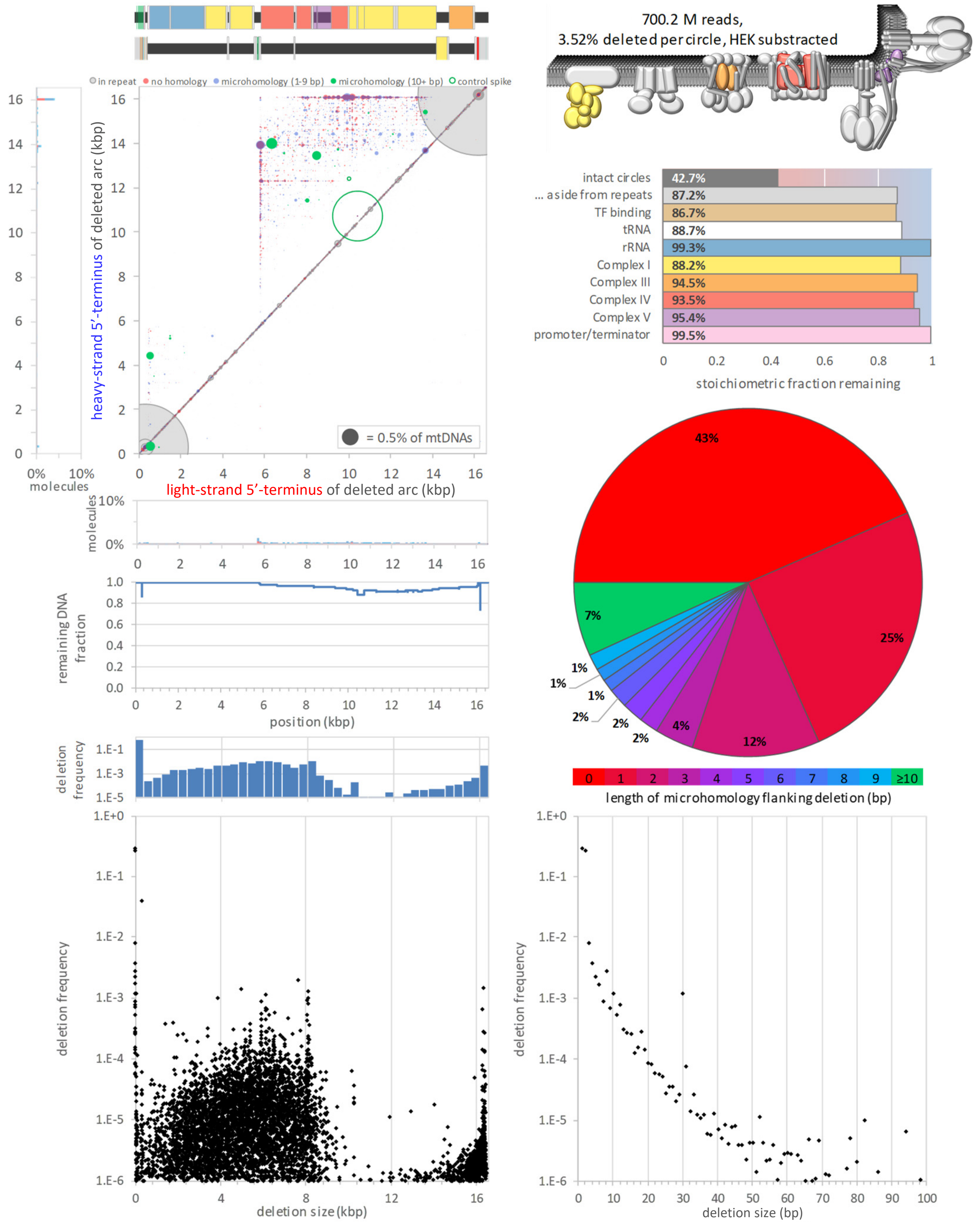

M23: 60 years at biopsy, *POLG* A467T;A467T

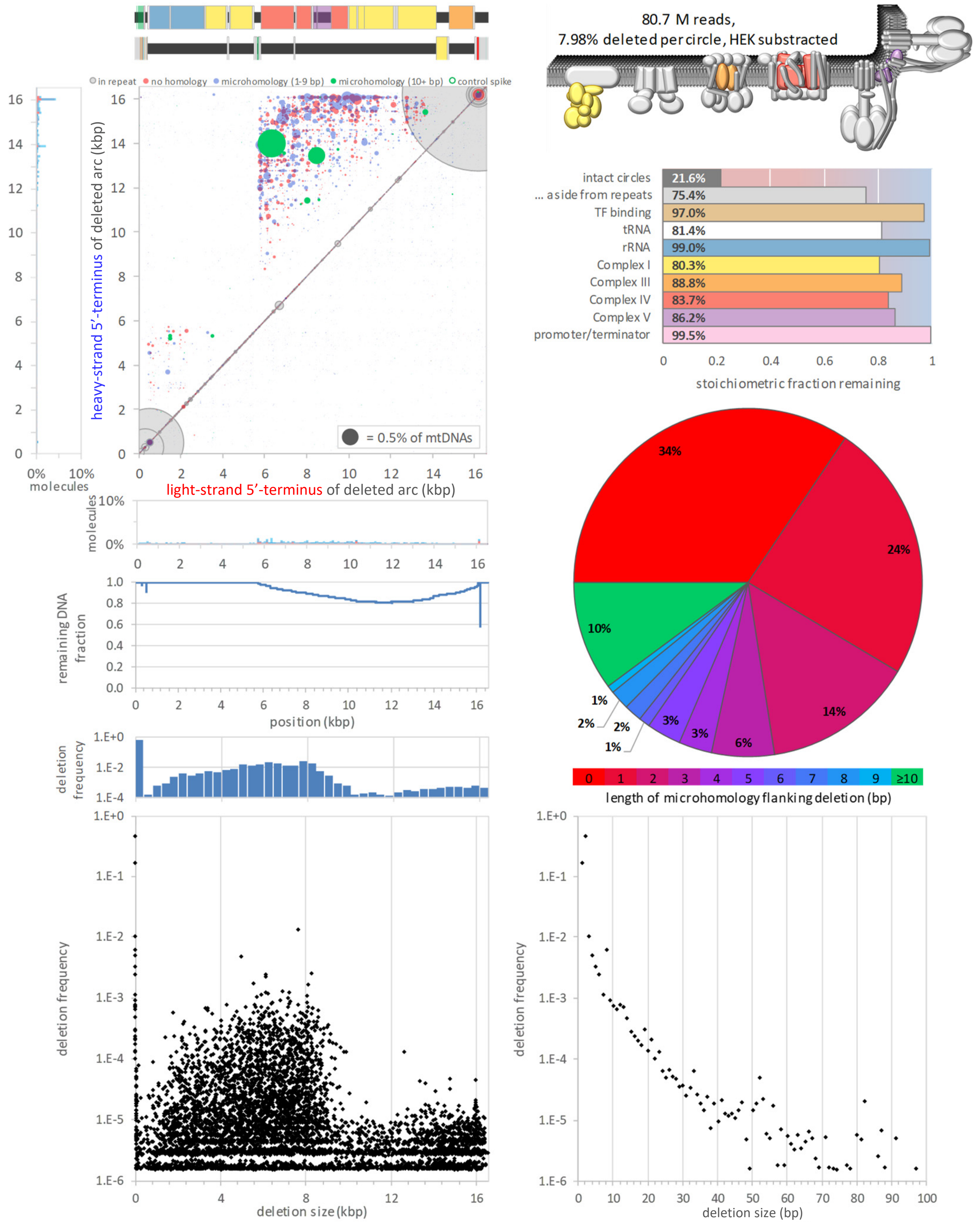

M24: 66 years at biopsy, *POLG* A467T;S933R

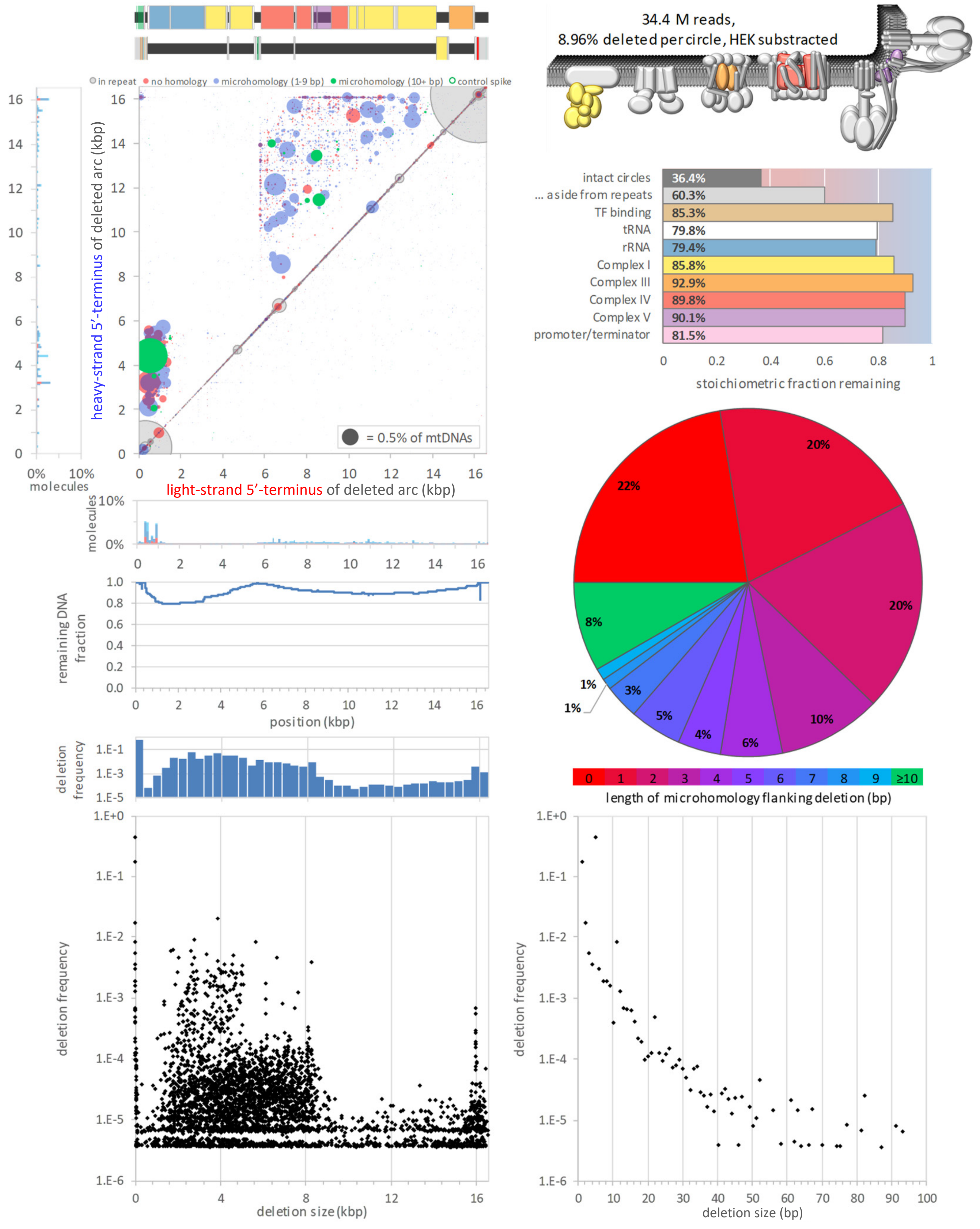

M25: 80 years at biopsy, *POLG* A467T;T251I/P587L

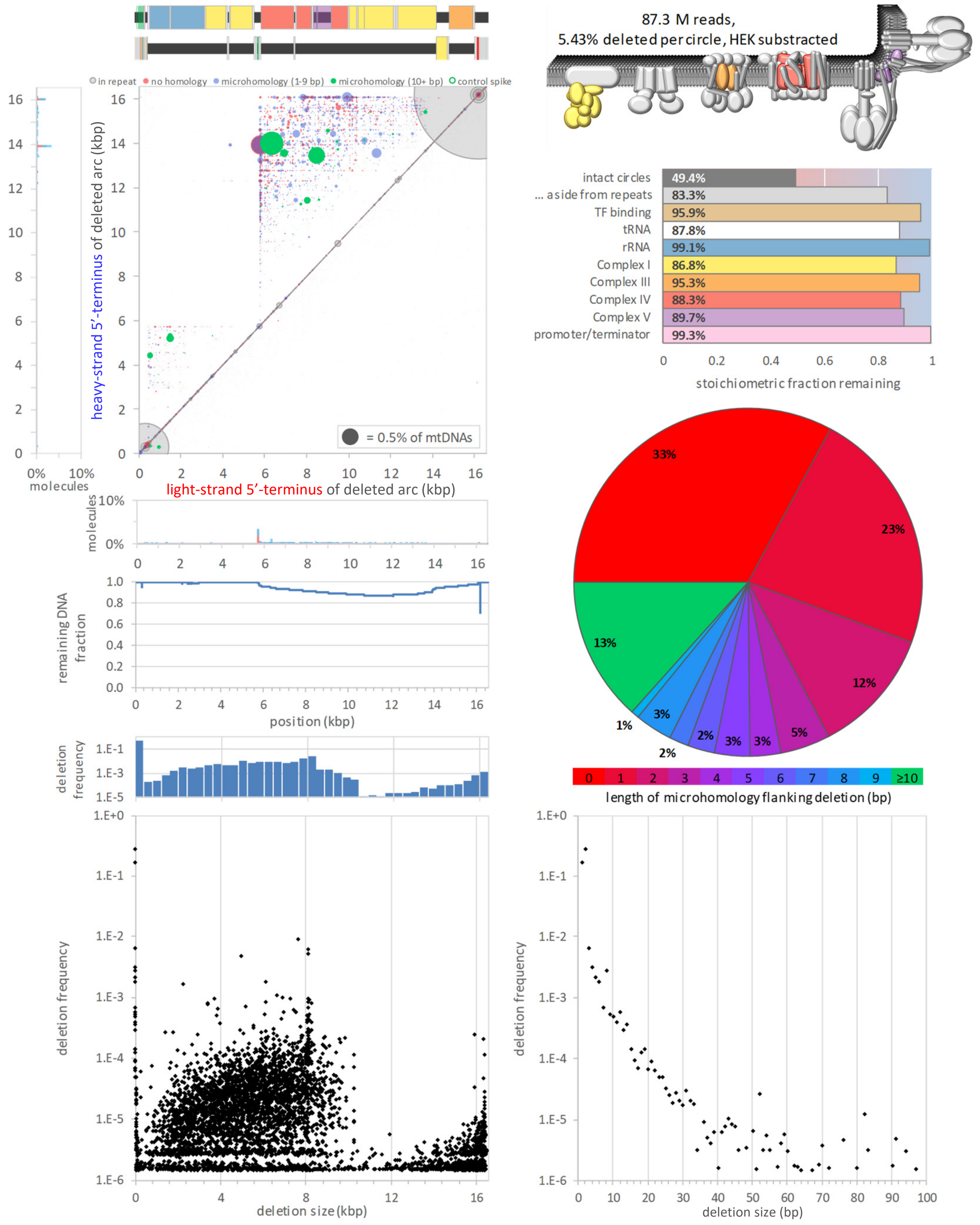

M26: 49 years at biopsy, *POLG* A467T;G737R

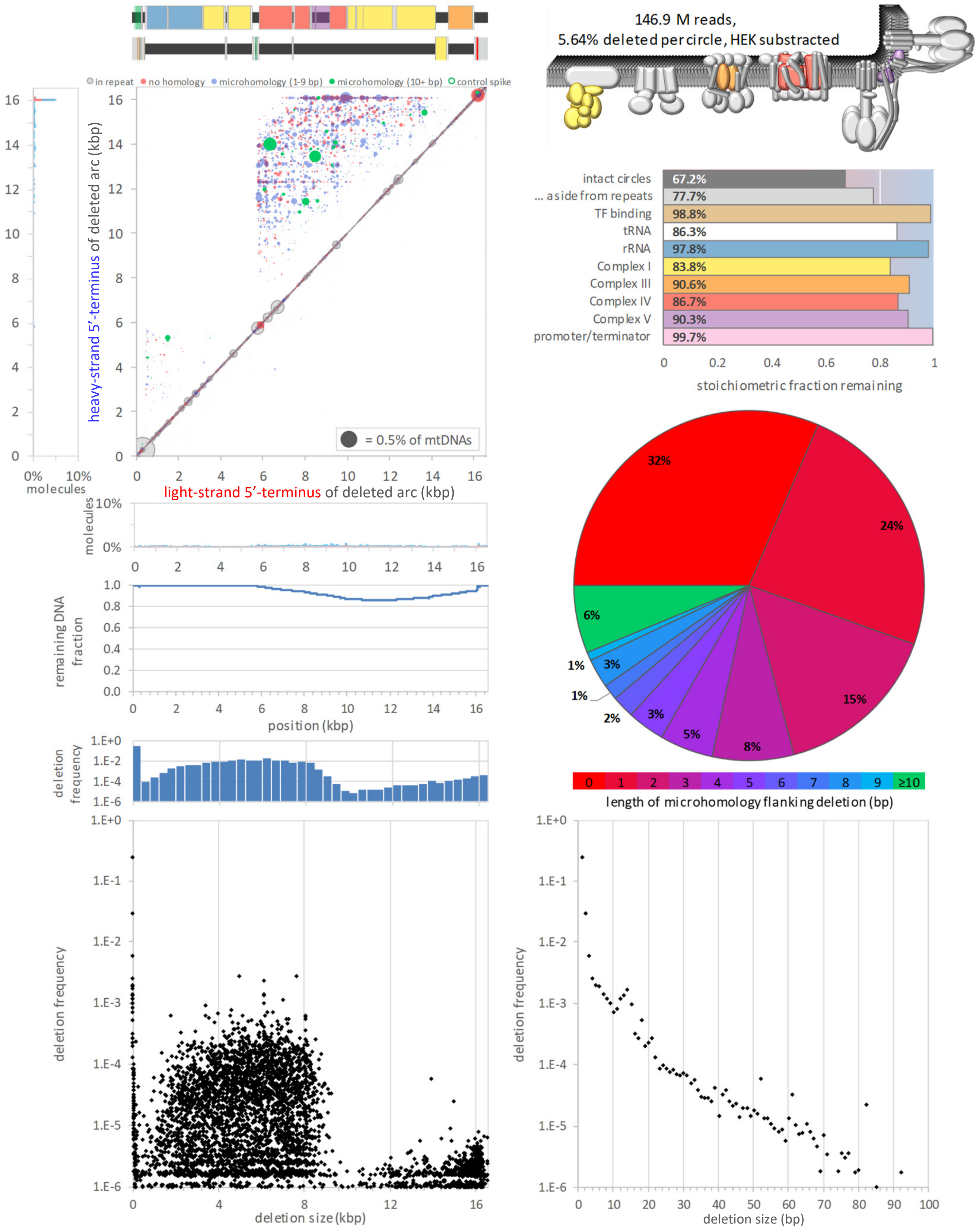

M27: 60 years, *POLG* A467T;p.X1240Gln+35aa

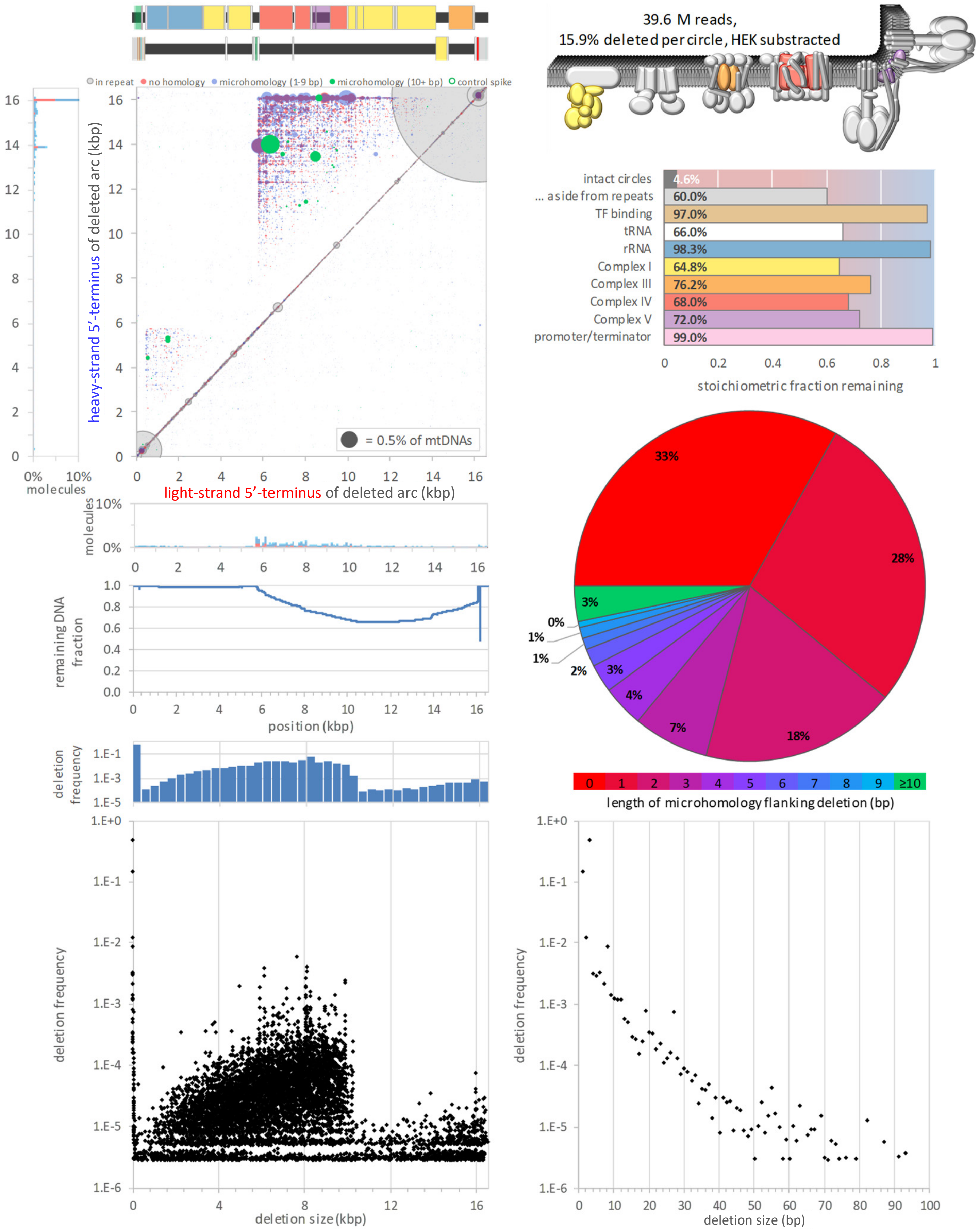

M28: 42 years at biopsy, *POLG* A467T;R1096C

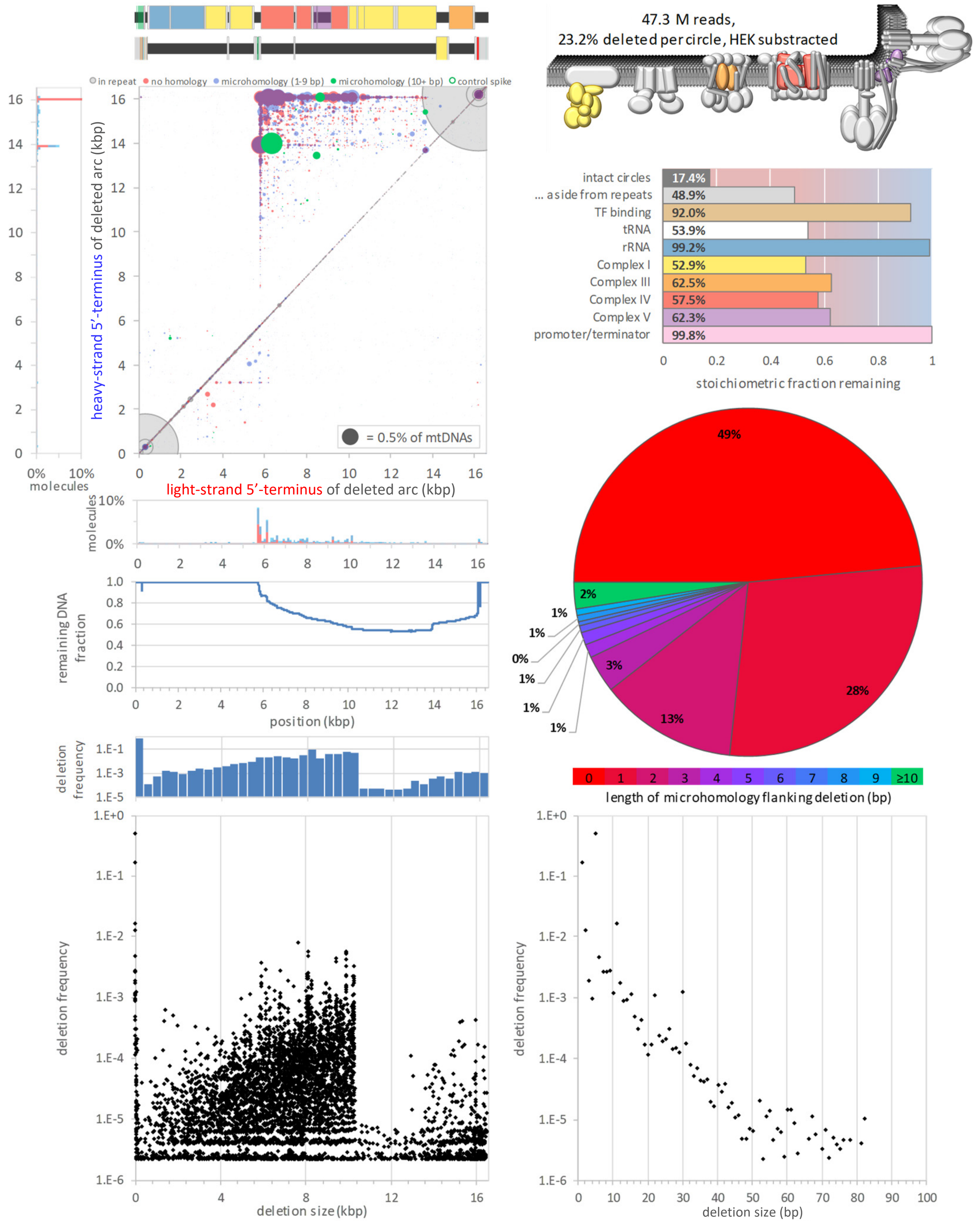

M29: 69 years at biopsy, *POLG* A467T;T251I/P587L

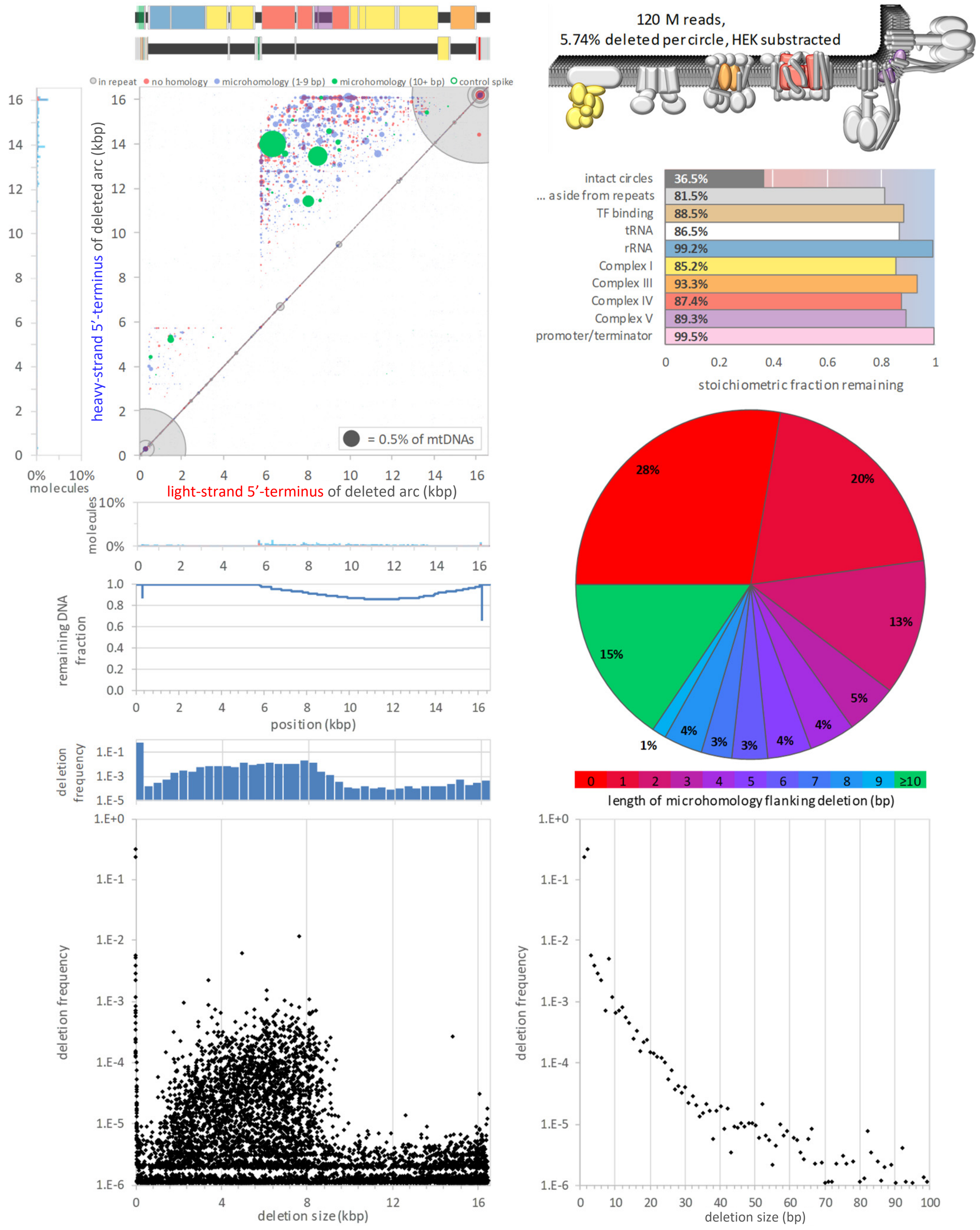

M30: 59 years at biopsy, *POLG* A467T;W748S

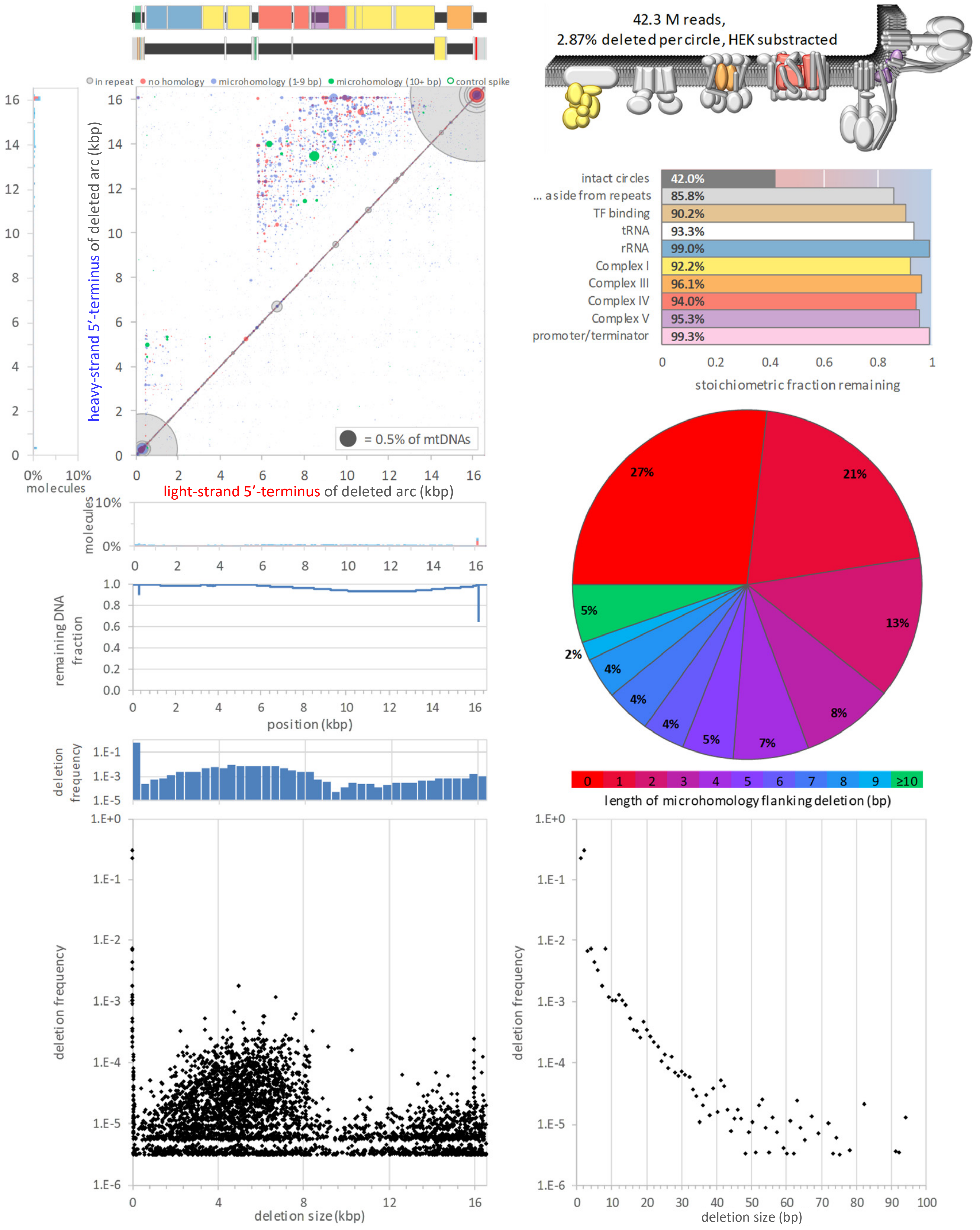

M31: 62 years at biopsy, *POLG* A467T;W748S

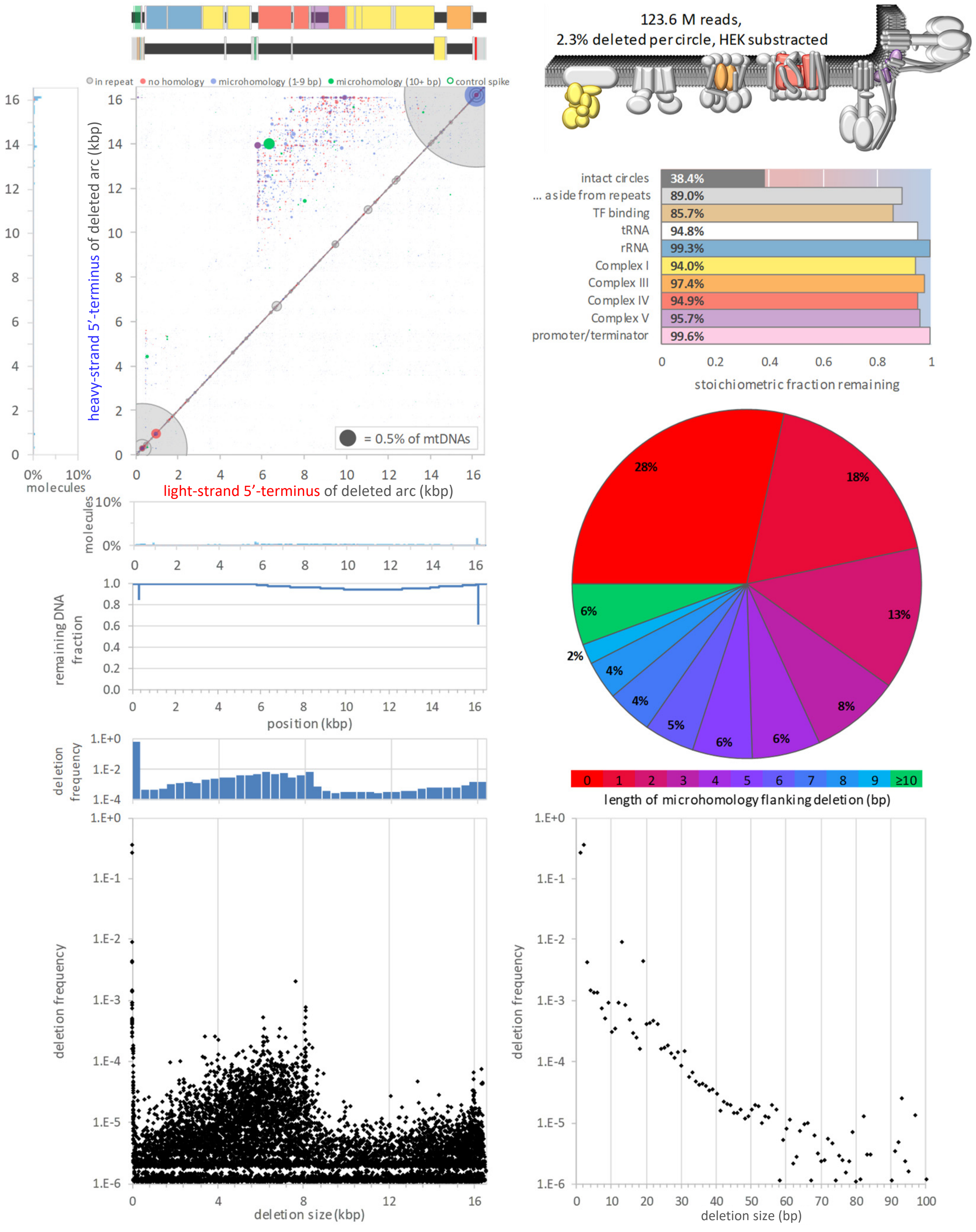

M32: 59 years at biopsy, *POLG* G848S;S1104C

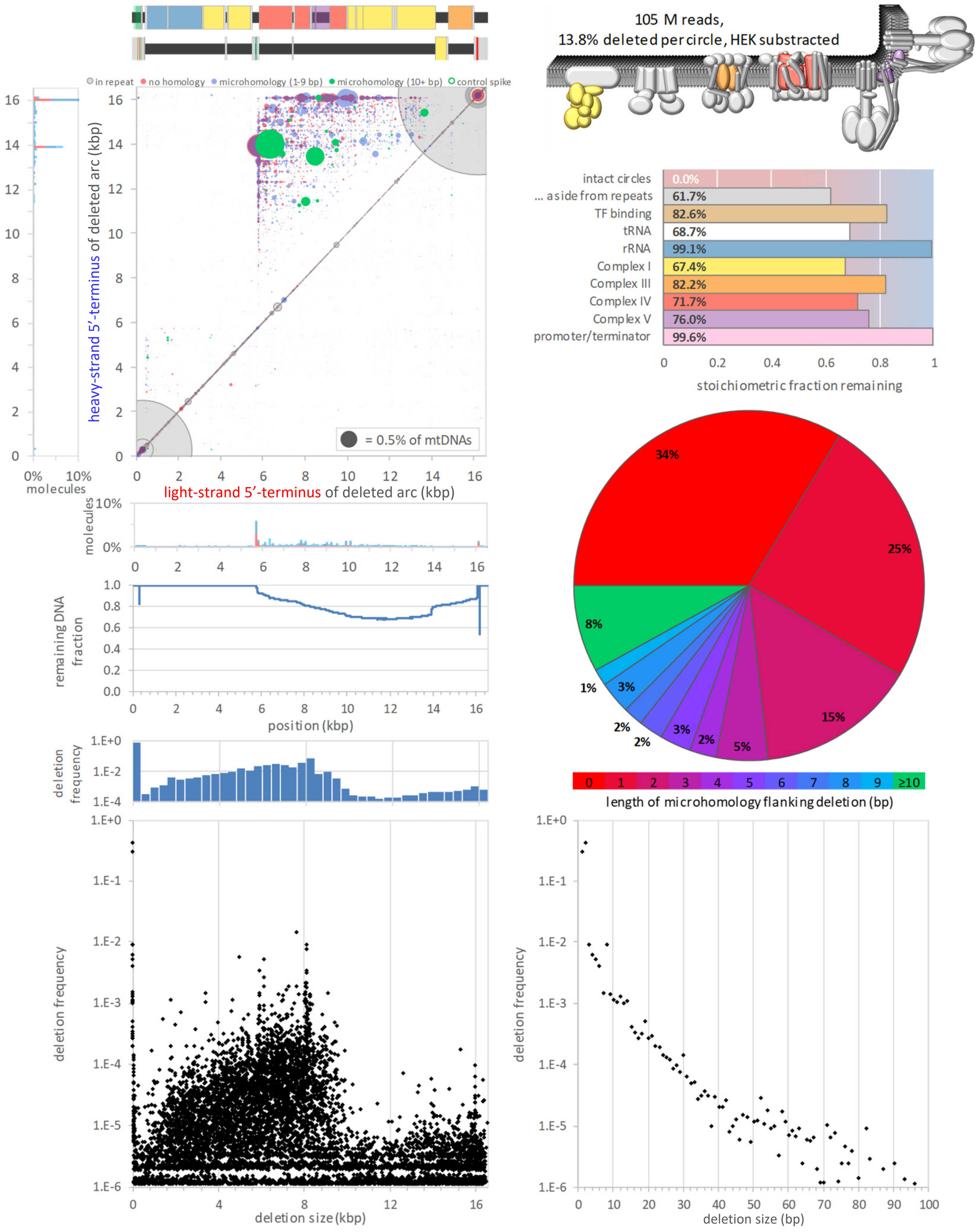

M33: 71 years at biopsy, *POLG* G952R

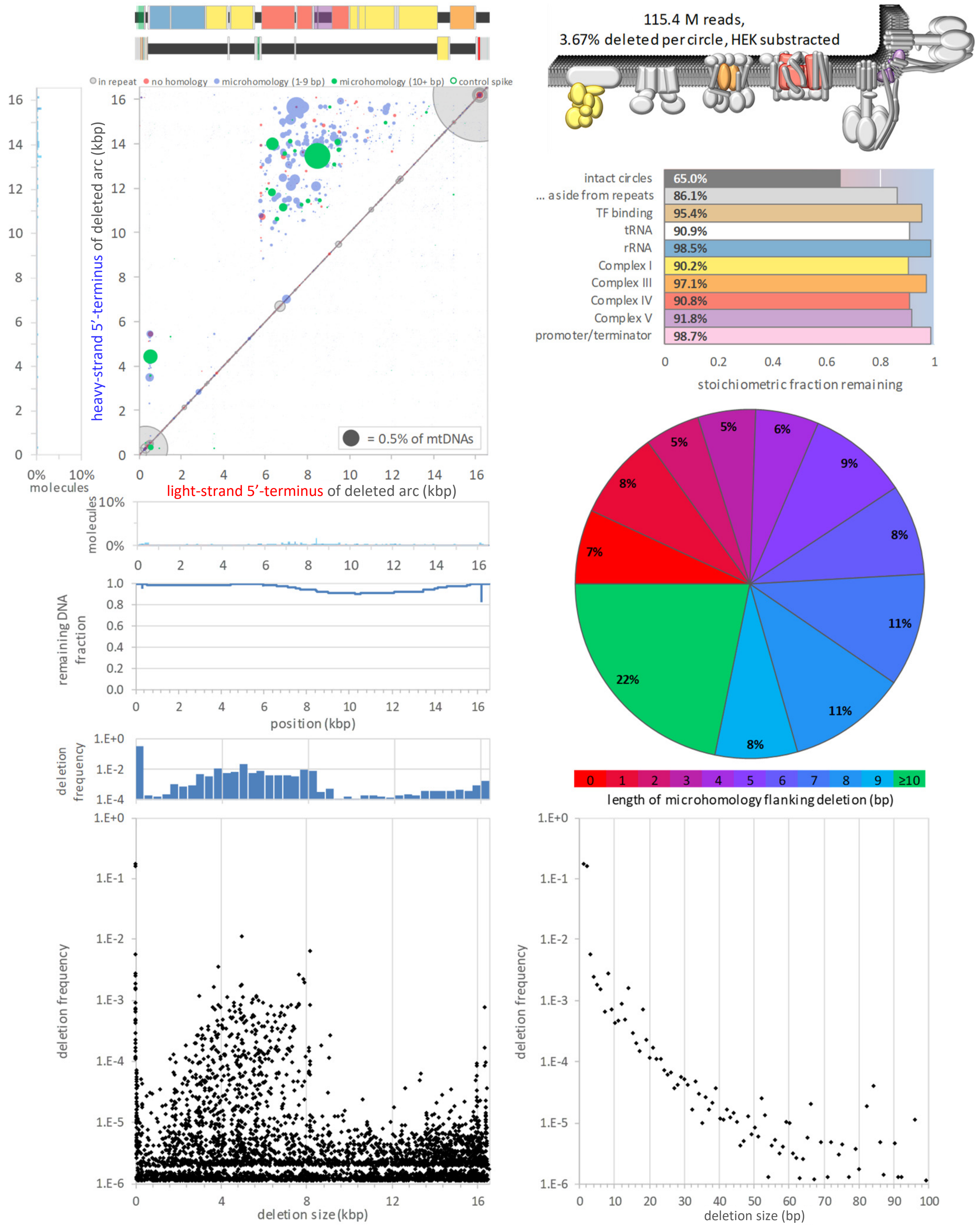

M34: 70 years at biopsy, *POLG* L411P;R574Q

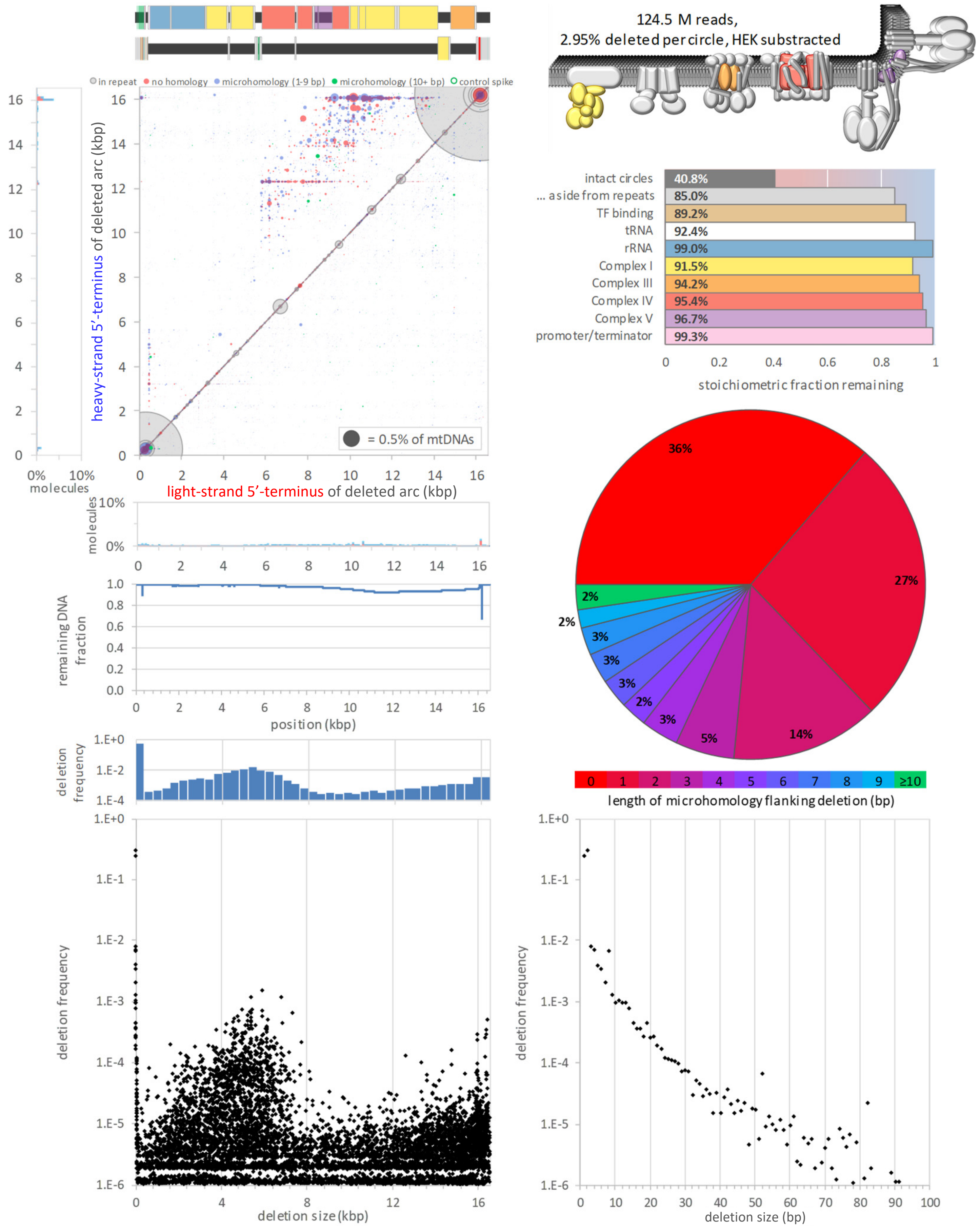

M35: 49 years at biopsy, *POLG* M797I;T251I/P587L

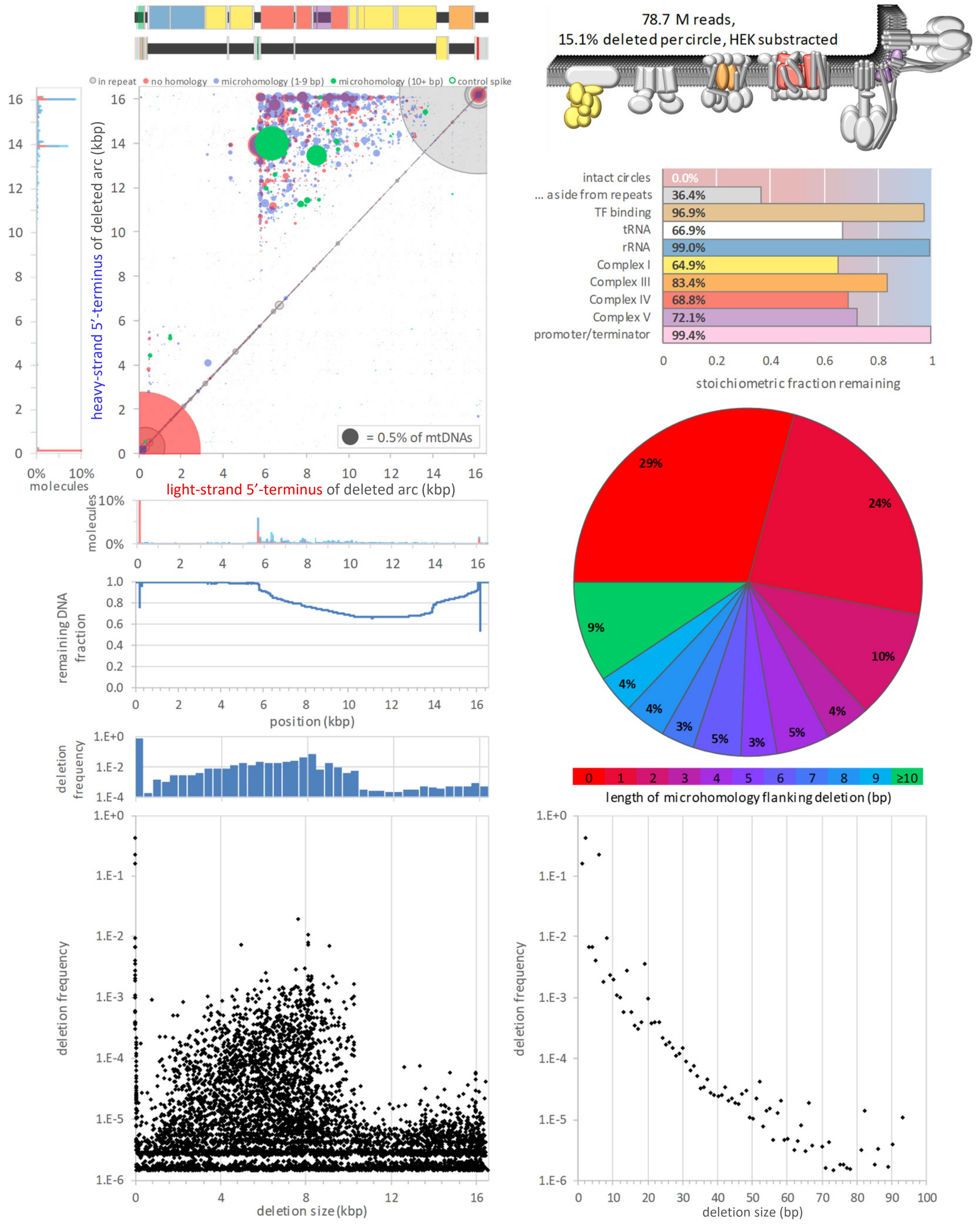

M36: 17 years at biopsy, *POLG* R597W;R597W

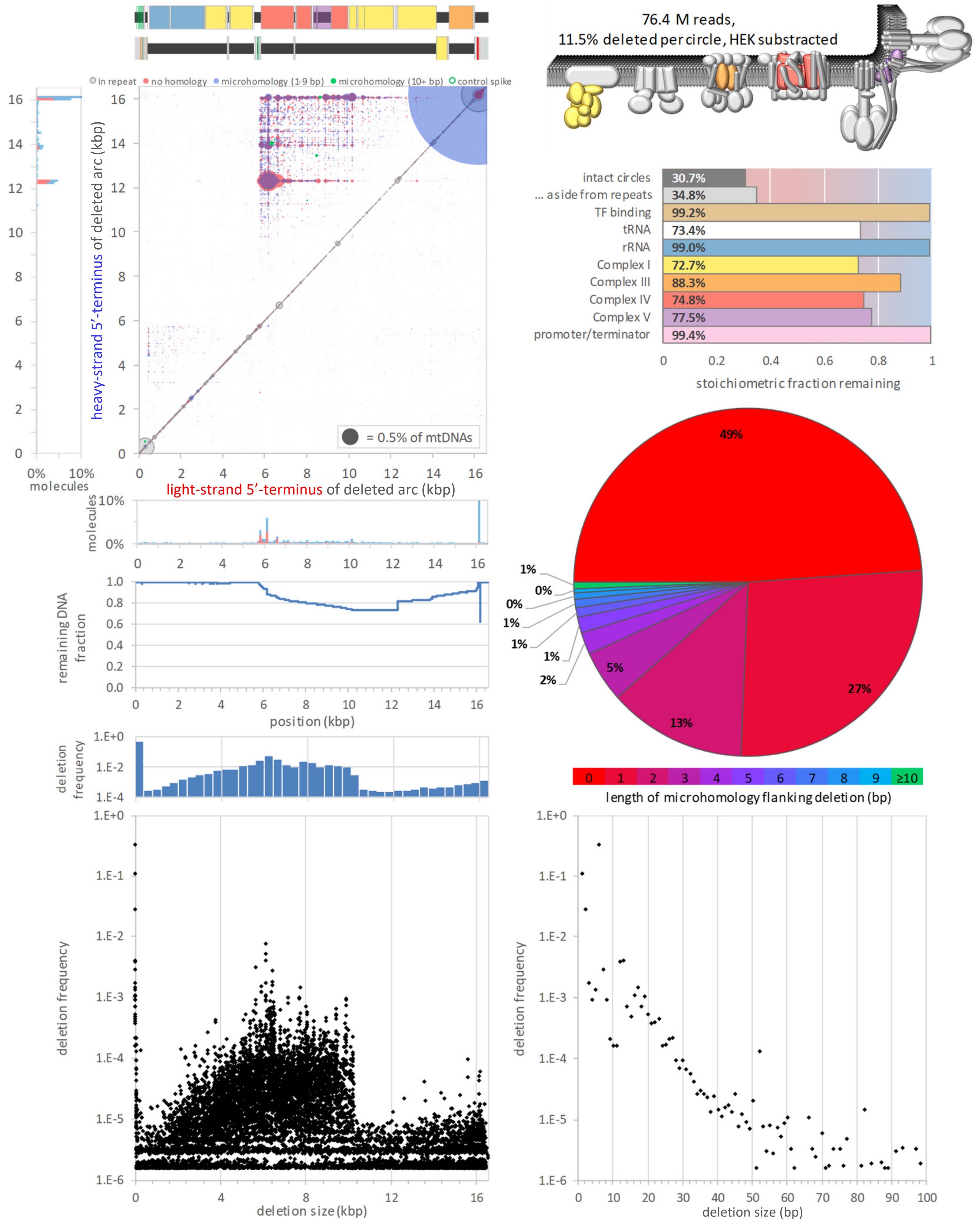

M37: 29 years at biopsy, *POLG* R627Q;G848S

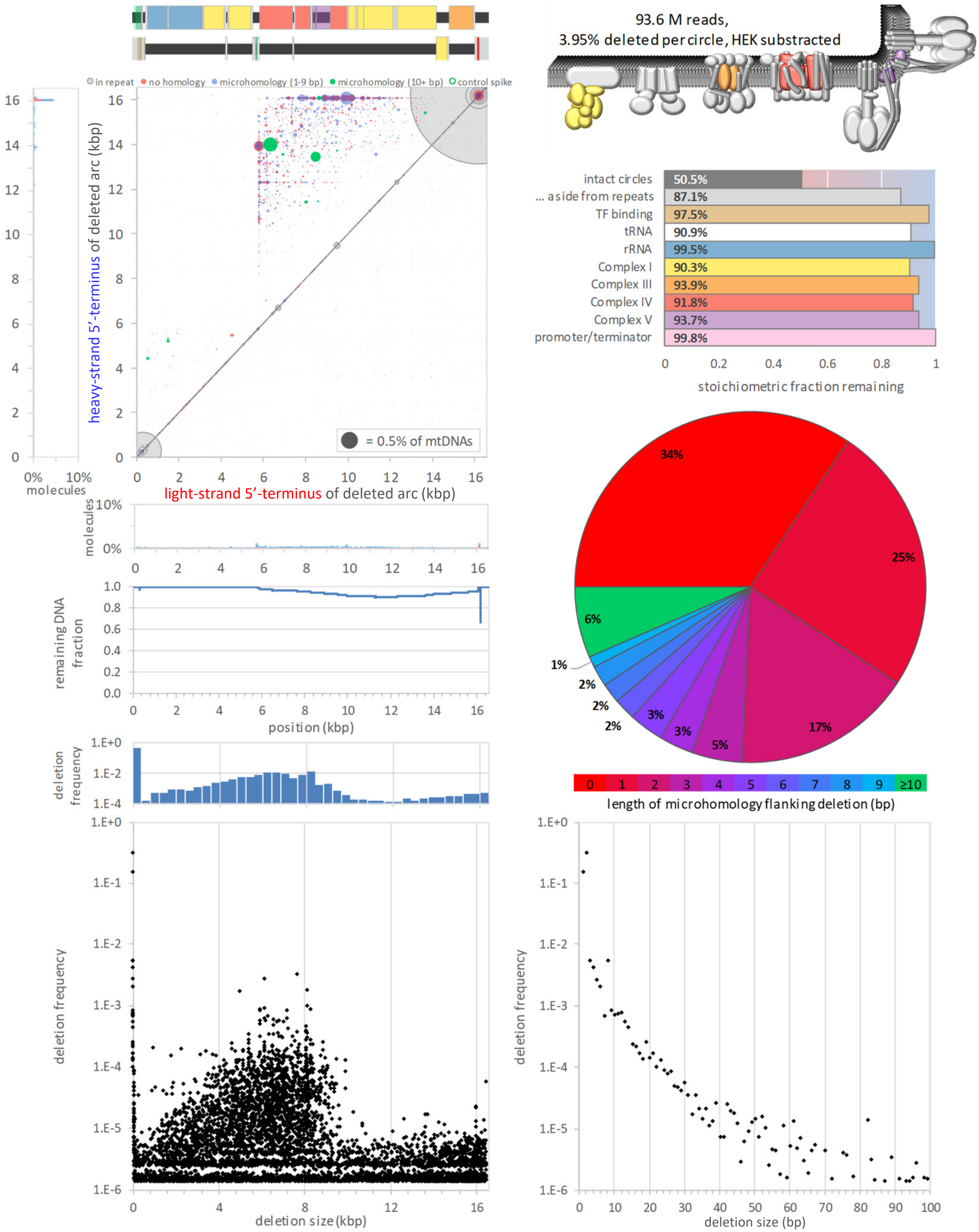

M38: 74 years at biopsy, *POLG* R852C;T251I/P587L

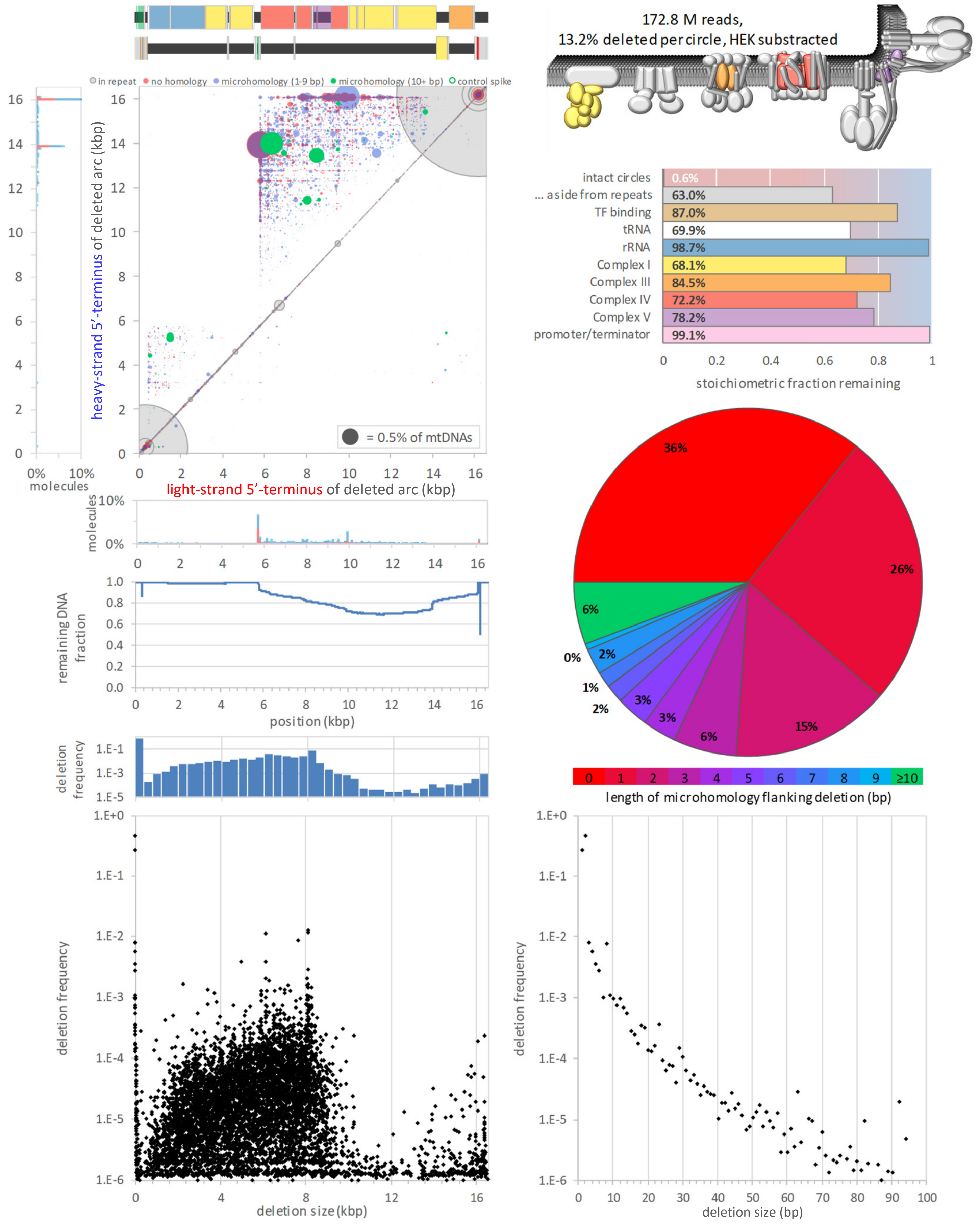

M39: 65 years at biopsy, *POLG* T914P;T251I/P587L

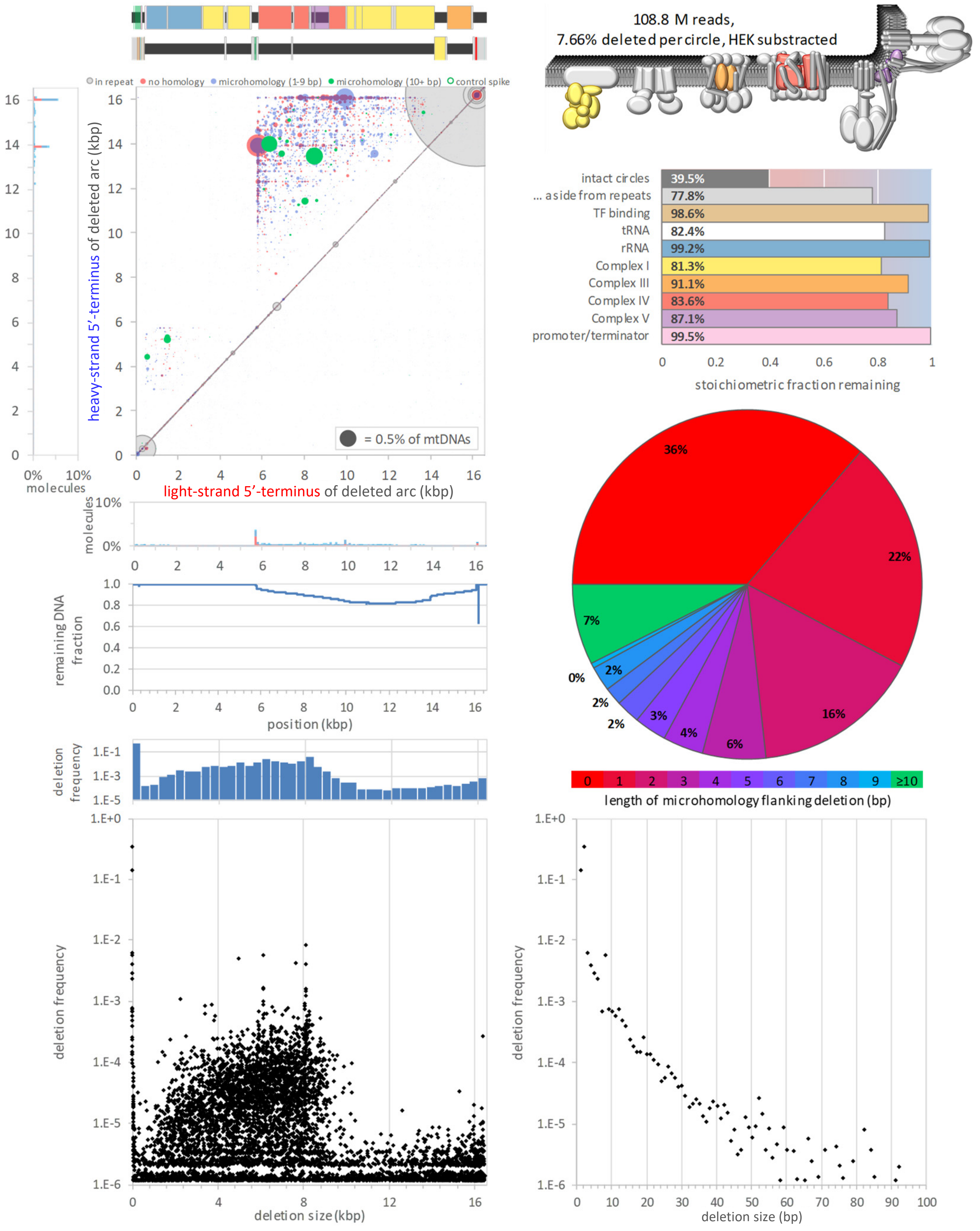

M40: 35 years at biopsy, *POLG* Y955C

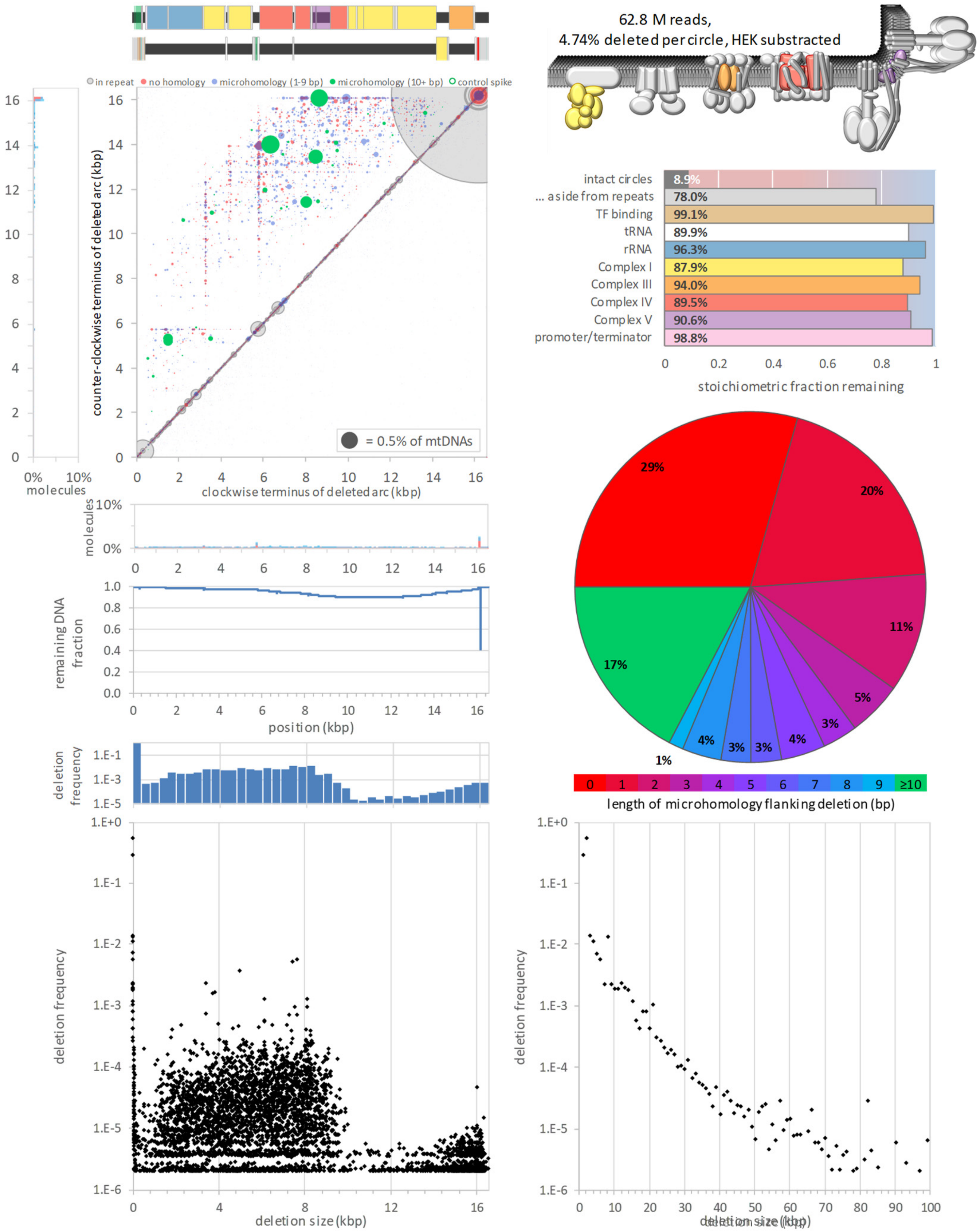

M41: 62 years at biopsy, *POLG* Y955C

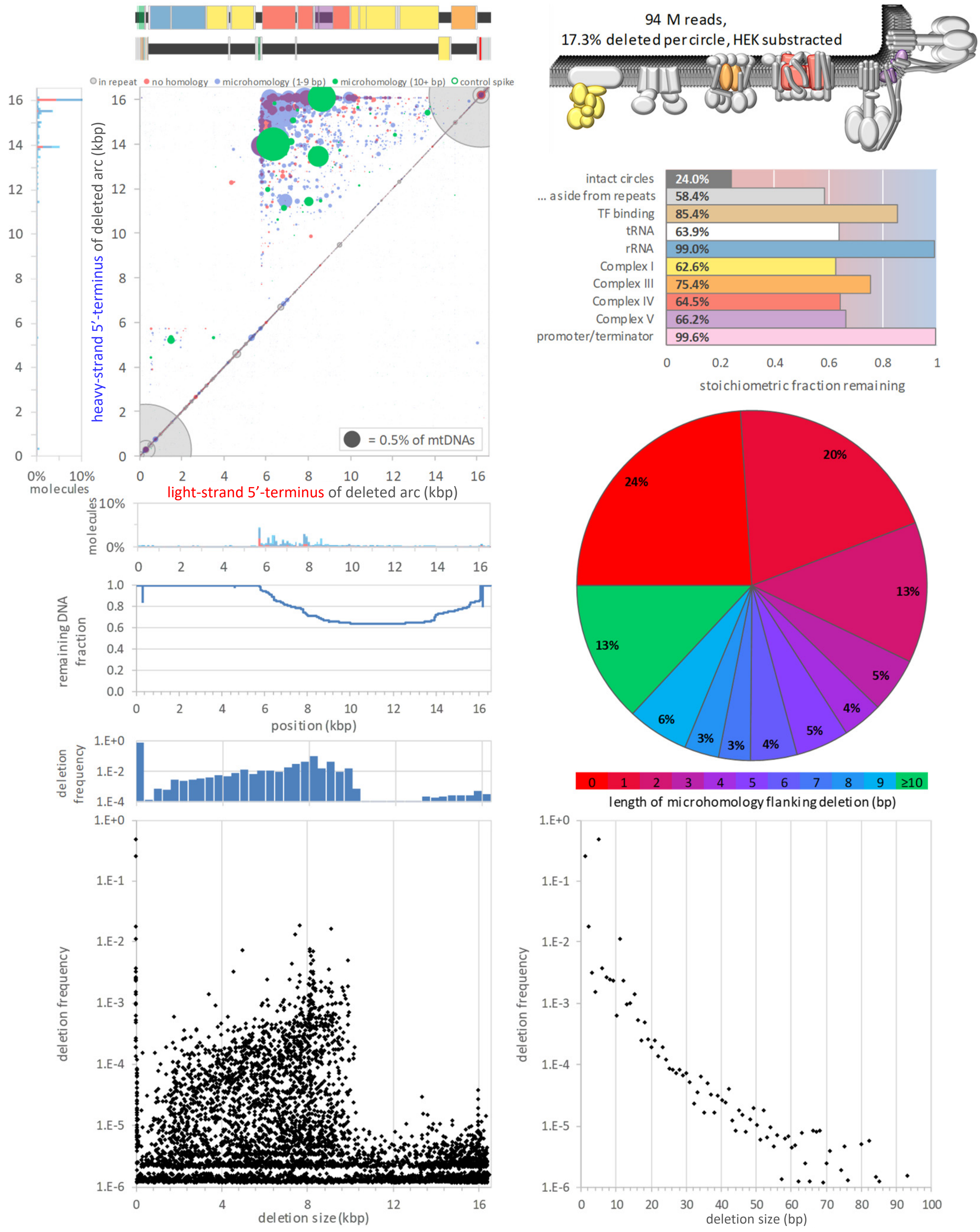

Supplement: Supplementary file 2 — Additional file 2. LostArc Reports. [file 13059_2020_2138_MOESM2_ESM.pdf]
