# Supplementary material for: Ultrasensitive deletion detection links mitochondrial DNA replication, disease, and aging
Source: Genome Biol. 2020 Sep 17;21:248. doi: 10.1186/s13059-020-02138-5 (PMC7500033; doi:10.1186/s13059-020-02138-5)

Sample	Gender	Age biopsy	Age onset	POLG genotype
M20	F	22	21	A467T;A467T

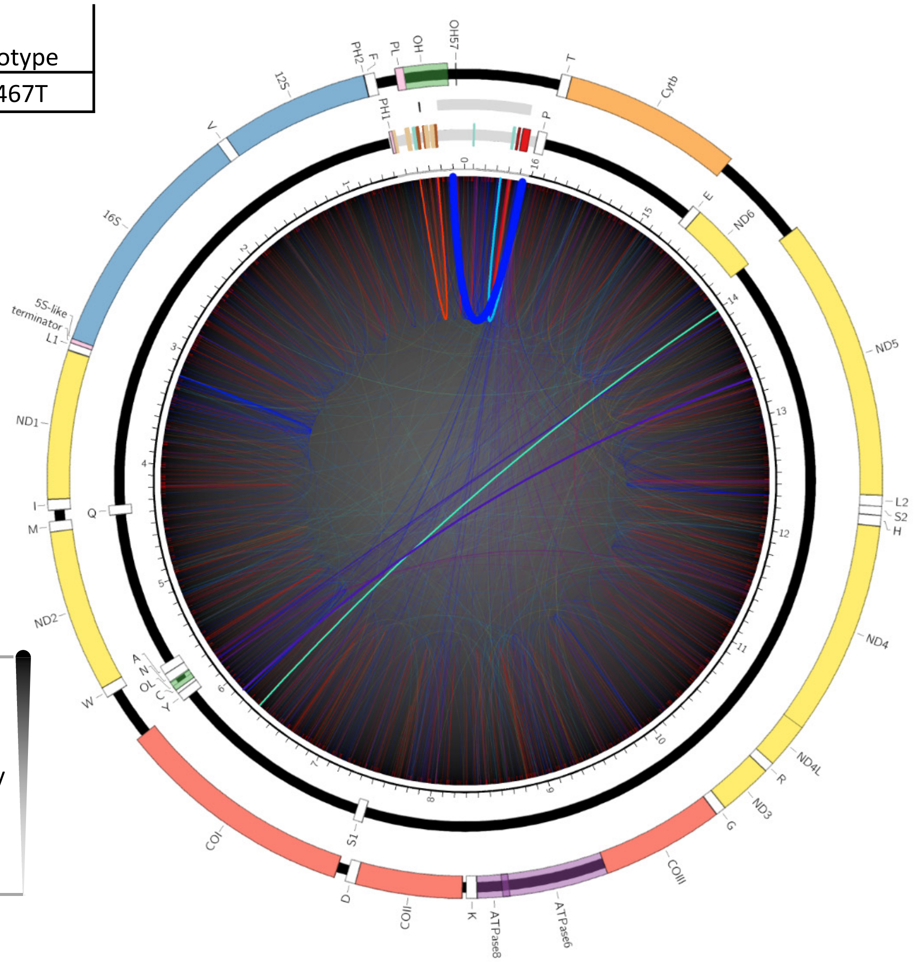

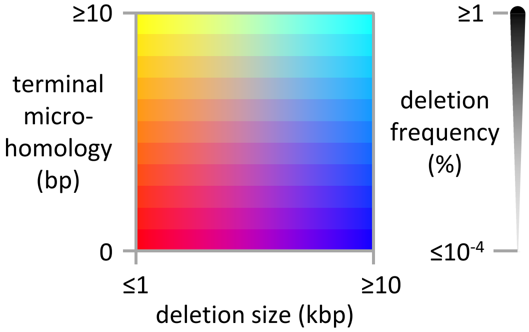

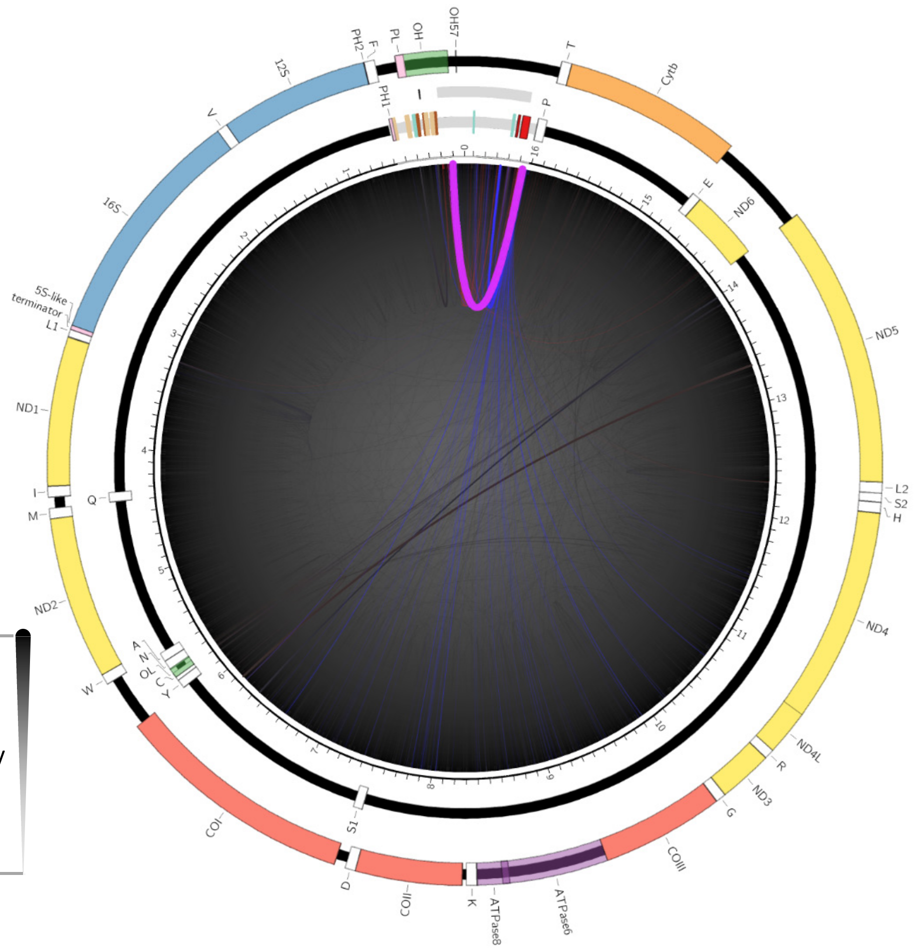

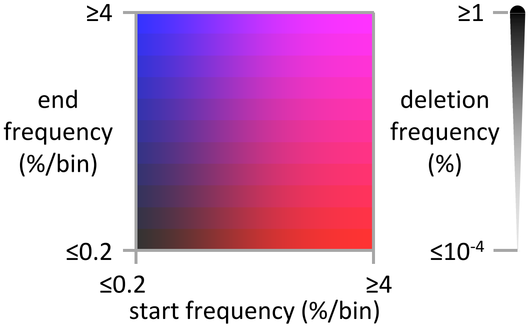

Sample	Gender	Age biopsy	Age onset	POLG genotype
M21	F	45	30	A467T;A467T

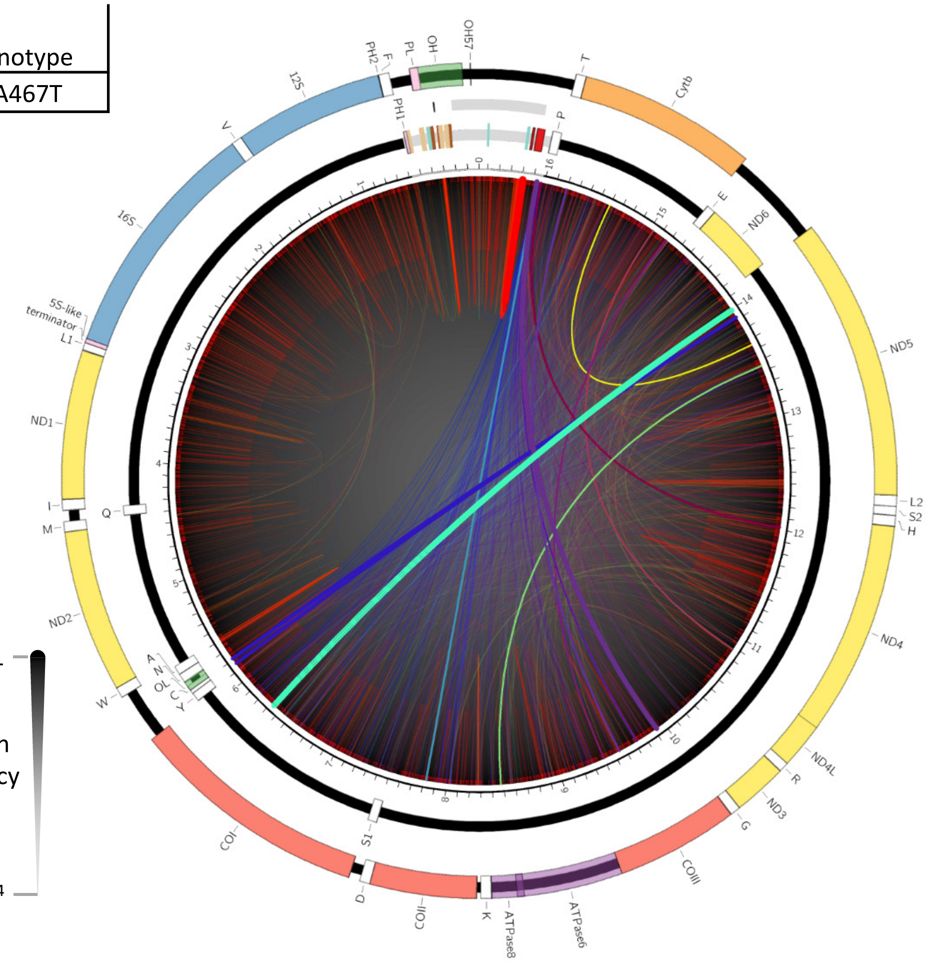

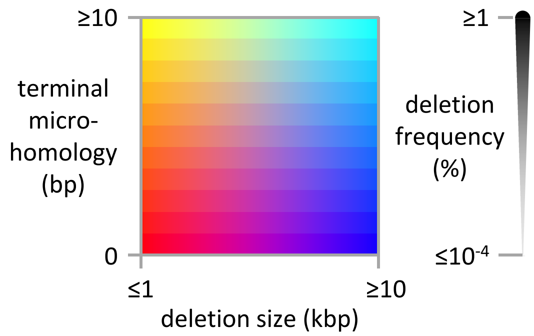

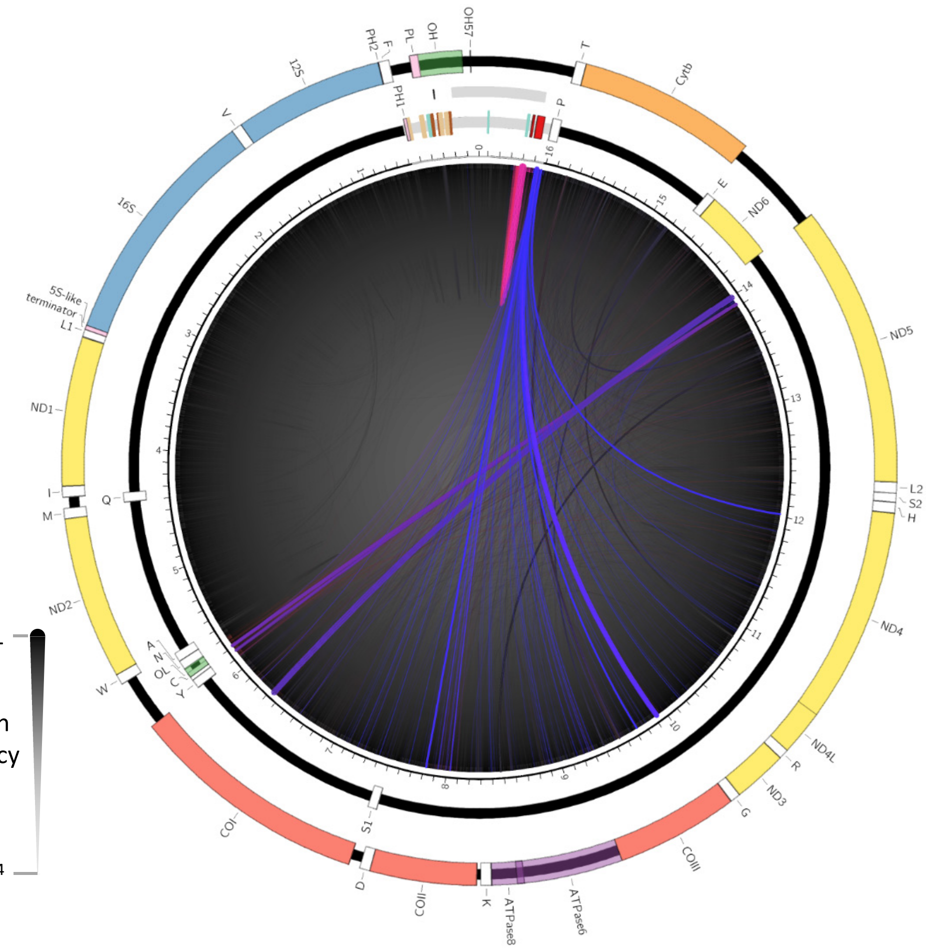

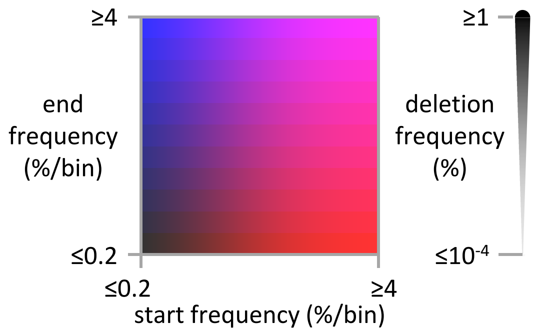

Sample	Gender	Age biopsy	Age onset	POLG genotype
M22	M	55	25	W748S;R1096C

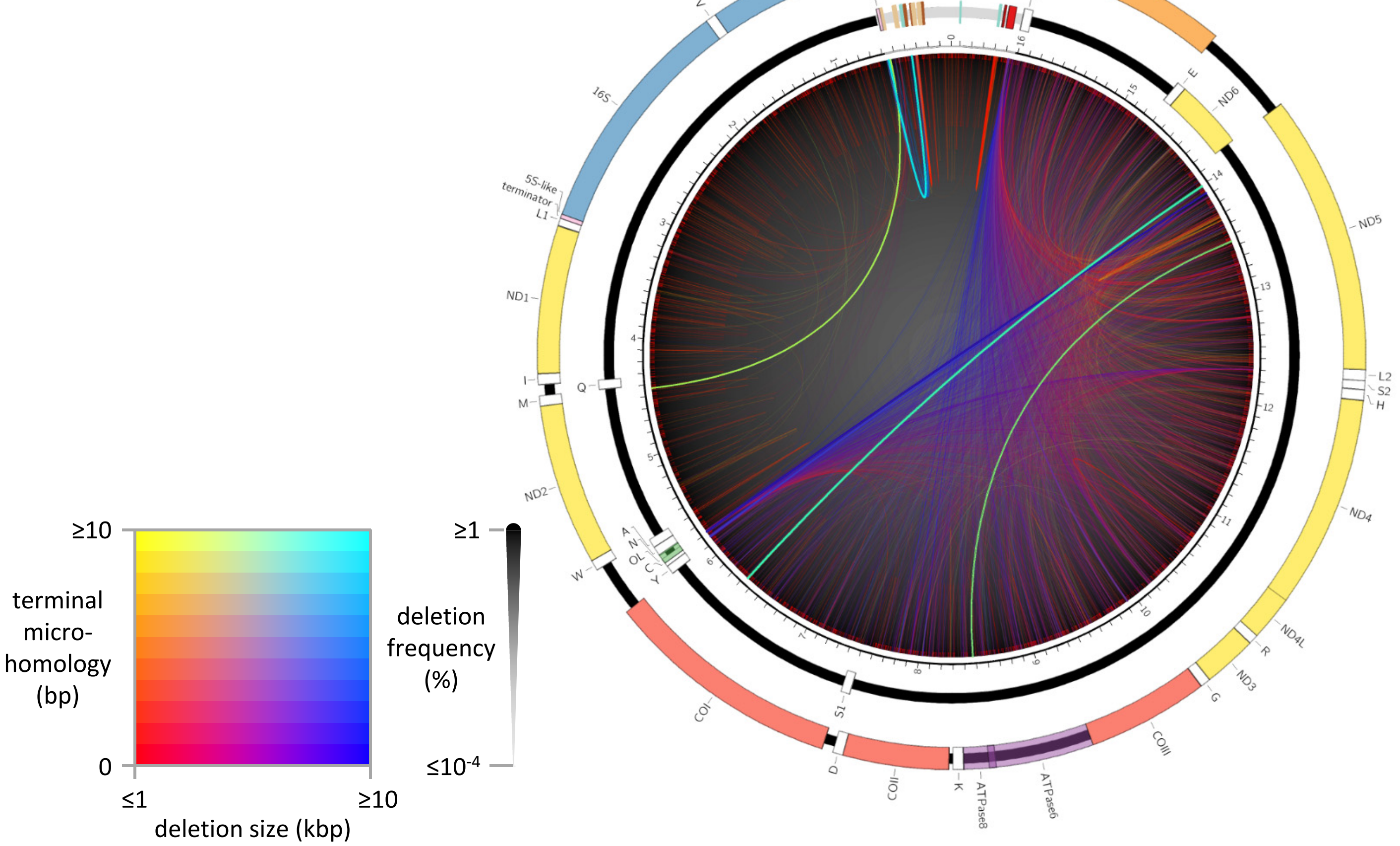

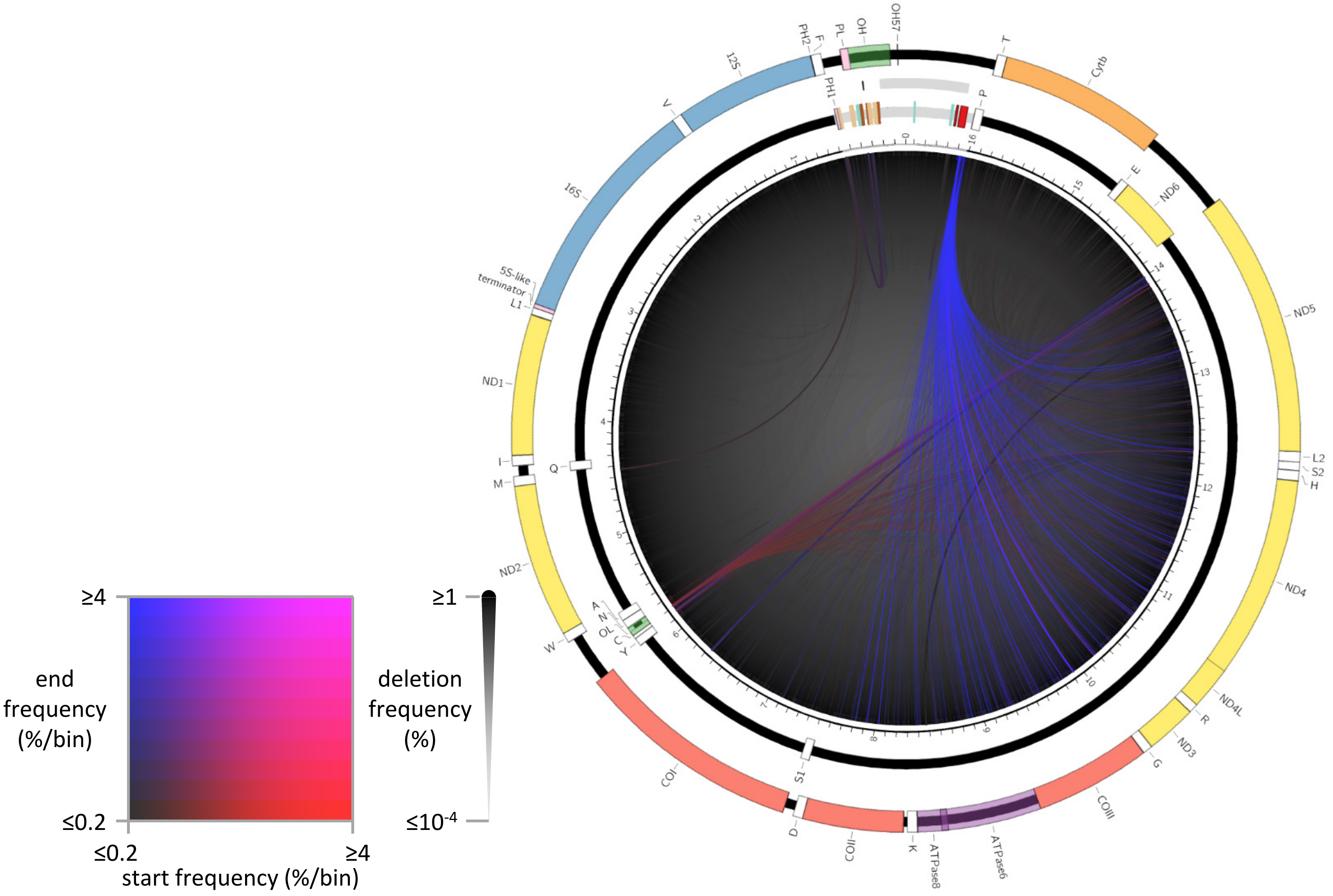

Sample	Gender	Age biopsy	Age onset	POLG genotype
M23	M	60	18	A467T;A467T

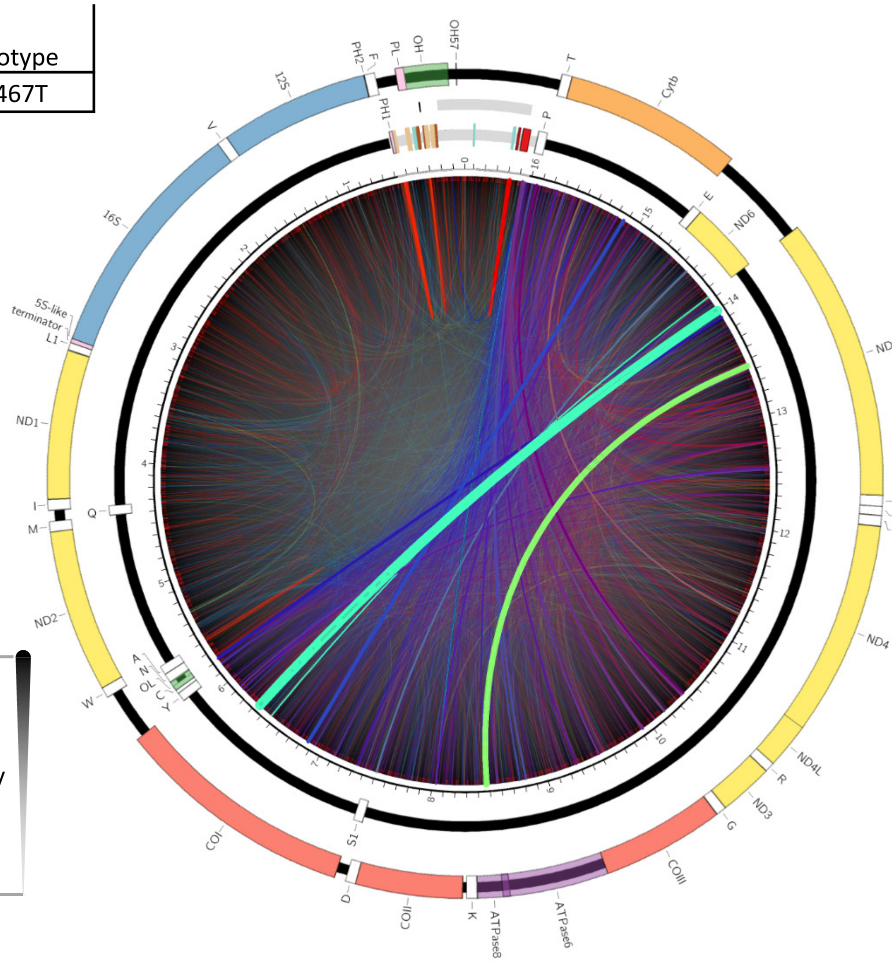

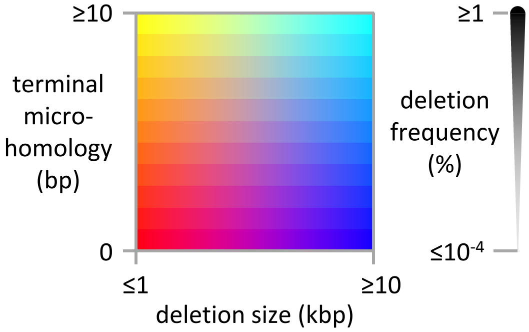

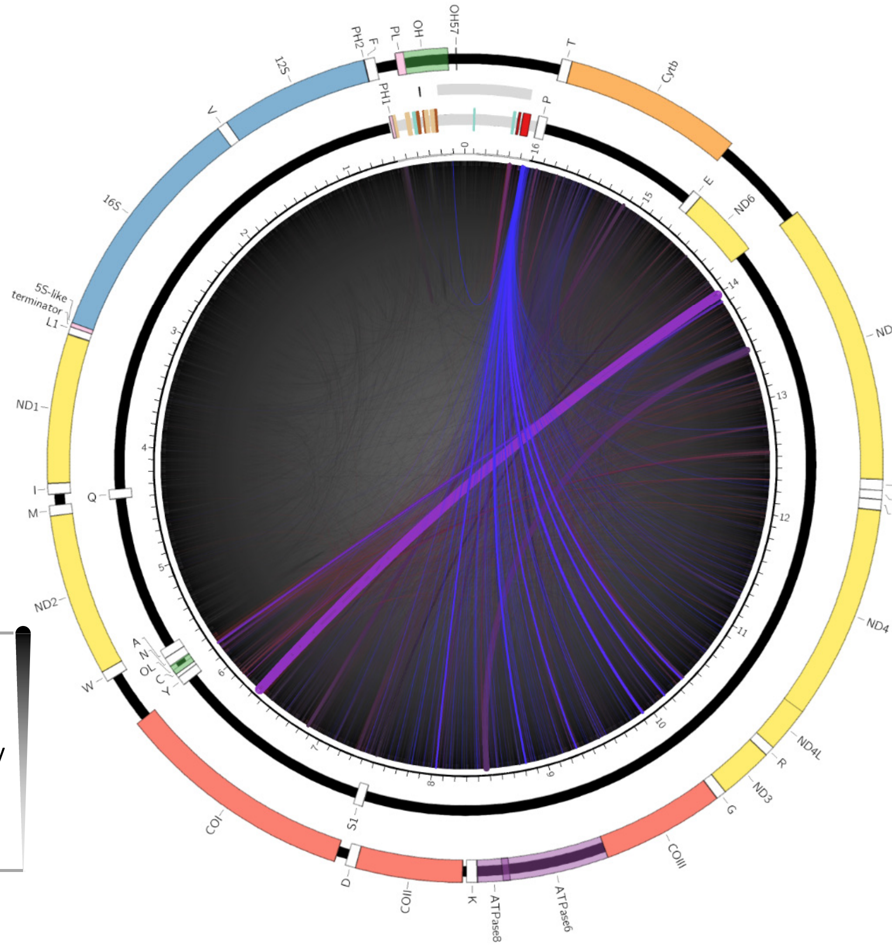

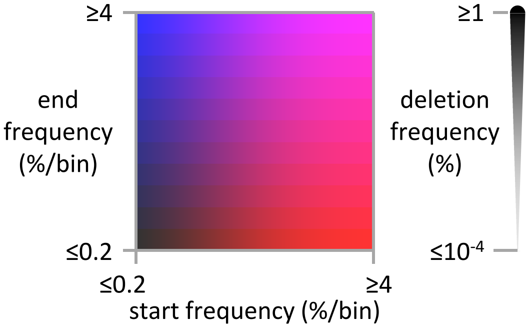

Sample	Gender	Age biopsy	Age onset	POLG genotype
M24	F	66	60	A467T;S933R

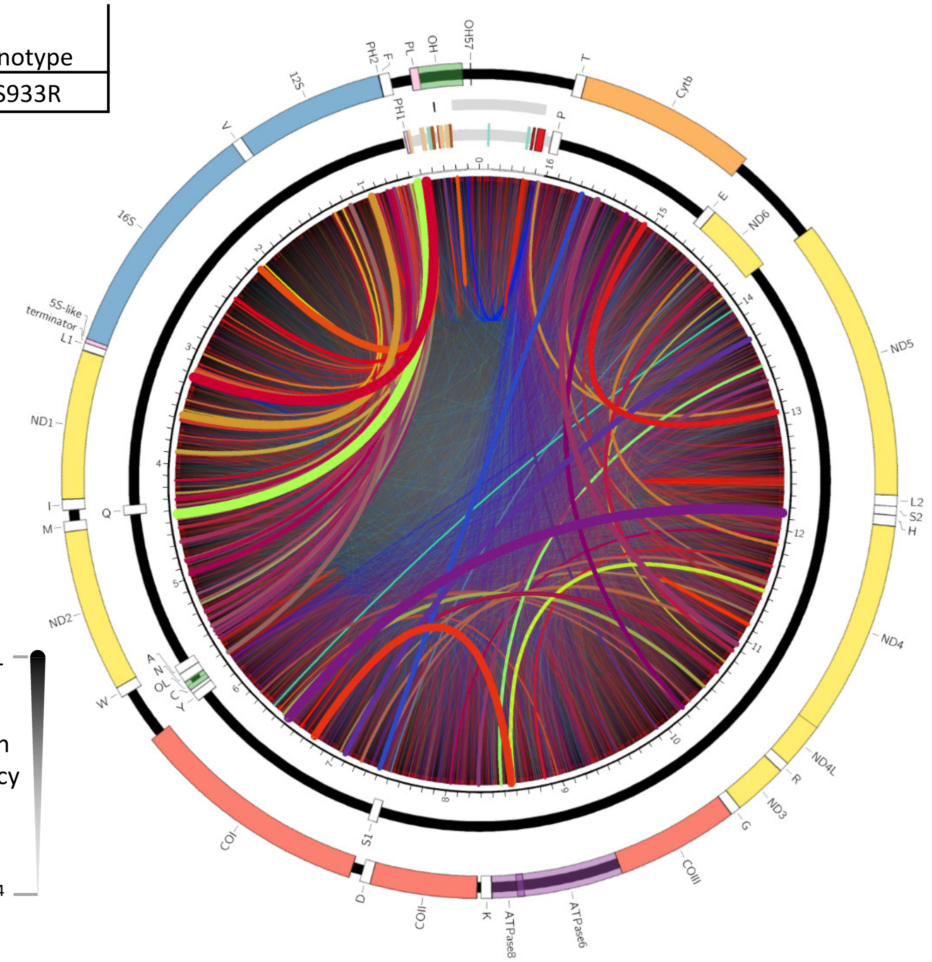

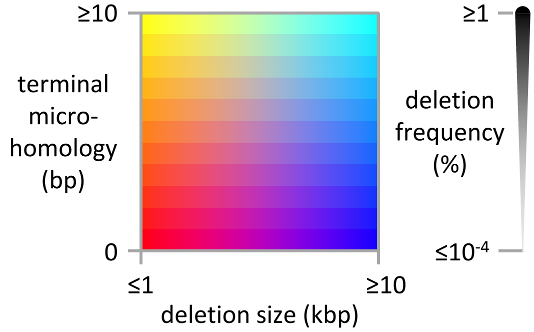

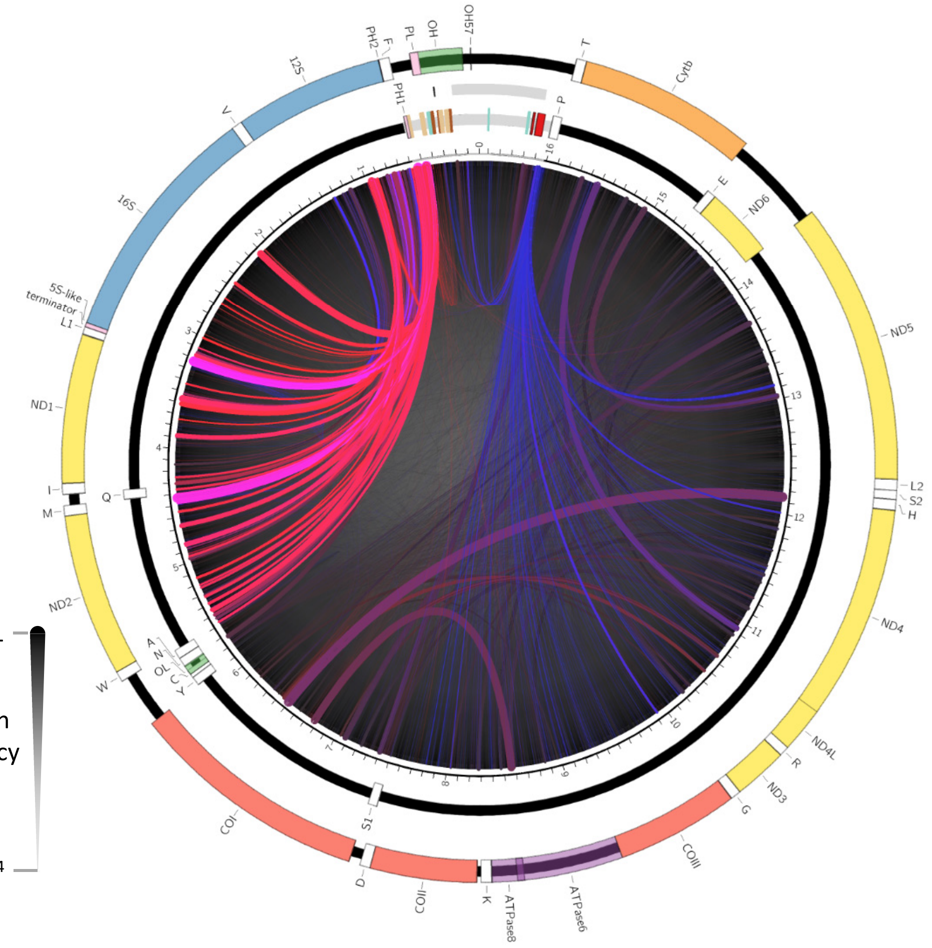

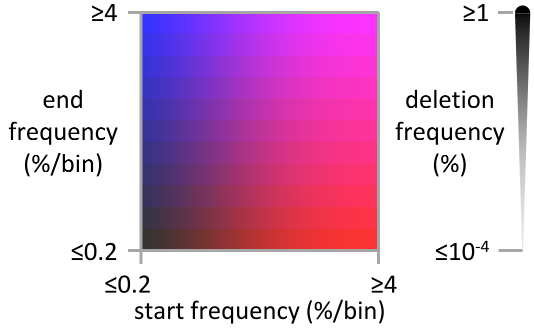

Sample	Gender	Age biopsy	Age onset	POLG genotype
M25	M	80	56	A467T;T251I/P587L

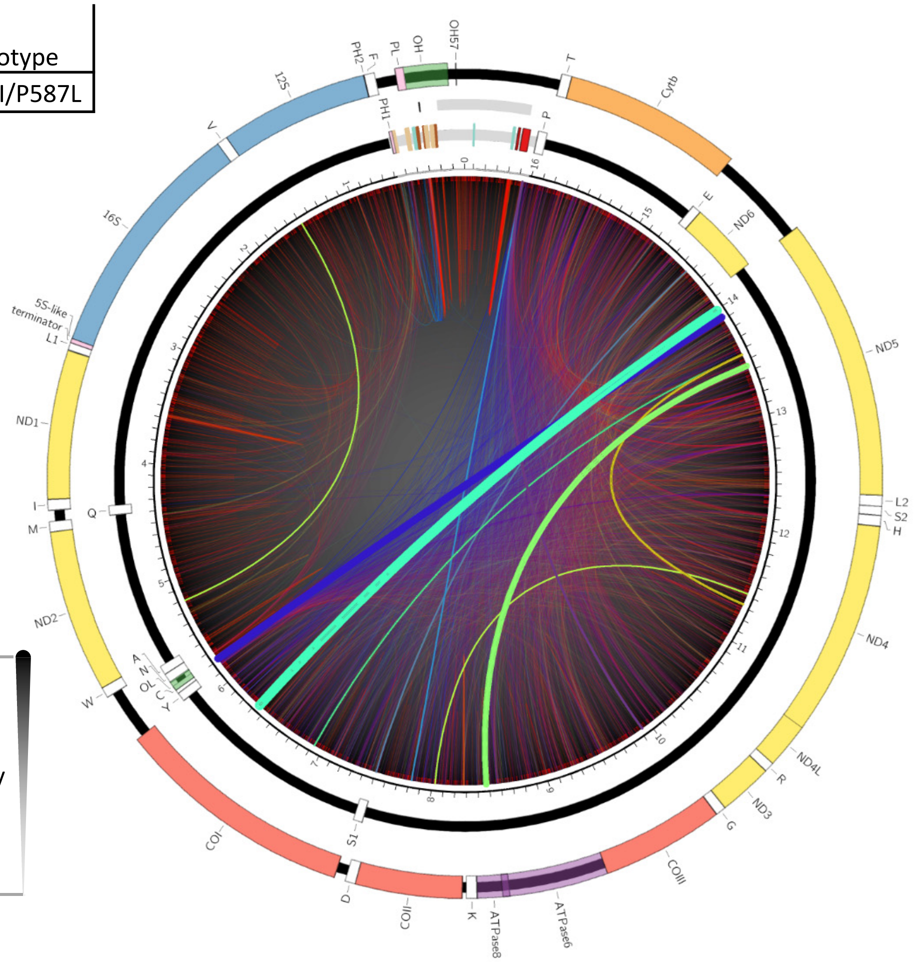

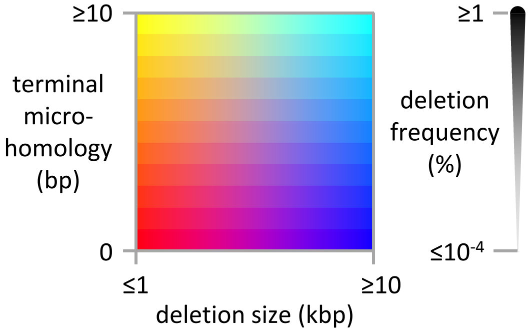

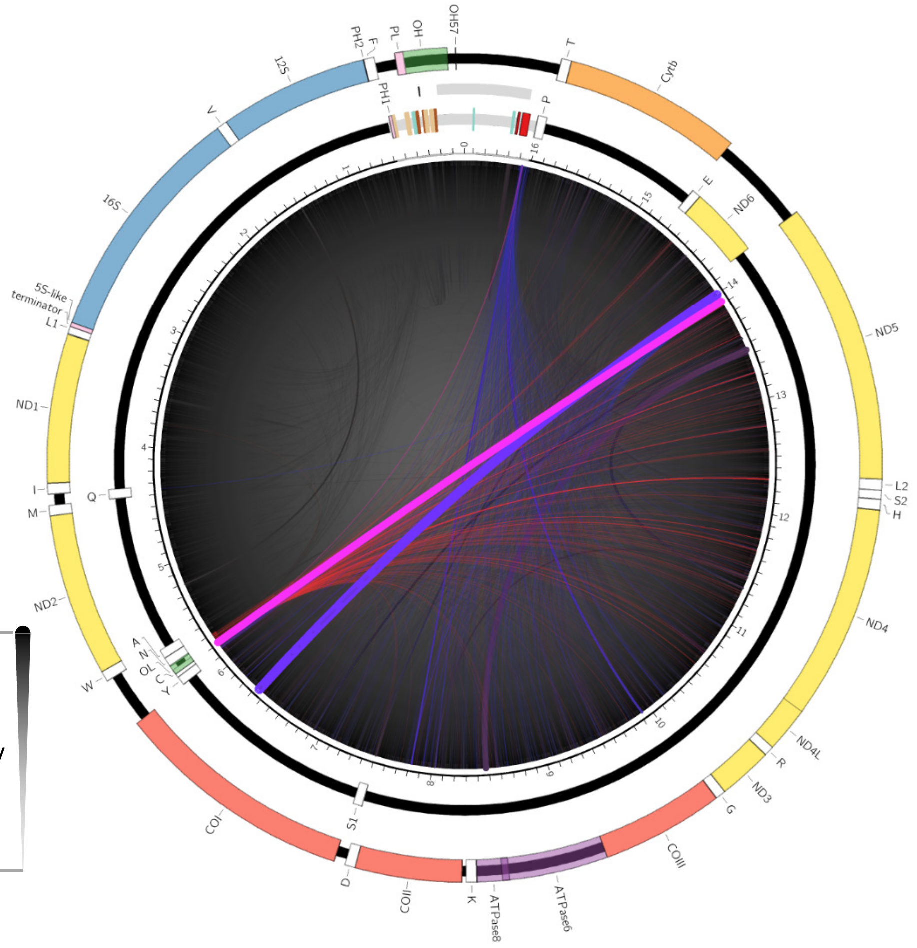

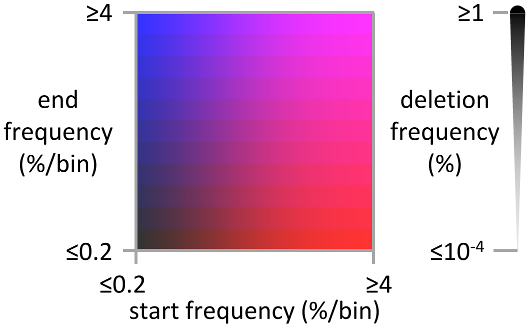

Sample	Gender	Age biopsy	Age onset	POLG genotype
M26	F	49	unk.	A467T;G737R

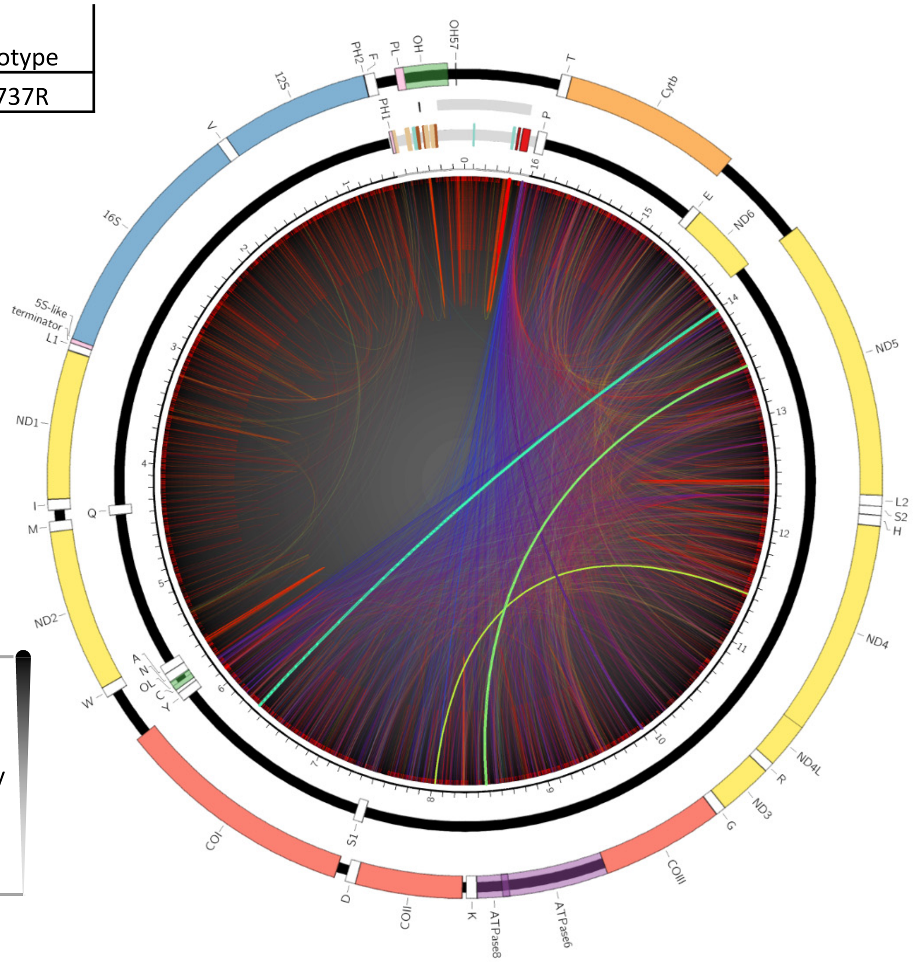

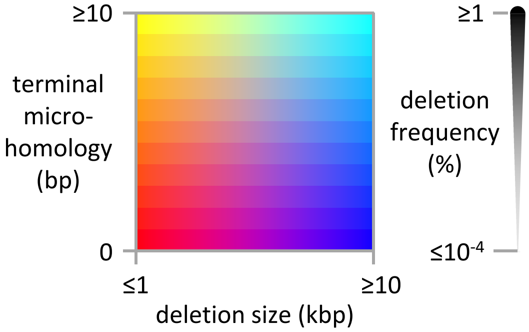

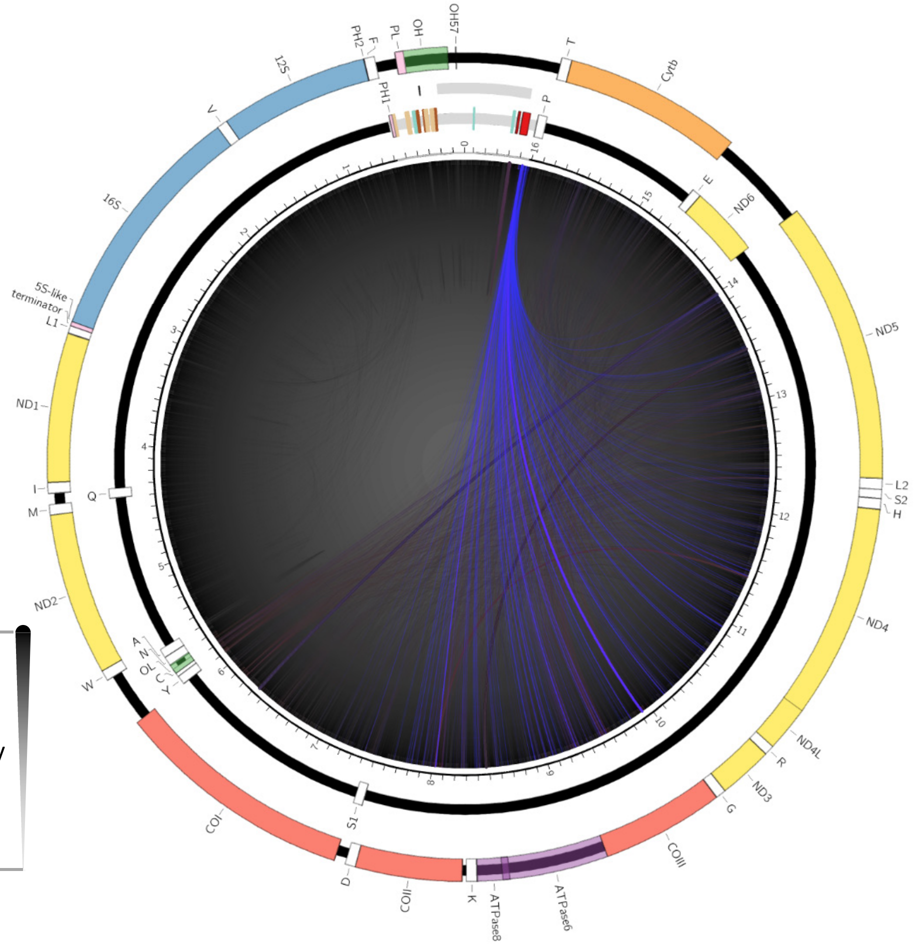

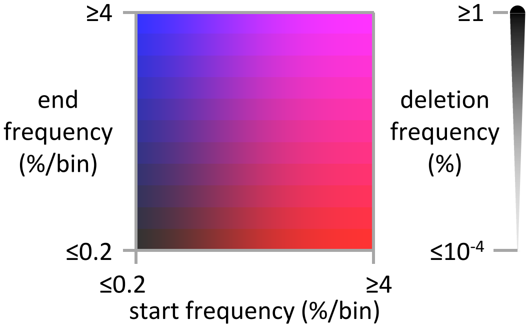

Sample	Gender	Age biopsy	Age onset	POLG genotype
M27	M	60	18	A467T; p.X1240Gln+35aa

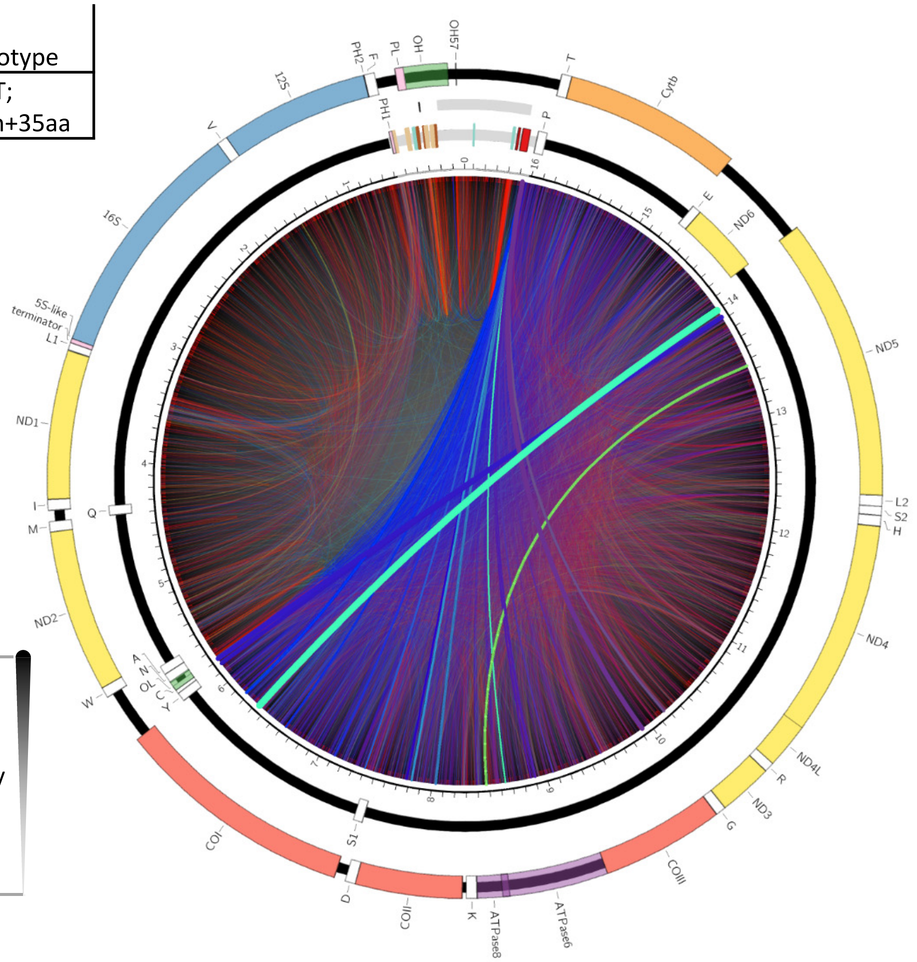

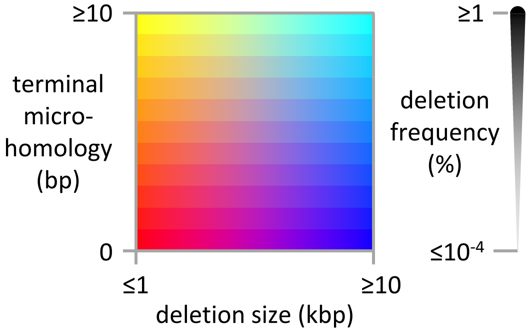

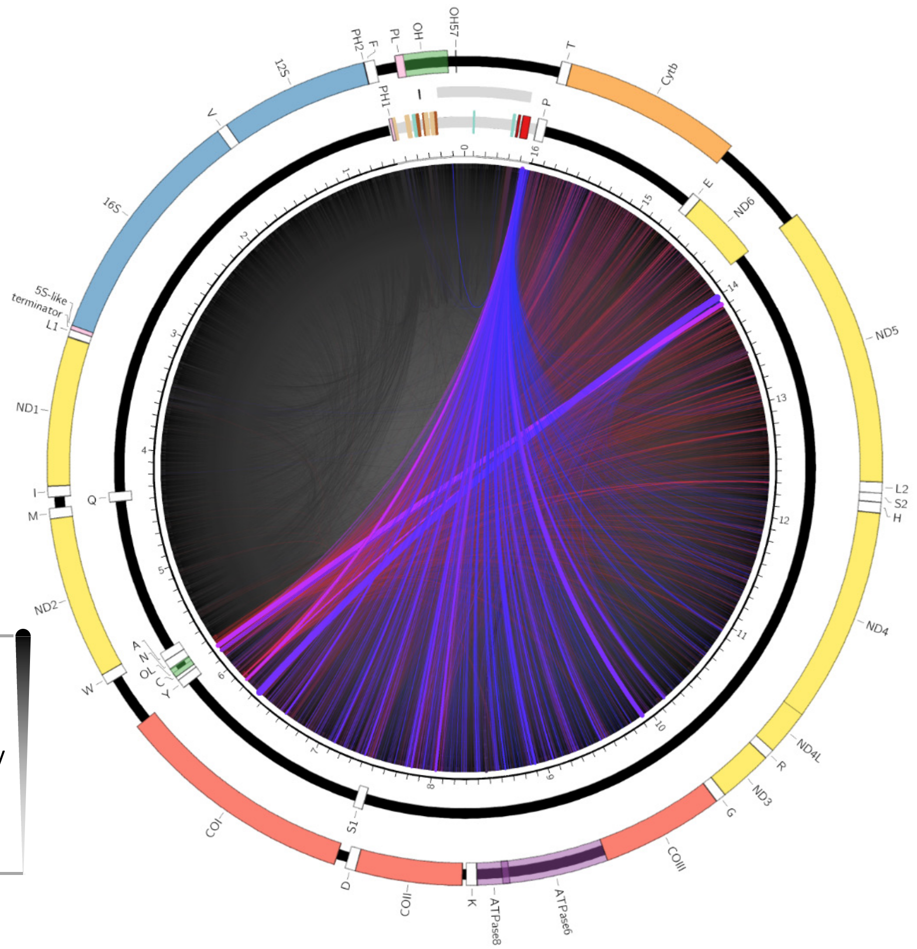

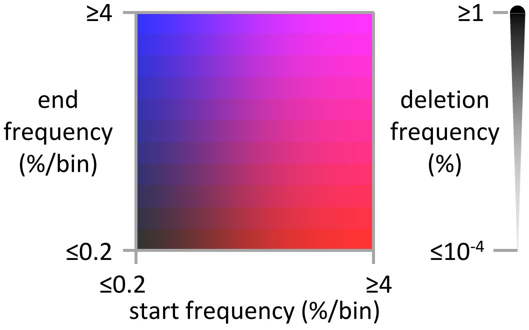

Sample	Gender	Age biopsy	Age onset	POLG genotype
M28	M	42	17	A467T;R1096C

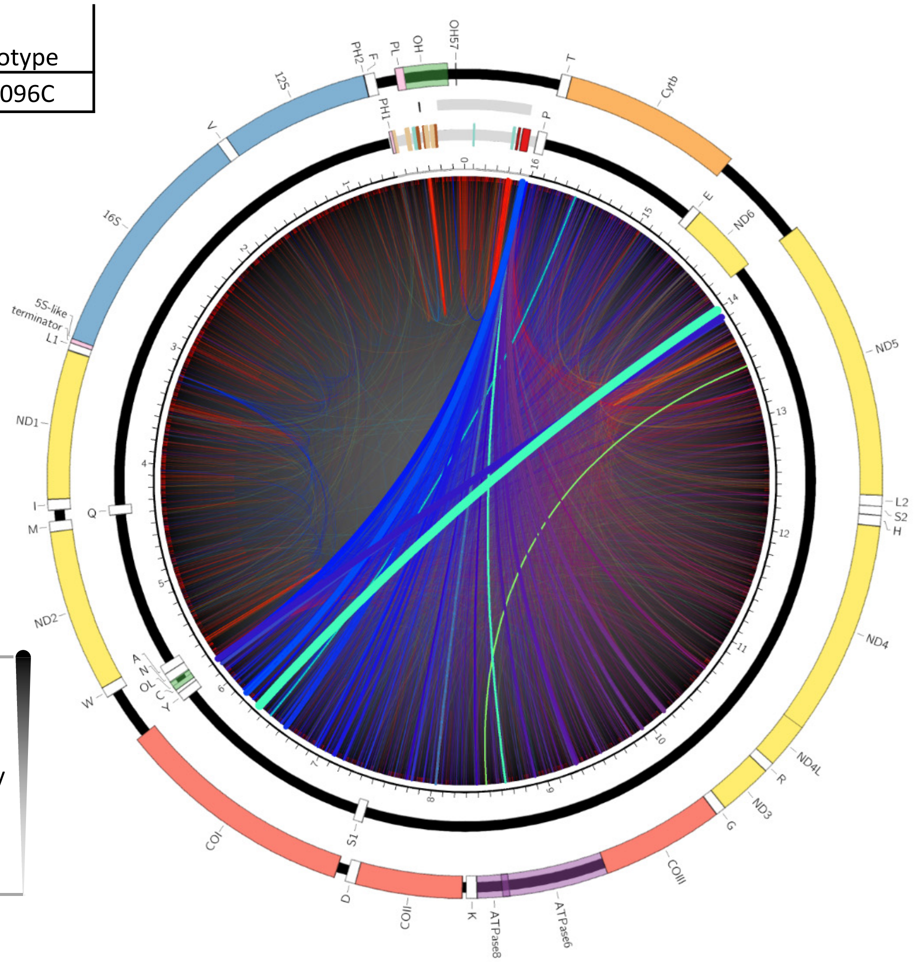

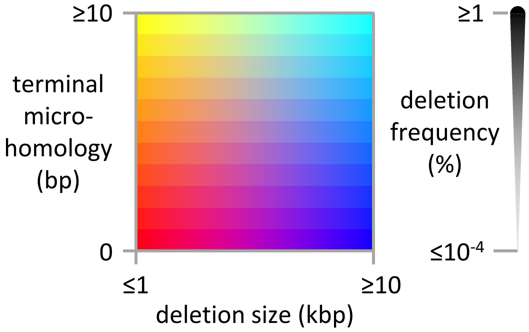

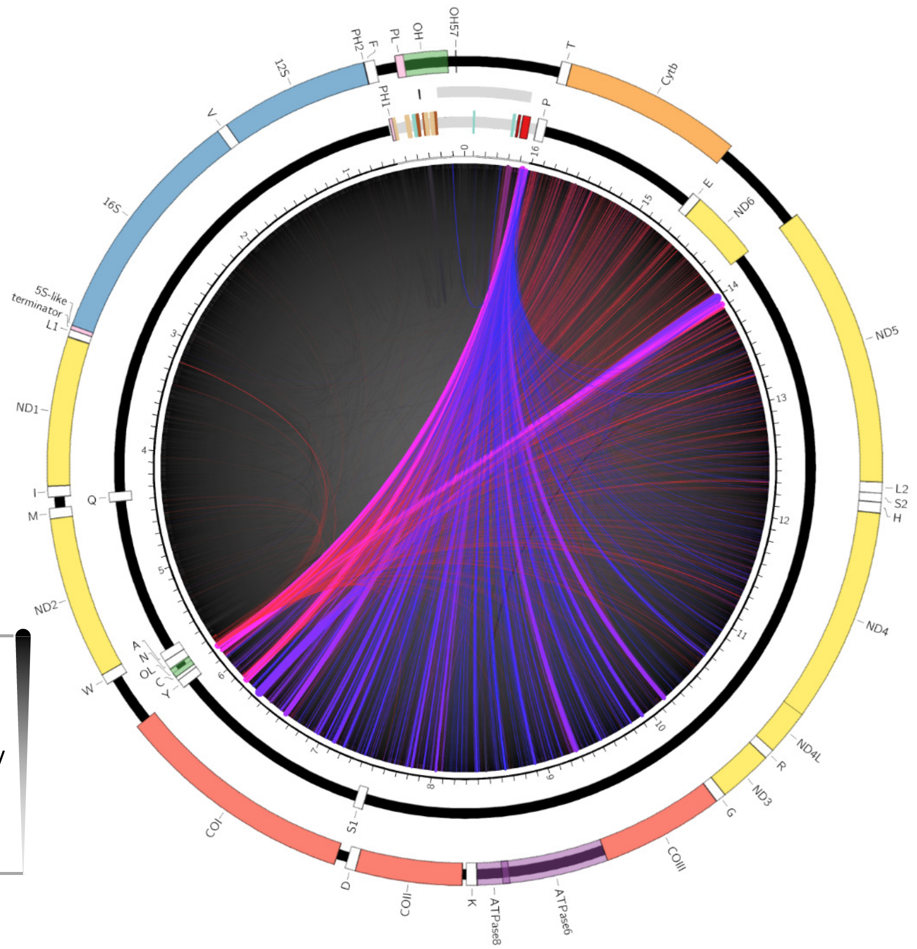

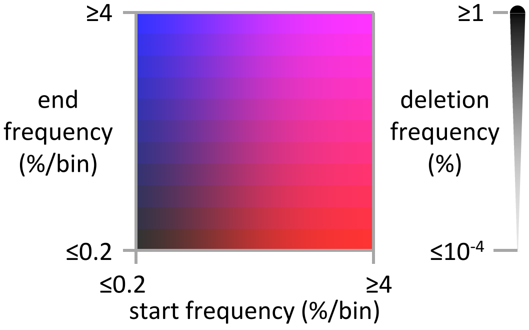

Sample	Gender	Age biopsy	Age onset	POLG genotype
M29	F	69	60	A467T;T251I/P587L

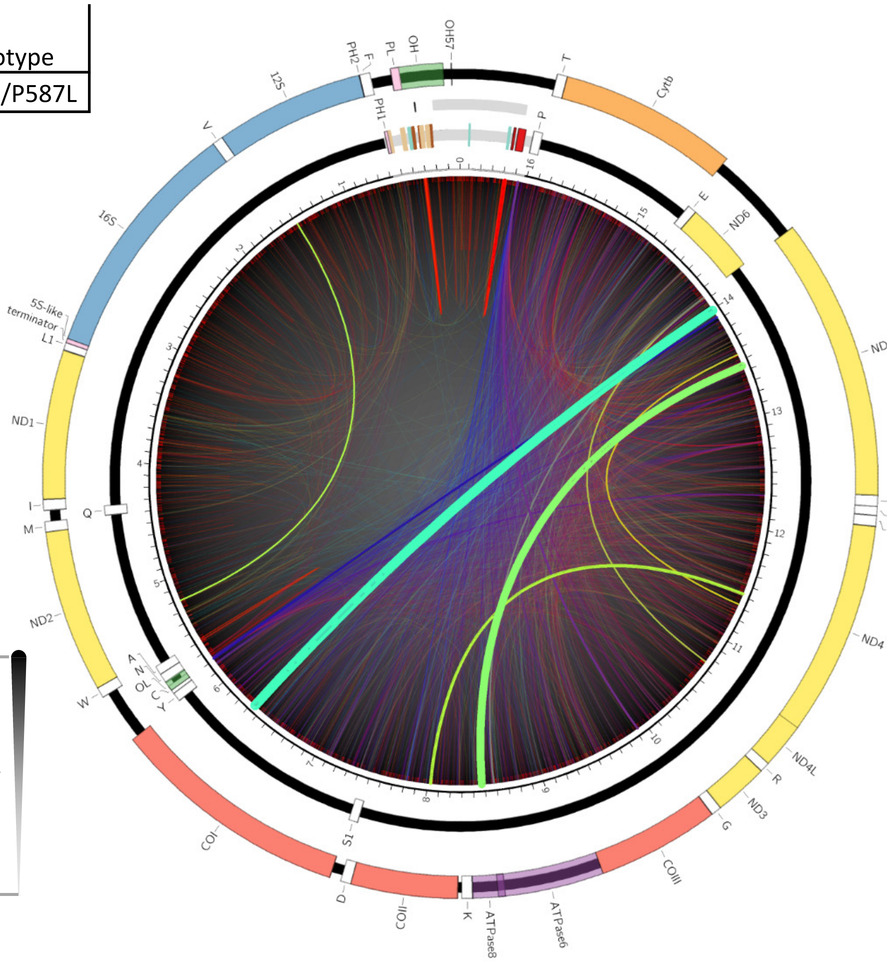

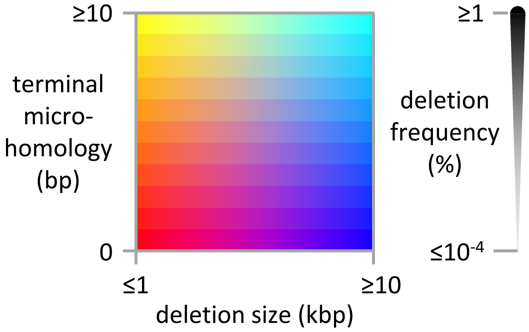

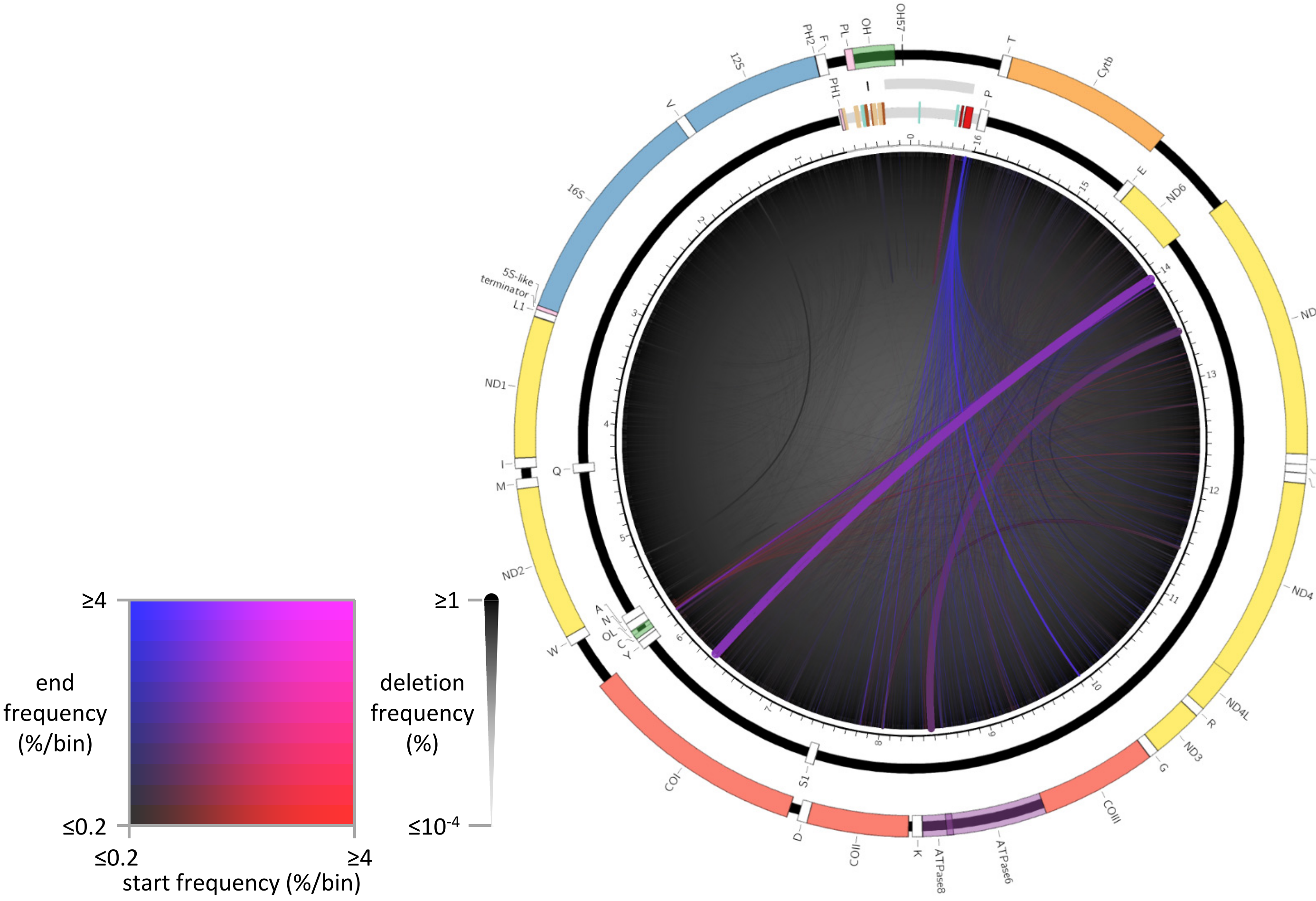

Sample	Gender	Age biopsy	Age onset	POLG genotype
M30	F	59	39	A467T;W748S

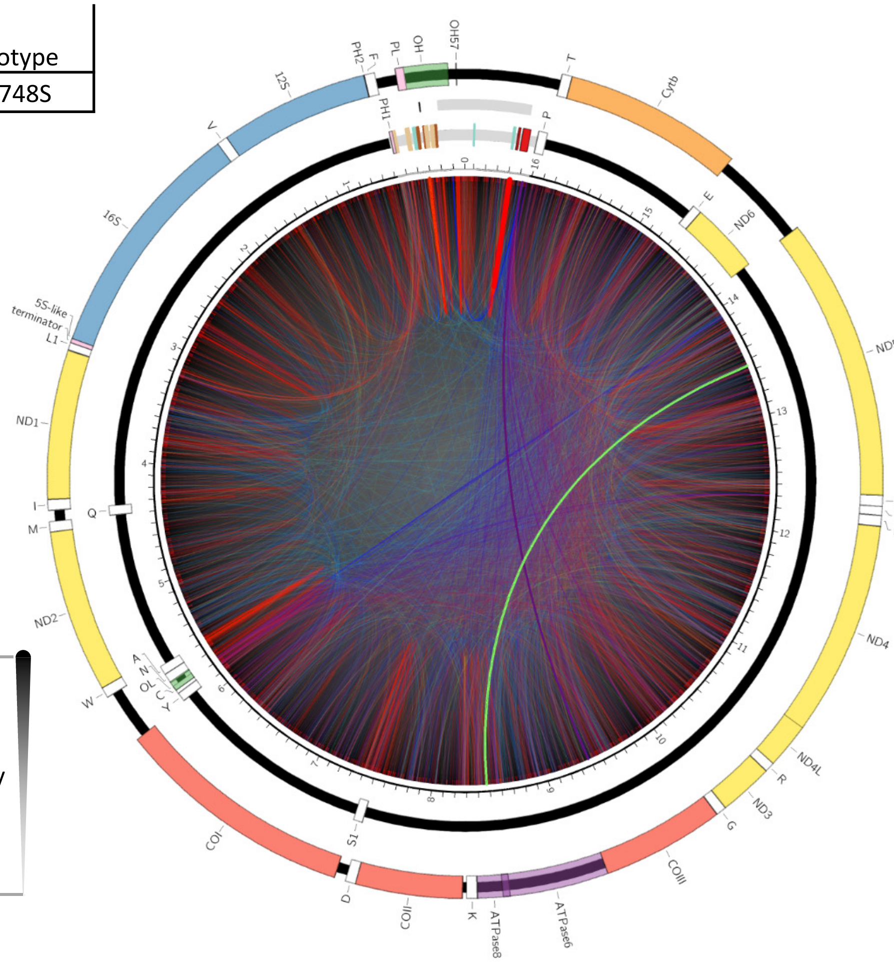

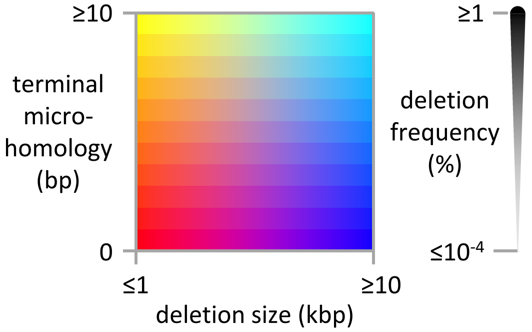

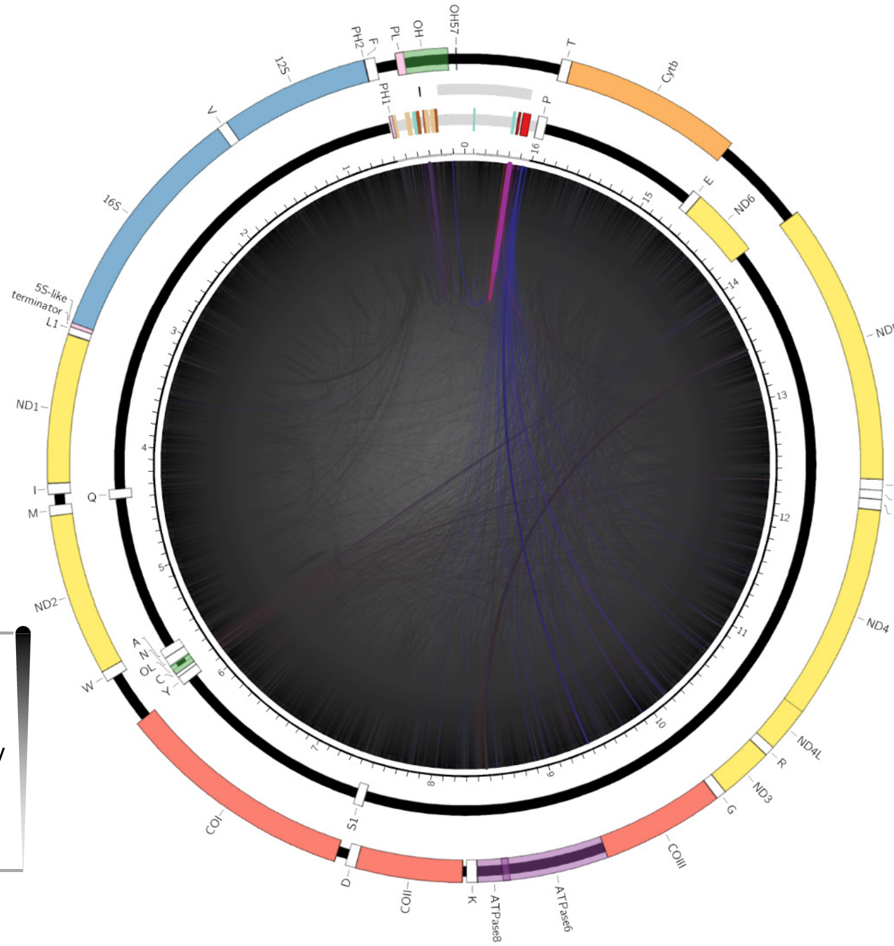

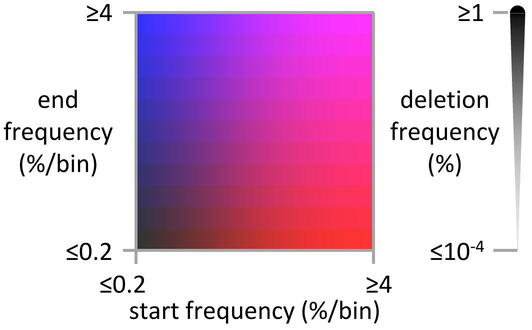

Sample	Gender	Age biopsy	Age onset	POLG genotype
M31	M	62	34	A467T;W748S

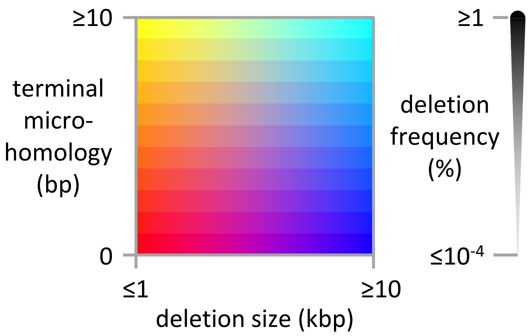

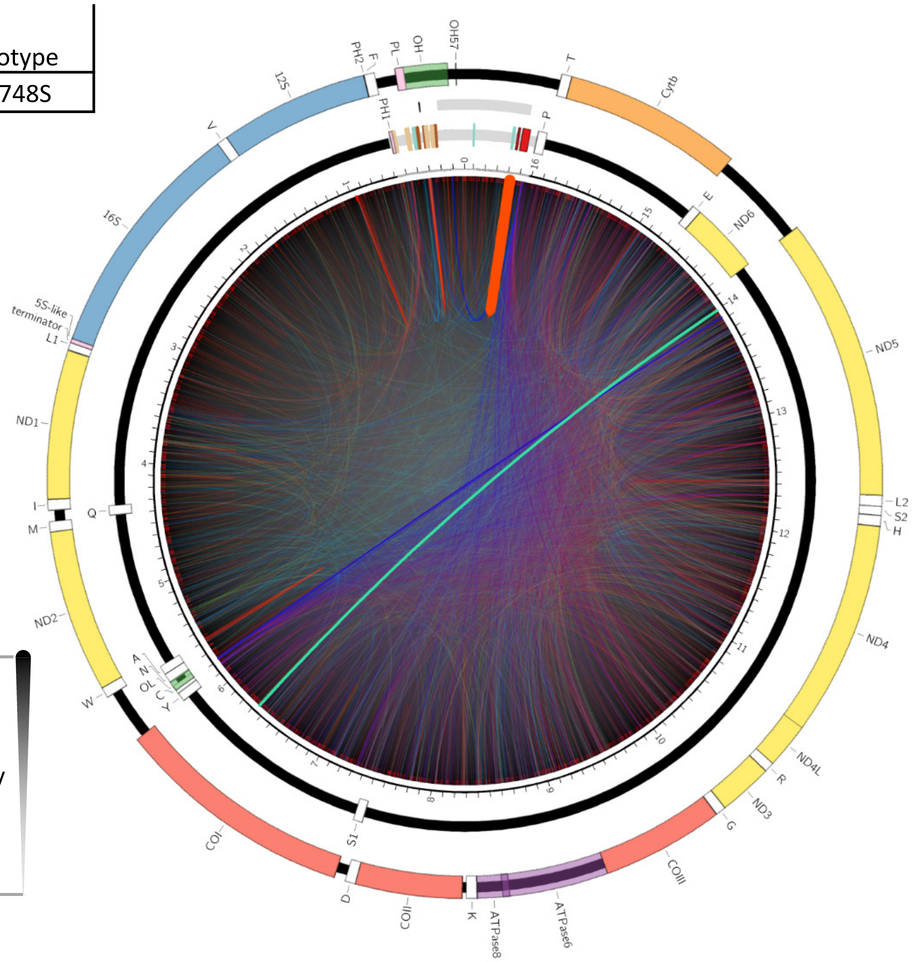

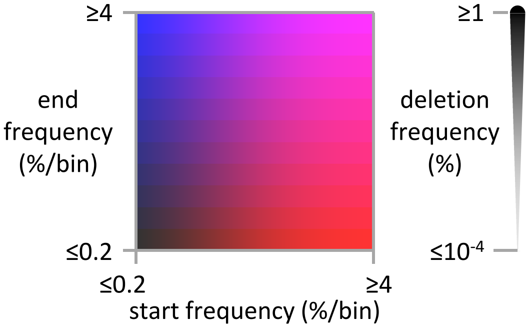

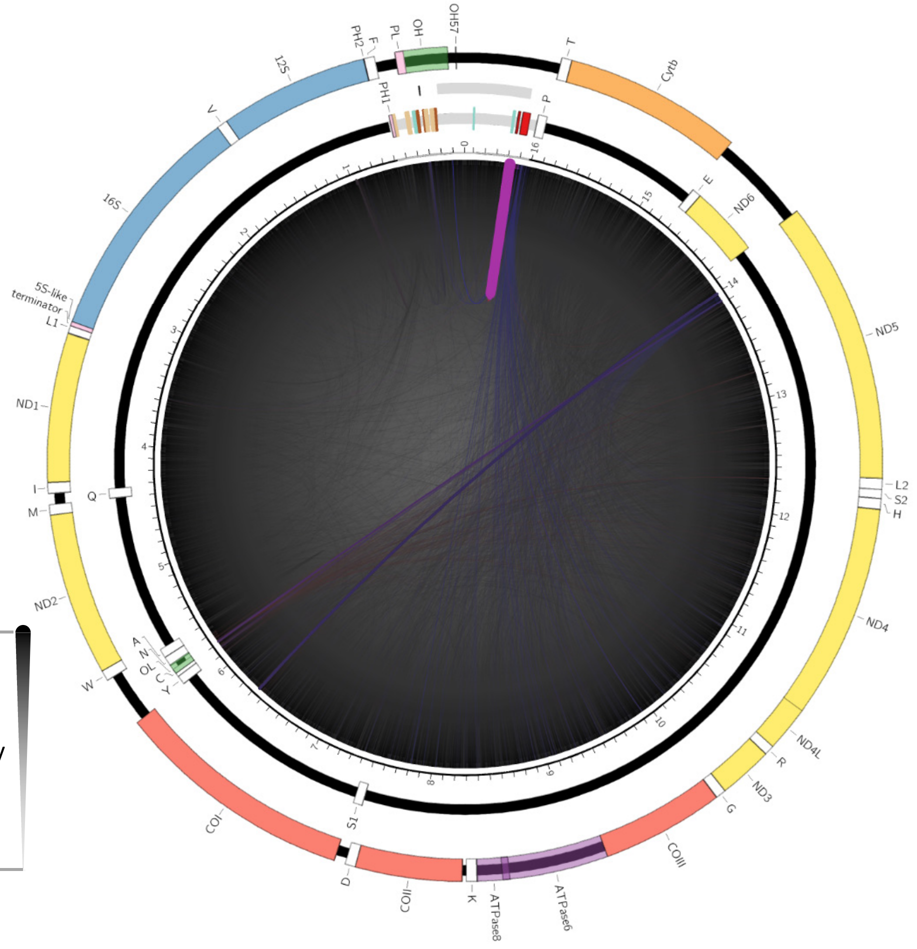

Sample	Gender	Age biopsy	Age onset	POLG genotype
M32	M	58	22	G848S;S1104C

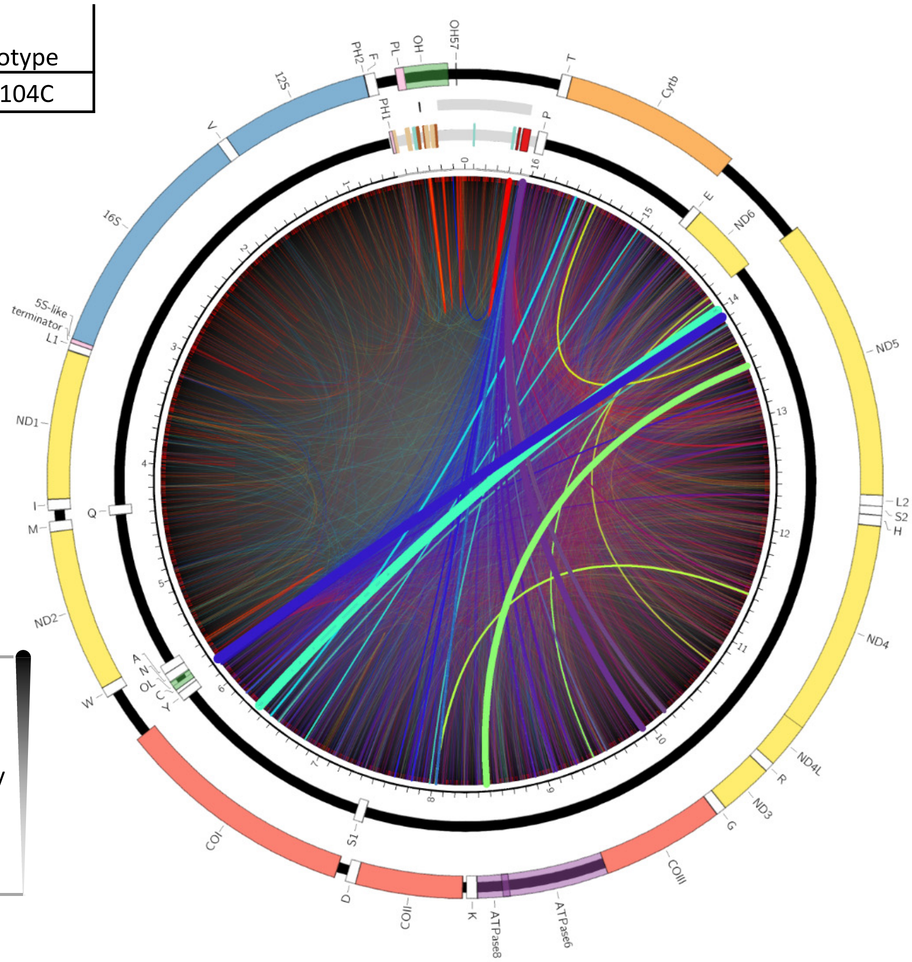

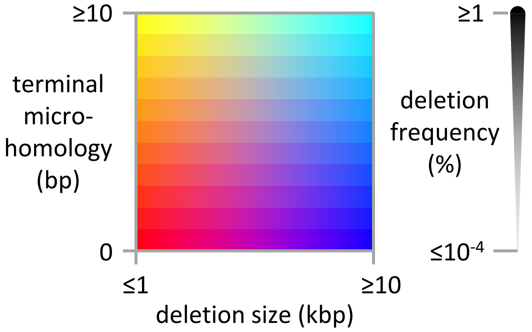

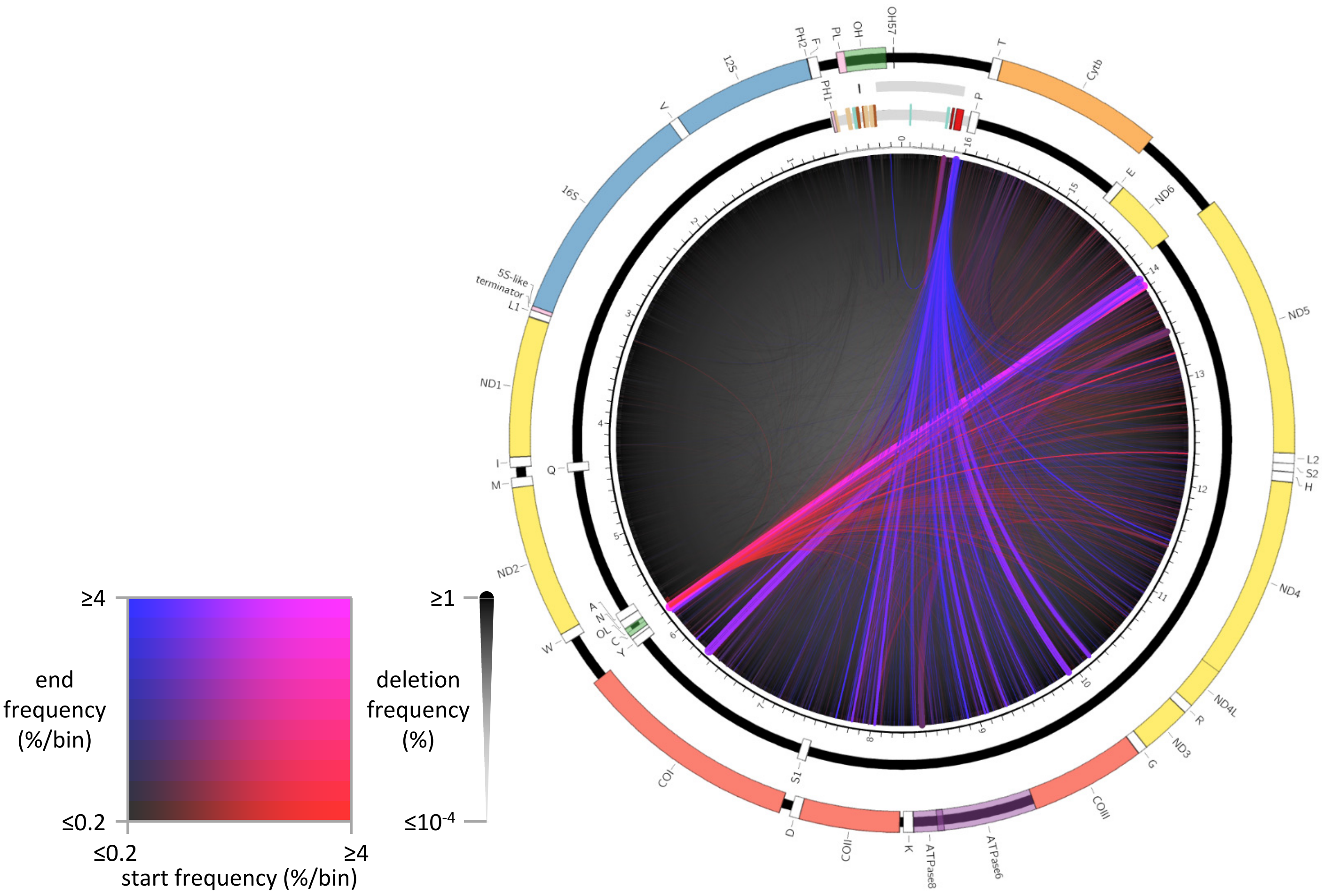

Sample	Gender	Age biopsy	Age onset	POLG genotype
M33	M	71	unk.	G952R

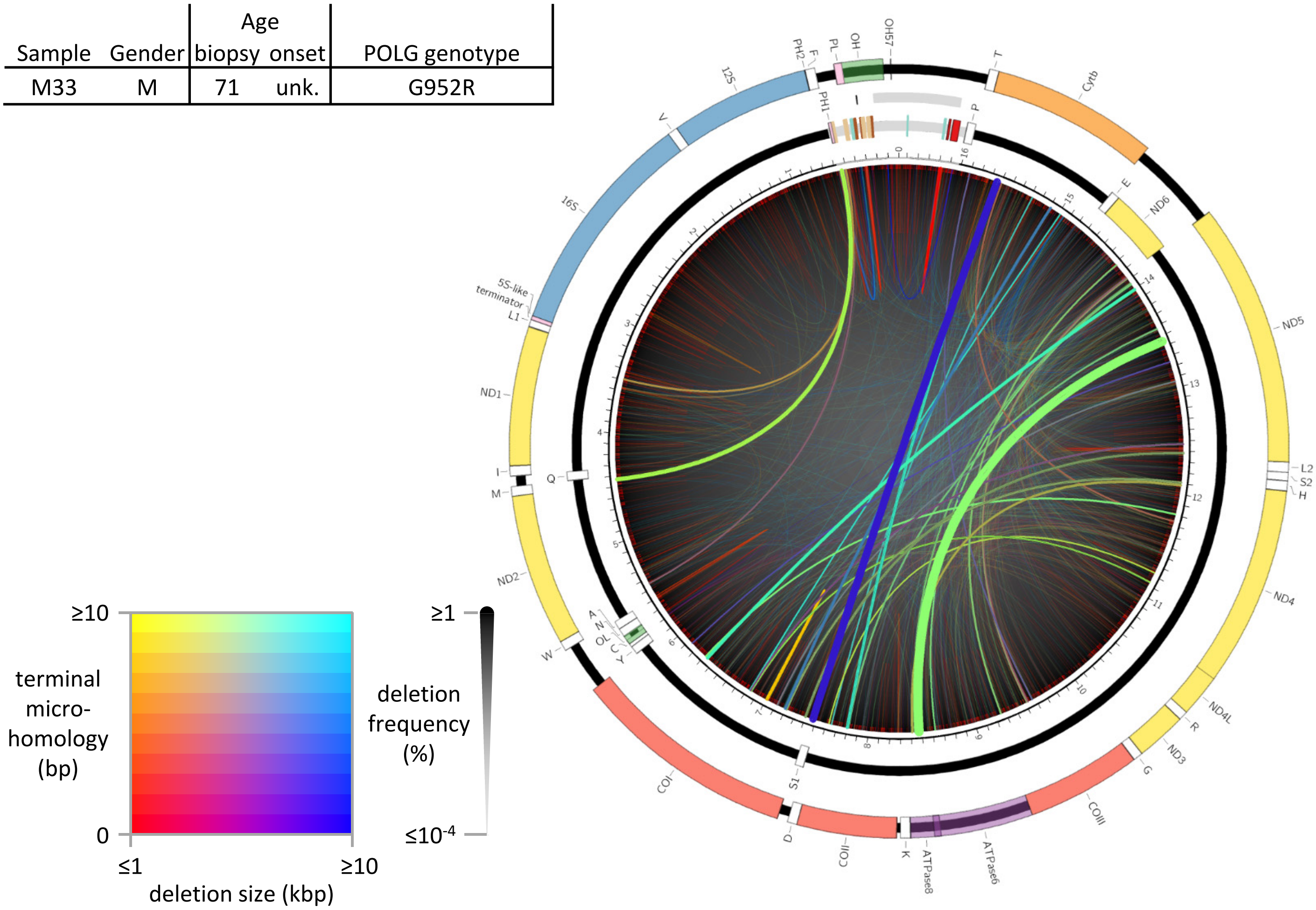

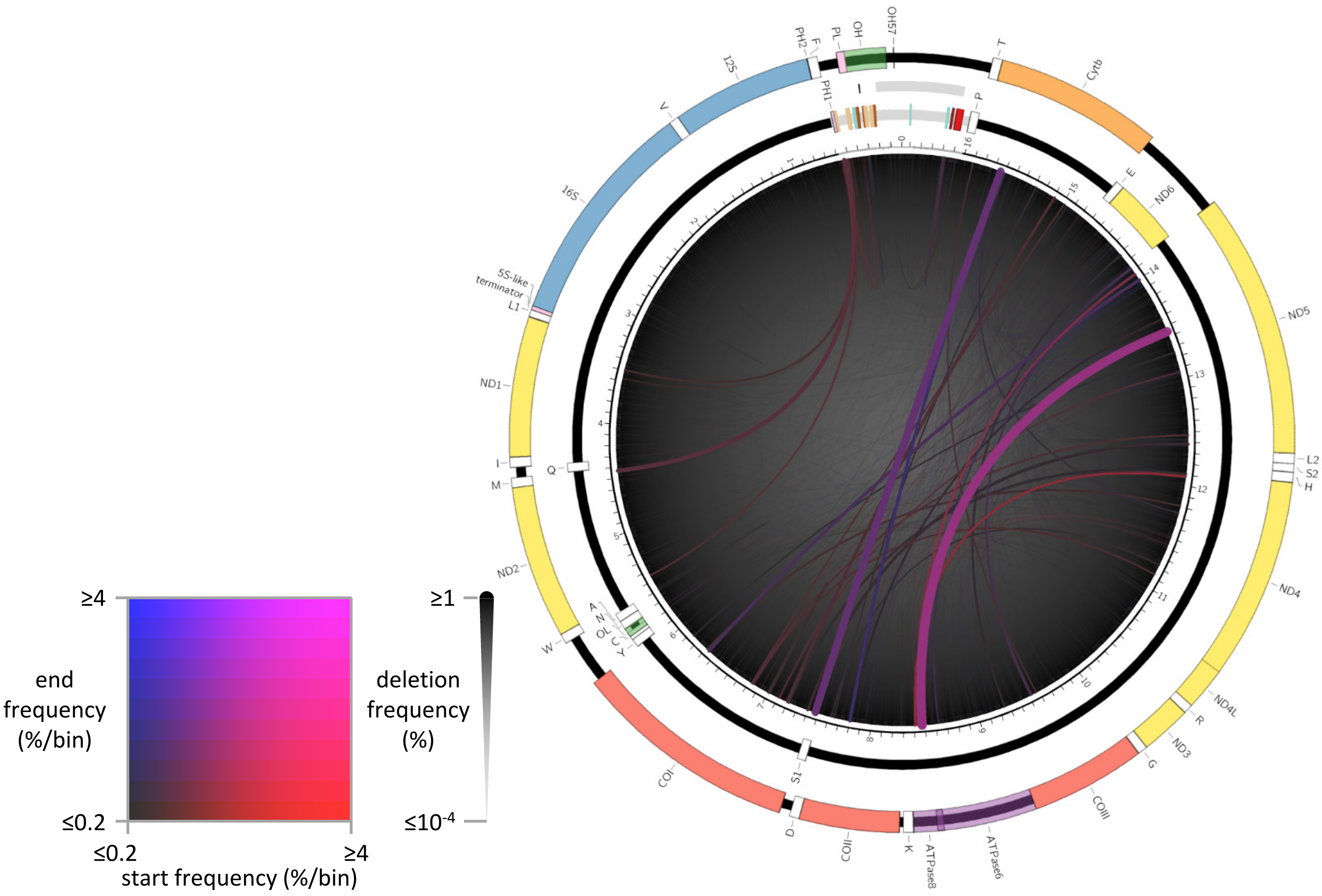

Sample	Gender	Age biopsy	Age onset	POLG genotype
M34	F	70	61	L411P;R574Q

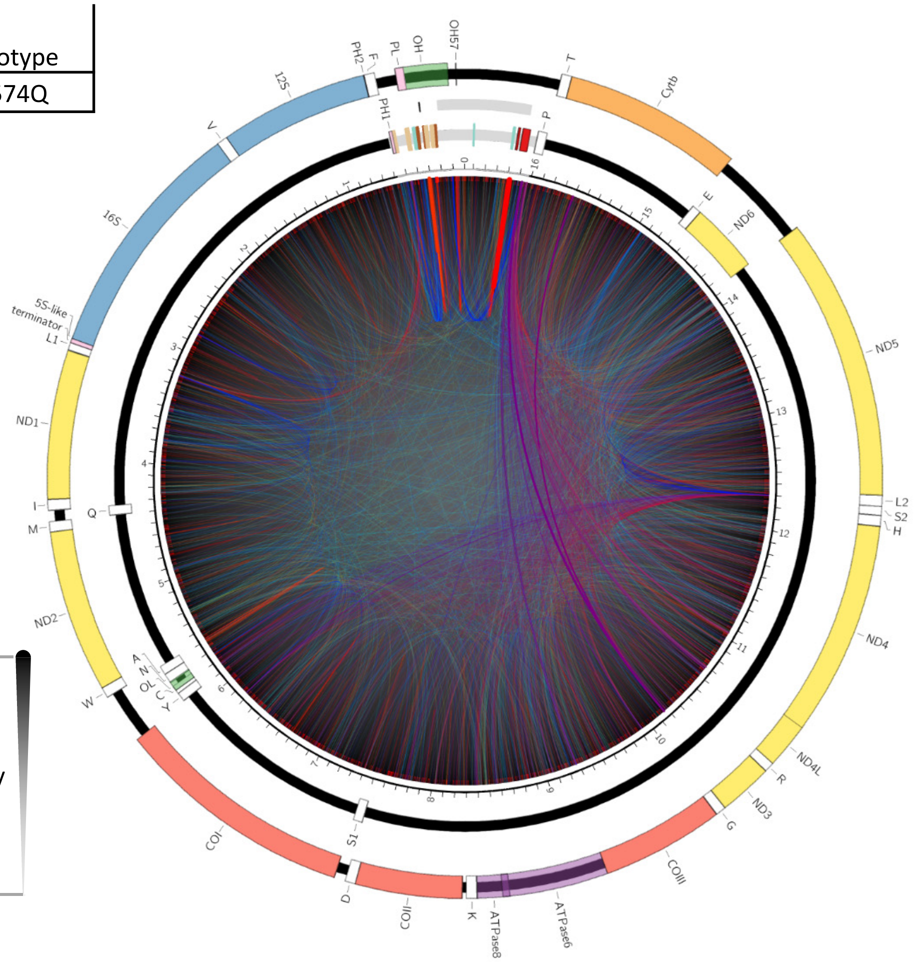

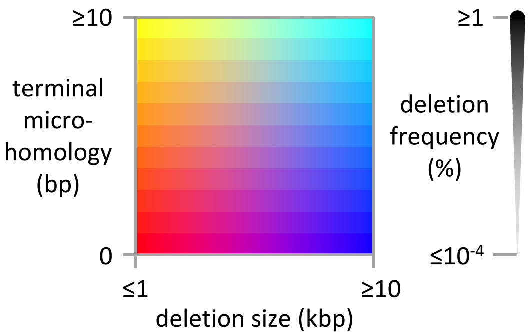

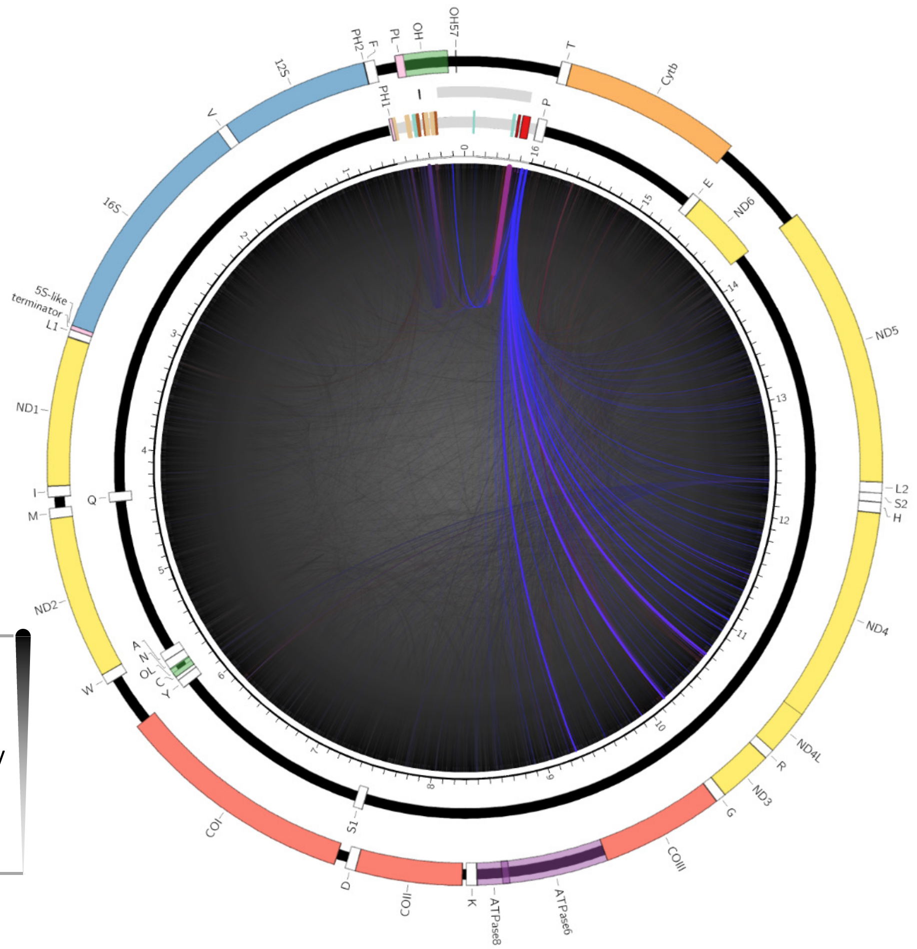

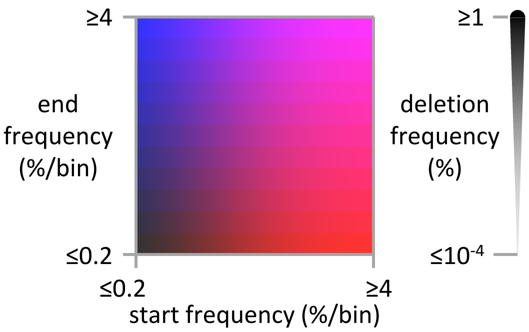

Sample	Gender	Age biopsy	Age onset	POLG genotype
M35	M	49	15	M797I;T251I/P587L

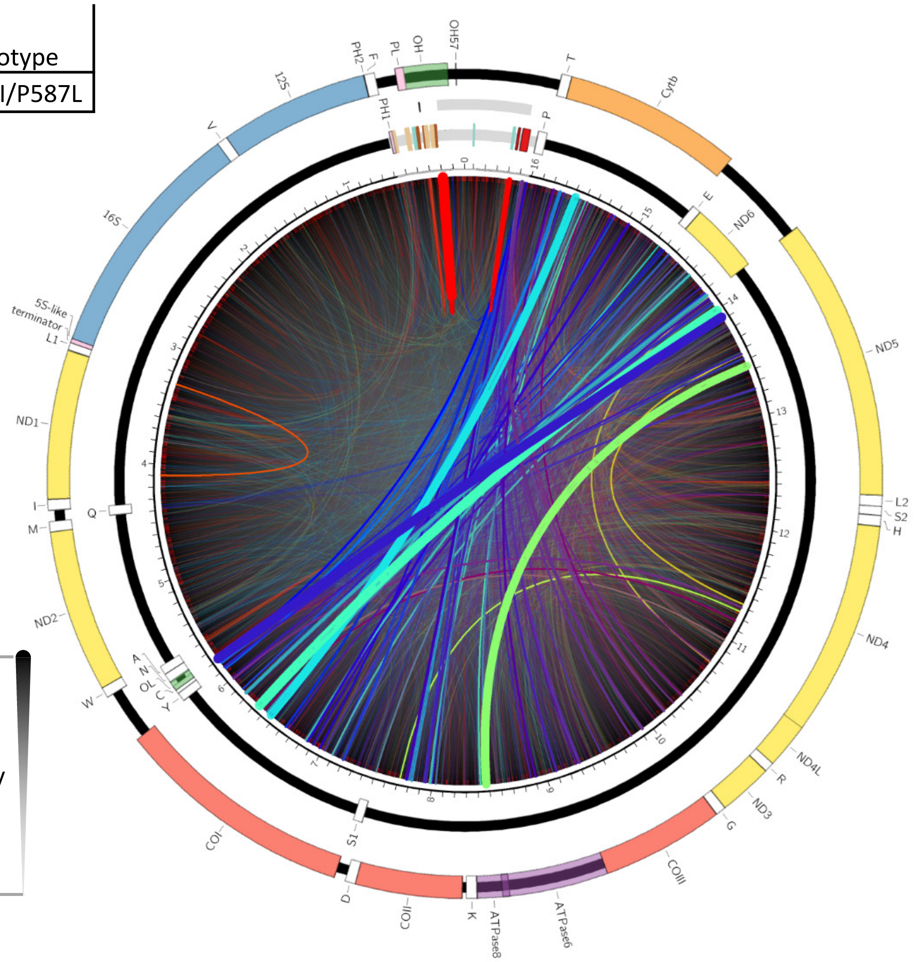

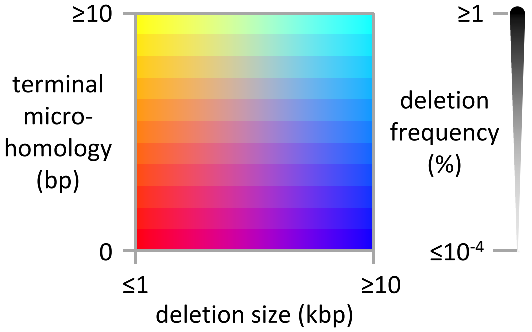

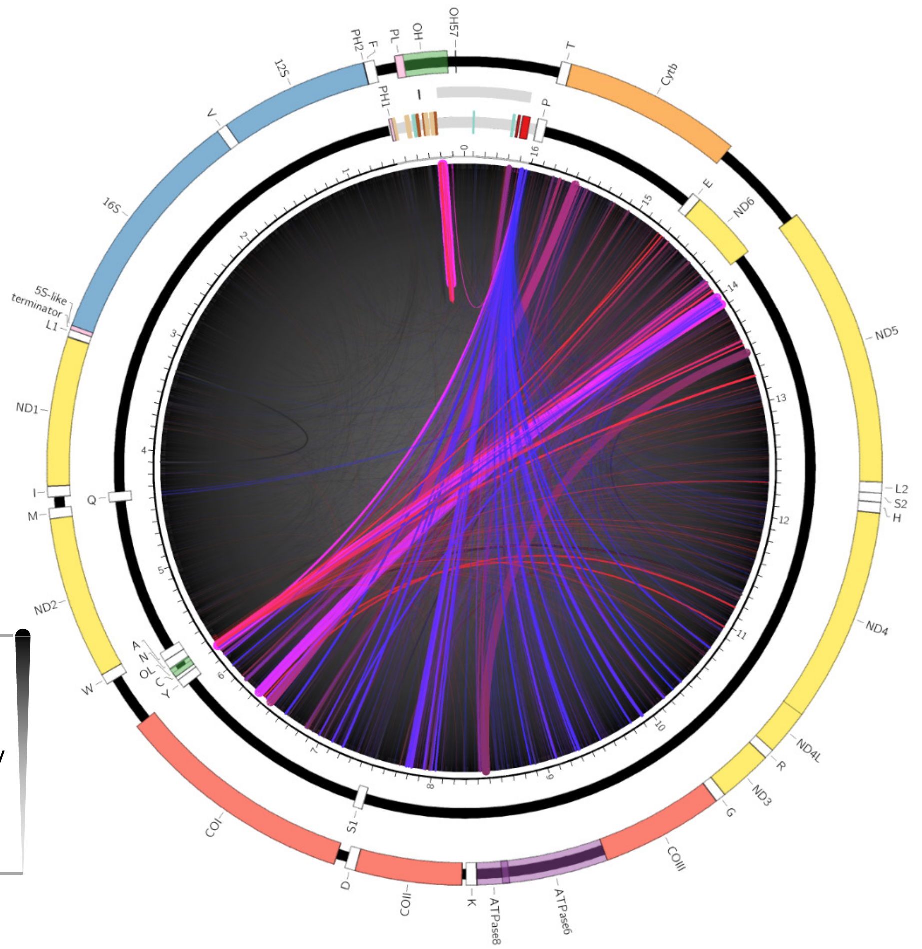

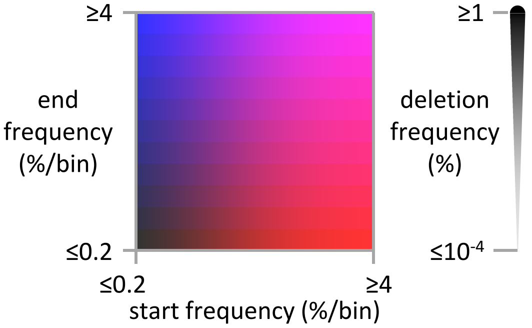

		Age		
Sample	Gender	biopsy	onset	POLG genotype
M36	M	17	16	R597W;R597W

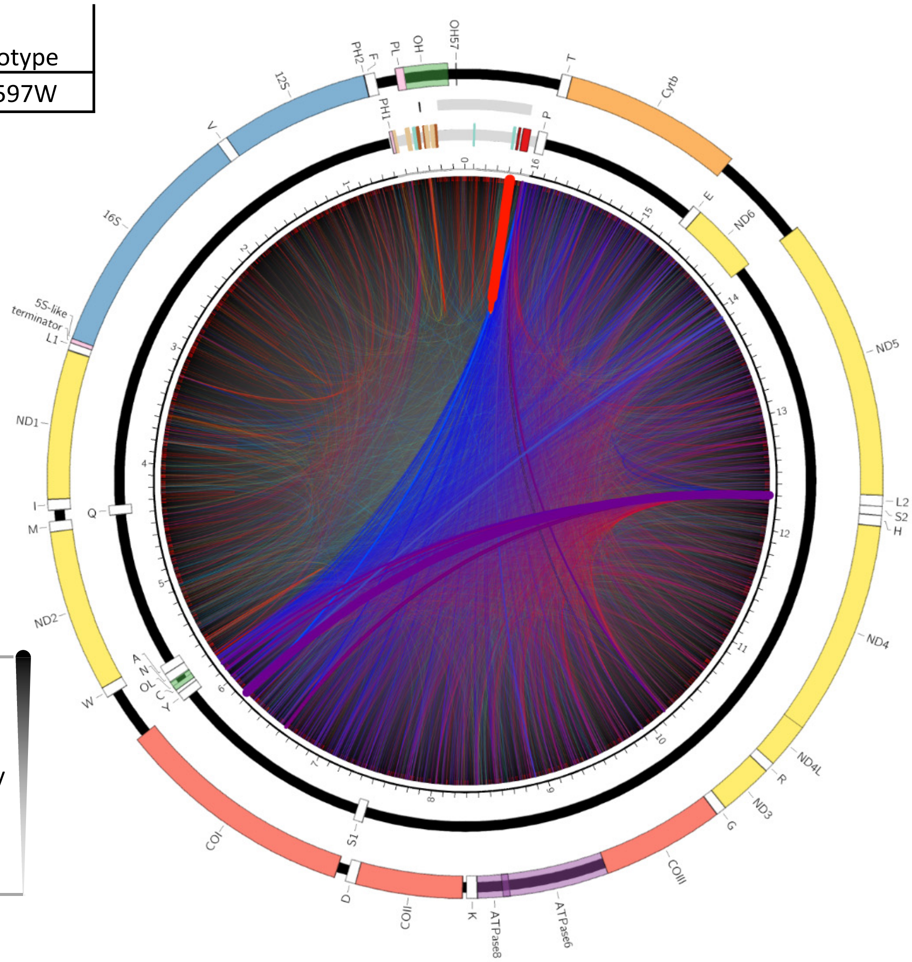

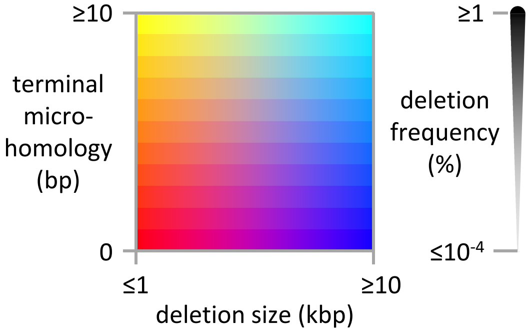

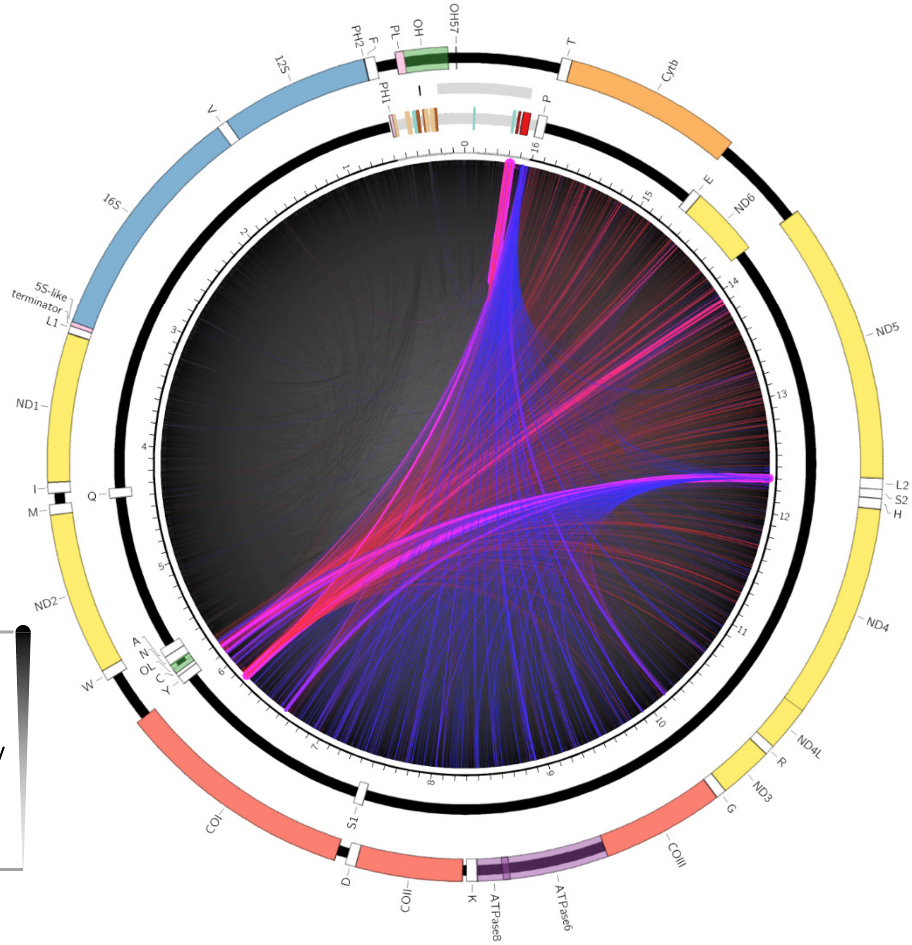

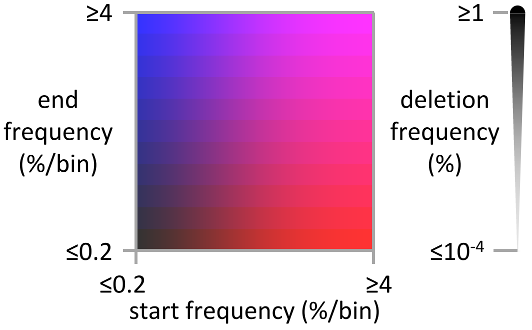

Sample	Gender	Age biopsy	Age onset	POLG genotype
M37	M	29	23	R627Q;G848S

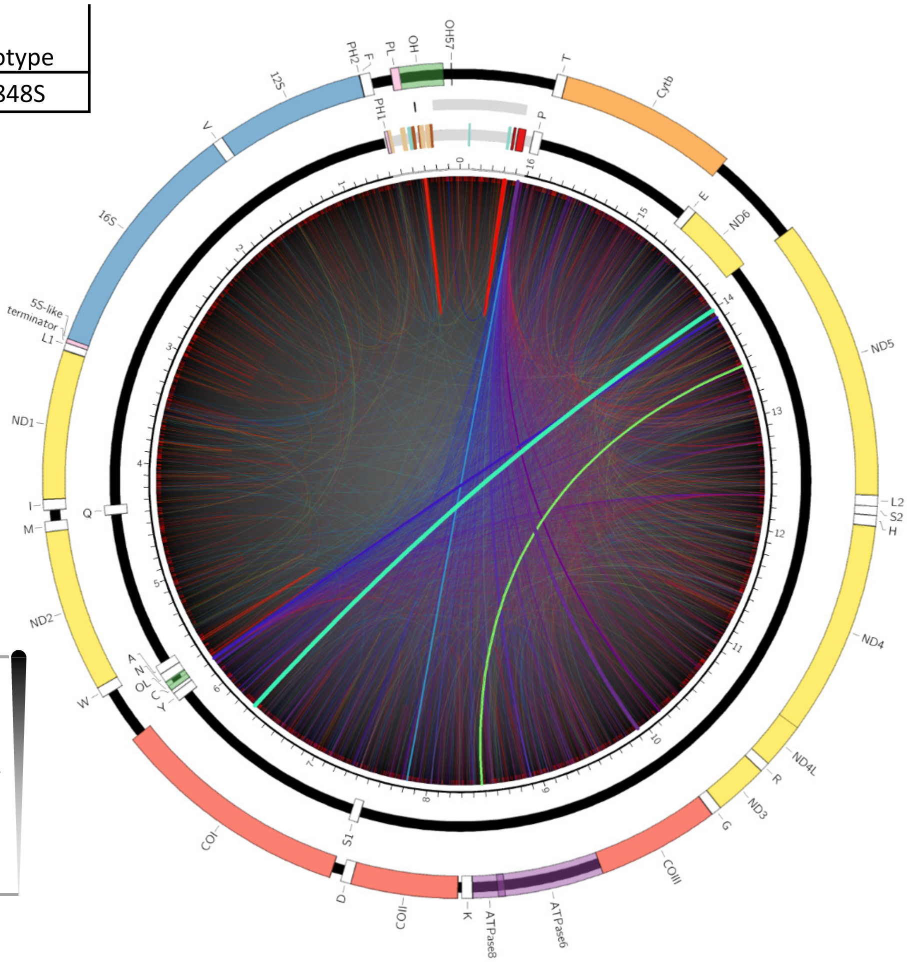

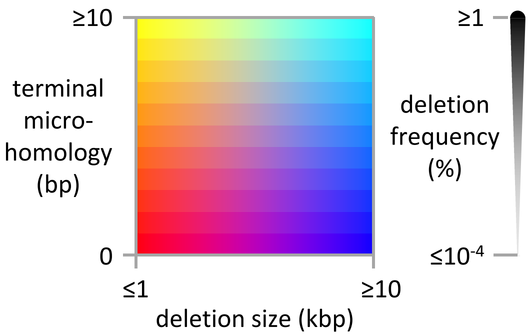

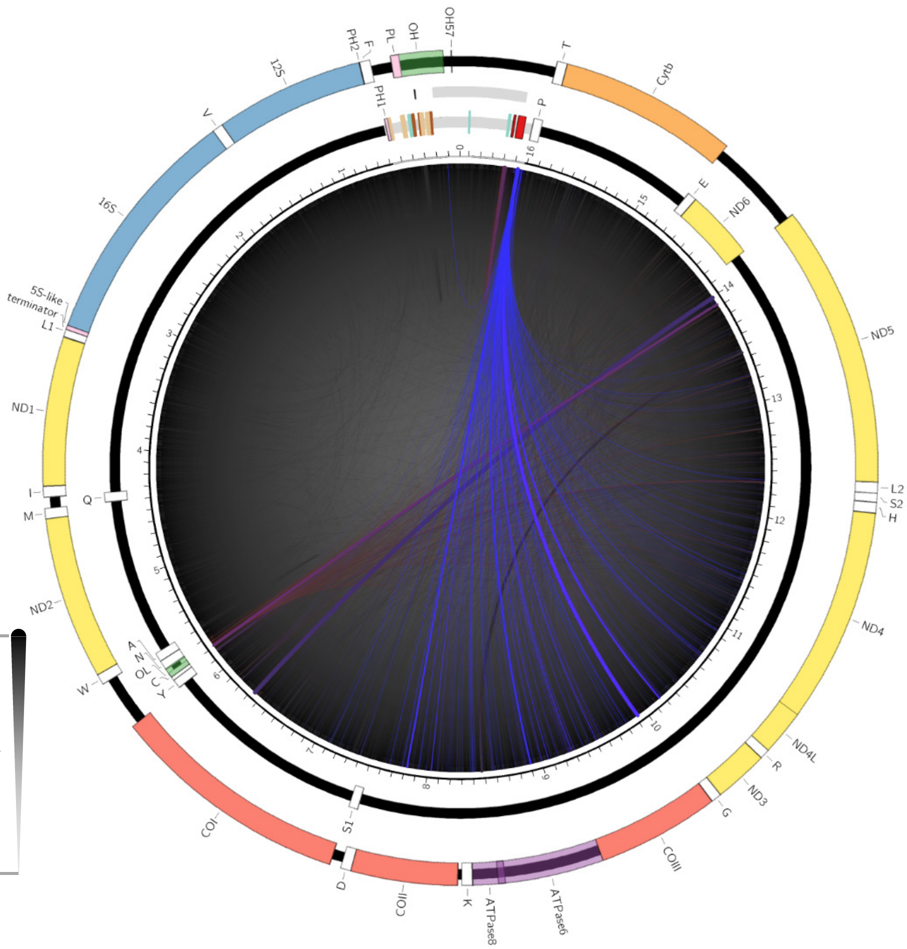

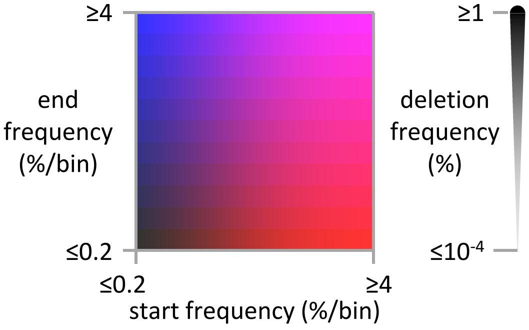

Sample	Gender	Age biopsy	Age onset	POLG genotype
M38	F	74	57	R852C;T251I/P587L

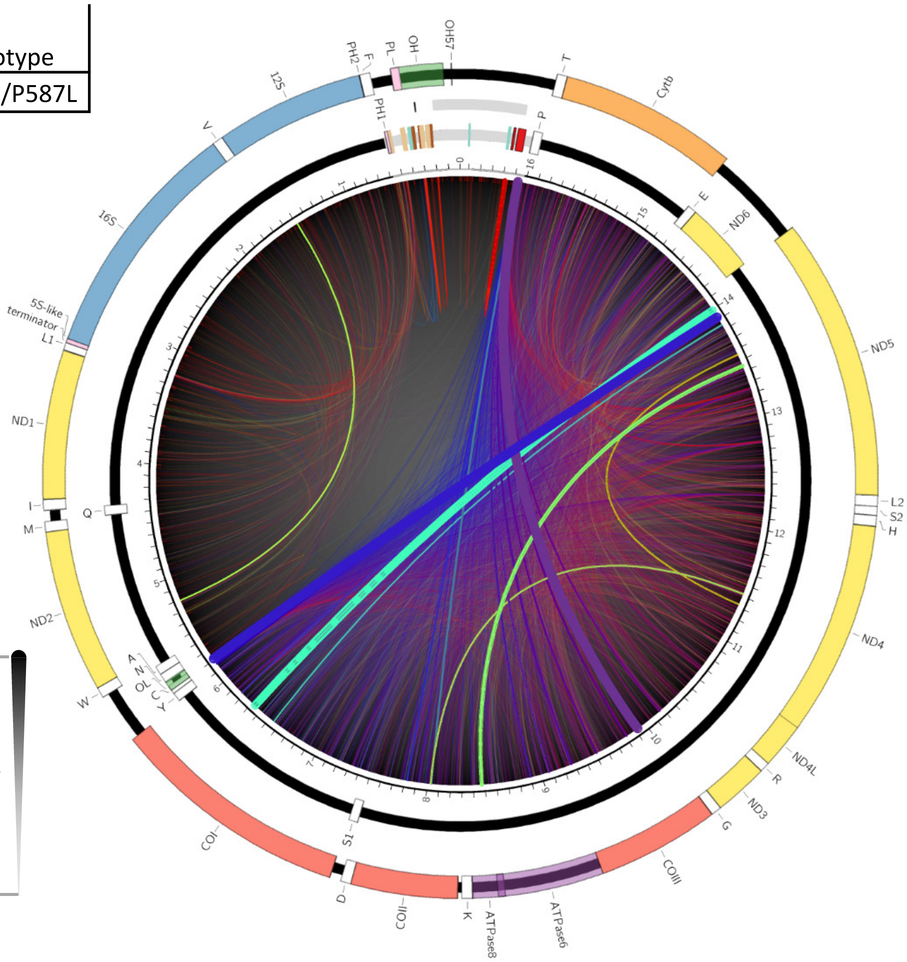

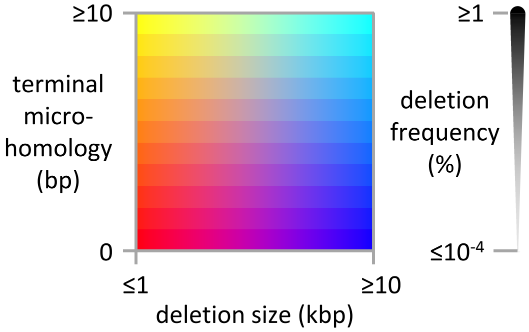

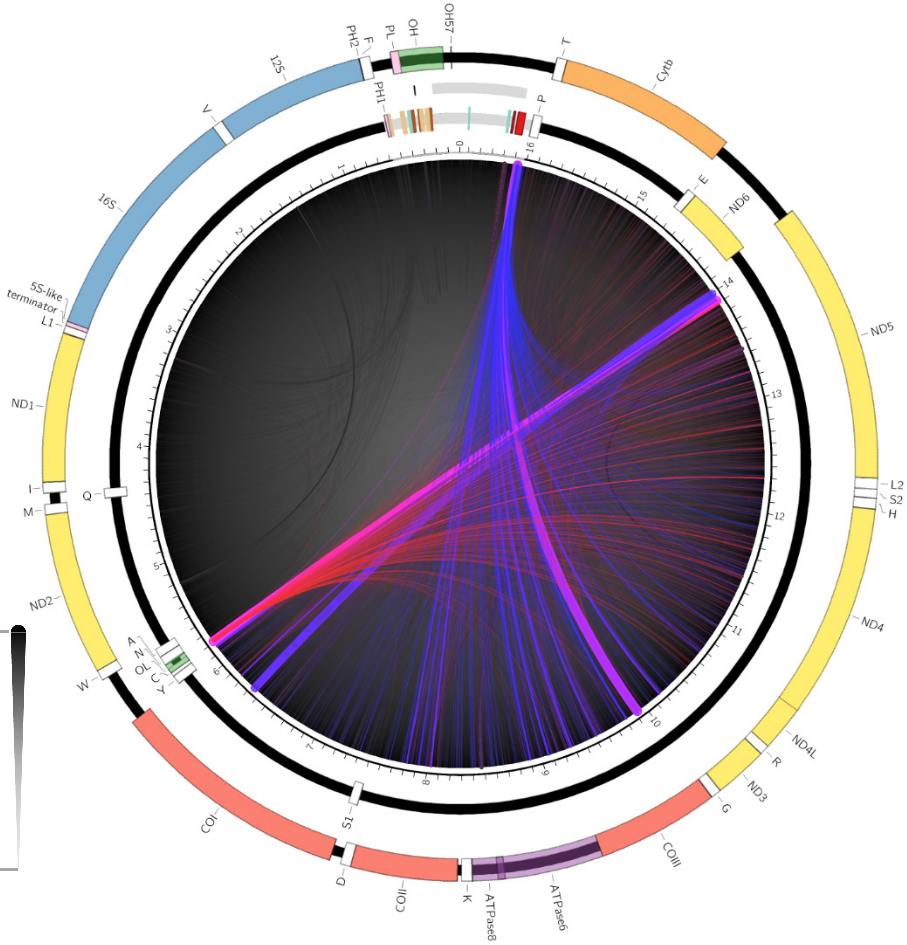

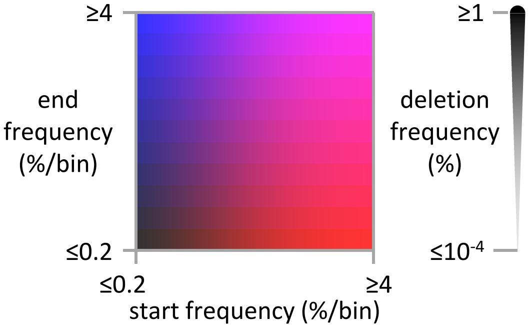

Sample	Gender	Age biopsy	Age onset	POLG genotype
M39	M	65	60	T914P;T251I/P587L

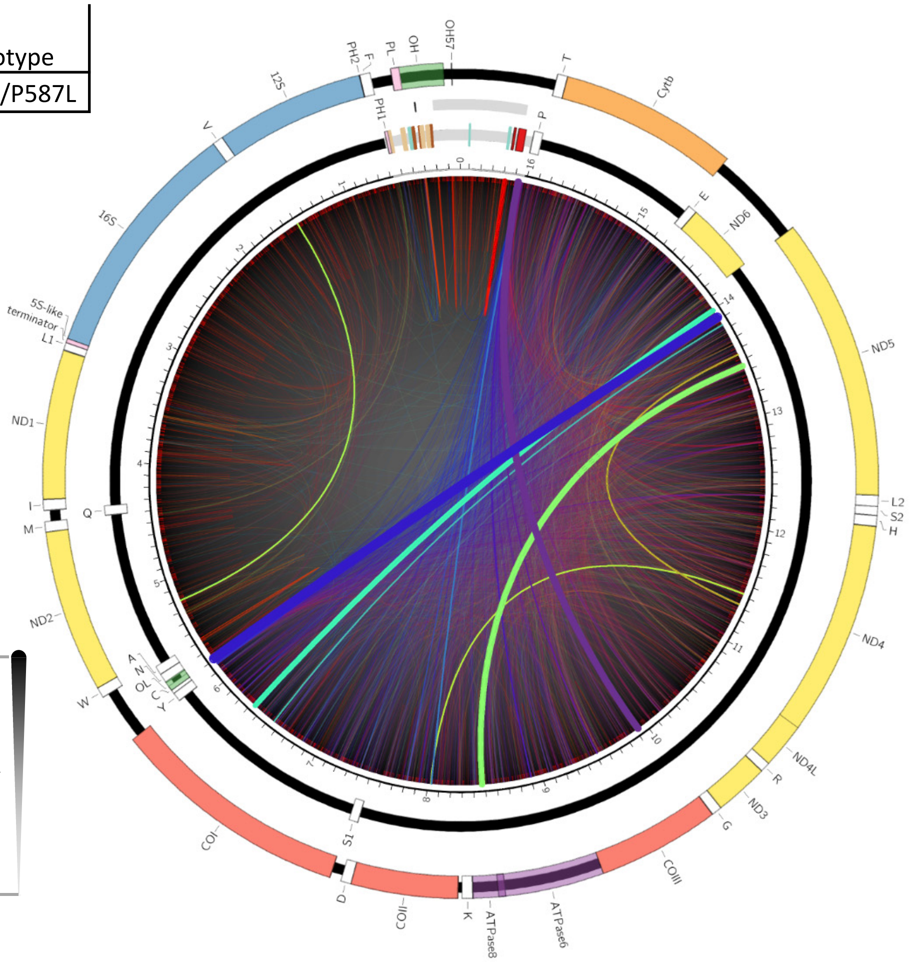

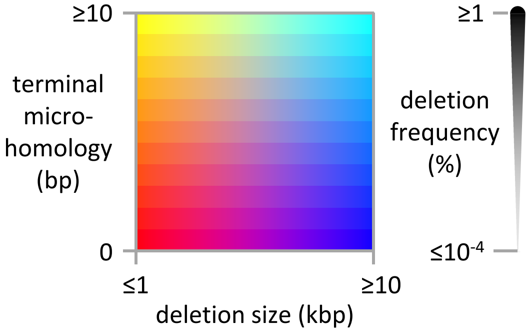

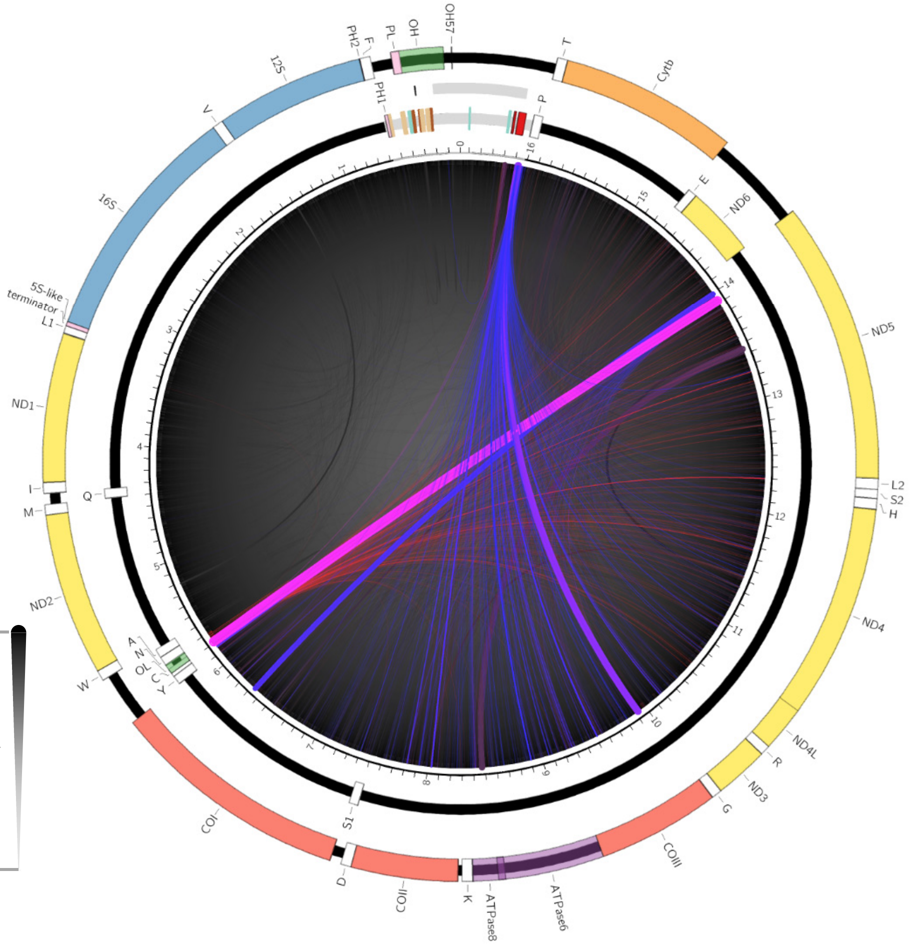

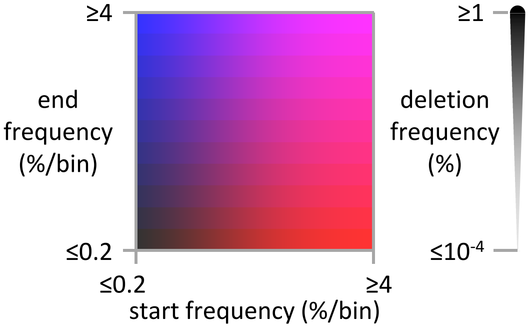

Sample	Gender	Age biopsy	Age onset	POLG genotype
M40	F	35	28	Y955C

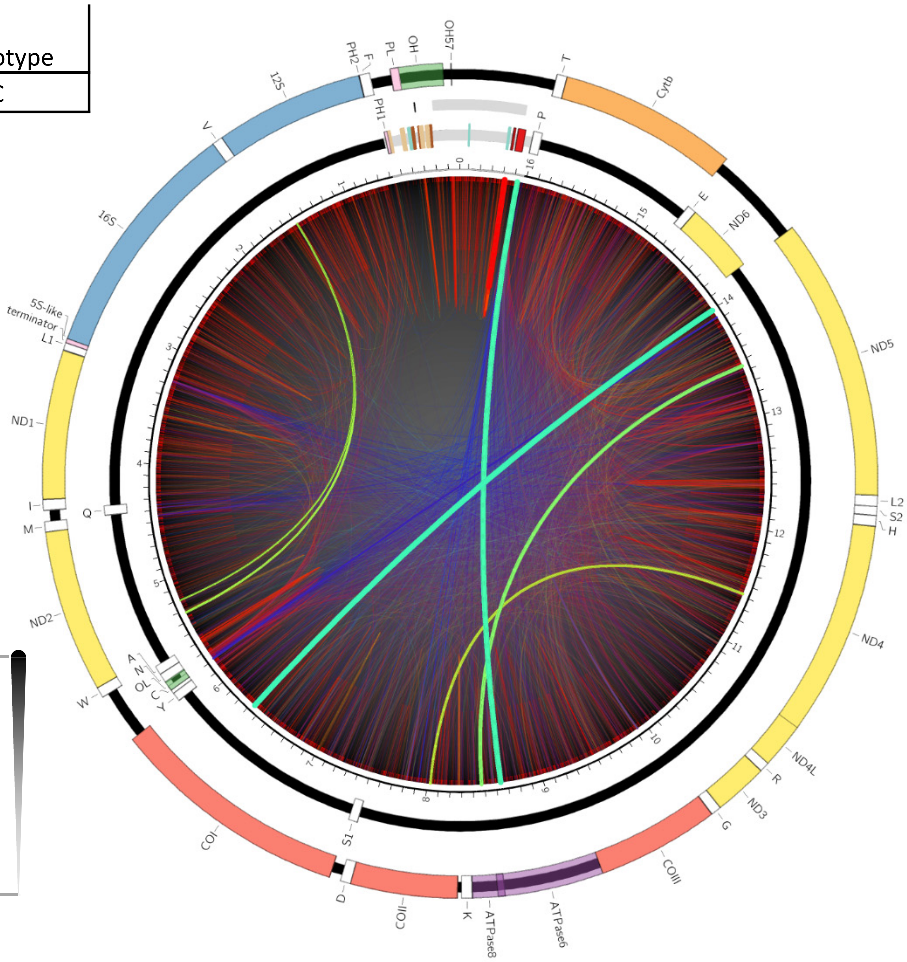

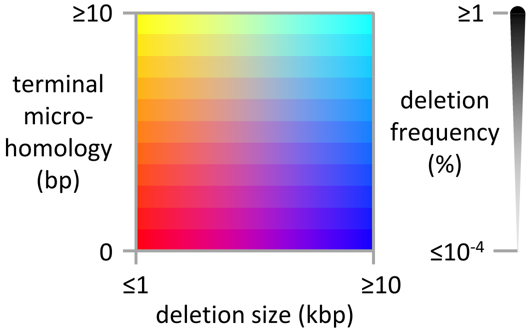

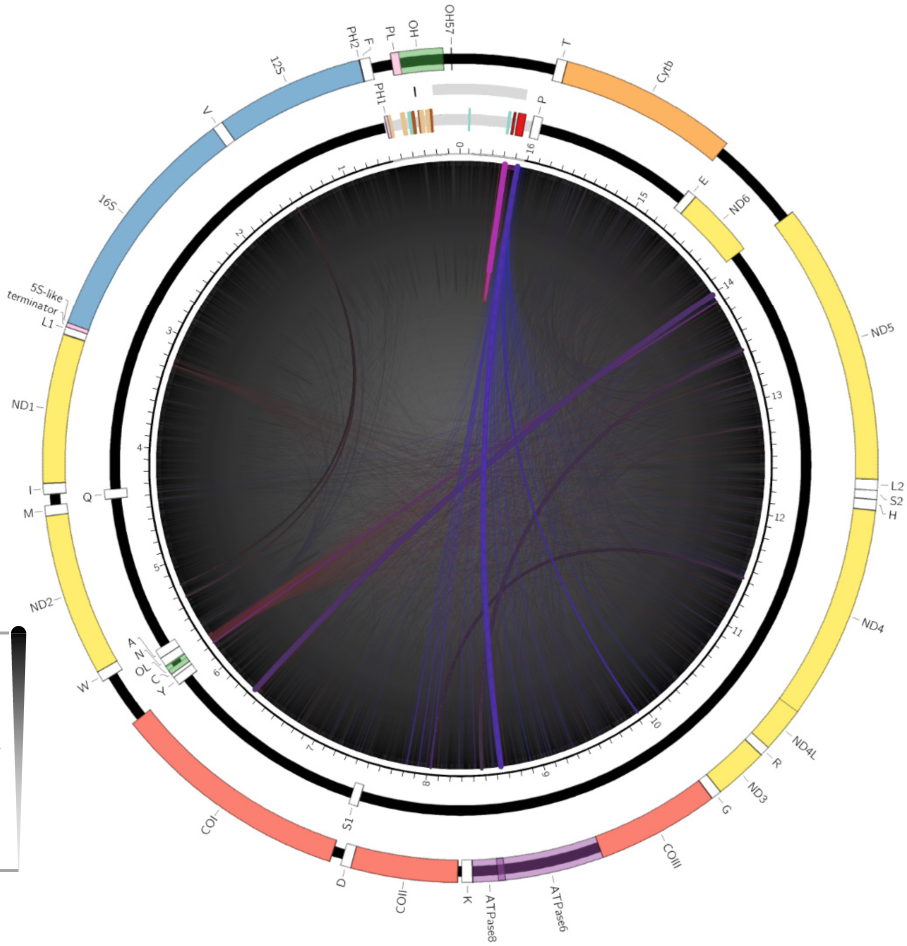

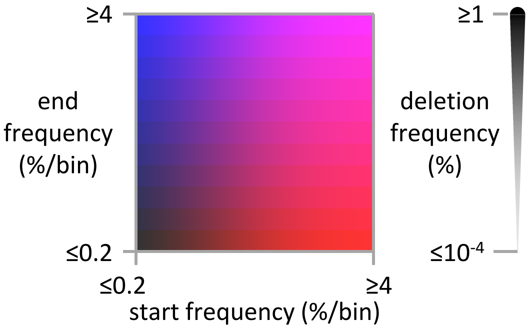

Sample	Gender	Age biopsy	Age onset	POLG genotype
M41	F	62	unk.	Y955C

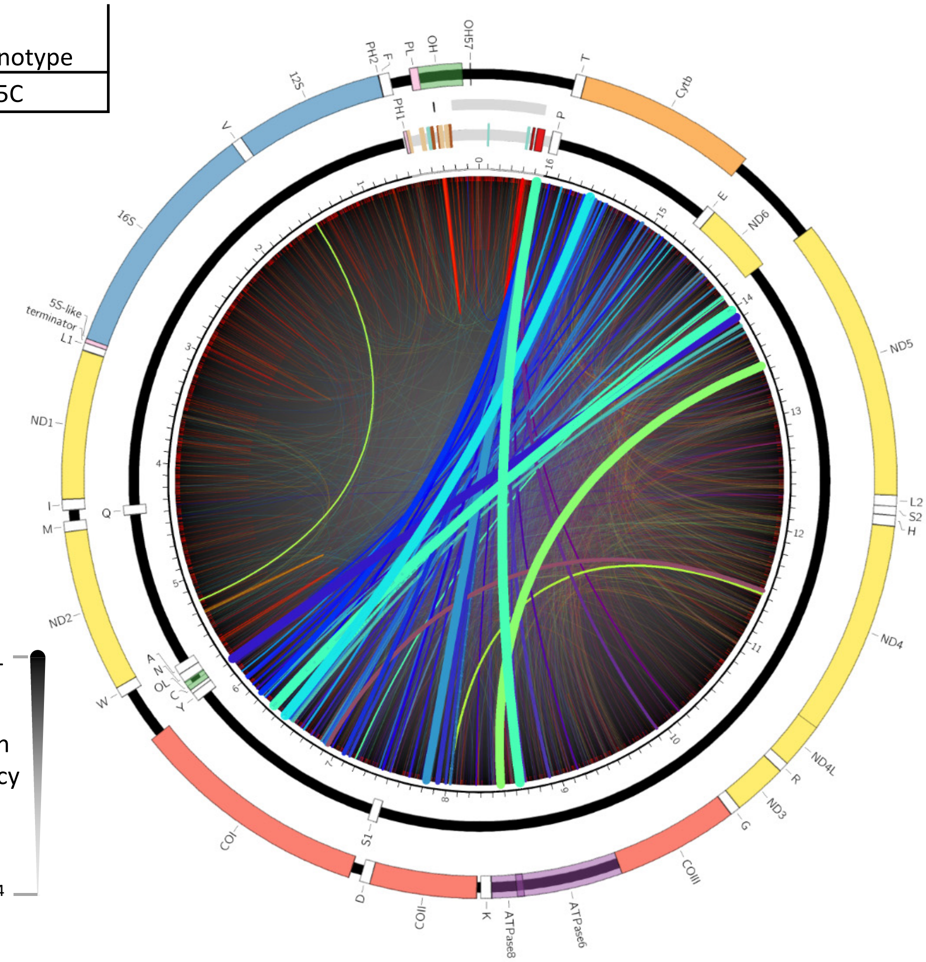

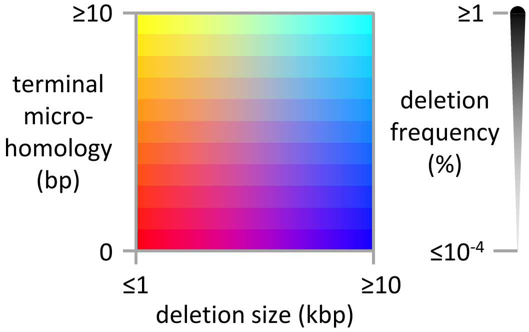

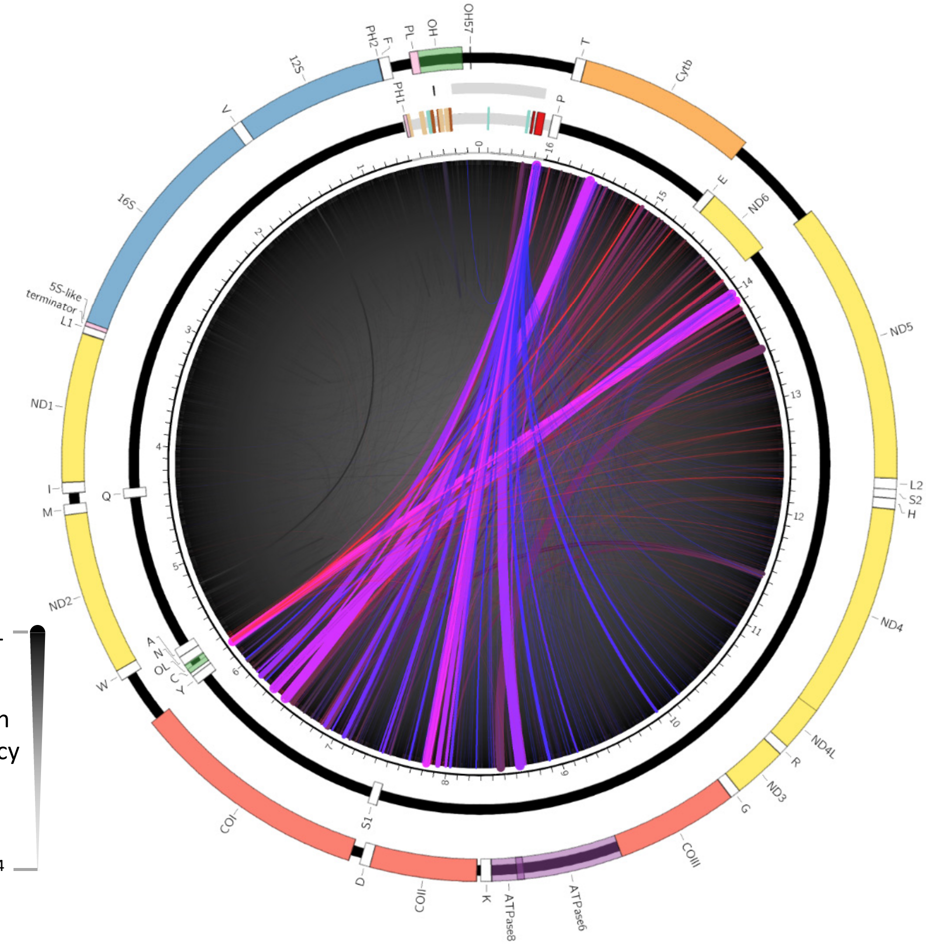

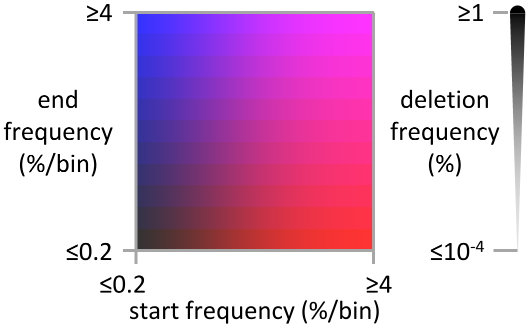

Supplement: Supplementary file 3 — Additional file 3. Arc Maps. [file 13059_2020_2138_MOESM3_ESM.pdf]
