# Supplementary material for: Ultrasensitive deletion detection links mitochondrial DNA replication, disease, and aging
Source: Genome Biol. 2020 Sep 17;21:248. doi: 10.1186/s13059-020-02138-5 (PMC7500033; doi:10.1186/s13059-020-02138-5)
Supplement: Supplementary file 4 — Additional file 4. Review history. [file 13059_2020_2138_MOESM4_ESM.docx]

Review History

**First round of review**

**Reviewer 1**

**Are you able to assess all statistics in the manuscript, including the appropriateness of statistical tests used?**

Yes, and I have assessed the statistics in my report.

**Comments to author:**

In this manuscript, the Copeland group create a novel and sensitive method, termed LostARC, to quantify deletions in mtDNA. By applying this powerful method on skeletal muscle sample from patients with PolG variants, the authors conclude that deletions are linked to replication stalling. Furthermore, they conclude that there is little purifying selection against mtDNA deletions in muscle. The study is well conducted and important. The results are interesting and the data is convincing. Although, the manuscript would benefit from some organization to make it more accessible to non-experts, it was a pleasure to read.  

Minor comments:

1.     the authors need to state the composition of the restriction enzyme cocktail and why were they chosen. Furthermore, they need to demonstrate that these enzymes are not removing potential mtDNA variants.

2.     Supplementary figures (e.g. Fig S4) need to be split into panels and have separated legends. Legends should be describing the graph as opposed to stating the interpretation.

3.     The authors make an important point that that post mitotic muscle fibers have little purifying selections against mtDNA deletions by mitophagy. To confirm their hypothesis, sequencing data of non-mitotic tissues of POLG patients or available cell lines carrying patient POLG mutations are needed as comparison.

4.     The various models of mtDNA replication are very well described, but it remains a little unclear how the link is made between the Monte Carlo simulation that best matches the observed deletion patterns and the strand-displacement replication model. It would be interesting if specific biological events used in the simulation were mentioned in the main text to better link the simulation results to data and to the model.

5.     While this could be beyond the scope of this study, it would be interesting to analyze known models of mtDNA deletions and possible perturbations (common deletion line + TLN to cut it, MGME1 KO, POLG mutant) and assess mtDNA sequencing data.  

6.     I found the notion that 1.3% of mtDNA mutations is threshold for clinical manifestation to be very compelling and it challenges the dogma in the field that 50-60% of deletions or mutations is what is needed to manifest in disease. It is important for the authors to discuss their results in the context of the previous results.

**Reviewer 2**

**Are you able to assess all statistics in the manuscript, including the appropriateness of statistical tests used?**

Yes, and I have assessed the statistics in my report.

**Comments to author:**

The manuscript outlines the development of LostArc, a novel mtDNA deletion quantification assay with a low limit of detection. Further, this work demonstrates the potential interplay between age, pathogenic mutations in polymerase γ (POLG), and mtDNA deletions. Pathogenic POLG mutations are the most common cause of mitochondrial disease and may lead to elevated levels of mutations and deletions in the mitochondrial genome during replication. This study utilized muscle biopsy specimens from people living with and without pathogenic POLG mutations, to test the novel LostArc method to detect mtDNA deletions. The authors demonstrated that the pattern of mtDNA deletions can be associated with older age and the presence of POLG variants. Additionally, the use of Monte Carlo simulations based on deletion patterns provide support for the strand displacement theory of mtDNA replication.
Identification and characterization of very low frequency mtDNA deletions of all sizes is novel and the evidence presented supporting the strand displacement theory for mtDNA replication adds relevant information to the field.


The data presented in this study are impressive, important and novel. The findings described add key insights to the field of mtDNA research, with respect to both aging and mitochondrial disease. The LostArc methodology itself is impressive and well described, along with the tools developed to analyze the data. Further, the methodology is very nicely outlined, providing clear details for others to use it in the future. However, there are several issues regarding the statistical analyses conducted, the interpretation of the data, as well as the overall length of the manuscript.
General summary comments:
1.     Assay reproducibility: As the manuscript is the first report on the LostArc assay, it would be crucial to present some data on intra- and inter-assay reproducibility. Namely, t the variability in the measures of interest when repeating the assay on the same specimen?
2.     Background estimation: The use of HEK cells (n=3, apparently technical replicates) as a mean of determining the background of the assay is problematic at best. The number of repeats is small, the choice of the cell line is not justified, and the passage number of the cell line and culture conditions could influence this result. Minimally, several independent cell lines should have been studied (e.g. biological replicates) to establish how much noise there is. Another control that should be studied is cells in which mtDNA has been completely depleted (ρ° cells). Although it is true that mtDNA damage can occur as cells are treated to eliminate mtDNA, this would nevertheless allow the authors to estimate the background generate from incomplete digestion of nuclear DNA.
3.     Large mtDNA deletions >15kb or pseudogene background? Another reason to carry out the LostArc assay on ρ° cells is that this would allow one to determine whether and how mtDNA pseudogenes may impact mtDNA deletion measures. The authors appear puzzled at observing mtDNA genomes that carry >15 kb deletions, as such deletions would eliminate the origins of replication and other critical regions. Based on typical pseudogene sizes, it would seem much more plausible that these aberrant deletions may be due to pseudogenes present in the nuclear DNA and not fully digested during the assay procedures. At a minimum, the authors must comment on this possibility.
4.     Over-interpretation of the data: The authors generated impressive data and developed equally impressive methodology to analyze and visualize said data. However, there is a tendency throughout the study to over-interpret the results. While it is possible that the authors will be proven correct in the future, the data presented here remain cross-sectional observational data, and on a relatively small study sample (n=19 and 22, less for several analyses), with statistics that are often poorly applicable or severely limited by the sample size. Because of this, while it is acceptable to discuss what these data suggest, the language must be toned down throughout the manuscript. Better controls, a larger sample, not excluding specimens from analyses, and longitudinal specimens would be required to speak as confidently as the authors do here.
5.     Study limitations not adequately identified and discussed: The authors clearly demonstrate the importance of mtDNA deletion in their study, but it seems at the expense of seeming dismissive about the relevance of mtDNA mutations. Such apparent tunnel vision is a disservice to the field as it is likely that both mtDNA deletions and mtDNA mutations play important roles in aging and disease. In fact, it is possible that mtDNA deletions are promoted by mtDNA point mutations that may disrupt the mtDNA replication machinery/regulation, a point that is completely ignored by the authors. Longitudinal studies (human or in model cell cultures) of both phenomena are required to answer this question and further increase our understanding of these processes.
6.     Manuscript length: The manuscript is very ambitious in its scope but evidently much too long at over 13,500 words, >9,000 in the intro/results/discussion sections alone. The authors should either split the manuscript in two or eliminate large sections. The simulation modeling of replication/deletion processes could potentially be a stand-alone manuscript, especially since it requires background material to be introduced in the result section. Alternatively, the PCA analyses which are fraught with issues could be eliminated entirely from the manuscript.

Specific Major Comments:
Abstract: It would be informative to somewhat define the term mtDNA ablation in the abstract as it will be new to most readers. Disease-associated instead of disease-correlated.
Page 2 Line 63. The authors state that "the accumulation of point mutations is orders of magnitudes too low to explain the phenotypes of aging." The reference cited does not implicate point mutations as insufficient to contribute to the aging phenotype, rather they challenged the traditional understanding of ROS-induced mutations, with a hypothesis that mutations contributing to the aging phenotype may be primarily due to replication errors instead, followed by the clonal amplification of said mutations. The authors should revise their statement, as there is strong evidence that mtDNA point mutations do contribute to the aging phenotype.
Page 3 Line 22. The authors indicate that the LostArc method is high throughput. It would be desirable in the discussion to come back to this point and provide further details on how many specimens can be assayed in how much time, and possibly compare its cost to other methodologies so that the reader can more fully appreciate the benefits this new assay offers.
Page 3 Line 35. The authors provide data on the study participants in Tables S1 and S2. However, many comparisons made between Gwt and Gvar only consider the age of the study subjects. Yet, the two groups are never compared with respect to age or other characteristics that could affect mtDNA deletions. For greater transparency toward the reader, and given that this is an observational human study, a demographic table is required. It should provide information in a Table format with comparisons (and p values) that allow the reader to compare the two groups with respect to their characteristics such as age, sex, BMI, ethnicity (if possible), and tobacco smoking status (if possible) as these are all factors known to influence the aging processes as well as disease. The authors should strongly consider following the checklist of the STROBE guidelines on reporting observational studies, especially in a high impact journal. https://www.equator-network.org/reporting-guidelines/strobe/
For example, more information should be provided on the inclusion/exclusion criteria for the participants. If any were excluded, the reasons why should be provided. The reasons/setting in which the study subjects were biopsied should be explained. It is rather unlikely that an 83 year old person had ACL knee surgery…Did participants provide informed consent for this study? Or were the specimens anonymized? This information should be provided.
Page 4 Line 5. The mention of "statistically different" must be accompanied with a p-value and be represented in a figure. Further, the statistical test used must be identified, whether it be in the methods or as a case-by-case basis. This statement refers to Figure 1C, but there is no statistical test conducted on this figure. Modification of figure 1C must be conducted to first identify the presence of sequencing bias after each critical step in the LostArc method, as well as demonstrate if there are any differences between Gwt and Gvar at each step (i.e. show data separately for the two groups). Figure S1A attempts to address this issue, but with large variation between specimens, it is necessary to identify if the percent of mtDNA throughout the LostArc process is comparable between groups. The authors can best represent this with a double bar graph and the appropriate statistical tests. Figure S1B should use Spearman's correlation (not Pearson's) as the data distribution does not meet the assumption for a Pearson correlation with a few outliers exerting undue influence on the regression.
Page 4 Line 9 (and elsewhere). What is "10-5.9 ± 0.09; 95% CL"? This is a highly unusual way to report summary statistics and the definition of CL is never provided. Data should be summarize as mean ± standard deviation, or mean ± 95% confidence interval if normally distributed (and these data are not always normally distributed), or median [interquartile range] if not normally distributed.
Page 4 Line 20-21. The authors claim that the correlation of the data in Figure 2A has an R2 < 0.002, but do not identify what test was used to generate that number. Correlation tests must show a p-value and either a Pearson's r or Spearman's rho.
Page 4 Line 23-28. These data are not displayed anywhere in the manuscript, yet are referred to in the result section and the discussion. The authors must make a figure to show these results and the statistical tests.
Page 4 Line 40-45. The Kolmogorov-Smirnov test is not useful to interpret the data in Figure 2C. To identify a relationship between deleted mtDNA and age, a correlation should be performed. Further, for the reader to interpret the t-tests performed on the binned age data, it must be shown in a separate figure, or show a dotted line that would separate the two age groups.
Page 4 Line 46-47. The relationship between age and mtDNA copy number is not demonstrated anywhere in the manuscript. Figure S1A does not address this relationship, and thus, as stated above, a figure must be included, or this statement removed.
Page 5 Line 2-14. This paragraph is all speculation as it does not directly follow that "ablation level must have been lower at the age of onset of symptoms." The authors can mention their assumption, but Figure 2E is too weak of an estimation to include and should be removed. Further, the mention of this analysis in the discussion on Page 13 Line 4-7 should also be removed.
Page 5 Line 20. In order to clearly show that "oriL and the 7S-DNA sequence are strongly preserved in Gvar and Gwt muscles", the authors should make the ablation patterns available for all specimens tested, not just four of them represented in Figure 2B. This can be done with an additional supplemental figure.
Figure 3C. This reviewer applauds the author for their extensive data visualization. However, they should consider using a categorical colour map rather than a continuous one for Figure 3C and other similar figures. As is the figure is too complicated and chaotic; the green line denoting the common deletion is not visible and the message gets lost in the complexity. The Blue colour in Figure 3D is invisible due to poor contrast.  Consider using a lighter blue.
Page 5 Line 51-54. An example of the COX histology images used for analysis would be desirable. Further, in order to determine "best correlation", correlation tests must be conducted and reported as p-values and respective Pearson's or Spearman's rho in Figure S3B.
Page 5 Line 62 - Page 6 Line 4. The authors claim that "since small deletions are markedly more frequent in muscle samples than in HEK cultures, they are included in overall deletion load and ablation calculations after HEK background subtraction". To my understanding, there are just as many small (>100bp) deletions in Figure S4 than in all other specimens analyzed. The authors should clarify this statement. Secondly, Figure 2A should not be referenced here as it is not relevant.
Page 5 Line 30. The authors state that "the failure to more fully retain these OXPHOS sequences implies incomplete or absent mitophagy in postmitotic muscle fibers." This statement would belong in the discussion rather than the result section. Further, is it not possible that mitochondria contain multiple copies of mtDNA that complement each other functionally so that membrane potential is maintained despite the presence of mtDNA deletions? After all, the same argument could be made about mtDNA mutations. In the absence of intermembrane potential data supporting this statement, it is too strong and should be toned down. The absence of evidence is not the evidence of absence…
Page 6 Line 36. A previous age-binning method was proposed prior to this one, the authors must choose one and use it for the entirety of the manuscript.
Page 6 Line 38. The authors note that the "following differences are significant (α = 0.05)", but do not indicate what statistical tests were performed. Further, the authors should modify Figure 3A to represent the age-binning used for the statistical tests and create a duplicate graph with the Gvar specimens to allow for visual comparison between groups.
Page 8 Line 7-19. The data in the paragraph do not refer to a figure. The authors must address this and make the statistical considerations obvious on a figure.
Page 9 Line 22 - Page 10 Line 9. The PCA portion of this manuscript is flawed and should be removed. The PCA as is does not specify all the variables considered and is only conducted on a subset of specimens. Such specimen "selection" is inappropriate as it most likely introduced bias that influences the results presented, something the authors hint at themselves. Typically, PCA analysis requires a larger sample size, especially when considering multiple different variables. The removal of this section would also lead to the removal of the following: all three mentions of PCA components on Page 10 Line 45-58, all mention of PCA analysis on Page 13 Line 52 - Page 14 Line 5, the paragraph on Page 14 Line 9-22, PCA in the methods on Page 21 Line 54 - Page 22 Line 24, Figure 4B-G, and any mention of PCA in the figure legends. It would also reduce the length of the manuscript, something needed, and perhaps some of the more interesting data presented in supplement could be brought back in the main text.
Page 11 Line 2-22. This section from "The 73-3' terminus…" to "counterclockwise directions" should be removed as it is unnecessary in the results, but the sentence "Each satisfied the following… in order to avoid overfitting" should be added to the Monte Carlo methods section.
Page 11 Line 58-61. As mentioned before, the authors claim "mtDNA copy number did not vary significantly" between Gwt and Gvar individuals, but there is no data in the results to support this. The authors must add a figure with this information or remove the statement.
Page 12 Line 28. The authors rightly point out that "Methods for further reducing or eliminating amplification require further study". Indeed, this remains a potential limitation in the present study, but is not discussed as such.

Minor Comments:
Page 3 Line 49-63. This section should be re-worked as it repeats the methods and what is shown in Figure 1B. The authors can simply remove the section and replace it with a sentence along the lines of "LostArc was conducted as outlined in Fig. 1b. For more details refer to the Materials and Methods."
Figure 1B. The authors should identify how the 30% cut-off for repetition of the nuclease treatment and purification was determined in Step 4b. Further, they should specify the number of specimens that underwent repetition in both the wild type POLG (Gwt) and variant POLG (Gvar) groupings.
Page 4 Line 3-4. The authors should define "very rare" and "ultra-rare" deletant species with the frequencies they represent.  
Figure 2. The authors should consider removing the randomly highlighted Gwt points (M02 & M17) as well as highlight all different Gvar mutations or none at all.
Page 4 Line 60-61. May be better suited in the discussion or removed.
Page 5 Line 22-32. May be better suited in the discussion or removed.
Page 5 Line 37-48. This section from "First, we assume…" to "examples in Figs. S4 and S5" can be removed.
Page 8 Line 30. The authors should modify the wording of "in hope of uncovering" to "in an attempt to uncover".
Page 8 Line 33-40. This portion of the paragraph summarizes the methodology and is not necessary. The authors should remove this section, move "The Bubble and Arc Plots… (see Methods)" to the next section, and rename that section "Unsupervised exploratory analysis of mtDNA deletion patterns separate samples by age and POLG genotype."
Page 10 Line 13-43. The very detailed description of the mtDNA replication modes is unnecessary as it is clearly demonstrated in Figure 5. This could be removed.
Page 10 Line 62. The authors should make it obvious in Figure S6A which mtDNA replication mode each Monte Carlo model is representing by adding a column to the table.
Page 17 Line 48-60. This paragraph may be better suited in the results.
Figure 4A. The Blue colour depicting WT POLG and the Blue depicting POLG A467T are too similar.
Figure 5I. Remove the line between two points.
Figure legends should stand alone and not constantly refer to other figures.
Please ensure consistency in figure legends and x- and y- axes. State the statistical tests utilized in each figure and whether any errors bars present represent

**Authors Response**

**Point-by-point responses to the reviewers’ comments:**

Reviewer reports:

Reviewer #1:

In this manuscript, the Copeland group create a novel and sensitive method, termed LostARC, to quantify deletions in mtDNA. By applying this powerful method on skeletal muscle sample from patients with PolG variants, the authors conclude that deletions are linked to replication stalling. Furthermore, they conclude that there is little purifying selection against mtDNA deletions in muscle. The study is well conducted and important. The results are interesting and the data is convincing. Although, the manuscript would benefit from some organization to make it more accessible to non-experts, it was a pleasure to read.

Minor comments:

1.	the authors need to state the composition of the restriction enzyme cocktail and why were they chosen. Furthermore, they need to demonstrate that these enzymes are not removing potential mtDNA variants.

*Response: The restriction enzymes (**AgeI, DraIII, FspI, PshAI, PspXI, SexAI, XmaI) are listed on page 17, in the Methods section entitled “mtDNA enrichment,” though their final concentrations were not stated. This oversight has been corrected. The new sentence now includes “… to final concentrations recommended by the manufacturer …”*

*As to removing potential mtDNA variants, this is a risk, but one that we judged to be acceptable. These seven enzymes have a combined 334 recognition sequences (due to degenerate base positions). There are 845,706 potential mtDNA deletion junctions that would create one of these sequences, representing 0.3% of the > 274 million possible junctions.*

2.	Supplementary figures (e.g. Fig S4) need to be split into panels and have separated legends. Legends should be describing the graph as opposed to stating the interpretation.

*Response: Thank you for catching the inadequacy of this legend. The interpretation has been shortened and moved to the Results section (p. 4, l. 25). Fig. S4-5 are intended as examples. Equivalent reports are available for each sample (n > 30; LostArc Reports supplemental PDF). Therefore, rather than breaking the panels into separate figures, we have chosen to rewrite the legend to clarify what each panel represents and how it relates to data in other tables and figures.*

3.	The authors make an important point that that post mitotic muscle fibers have little purifying selections against mtDNA deletions by mitophagy. To confirm their hypothesis, sequencing data of non-mitotic tissues of POLG patients or available cell lines carrying patient POLG mutations are needed as comparison.

*Response: Unfortunately, no other tissues were available from the participants in this study. We would like to test all manner of POLG and non-POLG variants in cell cultures and other tissues from patients, and we hope to do so in the future, but our current lack of wet-lab access and the current immensity of the manuscript render such experiments outside scope of this study. This comment is related to our response to Reviewer 2, Specific Major Comment #16 and Reviewer 2, General Summary Comment #2.*

4.	The various models of mtDNA replication are very well described, but it remains a little unclear how the link is made between the Monte Carlo simulation that best matches the observed deletion patterns and the strand-displacement replication model. It would be interesting if specific biological events used in the simulation were mentioned in the main text to better link the simulation results to data and to the model.

*Response: We agree. The main results now state (p. 11): “Two selected schemata were independent of mtDNA replication origins, as if deletions were triggered by attempted repair of random strand breaks or non-coding lesions without (Sim. 1) or with (Sim. 2) a dependence on deletion length. Four simulations (Sims. 3-6) were based on recovery of replication intermediates generated during previously proposed replication modes. Two (Sims. 3 and 4) assume formation of deletions during unidirectional H-strand replication from 7S-3’. One (Sim. 3) models deletions initiated during continuous H-strand synthesis as in the original RITOLS/Bootlace synchronous synthesis mode or the first stage of asynchronous replication in either the Rolling-Circle or Strand-Displacement synthesis modes. Another (Sim. 4) models deletions formed during original RITOLS/Bootlace DNA mode of synthesis without selection for oriL. The last two (Sims. 5 and 6) assume deletions arise during synchronous bidirectional replication from 7S-3’ (i.e. Strand-Coupled synthesis) or during asynchronous unidirectional synthesis from 7S-3’ and oriL (i.e. the Strand-Displacement Model), respectively.”*

5.	While this could be beyond the scope of this study, it would be interesting to analyze known models of mtDNA deletions and possible perturbations (common deletion line + TLN to cut it, MGME1 KO, POLG mutant) and assess mtDNA sequencing data.

*Response: As stated above, we intend to test all manner of variants in all manner of settings in the future. For now we must consider such experiments outside scope of this study.*

6.	I found the notion that 1.3% of mtDNA mutations is threshold for clinical manifestation to be very compelling and it challenges the dogma in the field that 50-60% of deletions or mutations is what is needed to manifest in disease. It is important for the authors to discuss their results in the context of the previous results.

*Response: We agree that our estimated minimal threshold for clinical manifestation is compelling, and we believe the prevailing dogma should be considered with a view toward recent advances in sequencing technology. Southern blot analysis can identify single and multiple frequent mtDNA deletions with deletion heteroplasmy as high as 30-60% in muscle samples from affected patients (e.g. PMID 7719341). QPCR-based methods are more sensitive and have a wider dynamic range for determination of deletion heteroplasmy. Digital drop PCR can easily assess specific, high frequency mtDNA deletions in tissues from mitochondrial disease patients, and deletion heteroplasmy levels of >50-75% due to clonal expansion (PMID 30076399) and 68% from maternal inheritance of a single large scale deletion (ref. 9: PMID 31479473) have been reported recently. Quantifying the frequency of very rare mtDNA deletions was not possible until the development of digital drop PCR, which partitions and amplifies samples to approach the precision and sensitivity of single molecule PCR for specific deletions. LostArc avoids amplification and enriches mtDNA by selectively degrading linear nuclear DNA with nucleases. LostArc’s high sequencing depth permits simultaneous quantification of high frequency deletions and deletions as rare as 10-6 across the whole mitochondrial genome. In most of our patient samples, we find that >70% of individual mtDNA genomes harbor a deletion. This value aggregates the population of rare, infrequent, and frequent mtDNA deletions in a sample. The combined deletion heteroplasmy is perfectly consistent with previous clinical findings that mtDNA deletion loads of 50-60% are needed to manifest disease.*

*As defined in detail in the manuscript, the “mtDNA ablation” level combines the frequencies and lengths of all detected deletions to estimate the degree of mtDNA lost to deletions within a given sample. We believe that mtDNA ablation is superior to deletion heteroplasmy fraction as a metric for gauging the extent of mtDNA deletions needed to manifest disease.*

Reviewer #2:

The manuscript outlines the development of LostArc, a novel mtDNA deletion quantification assay with a low limit of detection. Further, this work demonstrates the potential interplay between age, pathogenic mutations in polymerase γ (POLG), and mtDNA deletions. Pathogenic POLG mutations are the most common cause of mitochondrial disease and may lead to elevated levels of mutations and deletions in the mitochondrial genome during replication. This study utilized muscle biopsy specimens from people living with and without pathogenic POLG mutations, to test the novel LostArc method to detect mtDNA deletions. The authors demonstrated that the pattern of mtDNA deletions can be associated with older age and the presence of POLG variants. Additionally, the use of Monte Carlo simulations based on deletion patterns provide support for the strand displacement theory of mtDNA replication.

Identification and characterization of very low frequency mtDNA deletions of all sizes is novel and the evidence presented supporting the strand displacement theory for mtDNA replication adds relevant information to the field.

The data presented in this study are impressive, important and novel. The findings described add key insights to the field of mtDNA research, with respect to both aging and mitochondrial disease. The LostArc methodology itself is impressive and well described, along with the tools developed to analyze the data. Further, the methodology is very nicely outlined, providing clear details for others to use it in the future. However, there are several issues regarding the statistical analyses conducted, the interpretation of the data, as well as the overall length of the manuscript.

General summary comments:

1.	Assay reproducibility: As the manuscript is the first report on the LostArc assay, it would be crucial to present some data on intra- and inter-assay reproducibility. Namely, t the variability in the measures of interest when repeating the assay on the same specimen?

*Response: Thank you for this excellent suggestion. Fig. S2 already showed bubble plots for two subsamples of the muscle biopsy M22. Aside from the control plasmid spikes in S2b, only the closest visual inspection will find the variation between the two, except along the diagonal, where the smallest deletions lie (where sequencing errors are most prominent).*

*Figure S2 has been updated to show two additional direct comparisons between replicates. The legend now includes “D-E) Semi-transparent blue circles represent frequencies of deletions detected in two subsamples of muscle sample M22. Black circles represent the same data (above 10-6), binned by order of magnitude and averaged (log scale). Error bars represent standard deviations. Linear regressions (black lines; equation and R2 inset) are for the averaged data. D) Deletions detected in both M22 muscle subsamples A (DNA subsample A) and B (n = 13,490). These samples were forked before Step 1 in Fig. 1b. E) Deletions detected in both M22 muscle subsample A, DNA subsamples A and B (n = 7,863). These samples were forked before Step 2 in Fig. 1b.”*

*As you can see, “frequencies of individual deletions correlate well between DNA and tissue subsamples (Fig. S2d and e; slopes and R2 values very near 1)” (p. 17, l. 36), indicating excellent reproducibility. Relative variance does obviously increase with the inverse of deletion frequency, which is why we were careful to only discuss the rarest deletions as a population, as stated in the Results (e.g. p. 4, l. 10) and now also in the Methods (p. 21, l. 6).*

2.	Background estimation: The use of HEK cells (n=3, apparently technical replicates) as a mean of determining the background of the assay is problematic at best. The number of repeats is small, the choice of the cell line is not justified, and the passage number of the cell line and culture conditions could influence this result. Minimally, several independent cell lines should have been studied (e.g. biological replicates) to establish how much noise there is. Another control that should be studied is cells in which mtDNA has been completely depleted (ρ° cells). Although it is true that mtDNA damage can occur as cells are treated to eliminate mtDNA, this would nevertheless allow the authors to estimate the background generate from incomplete digestion of nuclear DNA.

*Response: The LostArc pipeline has very high reproducibility. Please see Figure S2, where we compare tissue subsamples of M22 and also repeated measurements of deletions in control plasmids. The latter is especially telling, given different plasmid sizes (~4.2 - 7.5 kbp), deletion sizes (304 – 3,652 bp), input concentrations (10-5 - 10-1), and sample milieus. Thus, we are confident in our measures of the HEK293 deletion spectrum with three replicates.*

*The reviewer is right that HEK293 cells provide a nonideal background. We would prefer a matched patient tissue that is mitophagy competent and easily obtainable, preferably by less painful means than muscle biopsy. In the meantime, HEK cells have active mitophagy, wt mtDNA, and are inexpensive and familiar to many researchers. Most importantly, the three HEK samples that we used have lower ablation levels than the Gwt muscle samples. HEK mtDNA is appropriate for estimating mappability factors (see calculations in Methods) and for setting an upper bound on sequencing noise. Rho(0) cells lack mtDNA, so mtDNA sequences and apparent deletions would come only from mtDNA sequences in nuclear DNA (NUMTs; please see Response 3 below for our perspective on NUMT noise). Rho(0) cells would therefore be inappropriate for estimating mappability factors or setting upper bounds on noise of sequencing circular mtDNA.*

3.	Large mtDNA deletions >15kb or pseudogene background? Another reason to carry out the LostArc assay on ρ° cells is that this would allow one to determine whether and how mtDNA pseudogenes may impact mtDNA deletion measures. The authors appear puzzled at observing mtDNA genomes that carry >15 kb deletions, as such deletions would eliminate the origins of replication and other critical regions. Based on typical pseudogene sizes, it would seem much more plausible that these aberrant deletions may be due to pseudogenes present in the nuclear DNA and not fully digested during the assay procedures. At a minimum, the authors must comment on this possibility.

*Response: Though few NUMTS are even long enough to mimic a mtDNA deletion of over 100 bp, we were initially worried about confounding NUMTs in undigested nuclear DNA. Our first attempt to establish a background used plasmids with mtDNA inserts, which we purified from bacteria. Unfortunately, the plasmids exhibited much higher deletion rates than anticipated given known PCR and Illumina errors. The deletions preserved drug resistance genes and plasmid origins, implying selective pressure working on true deletions made by bacterial systems rather than later sequencing errors. Thus, we switched to HEK cell mtDNA (reasons stated above).*

*Enrichment largely solves the problem of deletions in NUMTs. After enrichment, on average, mtDNA makes up > 60% of the DNA that goes into the library preparation (Fig. 1c). Therefore, in an average sample there are (0.6/16571)/(0.4/3,000,000,000) = 271,559 mtDNA circles for every 1 copy in a given NUMT. If a NUMT deletion was present in 100% of sampled nDNA (e.g. a heterozygous germline NUMT), its apparent frequency would be 1/271,559 = 3.7 x 10-6, which is right at the detection limit for our lower depth samples. Compare that number to the cloud of more frequent deletions in the deletion size spectrum of high-depth Gwt sample M01 (17 year old donor; Fig. S5). When discussing overall ablation, each NUMT deletion would be even less significant. The longest detectable deletion (16,571 nt - 300 nt = 16,271, limited by Illumina read length) would yield an ablation of (~16,271/16,571)/271,559 = 3.6 x 10-6, two orders of magnitude less than the lowest observed ablation in a muscle sample. The worst enrichment in this study was for sample M19 (Fig. S1a). A NUMT deletion in that sample would be present at no more than 3.7 x 10-5, as compared to an overall ablation level of 9.7 x 10-2.*

4.	Over-interpretation of the data: The authors generated impressive data and developed equally impressive methodology to analyze and visualize said data. However, there is a tendency throughout the study to over-interpret the results. While it is possible that the authors will be proven correct in the future, the data presented here remain cross-sectional observational data, and on a relatively small study sample (n=19 and 22, less for several analyses), with statistics that are often poorly applicable or severely limited by the sample size. Because of this, while it is acceptable to discuss what these data suggest, the language must be toned down throughout the manuscript. Better controls, a larger sample, not excluding specimens from analyses, and longitudinal specimens would be required to speak as confidently as the authors do here.

*Response: We appreciate the reviewer’s kind words about our data, methods and visualizations. We address several general criticisms in turn:*

“relatively small study sample”

*While we agree that more samples are always useful, we would like to point out that this is the largest study of its kind, with multiple Gwt samples and both donor genders in each bi-decadal age cohort (Fig. 3a). This includes 10 Gwt samples from donors aged > 60 years (where ablation increases linearly; Fig. 2c). In addition, we were careful to outline our assumptions, to enumerate all subsets, to restrict discussion to patterns with statistical significance after accounting for sample size, and to showcase alternative explanations. For instance, we avoid speculating about the mechanisms linking different Gvar genotypes to differences in observed deletion spectra, instead focusing on their commonalities and their relationships with the Gwt set.*

“statistics”

*Statistical critiques are handled point-by-point below.*

“Better controls”

*With apologies, we stand by our controls. If the reviewer is referring to the choice of HEK as background, please see the HEK discussion above. If the reviewer is referring to the cross-sectional use of Gwt samples, please see the discussions of longitudinal experiments below.*

“longitudinal specimens would be required to speak as confidently”

*We also point out the need for longitudinal specimens (Discussion, final paragraph), but cross-sectional analyses may be all that we as a field get to discuss for quite some time, at least in muscle fibers. Sampling the same muscle fibers twice is difficult, and repair of the first biopsy may change the deletion spectra of subsequent biopsies (see discussion of myosatellites and repair; p. 15). Most importantly, given that the interesting changes occur over decades, we are unable to complete longitudinal human studies during a feasible post-doctoral tenure. On the other hand, if the reviewer knows of available longitudinal samples it would be difficult to overstate how excited we would be to collaborate with the caretakers of such samples.*

5.	Study limitations not adequately identified and discussed: The authors clearly demonstrate the importance of mtDNA deletion in their study, but it seems at the expense of seeming dismissive about the relevance of mtDNA mutations. Such apparent tunnel vision is a disservice to the field as it is likely that both mtDNA deletions and mtDNA mutations play important roles in aging and disease. In fact, it is possible that mtDNA deletions are promoted by mtDNA point mutations that may disrupt the mtDNA replication machinery/regulation, a point that is completely ignored by the authors. Longitudinal studies (human or in model cell cultures) of both phenomena are required to answer this question and further increase our understanding of these processes.

*Response: We agree with the reviewer that mtDNA deletions are related to mtDNA point mutations. Our leading hypothesis for the mtDNA patterns reported in our study is that Pol gamma stalls following mis-insertion of an incorrect nucleotide to make a base-base mismatch or upon encountering a miscoding adduct. In both cases, continued synthesis creates point mutations in the next round of replication. Further, a deletion will result if the stalled replisome releases the template and relocates downstream, or also if synthesis restarts at a distal site and the resulting termini are ligated.*

*We imply no disrespect to researchers studying mtDNA point mutations. Please see further discussion under Specific Major Comments #2, below.*

6.	Manuscript length: The manuscript is very ambitious in its scope but evidently much too long at over 13,500 words, >9,000 in the intro/results/discussion sections alone. The authors should either split the manuscript in two or eliminate large sections. The simulation modeling of replication/deletion processes could potentially be a stand-alone manuscript, especially since it requires background material to be introduced in the result section. Alternatively, the PCA analyses which are fraught with issues could be eliminated entirely from the manuscript.

*Response: We agree that the manuscript is long. If the editor would prefer some parts to be moved to the Supplement, we would be amenable. We considered splitting the paper into two or more, and early outlines assumed multiple manuscripts. However, in the course of writing and in consultation with all of the authors, it became apparent that the various manuscripts would need to reference one another in such a way that would require simultaneous review and publication in the same journal as back to back publications. Thus, we chose to submit one unified manuscript of epic size.*

*Nevertheless, we have combined and reduced two sections in the Results (“Unsupervised exploratory analysis of mtDNA deletion patterns” and “Deletion patterns separate samples by age and POLG genotype”) and directed the reader to find pertinent details in the Methods section.*

*Also, we believe Reviewer 2 misunderstood the basis for our PCA analysis. Please see a discussion of our PCA analysis, below.*

Specific Major Comments:

1. Abstract: It would be informative to somewhat define the term mtDNA ablation in the abstract as it will be new to most readers. Disease-associated instead of disease-correlated.

*Response: Thank you for this suggestion. The abstract has been edited to include this definition (“Ablation, the deleted mtDNA fraction,…”) while remaining under the word count cap.*

2. Page 2 Line 63. The authors state that "the accumulation of point mutations is orders of magnitudes too low to explain the phenotypes of aging." The reference cited does not implicate point mutations as insufficient to contribute to the aging phenotype, rather they challenged the traditional understanding of ROS-induced mutations, with a hypothesis that mutations contributing to the aging phenotype may be primarily due to replication errors instead, followed by the clonal amplification of said mutations. The authors should revise their statement, as there is strong evidence that mtDNA point mutations do contribute to the aging phenotype.

*Response: We state in the Introduction that, alongside deletions, point mutations “accumulate with age and have been implicated in normal aging and age-related pathologies”. Certainly, both deletions and mtDNA point mutations in the germline or occurring very early in tissue development are expected to have phenotypic effects. We suspect that the “strong evidence that mtDNA point mutations do contribute to the aging phenotype” may refer to the original POLG mutator mouse results (Vermulst et al. 2008; ref. #30). The work therein was quite good but there was some controversy about the level of point mutations found therein (reviewed Stumpf, et al. PMID: 20927567 ). However, more good work from the same group showed that “point mutations do not limit the natural lifespan of mice” (Vermulst et al. 2007; ref. #25), and we have added the Vermulst et al., Nat Genetics 2007 reference to this section to clarify our point.*

*However, Kennedy et al. (ref. #24) used ultra-accurate Duplex-seq to show that even in old age TOTAL point mutations (not just those attributable to ROS) were too infrequent to explain the phenotypes of aging in another postmitotic tissue (brain). In our samples, point mutations were found at frequencies consistent with sequencing errors, regardless of age or genotype, including the known POLG mutator variant Y955C. Ablation, on the other hand, was higher in older samples and was sufficient to explain such phenotypes as COX deficiency. We do not comment on point mutation rates in our samples because of the technical, though obviously remote, possibility that point mutations in our muscle samples are many orders of magnitude more frequent than in brain but still below standard Illumina sequencing errors (> 0.002 per bp).*

3. Page 3 Line 22. The authors indicate that the LostArc method is high throughput. It would be desirable in the discussion to come back to this point and provide further details on how many specimens can be assayed in how much time, and possibly compare its cost to other methodologies so that the reader can more fully appreciate the benefits this new assay offers.

*Response: The throughput depends on the desired depth and available sequencers. Our sequencing was done with an Illumina NextSeq. This was sufficient given the relatively small scale of the study. Our core has since acquired a NovaSeq 6000, with which they have demonstrated over 20 billion 2x150 bp paired-end reads per flowcell at a cost of $25,000. This is sufficient for about > 100 samples at the average depth and degree of mtDNA enrichment reported here (< $250 / sample). This meets our definition of high throughput. However, given that we have not completed validation of LostArc samples on the NovaSeq, and also given that our pricing may not be representative, we would prefer not to discuss these details in the paper.*

4. Page 3 Line 35. The authors provide data on the study participants in Tables S1 and S2. However, many comparisons made between Gwt and Gvar only consider the age of the study subjects. Yet, the two groups are never compared with respect to age or other characteristics that could affect mtDNA deletions. For greater transparency toward the reader, and given that this is an observational human study, a demographic table is required. It should provide information in a Table format with comparisons (and p values) that allow the reader to compare the two groups with respect to their characteristics such as age, sex, BMI, ethnicity (if possible), and tobacco smoking status (if possible) as these are all factors known to influence the aging processes as well as disease. The authors should strongly consider following the checklist of the STROBE guidelines on reporting observational studies, especially in a high impact journal.

<https://www.equator-network.org/reporting-guidelines/strobe/>

For example, more information should be provided on the inclusion/exclusion criteria for the participants. If any were excluded, the reasons why should be provided. The reasons/setting in which the study subjects were biopsied should be explained. It is rather unlikely that an 83 year old person had ACL knee surgery…Did participants provide informed consent for this study? Or were the specimens anonymized? This information should be provided.

*Response: Aside from age and gender, demographic data on donors of Gwt samples was anonymized. We now have included ethnicity in the sequence data deposition to dbGaP. Due to the nature of consent forms for the collection of samples, we do not have other information except age, gender and ethnicity. We would love to study the effects of exposures, exercise, BMI, etc., provided that we get access to appropriate samples in sufficient numbers to test the effects of that many variables. As to gender, both the Gvar and Gwt cohorts had gender ratios near 1:1 (see Table S1). As mentioned above, both genders were near parity in the four bi-decadal Gwt age subcohorts as well.*

*Sample sources are stated in the Methods section entitled “Tissue samples and patient cohort” (p. 15). There was a mistake, wherein young and old Gwt sources were transposed. This was the source of the example of the 83 year-old with ACL surgery and has been corrected (l. 34-37). We thank the reviewer for catching this error.*

5. Page 4 Line 5. The mention of "statistically different" must be accompanied with a p-value and be represented in a figure. Further, the statistical test used must be identified, whether it be in the methods or as a case-by-case basis. This statement refers to Figure 1C, but there is no statistical test conducted on this figure. Modification of figure 1C must be conducted to first identify the presence of sequencing bias after each critical step in the LostArc method, as well as demonstrate if there are any differences between Gwt and Gvar at each step (i.e. show data separately for the two groups). Figure S1A attempts to address this issue, but with large variation between specimens, it is necessary to identify if the percent of mtDNA throughout the LostArc process is comparable between groups. The authors can best represent this with a double bar graph and the appropriate statistical tests. Figure S1B should use Spearman's correlation (not Pearson's) as the data distribution does not meet the assumption for a Pearson correlation with a few outliers exerting undue influence on the regression.

*Response:*

*We appear to have been unclear that the biases discussed are not between Gwt and Gvar, but instead systematic bias for or against mtDNA versus nDNA due to molecule size and/or topology selection by the library preparation. The first three bars in Fig. 1c are from qPCR quantification mtDNA and nDNA after DNA extraction (Step 1) and after the first (Step 3) and second (Step 4b) ExoV digests. Biases due to library preparation and sequencing would enter the pipeline at Steps 5-7. Therefore, we assess bias by comparing the products of Step 4b and Step 7. For a limited subset, we also measured DNA content after Step 1. Fig. S1a shows all samples for which all three measures exist.*

*We think the reviewer’s underlying point is that comparing the mean outputs of Step 4b and Step 7 (p > 0. 05; two-tail paired t-test) is insufficient to demonstrate a lack of sequencing bias. This is valid and we thank the reviewer for it. The offending sentence has been altered to reflect this and the legend for Fig. S1 now includes more relevant statistics.*

*The sentence in question (p. 4, l. 2) now reads “Library preparation and sequencing added no systematic bias for or against mtDNA (see Fig. 1c for averages, Fig. S1 for individual samples; see Methods for details).”*

*The legend for Fig. S1 now reads “A) Comparison of mtDNA content across samples and method steps (Fig. 1b). All samples were used where each measure is known. B) MtDNA fractions after read mapping (Fig. 1b, Step 7) correlate with fractions measured by qPCR (Fig. 1b, Step 4b; p = 0.00035, F-test of least squares linear regression). All samples were used where each measure is known. Clustering above or below the diagonal (dotted line) would indicate systematic bias. Such clustering is not significant (p > 0. 05; binomial test). Further, ratios of the two measures are centered near 1 (mean = 1.10; skew = 0.37, not significant, p > 0.05). The weakness of the correlation (Pearson r = 0.53; Spearman ρ = 0.41) cannot be fully explained by loss of PCR primer sites. Data from non-Gvar samples and commercially obtained Gwt DNA, used in pilot experiments, are included here for comparison but are not used in any other analyses.”*

6. Page 4 Line 9 (and elsewhere). What is "10-5.9 ± 0.09; 95% CL"? This is a highly unusual way to report summary statistics and the definition of CL is never provided. Data should be summarize as mean ± standard deviation, or mean ± 95% confidence interval if normally distributed (and these data are not always normally distributed), or median [interquartile range] if not normally distributed.

*Response: CL denotes "confidence limits". These data are log-normally distributed, so the confidence limits are symmetrical in the exponent (i.e. the median is 10^-5.9 and the upper and lower 95% limits are 10^-5.81 and 10^-5.99, respectively). This is now explicitly stated in the text (p. 4, l. 15).*

7. Page 4 Line 20-21. The authors claim that the correlation of the data in Figure 2A has an R2 < 0.002, but do not identify what test was used to generate that number. Correlation tests must show a p-value and either a Pearson's r or Spearman's rho.

*Response: R^2 is the square of the Pearson correlation coefficient. This was from a linear least squares regression, but this has little relevance, as the shotgun blast of data points in Fig. 2a has no other simple correlation either. With so little correlation a p-value is of little use.*

8. Page 4 Line 23-28. These data are not displayed anywhere in the manuscript, yet are referred to in the result section and the discussion. The authors must make a figure to show these results and the statistical tests.

*Response: These data are already in Table S1, as stated in the previous line.*

9. Page 4 Line 40-45. The Kolmogorov-Smirnov test is not useful to interpret the data in Figure 2C. To identify a relationship between deleted mtDNA and age, a correlation should be performed. Further, for the reader to interpret the t-tests performed on the binned age data, it must be shown in a separate figure, or show a dotted line that would separate the two age groups.

*Response: A nonlinear correlation across all ages would require a model, which we do not claim to have. The Kolmogorov-Smirnov test versus a uniform distribution says that ablation levels deviate from uniformity with regard to age. Subsequent comparisons show that ablation increases linearly after age 60. Vertical gridlines are already present for every 20 years of age.*

10. Page 4 Line 46-47. The relationship between age and mtDNA copy number is not demonstrated anywhere in the manuscript. Figure S1A does not address this relationship, and thus, as stated above, a figure must be included, or this statement removed.

*Response: This information was misplaced into the Methods section. It is now in Results sections “The LostArc pipeline, its outputs and the human mtDNA reference map” and “Levels of mtDNA do not vary significantly with age” (p. 3-4).*

11. Page 5 Line 2-14. This paragraph is all speculation as it does not directly follow that "ablation level must have been lower at the age of onset of symptoms." The authors can mention their assumption, but Figure 2E is too weak of an estimation to include and should be removed. Further, the mention of this analysis in the discussion on Page 13 Line 4-7 should also be removed.

*Response: The upper bound of ablation measured in older Gwt samples clearly corresponds with the minimum ablation estimated at disease onset in Gvar samples (Figs. 2d and 2e). We find this implied threshold too intriguing to leave unmentioned. Given that we explicitly lay out the assumptions and drawbacks underlying the estimates in Fig. 2e and state that they are “a simple first approximation,” we prefer to leave the figure as is. Also, we reworded the language in the second to last paragraph of the Discussion, changing a summary sentence from “…define a clinical threshold” to “…approximate a clinical threshold”.*

*This response directly relates to that for Reviewer 1, Minor Comment 6.*

12. Page 5 Line 20. In order to clearly show that "oriL and the 7S-DNA sequence are strongly preserved in Gvar and Gwt muscles", the authors should make the ablation patterns available for all specimens tested, not just four of them represented in Figure 2B. This can be done with an additional supplemental figure.

*Response: These are already included for each sample in the Supplemental Reports. We hope that the changes to the legends for the example reports make these easier to find (Figs. S4-5).*

13. Figure 3C. This reviewer applauds the author for their extensive data visualization. However, they should consider using a categorical colour map rather than a continuous one for Figure 3C and other similar figures. As is the figure is too complicated and chaotic; the green line denoting the common deletion is not visible and the message gets lost in the complexity. The Blue colour in Figure 3D is invisible due to poor contrast. Consider using a lighter blue.

*Response: In presenting this very rich and complicated data to different audiences we found that some viewers take away more information from the Bubble Plots and some from the Arc Plots. Some find both useful for different aspects of the data. The obscurity of the common deletion in Fig. 3c helps illustrate our point that the “common deletion” is rarely the most frequent and is not particularly special in this data set. This deletion may be easier to see as a green bubble in Fig. 3b. The blue shade in Fig. 3d is less prominent because we use the same color scale for all Clustering Arc Plots and M24 has less prominent clustering than some other samples.*

14. Page 5 Line 51-54. An example of the COX histology images used for analysis would be desirable. Further, in order to determine "best correlation", correlation tests must be conducted and reported as p-values and respective Pearson's or Spearman's rho in Figure S3B.

*Response: Example histology images are available in the literature for several Gvar samples (references in Table S2). R^2, as found in Fig. S3b, is the square of the Pearson correlation coefficient. These are from linear least squares regression, as seen in Fig. S3a. The following has been added (p. 5, l. 41): “…(Figs. S3a; p < 10-11, F-test of least squares linear regression)…”.*

15. Page 5 Line 62 - Page 6 Line 4. The authors claim that "since small deletions are markedly more frequent in muscle samples than in HEK cultures, they are included in overall deletion load and ablation calculations after HEK background subtraction". To my understanding, there are just as many small (>100bp) deletions in Figure S4 than in all other specimens analyzed. The authors should clarify this statement. Secondly, Figure 2A should not be referenced here as it is not relevant.

*Response: Fig. S4 shows HEK data. The frequencies of small deletions in all other samples (e.g. Fig. S5; all samples in LostArc Reports) are after HEK subtraction. Given that these frequencies are greater than zero, the frequencies in all other samples must have been higher than in HEK cells. The Fig. 2a reference has been clarified: “Since small deletions are markedly more frequent in muscle samples than in HEK cultures, they are included in overall deletion load and ablation calculations after HEK background subtraction (as in Fig. 2a).”*

16. Page 5 Line 30. The authors state that "the failure to more fully retain these OXPHOS sequences implies incomplete or absent mitophagy in postmitotic muscle fibers." This statement would belong in the discussion rather than the result section. Further, is it not possible that mitochondria contain multiple copies of mtDNA that complement each other functionally so that membrane potential is maintained despite the presence of mtDNA deletions? After all, the same argument could be made about mtDNA mutations. In the absence of intermembrane potential data supporting this statement, it is too strong and should be toned down. The absence of evidence is not the evidence of absence…

*Response: We thank the reviewer: this comment made us smile. Martin Rees' maxim is a favorite of the first author, an artefact of reading Carl Sagan as a child. We do not state that mitophagy does not function in post-mitotic muscle tissues. The impossibility of proving a negative is the reason that we were careful to say that the deletions patterns IMPLY rather than PROVE a lack of mitophagy. Our ample evidence for deleted mtDNA molecules in our muscle samples simply suggests that any mitophagy is insufficient to remove the deleted mtDNAs. The very next paragraph references evidence for complementation, which could delay or reduce the need for mitophagy. However, we believe that the argument underlying our assertion stands: any negative selection by mitophagy should leave a footprint in the deletion pattern, and the deletion pattern is evidence of absence of mitophagy to the detection level of the assay.*

*As an aside on complementation, mitochondria in skeletal muscles sort into at least two groups. Intermyofibrillar mitochondria and sub-sarcolemmal mitochondria. They can exist as a reticulum, due to fusion, but discrete mitochondrial organelles exist, too, which can be seen in electron micrographs. Fusion means that a reticulum must contain a mixture of mtDNAs. The degree of complementation in one of our samples is dependent on several variables, including the fraction of reticular versus discrete organelles, the number of nucleoids in each, and the number of mtDNA circles per nucleoid, as suggested by the reviewer. We are only really confident in the last point, as super-resolution microscopy has shown that there’s only ~1 mtDNA per nucleoid (PMIDs 21808029, 23721879) and cell biology with fused cells shows that mitochondrial nucleoids do not mix (PMID 28680532).*

17. Page 6 Line 36. A previous age-binning method was proposed prior to this one, the authors must choose one and use it for the entirety of the manuscript.

*Response: The two sets of bins serve different purposes, so we would prefer to keep them as is. The earlier bins were chosen based on the nonlinear shape of the data in Fig. 2c in order to describe that shape. In contrast, the analysis described on Page 6, with three axes of interest (age, deletion frequency, and terminal microhomology length (TMH)), was always going to be susceptible to reduced statistical power through multiple-hypothesis testing. Thus, in advance of analysis, we selected a small number of equally spaced cohorts with sufficient sample numbers in each.*

18. Page 6 Line 38. The authors note that the "following differences are significant (α = 0.05)", but do not indicate what statistical tests were performed. Further, the authors should modify Figure 3A to represent the age-binning used for the statistical tests and create a duplicate graph with the Gvar specimens to allow for visual comparison between groups.

*Response: We thank the reviewer for catching this omission. The line has been amended to “…Welch’s t-test with Šidák correction for multiple hypothesis testing…”. The figure already reflects age-binning used in these tests. We compared deletion frequencies for each TMH length for each age cohort in the figure to each other cohort. In the paragraph in question we report all pairs that passed the significance threshold after accounting for multiple-hypothesis testing. Gvar deletion spectra vary more by genotype than by age, so an equivalent graph would have little meaning. The most explanatory general component of Gvar deletion spectra does not fall along an axis of age (see PCA Component 1, e.g. Fig. 4d). Equivalent data are reported for each sample in the Supplemental LostArc Reports.*

19. Page 8 Line 7-19. The data in the paragraph do not refer to a figure. The authors must address this and make the statistical considerations obvious on a figure.

*Response: We suspect that the reviewer is referring to Page 9 lines 8-19, wherein we compare ablation levels between sample clades. The data are included and properly labelled in the figure but were not referenced in the legend. We thank the reviewer for catching this oversight. The legend now includes “Purple bars below the dendrogram illustrate ablation levels.”*

20. Page 9 Line 22 - Page 10 Line 9. The PCA portion of this manuscript is flawed and should be removed. The PCA as is does not specify all the variables considered and is only conducted on a subset of specimens. Such specimen "selection" is inappropriate as it most likely introduced bias that influences the results presented, something the authors hint at themselves. Typically, PCA analysis requires a larger sample size, especially when considering multiple different variables. The removal of this section would also lead to the removal of the following: all three mentions of PCA components on Page 10 Line 45-58, all mention of PCA analysis on Page 13 Line 52 - Page 14 Line 5, the paragraph on Page 14 Line 9-22, PCA in the methods on Page 21 Line 54 - Page 22 Line 24, Figure 4B-G, and any mention of PCA in the figure legends. It would also reduce the length of the manuscript, something needed, and perhaps some of the more interesting data presented in supplement could be brought back in the main text.

*Response: The legend for Fig. 4 states: “Hierarchical clustering revealed similarities between samples and Principal Component Analysis (PCA) revealed the underlying patterns that explain the most variation between samples. Both analyses used the fraction of deletions, binned by terminus positions (80 x 80 bins), rather than deletion frequencies or mtDNA ablation levels.”*

*We suspect that the reviewer’s objections are based on a misunderstanding of the underlying data. Neither age, nor genotype, nor ablation level were included as variables. The ONLY variable we considered was deletion fractions binned by terminus positions. These data are fundamentally simple (each sample’s data could be represented as a three-dimensional plot) but exceedingly rich (each sample has > 42,000 data points). This richness provides the large data set that makes PCA possible and appropriate. The simplicity is appropriate for the number of samples, provided that we look at very few PCA components. The insistence on looking at very few PCA components is why we had to select a representative subset of Gvar samples in advance. Otherwise we would risk spending the most important components on individual sample idiosyncrasies rather than the underlying patterns that we show have explanatory power (we still lost Component 2 to the idiosyncrasies of sample M24).*

*The PCA revealed the underlying patterns that explain much of the hierarchical clustering. We were both delighted and surprised by the agnostic identification of disease-correlated (Fig. 2d) and age-correlated (Fig. 2e) vectors by PCA. Without the PCA we may never have discovered the pattern (Component 1), which allowed us to test replication modes via Monte Carlo simulation (Fig. 5, Fig S6). PCA is important to the story of this manuscript, and we believe the readers are best served by keeping it in the manuscript.*

21. Page 11 Line 2-22. This section from "The 73-3' terminus…" to "counterclockwise directions" should be removed as it is unnecessary in the results, but the sentence "Each satisfied the following… in order to avoid overfitting" should be added to the Monte Carlo methods section.

*Response: This section was edited in response to reviewer comments above, which may negate this suggestion. If the editor prefers that the section be moved or eliminated we will do so.*

22. Page 11 Line 58-61. As mentioned before, the authors claim "mtDNA copy number did not vary significantly" between Gwt and Gvar individuals, but there is no data in the results to support this. The authors must add a figure with this information or remove the statement.

*Response: Representative values are in Fig S1A (blue bars). This is now emphasized in the text, and the reader is directed to Fig. S1a (p. 12, l. 28).*

23. Page 12 Line 28. The authors rightly point out that "Methods for further reducing or eliminating amplification require further study". Indeed, this remains a potential limitation in the present study, but is not discussed as such.

*Response: This section of the Discussion compares methods for preparing sequencing libraries for detection of mtDNA deletions. It reminds the reader that LostArc achieves mtDNA enrichment without long-range PCR amplification, which can inflate frequencies of random subsets of deletant mtDNA species. Because minimal amplification is a necessary step for Illumina paired-end sequencing, some amplification is unavoidable. Therefore, amplification may only be reduced by improvements to sequencing technology or larger samples, both of which are beyond the scope of this study.*

Minor Comments:

1. Page 3 Line 49-63. This section should be re-worked as it repeats the methods and what is shown in Figure 1B. The authors can simply remove the section and replace it with a sentence along the lines of "LostArc was conducted as outlined in Fig. 1b. For more details refer to the Materials and Methods."

*Response: The LostArc pipeline is itself one of the results of this study. We would prefer to leave this summary paragraph for readers who have not yet decided to read through the extensive Methods. If the editor agrees with the reviewer that the summary is redundant, we will remove it.*

2. Figure 1B. The authors should identify how the 30% cut-off for repetition of the nuclease treatment and purification was determined in Step 4b. Further, they should specify the number of specimens that underwent repetition in both the wild type POLG (Gwt) and variant POLG (Gvar) groupings.

*Response: The 30% cut-off was determined empirically during pilot experiments, balancing the expense (deeper sequencing) or risk (more amplification) for less enriched samples with the time, effort, expense, and sample losses inherent to repeated enrichment. Only 4 samples required a second round of enrichment, with no obvious patterns due to age, gender or disease state.*

3. Page 4 Line 3-4. The authors should define "very rare" and "ultra-rare" deletant species with the frequencies they represent.

*Response: The revised sentence (p.4, l. 11) now includes “… (boundary set at approximately 3 x 10-5 by the sensitivity achieved; see Methods) …”*

*The Methods now include “Deletion frequencies from subsamples of M22 correlate well with one another above individual rates of approximately 3 x 10-5 (Fig. S2d and e). Above this boundary lie very rare deletions, for which individual rates are confidently determined. Below this boundary lie ultra-rare deletions, for which collective rates are accurate but individual rates are subject to stochastic variation.” (p. 21, l. 6).*

4. Figure 2. The authors should consider removing the randomly highlighted Gwt points (M02 & M17) as well as highlight all different Gvar mutations or none at all.

*Response: M02 and M17 were selected to be touchstones, representatives of the oldest and youngest Gwt cohorts that the reader can use to orient themselves in various figures. See Figs 2a-d, 2g upper left, 4a, 4d-g, and 5h-i. Gvar samples are highlighted if multiple samples bear the same POLG genotype. Emphasizing these can highlight increasing ablation with age (Fig. 2d), consistent projected ablation at onset (Fig. 2e), trends in deletion patterns (Fig. 2g), clustering of patterns with mechanistic implications (Fig. 4a), the underlying aging component (Fig. 4e), and increasing deletion size with age (Fig. 5i). We believe it makes sense to be consistent between figures wherever possible.*

5. Page 4 Line 60-61. May be better suited in the discussion or removed.

*Response: This sentence uses general terms to describe three possible processes that would result in accumulation of deletions in mtDNA. Because some readers may be unfamiliar with molecular mechanisms of genome maintenance, we prefer to retain this sentence at the original position in the manuscript to guide the thinking of non-expert readers of this intricate study.*

6. Page 5 Line 22-32. May be better suited in the discussion or removed.

*Response: This paragraph reports results (ablation patterns, Fig. 2b) that are difficult to explain without knowing the underlying logic. We would prefer to leave a few guiding lines for the reader.*

7. Page 5 Line 37-48. This section from "First, we assume…" to "examples in Figs. S4 and S5" can be removed.

*Response: This is a similar situation to the previous comment, but for the logic underlying the correlation between ablation and COX deficiency. We would prefer to leave it where the reader first encounters these results.*

8. Page 8 Line 30. The authors should modify the wording of "in hope of uncovering" to "in an attempt to uncover".

*Response: We thank the reviewer for the suggestion. It has been done.*

9. Page 8 Line 33-40. This portion of the paragraph summarizes the methodology and is not necessary. The authors should remove this section, move "The Bubble and Arc Plots… (see Methods)" to the next section, and rename that section "Unsupervised exploratory analysis of mtDNA deletion patterns separate samples by age and POLG genotype."

*Response: We thank the reviewer for the suggestion. It has been done, though we ask the editor to confirm whether this is preferable.*

10. Page 10 Line 13-43. The very detailed description of the mtDNA replication modes is unnecessary as it is clearly demonstrated in Figure 5. This could be removed.

*Response: This description was requested by formal reviewers within our Institute, one of whom was selected for sequencing expertise rather than familiarity with mitochondrial processes. We would like to keep the description for such readers.*

11. Page 10 Line 62. The authors should make it obvious in Figure S6A which mtDNA replication mode each Monte Carlo model is representing by adding a column to the table.

*Response: We have now also made the relationships between replication models and simulations explicit in the Results (p. 11; also see Reviewer 1, minor comment #4). Additionally, this information is in the Fig. S6 legend, which states: “Models 1 and 2 are most relevant for replication-independent deletion mechanisms.” In the main text, deletion mechanisms unrelated to replication are mentioned. The legend continues: “Models 3 and 4 are most relevant for deletion mechanisms that depend on heavy strand replication (any replication mode in Fig. 5a-d). Model 5 is for deletions that depend on bidirectional replication from 7S-3’ (as in Fig. 5d). Model 6 is for deletions that depend on unidirectional replication from either oriL or 7S-3’ in roughly equal measures (best for Fig. 5a).”*

12. Page 17 Line 48-60. This paragraph may be better suited in the results.

*Response: We thank the reviewer for the suggestion. It has been done. The information is now in Results sections “The LostArc pipeline, its outputs and the human mtDNA reference map” and “Levels of mtDNA do not vary significantly with age” (p. 3-4).*

13. Figure 4A. The Blue colour depicting WT POLG and the Blue depicting POLG A467T are too similar.

*Response: We suspect that this may be a screen/printer effect. These are the same shades as in other figures. We prefer to keep them for the sake of consistency. In this panel, readers have the assistance of the labeled WT Clade, the presence of triangles indicating touchstone Gwt samples M02 and M17, and the segregation of these blue boxes into rows by variant.*

14. Figure 5I. Remove the line between two points.

*Response: The lines are meant, in part, to link points from samples with like POLG genotypes and clades.*

15. Figure legends should stand alone and not constantly refer to other figures.

*Response: This is a valid opinion with which we respectfully disagree, at least for this situation. The story told here is complex and the points made in each main body figure reinforce one another. There is also value in pointing out where a main body figure summarizes data that are available for each sample in the Supplement.*

16. Please ensure consistency in figure legends and x- and y- axes. State the statistical tests utilized in each figure and whether any errors bars present represent.

*Response: All axes are fully labeled, including units where applicable. All error bars are explained in the corresponding legends. Statistical tests are confined to the text where they are discussed.*

**Second round of review**

**Reviewer 1**

the authors addressed my concerns.

**Reviewer 2**

The authors have addressed several of the points brought up, and the manuscript has been improved; however, I still have some concerns.
General Summary Comment 1) In regard to assay reproducibility, the authors demonstrate the correlation between two different muscle subsamples from the same participant and two DNA subsamples from the same muscle subsample from the same participant. However, the authors should acknowledge in their manuscript that more experiments are needed to be done in the future to determine the intra- and inter-assay coefficient of variations (CVs) for this assay. The inter-assay CV can be done as more runs accumulate.
General Summary Comments 2 & 3) In regard to background estimation and large mtDNA deletions, I acknowledge the authors point on the rho° cells and agree. However, it would be of interest in the future to conduct a LostArc on these cells to confirm the points brought up by the authors. I understand that these cells would not be useful for estimating mappability factors or setting an upper bound on sequencing noise, but they could be most useful for confirming that the large 15kb deletions are not actually artefacts caused by nuclear DNA (NMPs) following incomplete digestion of the nuclear DNA. These large deletions that include the origin of replication are highly implausible.
Specific Major Comment 2) According to the authors’ hypothesis communicated in response to point 5 of the General Summary Comments, they note that mtDNA point mutations are involved in subsequent mtDNA deletions and thus should be acknowledge in this section. A slight modification to the sentence “However, as assayed in human brain tissue, the accumulation of point mutations is orders of magnitude too low to explain the phenotypes of aging on their own (24)” could suffice.
Specific Major Comment 5) I do acknowledge that the bias examined was for systemic bias of the library preparation, but it would be of further interest to examine if any systemic bias present is the same or different between the two groups. Instead of lumping them together and claiming that there is no bias, examining each separately and concluding that there is no bias in each group, as well as no difference between the groups, would be a stronger argument. I bring this up as it is very obvious in Figure S1A that the Gwt specimens M10, M14, M13, M19, M15, and M12 show very little loss, and even some small gains, of % mtDNA from post-ExoV to the final mapped reads whereas two of the Gvar specimens (M27 and M32) show huge loss of % mtDNA and M25 shows a large increase. This is further demonstrated in Figure S1B where clustering of Gwt above the line and clustering of Gvar below the line are demonstrated. Thus, to increase transparency, the authors should present their data as a double bar graph in the place of Figure 1C, previously mentioned in the original review.
Specific Major Comment 11) Please modify the y-axis of Figure 2E to further indicate that the data is an estimation, “Estimated mtDNA Deleted” would suffice.

**Authors Response**

**Point-by-point responses to the reviewers’ comments:**

Reviewer #1:

the authors addressed my concerns.

*Response: We sincerely thank the Reviewer for taking the time to evaluate our detailed rebuttal.*

Reviewer #2:

The authors have addressed several of the points brought up, and the manuscript has been improved; however, I still have some concerns.

1)	General Summary Comment 1) In regard to assay reproducibility, the authors demonstrate the correlation between two different muscle subsamples from the same participant and two DNA subsamples from the same muscle subsample from the same participant. However, the authors should acknowledge in their manuscript that more experiments are needed to be done in the future to determine the intra- and inter-assay coefficient of variations (CVs) for this assay. The inter-assay CV can be done as more runs accumulate.

*Response: To address the reviewer’s concern, we added two new supplemental figures to our first revision of the manuscript that document LostArc reproducibility between DNA and tissue subsamples* ***(****Figs. S2d and S2e****;*** *slopes and R^2^ values very near 1). With respect to calculating coefficients of variation for deletion frequencies, we note that estimates of individual deletion frequencies are independent of one another and vary from 10^-7 to 10^-1 within a sample. Accordingly, we calculated an inter-assay coefficient of variation (relative deviation) from technical replicate samples spiked with plasmid standards containing mtDNA sequence with artificial deletion junctions. We have added a sentence to the legend of Fig. S2c, stating “Note: the relative deviation was 65% for repeated measures (n = 5) of the 2,392 bp deletion at an input frequency of 10^-4^.” Similarly, we calculated intra-assay coefficients of variation for two sub-samples of muscle sample M22 that we binned by order of magnitude. We have added sentences to the legend of Fig. S2e, stating “Errors are distributed normally for deletion frequencies over 10^-6^, with standard deviations (per bin) varying linearly with frequency (R^2^ > 0.99). Assuming an intercept at zero, this means that the relative deviation is essentially constant. Regression puts the relative deviation at 59%, but this is dominated by high frequency bins. The average relative deviation across bins is 78%.”.*

2)	General Summary Comments 2 & 3) In regard to background estimation and large mtDNA deletions, I acknowledge the authors point on the rho° cells and agree. However, it would be of interest in the future to conduct a LostArc on these cells to confirm the points brought up by the authors. I understand that these cells would not be useful for estimating mappability factors or setting an upper bound on sequencing noise, but they could be most useful for confirming that the large 15kb deletions are not actually artefacts caused by nuclear DNA (NMPs) following incomplete digestion of the nuclear DNA. These large deletions that include the origin of replication are highly implausible.

*Response: When we first detected mitochondrial genomes with very large deletions, we assumed these rare molecules were dead end deletion products because they lacked one of the two accepted origins of mtDNA replication. We were delighted at the sensitivity of LostArc, and somewhat astonished to detect what we believed to be a non-replicating DNA molecules. Like the Reviewer, we were also concerned about nuclear mitochondrial pseudogenes (abbreviated NMPs or NUMTs) mimicking mtDNA bearing a large deletion. However, after reviewing calculations previously described in our original rebuttal document, we again conclude that even a NUMT present within every cell of a muscle sample would exist at a frequency of ~4 x 10^-6, which is near the lower limit of detection for individual deletions by LostArc, below the cutoff for ultra-low deletions stated in the paper, and too rare to be visible in Arc or Bubble plots or to affect any analyses presented.*

3)	Specific Major Comment 2) According to the authors' hypothesis communicated in response to point 5 of the General Summary Comments, they note that mtDNA point mutations are involved in subsequent mtDNA deletions and thus should be acknowledge in this section. A slight modification to the sentence "However, as assayed in human brain tissue, the accumulation of point mutations is orders of magnitude too low to explain the phenotypes of aging on their own (24)" could suffice.

*Response: Thank you. We believe the reviewer is conflating nucleotide misinsertion with generation of point mutations. These are related but separate events. Insertion of incorrect nucleotides by a DNA polymerase can promote replisome stalling. Such stalling increases the probability of either (1) mispair extension or (2) replisome disassociation and relocation downstream. The first results in a point mutation in the next round of replication. The second creates a template strand loop and results in a deletion in the next round of replication. As stated in the original manuscript, mtDNA point mutations and deletions accumulate with age and may be caused by base-base mispairs. However, point mutations do not promote the subsequent formation of deletions.*

*In our first revised manuscript, we attempted to address Specific Major Comment 2 from Reviewer 2 by adding a supporting literature reference that describes how “point mutations do not limit the natural lifespan of mice” (Vermulst et al., Nat Genetics 2007; ref. #25). We hope this explanation and our modification of the third paragraph of the Introduction are sufficient to address this concern of Reviewer 2.*

4)	Specific Major Comment 5) I do acknowledge that the bias examined was for systemic bias of the library preparation, but it would be of further interest to examine if any systemic bias present is the same or different between the two groups. Instead of lumping them together and claiming that there is no bias, examining each separately and concluding that there is no bias in each group, as well as no difference between the groups, would be a stronger argument. I bring this up as it is very obvious in Figure S1A that the Gwt specimens M10, M14, M13, M19, M15, and M12 show very little loss, and even some small gains, of % mtDNA from post-ExoV to the final mapped reads whereas two of the Gvar specimens (M27 and M32) show huge loss of % mtDNA and M25 shows a large increase. This is further demonstrated in Figure S1B where clustering of Gwt above the line and clustering of Gvar below the line are demonstrated. Thus, to increase transparency, the authors should present their data as a double bar graph in the place of Figure 1C, previously mentioned in the original review.

*Response: During development of LostArc protocols, we carefully monitored enrichment of mtDNA at various steps of library preparation (Fig. 1C). Following the development period our efforts expanded to process all the samples needed for the study, and we continued to ensure proper enrichment by assessing mtDNA content after the final two steps. We appreciate that Reviewer 2 asks us to sort the data further to search for any systematic enrichment bias for/against Gwt and Gvar samples. Unfortunately, data from intermediate enrichment steps does not exist for a sufficient number of samples to allow us to modify Fig. 1C as requested.*

*We have been transparent on this issue. We show bar graphs for all samples where we had all three measures (Fig S1a), and we show a scatter plot for all samples where we had the two final measures (Fig S1b). As stated in this Figure legend in the original submitted manuscript, “All samples were used where each measure is known.”. In our first revision of the manuscript, we explicitly note the reviewer’s concern by writing, “Several samples, including one Gwt (M05), had lower mtDNA fractions than indicated by qPCR (****Fig. S1b****). These samples were not unified by age or genotype, so the cause of this effect is unknown.” Lastly, we believe the enrichment pattern is best presented in our original scatter plot showing all data points (Fig. S1b), rather than a bar graph that groups Gwt and Gvar samples.*

5)	Specific Major Comment 11) Please modify the y-axis of Figure 2E to further indicate that the data is an estimation, "Estimated mtDNA Deleted" would suffice.

*Response: This is an excellent suggestion. Thank you. We have changed the label on the y-axis of Figure 2E from “mtDNA deleted” to “estimated mtDNA deleted”. This change should help readers to distinguish measured values (Fig. 2D) from extrapolated values (Fig. 2E).*
